# The impact of the protein interactome on the syntenic structure of mammalian genomes

**DOI:** 10.1371/journal.pone.0179112

**Published:** 2017-09-14

**Authors:** Isa Kristina Kirk, Nils Weinhold, Søren Brunak, Kirstine Belling

**Affiliations:** 1 Novo Nordisk Foundation Center for Protein Research, Faculty of Health and Medical Sciences, University of Copenhagen, Copenhagen, Denmark; 2 Memorial Sloan Kettering Cancer Center, Computational Biology Program, New York, NY, United States of America; Centre for Research and Technology-Hellas, GREECE

## Abstract

Conserved synteny denotes evolutionary preserved gene order across species. It is not well understood to which degree functional relationships between genes are preserved in syntenic blocks. Here we investigate whether protein-coding genes conserved in mammalian syntenic blocks encode gene products that serve the common functional purpose of interacting at protein level, i.e. connectivity. High connectivity among protein-protein interactions (PPIs) was only moderately associated with conserved synteny on a genome-wide scale. However, we observed a smaller subset of 3.6% of all syntenic blocks with high-confidence PPIs that had significantly higher connectivity than expected by random. Additionally, syntenic blocks with high-confidence PPIs contained significantly more chromatin loops than the remaining blocks, indicating functional preservation among these syntenic blocks. Conserved synteny is typically defined by sequence similarity. In this study, we also examined whether a functional relationship, here PPI connectivity, can identify syntenic blocks independently of orthology. While orthology-based syntenic blocks with high-confident PPIs and the connectivity-based syntenic blocks largely overlapped, the connectivity-based approach identified additional syntenic blocks that were not found by conventional sequence-based methods alone. Additionally, the connectivity-based approach enabled identification of potential orthologous genes between species. Our analyses demonstrate that subsets of syntenic blocks are associated with highly connected proteins, and that PPI connectivity can be used to detect conserved synteny even if sequence conservation drifts beyond what orthology algorithms normally can identify.

## Introduction

Conserved synteny refers to the preserved gene order between species [1]. The prevalence and genomic positions of conserved syntenic blocks have been extensively studied using sequence conservation and conserved gene order between genomes [2–4]. Certain genes tend to stay together throughout evolution and remain as conserved syntenic blocks across a wide range of species. Functional relationships are possible driving forces for synteny to be conserved over millions of years, e.g. regions that are under the same regulatory control [5–7].

The interactome is the term for all known physical interactions within a genome, including protein-protein interactions (PPIs) [8]. The understanding of whether genes in conserved syntenic blocks interact more closely at the level of gene product interactions is still limited. It is, however, well established that the topology of PPIs is highly conserved throughout evolution, and in many instances exceeds the conservation of protein sequence and structure [9,10]. It is therefore of relevance to investigate whether PPIs act as a functional driver for conserved synteny.

Here we examine the pattern of PPIs in conserved syntenic blocks. We used both an orthology-based and a connectivity-based approach to define syntenic blocks. We did not find PPI connectivity to act as a global driver of synteny. However, we did find a small number of blocks with significantly higher connectivity than expected. More interestingly, we discovered that the functional approach, i.e. the connectivity-based approach, could point to potential orthologs between species.

## Results

### Orthology-based conserved syntenic blocks in the human genome

**Fig 1:**
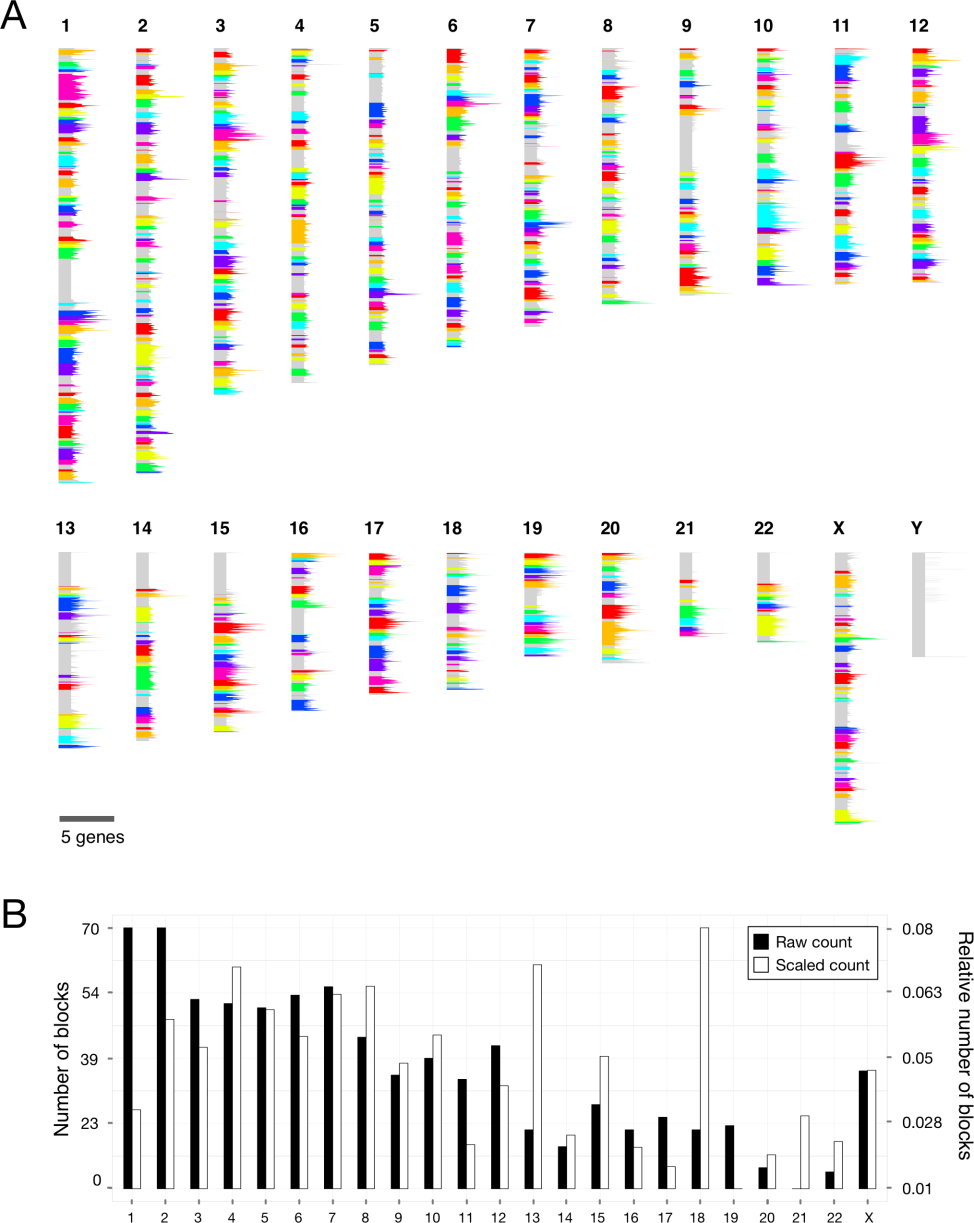
Chromosomal positions and gene counts of orthology-based conserved syntenic blocks. (A) A total of 829 conserved syntenic blocks in the human genome were identified by finding genomic regions with preserved order of orthologous genes across five mammalian species. The blocks are marked in alternating, randomly chosen colors. Grey color represents non-syntenic regions. The amplitude on the chromosomes indicates density of protein-coding genes per bin (chromosome length/1,000). The amplitude scale in the bottom right corner marks the maximum amplitude of five genes in a bin. Chromosome Y had no conserved syntenic blocks due to lack of sufficient orthologous sequences. (B) The distribution of the syntenic blocks across the human chromosomes. Black bars indicate the total number of blocks per chromosome (left Y axis). White bars indicate numbers of blocks relative to the number of protein-coding genes per chromosome (right Y axis).

Conserved syntenic blocks were defined in the human genome as regions with preserved order of orthologous protein-coding gene across the five species: human, chimpanzee, mouse, pig and dog. A total of 362, 581, 600 and 499 pairwise syntenic blocks were identified when comparing human to chimpanzee, mouse, pig, and dog, respectively (S1 Fig, S2 Fig and S1–S4 Tables). The pairwise syntenic blocks were merged into conserved syntenic blocks by intersection of genomic coordinates using the human genome as reference. For both the pairwise and the conserved syntenic blocks a requirement of minimum two genes separated by a maximum of 1Mb was used to define blocks, see Methods. In total 829 conserved syntenic blocks covered 55.29% of the human genome, i.e. these blocks had preserved gene order in all five mammals (Fig 1A, Table 1 and S5 Table). The blocks contained known conserved gene loci, e.g. the globin loci on human chromosome 11 (block 529) and the two T cell loci beta and gamma on human chromosome 7 (blocks 399 and 367). No conserved syntenic blocks were defined on chromosome Y since the sequence is still unavailable for the dog genome. The conservation of chromosome Y is therefore rather low, which is in agreement with the current understanding of the evolution of this chromosome [11].

**Table 1:** Number of orthology-based syntenic blocks and their gene counts.

Comparison	Number of blocks	Orthologous genes	Non-orthologous genes	Genes in total
Human—Chimpanzee	362	17,173	2,439	19,612
Human—Mouse	581	16,231	3,003	19,234
Human—Pig	600	14,048	4,607	18,655
Human—Dog	499	15,773	3,618	19,391
Conserved syntenic blocks	829	11,576	5,979	17,555

As expected, conserved syntenic blocks were most abundant in the longest human chromosomes 1 and 2, while the shortest chromosome, 21, had fewest blocks. The trend changed upon normalizing by the number of protein-coding genes per chromosome. Here, chromosomes 13, 18 and 21 had a high block-to-gene ratio of which chromosome 18 had a significantly higher ration than all other chromosomes (p-value = 0.02). Further, chromosomes 1, 11 17, and 19 had a lower ratio although none of these were significantly low (Fig 1B). A high number of conserved syntenic blocks indicated either extensive genomic fragmentation or sequence evolution since orthologous genes were used here to define blocks.

### Functional preservation in conserved syntenic blocks

**Fig 2:**
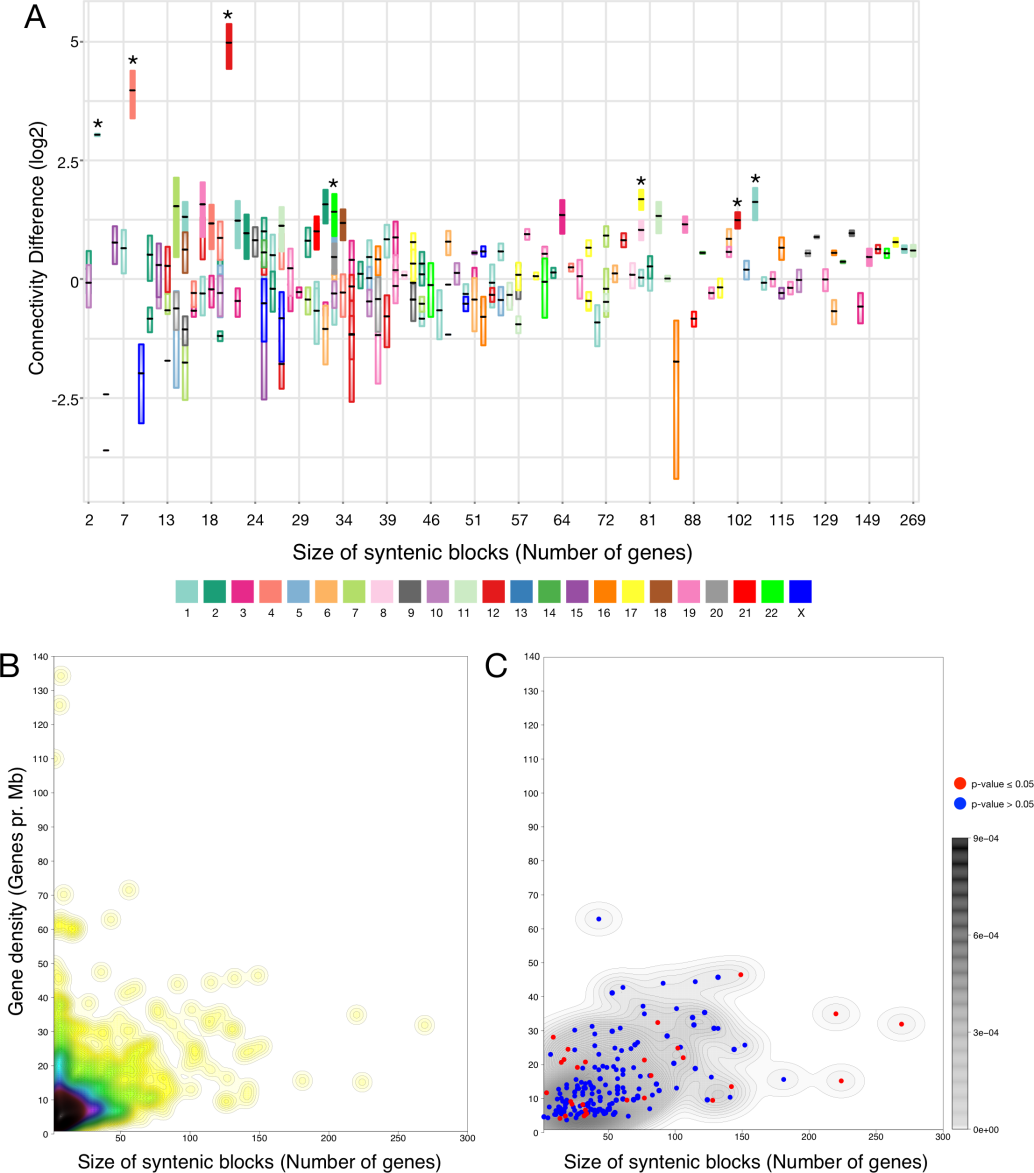
Connectivity and gene density of conserved syntenic blocks. (A) The connectivity ratio (CR) per block was calculated as the number of observed cis-PPIs divided by the number of observed trans-PPIs, each normalized by the theoretical number of cis- and trans-PPIs. The plot shows the 192 blocks with high-confidence PPIs. Each bar represents a block where the median (black horizontal line) and standard error (bar height) for the CRs were calculated from the three different confident sets of PPIs. The true CRs were divided by the median CRs from random blocks of the same length. Thus, blocks where the true CR was not different from the median of the randomization had a connectivity ratio (Y axis) around 0. We did not observe a global trend of increased CR for the 192 blocks. Some blocks (n = 29) did show significantly increased CR compared to random (marked with fill color), but only seven blocks (3.6%) were significant after correction for multiple testing (BH adjusted p-values ≤ 0.05). These are marked with asterisks (*). (B) The gene density (genes per Mb) compared to block size (number of genes per block) for all the conserved syntenic blocks (n = 829). The block with the highest gene density (229 genes per Mb) is not illustrated due to scaling. (C) Density plot limited to blocks with high confidence PPIs (n = 192). Some general trends and characteristics of gene number and gene density in the blocks were observed. However, numerous outliers were also observed showing either gene richness and low average gene density, or gene poorness with high gene density. Blocks with higher CR than expected at random (n = 29) are marked red.

Conserved syntenic blocks constitute a measure of genome preservation. Here we investigated whether the conserved syntenic blocks harbor functionally related gene products in form of high connectivity through PPIs. A connectivity ratio (CR) for each block was determined as the number of within-block PPIs (termed cis-PPIs) relative to the number of PPIs between block proteins and proteins encoded elsewhere in the genome (termed trans-PPI). Cis- and trans-PPI counts were normalized by the possible number of cis- and trans-PPIs for each block, which allowed for direct comparison of blocks of arbitrary lengths. Since PPI analysis is sensitive to confidence scoring we accessed the robustness of connectivity by using three different sets of PPIs in the analysis: a low, a high, and a very high confidence set (see Methods). CRs were calculated for blocks with high-confidence PPIs (192 blocks). Of these blocks, 29 had a CR higher than expected by random (15.1%) and seven (3.6%) were significant after correction for multiple testing (BH adjusted p-values ≤ 0.05, Fig 2A). Clustering coefficients of all high-confidence PPIs in the entire interactome (the global protein network) and in the complete synteny network were very similar (Table 2). We thereby confirmed that the PPIs in the conserved syntenic blocks included in the analysis had similar properties as the global protein network. On average, we found 4.4 connected components per block in the complete syntenic network. The internal synteny network, which only included cis-PPIs, was more densely connected than the complete syntenic network when looking at the per block numbers (Table 2).

**Table 2:** Network statistics for the global protein network (GPN), the complete synteny network (CSN) and the internal synteny network (ISN).

	GPN	CSN	ISN	CSN per block^a^	ISN per block^a^
Nodes	12,767	12,341	1,159	505.52 ± 583.11	6.04 ± 8.36
Edges	291,722	271,142	875	627.62 ± 936.75	4.56 ± 9.06
Connected components	12	13	401	4.40 ± 2.88	2.09 ± 1.98
Average degree	45.70	43.94	1.51	2.11 ± 0.37	1.21 ± 0.34
Clustering coefficient	0.29	0.28	0.09	0.02 ± 0.05	0.04 ± 0.15

^a^Mean values per block with standard deviations.

Conserved syntenic blocks differed with respect to the number of genes and gene density. Most blocks had up to 50 genes and a gene density up to 30 genes per Mb (Fig 2B). The conserved syntenic blocks without observed PPIs (n = 637) were in general gene poor with a gene density below three genes per Mb and on average ten genes per block. Blocks with high confidence PPIs (n = 192) were in contrast more diverse and blocks with significantly high connectivity were found in all lengths (Fig 2C).

**Fig 3:**
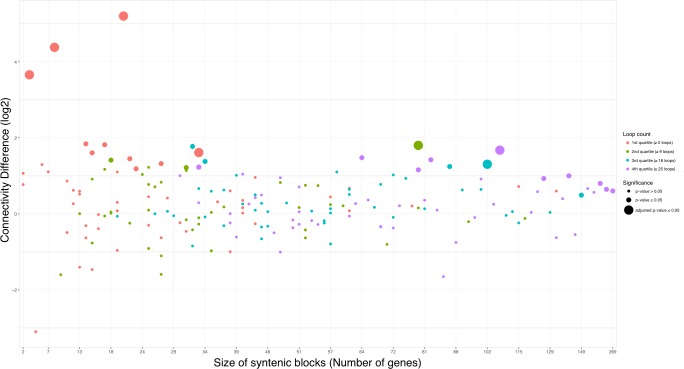
Distribution of chromatin loops in conserved syntenic blocks. Rao *et al*. (2014) defined chromatin loops associated with coordinated gene expression. Shown is the distribution of chromatin loops per conserved syntenic block. The colors represent quartile 1–4 for the loops per block distribution. Dot size indicates whether the block had significantly high connectivity or not. We found an enrichment of chromatin loops in the conserved syntenic blocks with observed high confident PPIs, but no correlation between increasing connectivity and increased loop count.

Conserved syntenic blocks have previously been associated with coordinated gene expression [5–7]. A study by Rao *et al*. (2014) [12] identified 9,448 chromatin loops at 5kb resolution conserved across cell types and species studied. These loops were found to frequently link promoters and enhancers while correlating with gene activation [12]. We found a higher proportion (69.65%) than expected by chance of these loops inside the syntenic blocks. Significantly more loops (p = 2.2e-16) were observed in the 192 blocks with high-confidence PPIs (Mean = 18.82 loops, standard error (SE) = 1.04) compared to the remaining blocks (Mean = 4.96 loops, SE = 0.25). Yet, we did not observe any correlation of increasing numbers of loops with increasing block size (number of genes) nor with increasing connectivity in the 192 blocks (Fig 3). Hence, no correlation of high connectivity and high loop counts, and thus coordinated expression was observed.

High connectivity in conserved syntenic blocks indicated functional relatedness. The five blocks with highest connectivity compared to random blocks included clusters of gene families and functional enrichment: Block 54 encoded four gene products that are all part of the immunological complement and coagulation cascades, and three of the genes are associated with the Gene Ontology category “immune process”; block 213 contained the casein gene cluster previously shown to be co-expressed [13], further all the genes are associated with the Gene Ontology category “extracellular region” fitting with the cluster being a phosphor proteins; block 562 included four C-type lectin domain containing receptors and five Killer cell lectin-like receptors gene family members and the block was enriched in the Gene Ontology molecular function category “carbohydrate binding” (p = 1.41e-14); block 784 was enriched in Class A Rhodopsin-like G-protein coupled receptors (p = 3e-2 for Gene Ontology category “adenylate cyclase-modulating G-protein coupled receptor signaling pathway”); and block 715 was enriched in the Gene Ontology molecular function category “single stranded RNA binding” (p = 1.68e-4) and the cellular component categories “PRC1 complex”, “nuclear ubiquitin ligase complex”, “filamentous actin” and “PcG protein complex” (p = 1.07e-5, 1.19e-5, 1.52e-4 and 6.3e.4 respectively). Information for all blocks regarding chromosome location, connectivity, genes, HGNC family and first level of Gene Ontology associated terms are available in S6 Table.

### Definition of connectivity-based conserved syntenic blocks

Traditionally, conserved synteny has been studied by sequence comparison. Yet, this approach is not optimal in all cases when studying functional conservation since protein function can be more conserved than protein or DNA sequences. To overcome these limitations, a reverse approach can be utilized, that is to define synteny using molecular data covering the functional relationship of interest, here connectivity through PPIs. In the reverse approach synteny was therefore defined using PPI data alone. Using the connectivity-based approach, a total of 163 blocks were identified. This number was similar to the number of orthology-based blocks with high-confidence PPIs (n = 192). The two types of blocks were highly overlapping with similar genomic borders and sizes. A weak tendency was observed for the orthology-based blocks to be included in the connectivity-based blocks, i.e. the connectivity-based blocks were in general larger.

A total of 16 connectivity-based blocks had significant high connectivity compared to random blocks of the same size of which ten were significant after correcting for multiple testing (BH adjusted p-values ≤ 0.05). Six of these blocks overlapped with orthology-based blocks with a non-significant connectivity. Thus, not being restricted to orthology revealed additional functional conservation. Interestingly, one block with significant connectivity was solely detected using the connectivity-based approach. This block (#335) was located on human chromosome 3 and contained four protein-coding genes *Roundabout guidance receptor 1 (ROBO1)*, *Roundabout guidance receptor 2 (ROBO2)*, *FSHD region gene 2 family member C (FRG2C)*, and *Zink finger protein 717 (ZNF717)*. This block was not defined based on orthology since *ZNF717* does not have orthologs in neither of the four other genome assemblies used in this study. Yet, blasting the human protein-coding sequence against the chimpanzee proteome and genome revealed a predicted protein with accession XP_016796982 with 98% identity match and located in the same region as the other genes. In the current chimpanzee genome assembly (3.0) the *ZNF717* (NCBI Gene ID: 460515) has been characterized in the same location as predicted.

**Fig 4:**
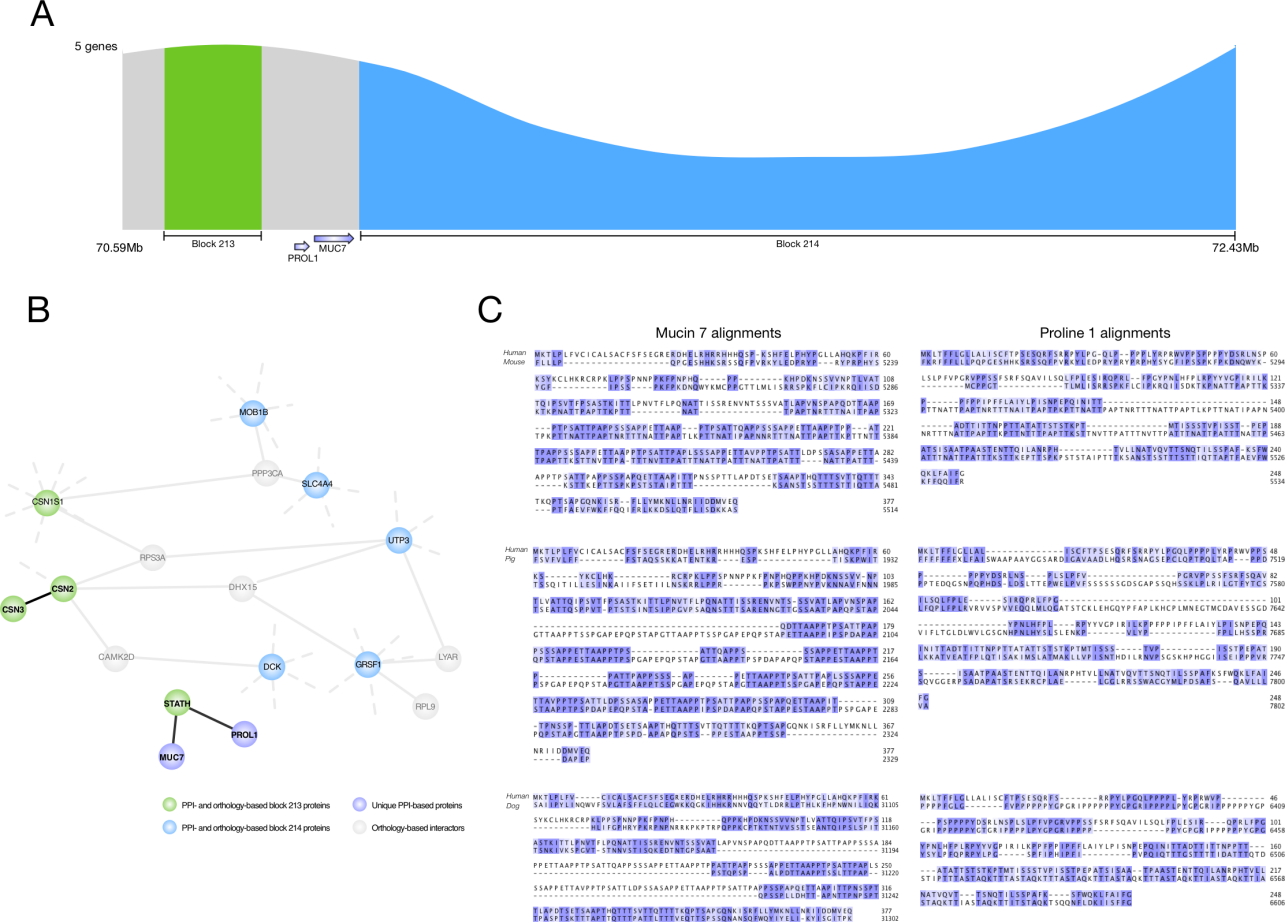
Connectivity-based syntenic blocks can identify protein-coding genes across species. (A) The connectivity-based block 442 included orthology-based block 213 (green) and 214 (blue) along with the intermediate region and an upstream flanking region (both grey). (B) The protein-protein interaction (PPI) network for block 442. The nodes are colored by the location of the protein-coding gene. Purple: unique for block 442, green: also in orthology-based block 213, blue: also in orthology-based block 214, and grey: in another connectivity-based block on the same chromosome. Black edges represent PPIs unique for block 442. PROL1 and MUC7 make the small network that identified block 442 as syntenic. Their genomic location in the intermediate stretch could include orthologous genes in the compared species despite not yet being identified. (C) Protein alignments of the human *PROL1* and *MUC7* against the translated intermediate block region in pig, dog and mouse. These alignments revealed sequence conservation.

Another interesting connectivity-based block was block 442. This included orthology-based blocks 213 and 214 with two surrounding genome stretches (Fig 4A). Block 442 was primarily defined based on PPIs between *Statherin* (*STATH*), *Prolin-rich lacrimal 1* (*PROL1*) and *Mucin 7* (*MUC7*) (Fig 4B), all located in the intermediate region of orthology-based blocks 213 and 214. This intermediate region was not included in any orthology-based blocks since orthologs of *PROL1* have not yet been identified in the pig and dog genomes, neither has a *MUC7* ortholog been identified in the mouse genome. Blasting all six possible reading frames of the intermediate region for species against the human genome revealed protein sequences that matched the human proteins encoded by *PROL1* and *MUC7*. Alignment of both the known and potential orthologous sequences indeed revealed sequence conservation (Fig 4C). Together these results suggested that the connectivity-based definition of synteny can be used to detect or verify new protein-coding genes.

## Discussion

The preservation of conserved syntenic blocks is well studied at the DNA and amino acid sequence levels [14–16]. Ancestral genome reconstruction by cross-species chromosome painting and comparative genomic approaches have collected evidence that conserved syntenic associations extend back approximately 360 million years [17,18]. Most studies have focused on the conservation of gene architecture, while only a few have explored the underlying biological reasons for the selective pressure that keeps genes in close proximity throughout evolution. Several studies have associated conserved blocks with coordinated gene expression [5–7]. Yet, multiple functional drivers might influence conserved synteny of which we were interested in investigating whether PPIs are one of them. In this study, we found that PPIs are not a general driver of conserved synteny. Only a few syntenic blocks had significantly high PPI connectivity. Although the orthology-based blocks with high confidence PPIs had significantly more chromatin loops than the remaining blocks, no correlation was observed between high connectivity and coordinated expression.

Conserved syntenic blocks defined by orthology are influenced by genome coverage and annotation. Orthologous genes are based on sequence similarity that might not always imply functional conservation since some genes may have diverged in sequence while still encoding a functionally conserved protein. Defining connectivity-based syntenic blocks based on PPIs confirmed that the orthology-based study of synteny revealed functional conservation and missing annotation indeed influenced the number of orthology-based syntenic blocks.

Non-coding genes also have a strong impact on genome function [19] and synteny studies have been used to detect conserved non-coding RNAs across species [20]. A recent study investigated structured non-coding RNAs in syntenic blocks in the pig genome [21]. However, as the relationship between non-coding RNAs and their targets in some cases is promiscuous and not fully understood, it is at present difficult to perform a functional study of conserved synteny with a focus on non-coding RNA. To our knowledge, no study has yet enlightened the role of non-coding RNAs in the conservation of gene order in syntenic blocks.

The increasing coverage of the human proteome [22,23] and interactome will enable an even better genome-wide study of the conservation of PPIs in conserved syntenic blocks. This study demonstrates the feasibility of an approach defining conserved synteny based on functional relationships. Using the same approach with other data types might reveal still unknown functional relationships preserved in conserved syntenic blocks. Although PPI connectivity did not appear as a general driver of conserved synteny, some syntenic blocks still showed high connectivity. The connectivity-based approach presented here revealed new preserved functionalities that cannot be found by the traditional orthology-based methodology. Thus, not only can PPIs increase detection of potential syntenic blocks but it can also support gene-finding efforts and add supportive evidence for putative open reading frames. Further, knowledge of the location of conserved syntenic blocks could similarly be used in the confidence scoring of PPIs, thus, in turn, refining the general PPI map used in studies like these.

## Methods

### Orthology-based conserved syntenic blocks

Orthology-based conserved syntenic blocks were defined in two steps with an approach inspired by Cinteny [24], SyntenyTracker [25] and i-ADHoRe [22,23]. First, pairwise syntenic blocks were defined by comparing the order of orthologous protein-coding genes in the human genome (assembly GRCh37.p8) with the chimpanzee genome (assembly Chimp2.1.4), the mouse genome (assembly GRCm38), the pig genome (assembly Scrofa10.2), and the dog genome (assembly CanFam3.1), respectively (S1 Fig). All data were extracted from Ensembl [26]. Pairwise syntenic blocks were defined as minimum two genes separated by less than a gap size of 1Mb. To account for micro-rearrangements, i.e. insertions, deletions and transversions, a second step was implemented. Here, the initial blocks were concatenated if a distance less than the maximum gap size of 1Mb separated them. Last, conserved syntenic blocks were defined in the human genome as common overlap of the four sets of pairwise syntenic blocks. Similar gap size and concatenation approach as in the pairwise comparisons were used in this step.

### Protein-protein interaction data

The InWeb_IM [27] interactome data is a network of human PPIs based on experimental interaction data from humans and model organisms extracted from various protein interaction resources. All interactions in InWeb_IM are scored and benchmarked against a gold standard. In this study we excluded self-interactions, histone proteins and ubiquitin C leaving 1,364,024 PPIs. Further, only one member was included from each family of tandem duplicated genes registered in Duplicated Genes Database [28,29]. Three sets of PPIs were used in our analyses to assess robustness: A low confidence set (confidence score ≥ 0.05; n = 396,959), a high confidence set (confidence score ≥ 0.1; n = 251,401, recommended cut off based on benchmarking), and a very high confidence set (confidence score ≥ 0.15; n = 195,207).

### Protein-protein interaction connectivity

To address whether proteins encoded within the same block interact more within-block (cis-PPIs) than with proteins encoded elsewhere in the genome (trans-PPIs), we investigated the PPI connectivity of proteins encoded in each conserved syntenic blocks. The connectivity analysis was performed on blocks having minimum one high confidence cis-PPI and one high confidence trans-PPI. The CR was calculated for each conserved syntenic block as the number of observed cis-PPIs divided by the number of observed trans-PPIs at each confidence score cut off. The observed counts were normalized by their corresponding theoretic counts of PPIs as in Eq (1).

$$\mathrm{CR}=\frac{{\mathrm{PPI}}_{\mathrm{cis},\mathrm{obs}}/{\mathrm{PPI}}_{\mathrm{cis},\mathrm{theo}}}{{\mathrm{PPI}}_{\mathrm{trans},\mathrm{obs}}/{\mathrm{PPI}}_{\mathrm{trans},\mathrm{theo}}}$$ (1)

where PPI_cis,obs_ and PPI_trans,obs_ were the observed cis- and trans-PPIs extracted from InWeb_IM. The theoretic counts of cis- and trans-PPIs were calculated as: ${\mathrm{PPI}}_{\mathrm{cis},\mathrm{theo}}=\frac{N\;x\;(N-1)}{2}$ and PPI_trans,theo_ = N · ∑_*M,M*≠*N*_{*M*} with N is all protein-coding genes in a conserved syntenic block and M is the number of any other protein-coding genes than N in the human genome.

The significance of the CR of each conserved syntenic block, *CR*_*true*_, was determined based on CRs calculated randomly creating new blocks that matched the true blocks in gene count, *CR*_*random*_. Random blocks were created by shuffling all protein-coding genes in the genome while maintaining the same number of interactions as the high confidence PPI set. A total of 10,000 randomizations were performed. P-values for each block were calculated as the number of random blocks with *CR*_*random*_ ≥ *CR*_*true*_ divided by number of random iterations, i.e. 10,000. The p-values were adjusted for multiple testing using Benjamini-Hochberg correction.

### Chromatin loops as determinant for coordinated expression

Data on chromatin loops at 5kb resolution published by Rao *et al*. (2014) [12] was used as a determinant for coordinated expression in the conserved syntenic blocks. The loops were mapped to the positions of the conserved syntenic blocks in the human genome. We applied a strict location and therefore did not allowed loops to exceed neither the start or end positions of syntenic blocks.

### Connectivity-based syntenic blocks

All protein-coding genes from the human genome (assembly GRCh37.p8) were used to determine syntenic blocks based on high confidence PPIs obtained from InWeb_IM (confidence-score ≥ 0.1). All chromosomes were analysed from start to end position for neighbouring genes with interactions. To allow for small rearrangements, a gap size of minimum 1Mb between genes was allowed. This threshold was similarly to the one used when defining orthology-based syntenic blocks. Two genes separated by less than gap size were combined to a block also when not having an interaction. A block was ended if the next neighbouring gene did not have an interaction with any gene in the block and its distance to the nearest interaction partner was more than the allowed gap size.

## Supporting information

S1 Fig Workflow for defining orthology-based pairwise- and conserved syntenic blocks. Syntenic blocks were defined as chromosome regions with conserved order of at least two orthologous protein-coding genes in the five species: human, chimpanzee, mouse, pig and dog. (A) Pairwise syntenic blocks were defined by pairwise comparisons of the order of protein-coding genes being orthologous between two species while always using the human genome as reference. The figure shows pairwise syntenic blocks on human chromosome 1 conserved on mouse chromosome 1, 3 and 17. Zooming in on the blocks between the human and mouse chromosome 1, the orthologous gene pairs were identified first (yellow squares). A block was initiated at human chromosome start (potentially orthologous gene pair 1–101) or when a gene was not part of the previous block (potentially orthologous gene pair 6–106). A new block was initiated either due to a distance between genes that was greater than the maximum gap size of 1Mb in either species (e.g. between gene 1 and 2, or 5 and 6) or if the gene in the compared species was not located next to the previous gene (e.g. being on another chromosome). Oppositely, a block ended at human chromosome end or when the next orthologous gene in the compared species was not located next to the previous gene. Gene pairs were defined as blocks if they had a minimum of two genes, i.e. lonely gene pairs, e.g. 1–101, should be excluded. The initial blocks were subsequently collapsed if they were separated by less than 1Mb. This step allowed for block collapse if the genes included in pairwise syntenic blocks had been rearranged in their close neighborhood. (B) The conserved syntenic blocks defined in the human genome were the common overlap of the pairwise syntenic blocks of all four pairwise comparisons. Exemplified here are all orthologous gene pairs and two pairwise syntenic blocks from the four pairwise comparisons on human chromosome 1. The final conserved syntenic blocks were the common areas in regard to the human genome identical across all five species. If a non-orthologous gene was present within a block, e.g. gene 3, this was included in the block as long as the distances between the two orthologous neighboring genes were less than 1Mb. As previous, this accounted for micro-rearrangements within the region.
(PDF)

S2 Fig **Pairwise syntenic blocks between the human and the four compared mammalian species: (A) chimpanzee, (B) mouse, (C) pig, and (D) dog.** The pairwise syntenic blocks are shown in the human genome. The colors mark the chromosomal location in the compared species genome and grey indicates non-syntenic blocks. The peaks on the chromosomes mark gene density per bin, where each bin is 1/1000 of the chromosome length.
(PDF)

S1 Table Genome positions and gene count of the pairwise syntenic blocks between human and chimpanzee. The blocks are ordered by the location on the human chromosome. There are 362 blocks in total containing 17,173 orthologous protein-coding genes.
(PDF)

S2 Table Genome positions and gene count of the pairwise syntenic blocks between human and mouse. The blocks are ordered by the location on the human genome. There are 581 blocks in total containing 16,231 orthologous protein-coding genes.
(PDF)

S3 Table Genome positions and gene count of the pairwise syntenic blocks between human and pig. The blocks are ordered by the location on the human genome. There are 600 blocks in total containing 14,048 orthologous protein-coding genes.
(PDF)

S4 Table Genome positions and gene count of the pairwise syntenic blocks between human and dog. The blocks are ordered by the location on the human genome. There are 499 blocks in total containing 15,773 orthologous protein-coding genes.
(PDF)

S5 Table The genome positions, gene count and gene product interaction counts of the conserved syntenic blocks between the five mammalian species. The blocks were defined based on the overlap of the four sets of pairwise blocks. There are 829 blocks in total containing 17,555 orthologous protein-coding genes. The blocks are ordered by the location on the human genome. The counts of cis- and trans-PPIs are given for the 192 blocks with minimum one high-confident cis- and one trans-PPI. **“**Obs/Theo (Cis) = NA” indicates that there was one gene in the block after removing tandem duplicates. “Obs/Theo (Trans) = NA” indicate that there were no genes in the block after removing tandem duplicates.
(PDF)

S6 Table Information for the conserved syntenic blocks with minimum one high-confident cis-PPI and one high-confident trans-PPI. In total 192 blocks had at least one high-confident cis- and trans-PPI. Here are information on these blocks in regard to chromosome position, CR, CR difference from the median of the randomization, number of genes in block, gene family information from HGNC [30] and the first level GO terms.
(PDF)
