# Supplementary figures and images for: The impact of the protein interactome on the syntenic structure of mammalian genomes
Source: PLoS One. 2017 Sep 14;12(9):e0179112. doi: 10.1371/journal.pone.0179112 (PMC5598925; doi:10.1371/journal.pone.0179112)

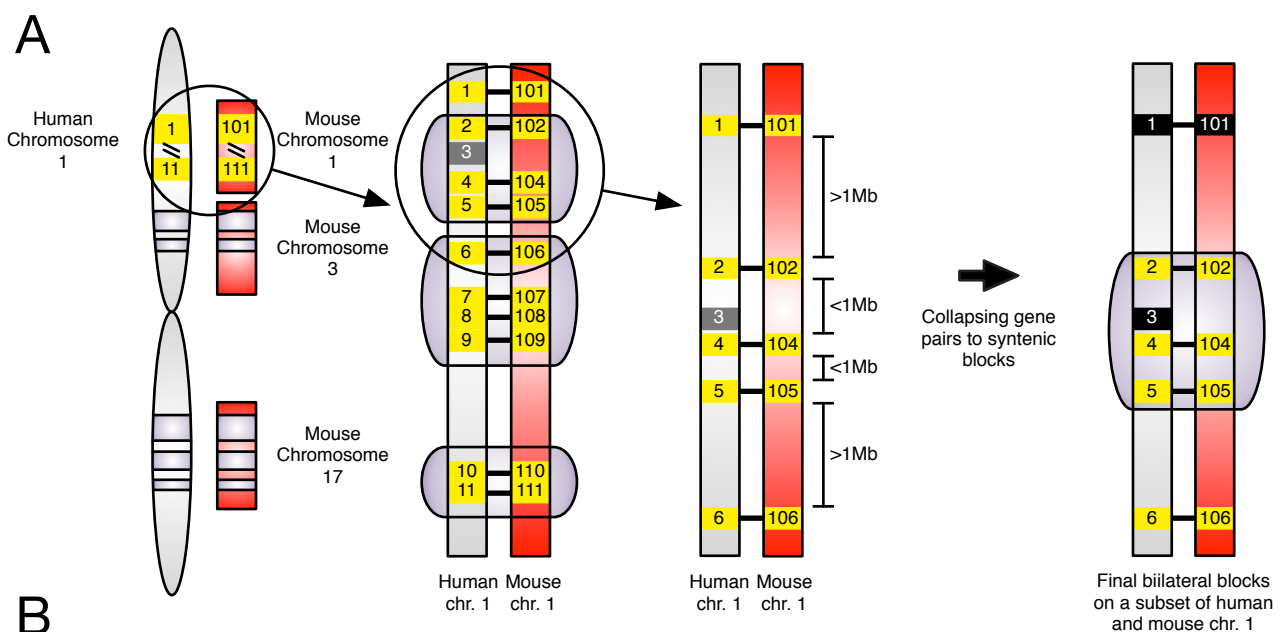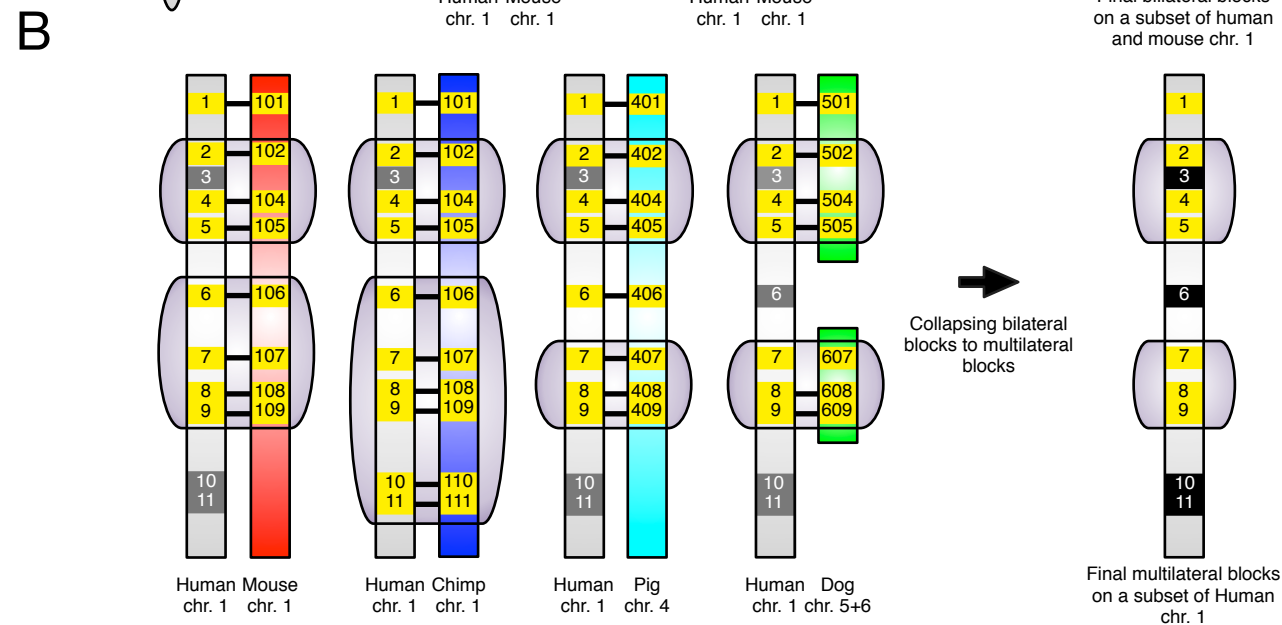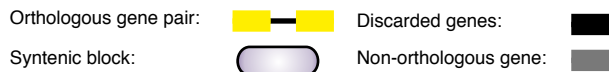

Supplement: S1 Fig — Syntenic blocks were defined as chromosome regions with conserved order of at least two orthologous protein-coding genes in the five species: human, chimpanzee, mouse, pig and dog. (A) Pairwise syntenic blocks were defined by pairwise comparisons of the order of protein-coding genes being orthologous between two species while always using the human genome as reference. The figure shows pairwise syntenic blocks on human chromosome 1 conserved on mouse chromosome 1, 3 and 17. Zooming in on the blocks between the human and mouse chromosome 1, the orthologous gene pairs were identified first (yellow squares). A block was initiated at human chromosome start (potentially orthologous gene pair 1–101) or when a gene was not part of the previous block (potentially orthologous gene pair 6–106). A new block was initiated either due to a distance between genes that was greater than the maximum gap size of 1Mb in either species (e.g. between gene 1 and 2, or 5 and 6) or if the gene in the compared species was not located next to the previous gene (e.g. being on another chromosome). Oppositely, a block ended at human chromosome end or when the next orthologous gene in the compared species was not located next to the previous gene. Gene pairs were defined as blocks if they had a minimum of two genes, i.e. lonely gene pairs, e.g. 1–101, should be excluded. The initial blocks were subsequently collapsed if they were separated by less than 1Mb. This step allowed for block collapse if the genes included in pairwise syntenic blocks had been rearranged in their close neighborhood. (B) The conserved syntenic blocks defined in the human genome were the common overlap of the pairwise syntenic blocks of all four pairwise comparisons. Exemplified here are all orthologous gene pairs and two pairwise syntenic blocks from the four pairwise comparisons on human chromosome 1. The final conserved syntenic blocks were the common areas in regard to the human genome identical across all five species. [file pone.0179112.s001.pdf]
