# Supplementary material for: The impact of the protein interactome on the syntenic structure of mammalian genomes
Source: PLoS One. 2017 Sep 14;12(9):e0179112. doi: 10.1371/journal.pone.0179112 (PMC5598925; doi:10.1371/journal.pone.0179112)

A Pairwise syntenic blocks between human and chimp

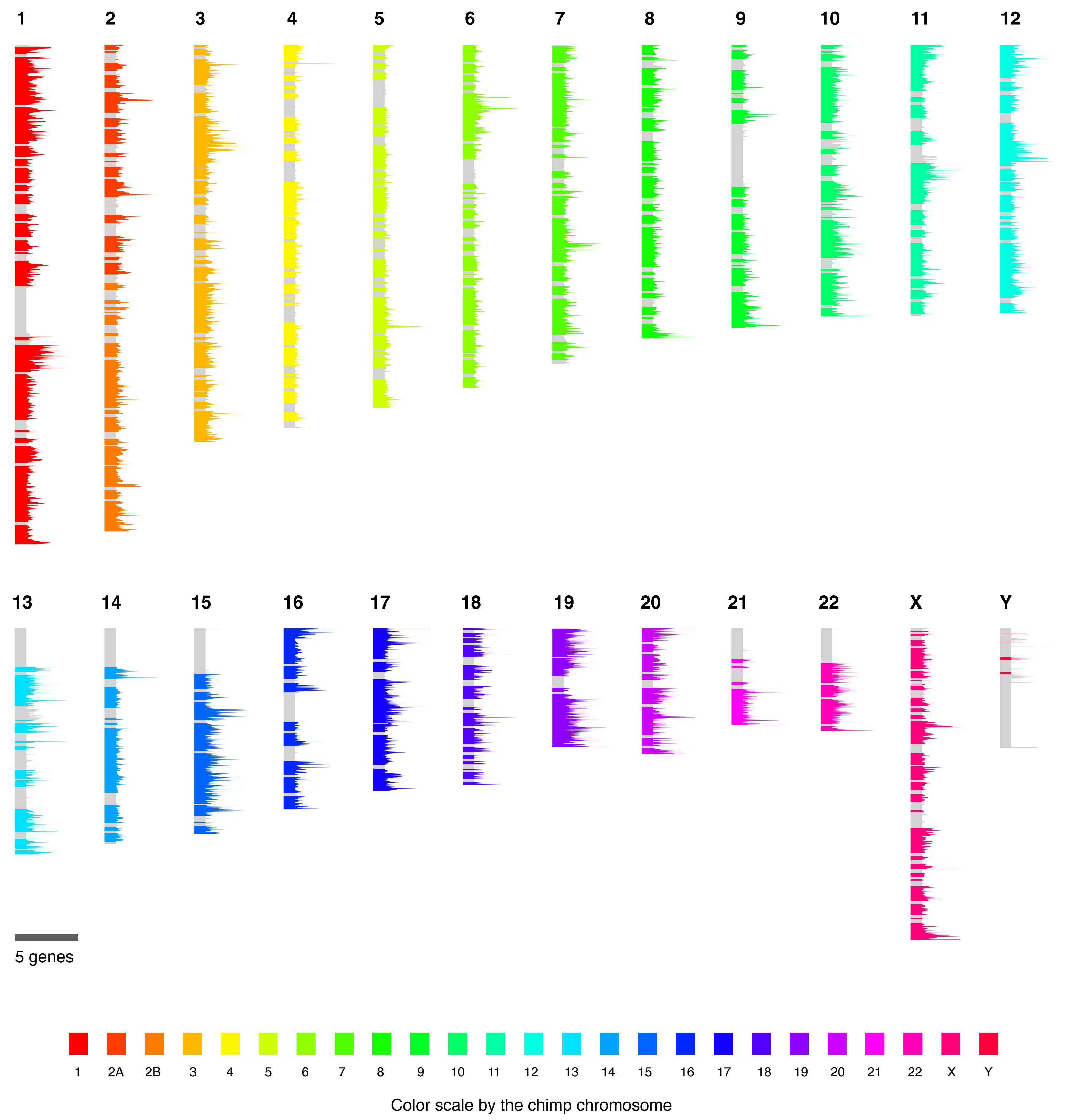

B Pairwise syntenic blocks between human and mouse

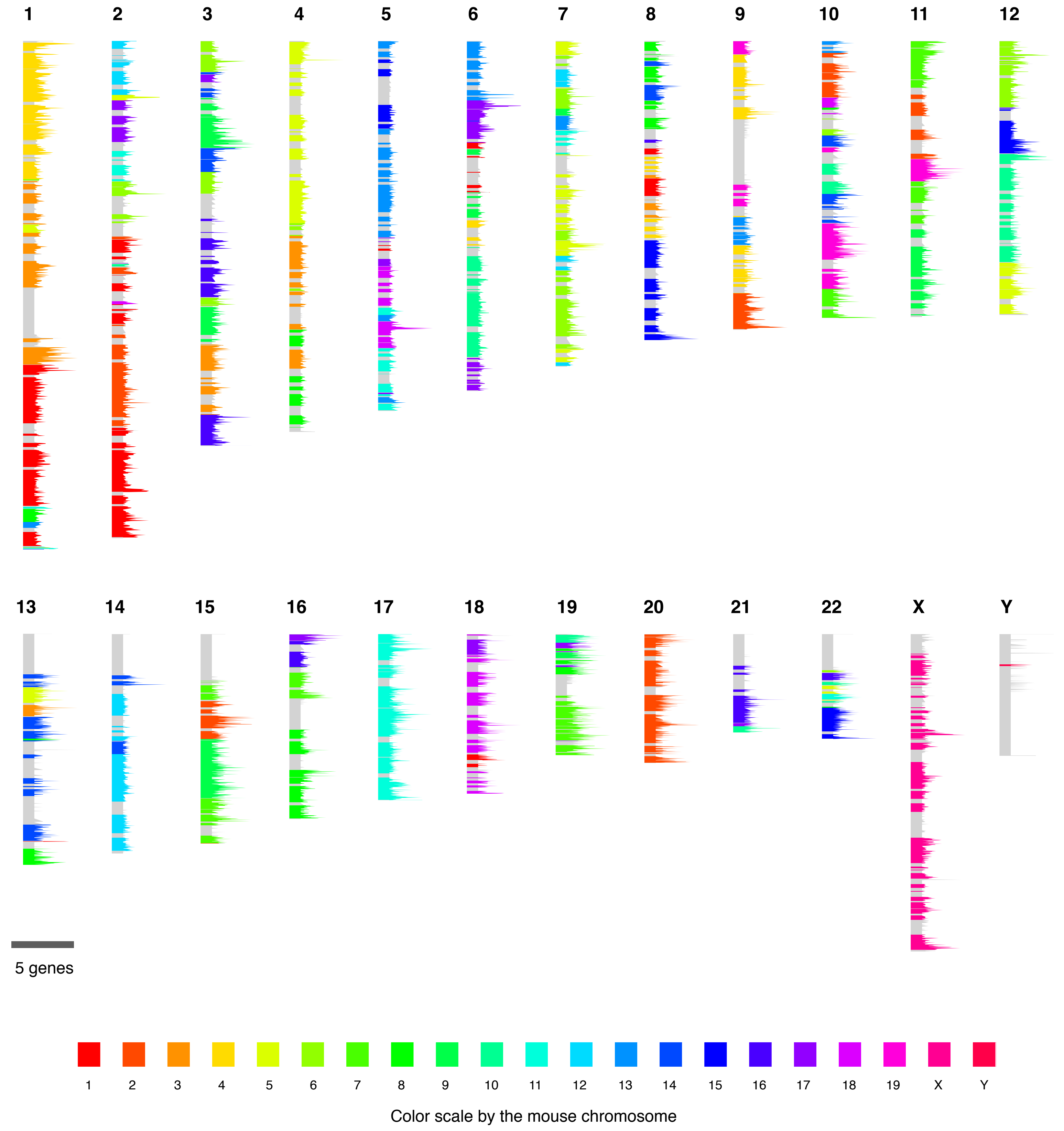

C Pairwise syntenic blocks between human and pig

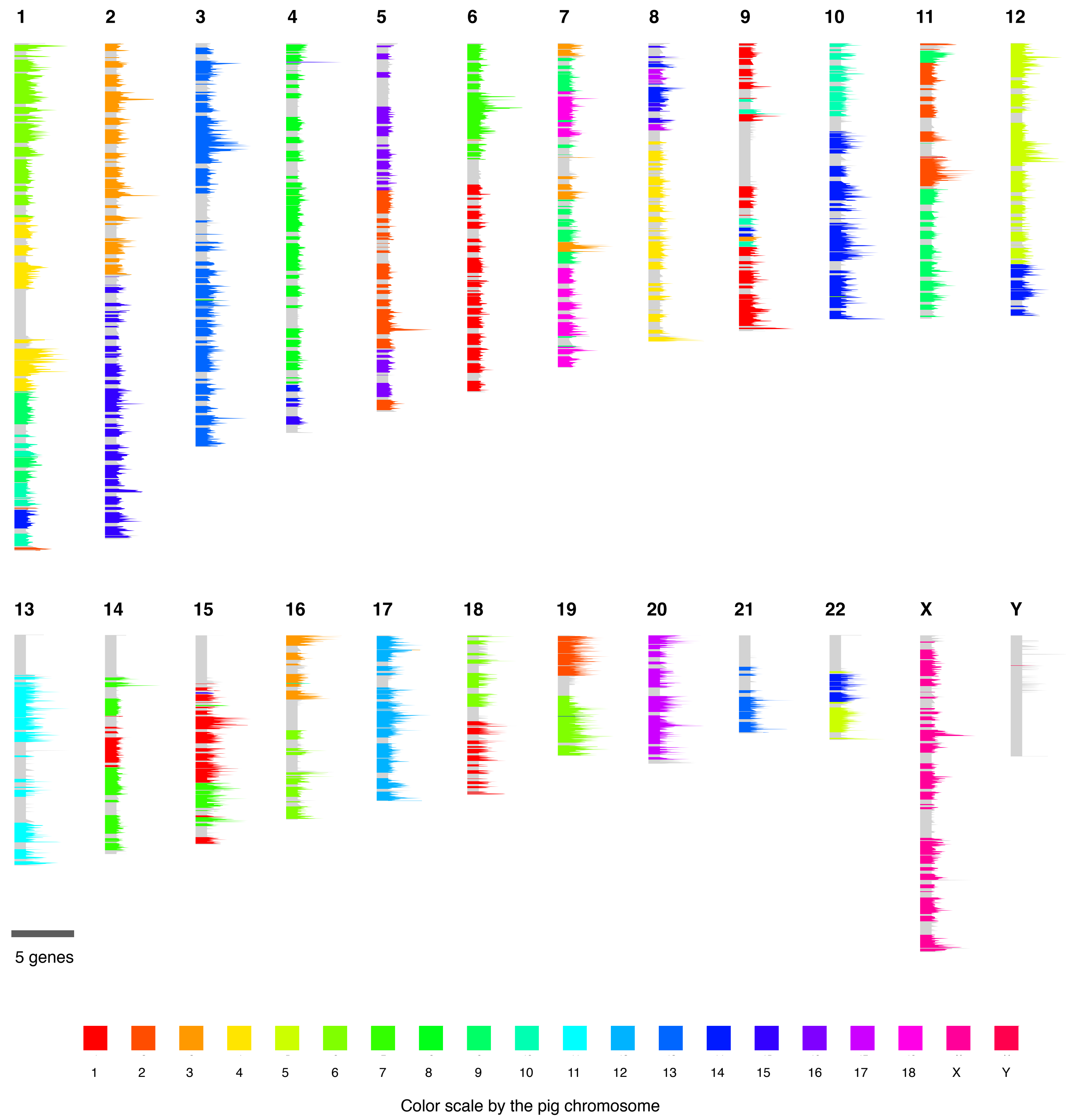

D Pairwise syntenic blocks between human and dog

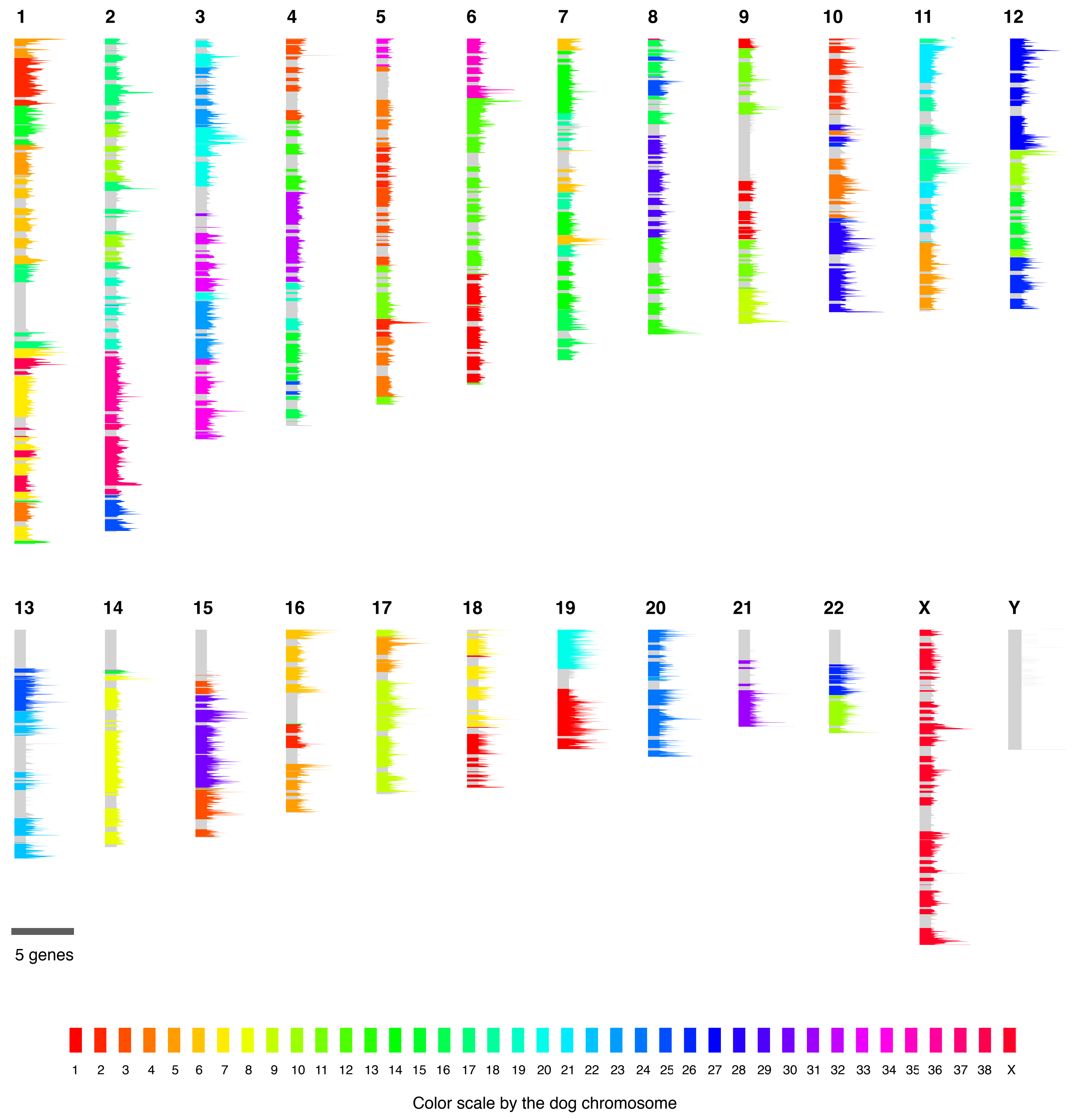

Supplement: S2 Fig — Pairwise syntenic blocks between the human and the four compared mammalian species: (A) chimpanzee, (B) mouse, (C) pig, and (D) dog. The pairwise syntenic blocks are shown in the human genome. The colors mark the chromosomal location in the compared species genome and grey indicates non-syntenic blocks. The peaks on the chromosomes mark gene density per bin, where each bin is 1/1000 of the chromosome length. (PDF) [file pone.0179112.s002.pdf]
