# Supplementary material for: The impact of the protein interactome on the syntenic structure of mammalian genomes
Source: PLoS One. 2017 Sep 14;12(9):e0179112. doi: 10.1371/journal.pone.0179112 (PMC5598925; doi:10.1371/journal.pone.0179112)
Supplement: S1 Table — The blocks are ordered by the location on the human chromosome. There are 362 blocks in total containing 17,173 orthologous protein-coding genes. (PDF) [file pone.0179112.s003.pdf]

Block No	Gene No	Human Chr	Human Start	Human End	Human Length	Chimp Chr	Chimp Start	Chimp End	Chimp Length
1	8	1	860260	991496	131236	7	159377157	159501802	124645
2	48	1	1109264	4843850	3734586	1	1099891	4674734	3574843
3	280	1	6161853	29653325	23491472	1	6092756	29385880	23293124
4	226	1	31184124	49242641	18058517	1	30790713	49138936	18348223
5	59	1	50459990	55681039	5221049	1	50356458	55707345	5350887
6	6	1	56960419	59012406	2051987	1	57012014	57827326	815312
7	7	1	59041099	60539442	1498343	1	59106889	60621699	1514810
8	40	1	61330931	68962904	7631973	1	61631374	69119974	7488600
9	9	1	70034081	72748417	2714336	1	70396406	72917928	2521522
10	28	1	74491699	79472403	4980704	1	74692862	79691104	4998242
11	27	1	84330711	87812788	3482077	1	84603346	88085090	3481744
12	47	1	89149905	95712781	6562876	1	89476108	96025554	6549446
13	23	1	97187221	102462586	5275365	1	97517058	102692033	5174975
14	3	1	103342023	104122156	780133	1	103756509	104679443	922934
15	32	1	107599267	110208118	2608851	1	107927338	110420094	2492756
16	95	1	110230436	120439118	10208682	1	116781176	127164964	10383788
17	23	1	145456236	147381393	1925157	1	111328496	113040126	1711630
18	345	1	149675978	163325554	13649576	1	127872292	141639441	13767149
19	138	1	164524821	186958113	22433292	1	142878119	165614052	22735933
20	10	1	192127587	193223031	1095444	1	170791276	171885799	1094523
21	15	1	196194909	198726545	2531636	1	174928575	177478533	2549958
22	107	1	199996730	208417665	8420935	1	178759166	187307319	8548153
23	164	1	209757062	238129359	28372297	1	188680737	217201455	28520718
24	59	1	239549865	248814185	9264320	1	219223382	228144300	8920918
25	11	10	92828	1779670	1686842	10	98074	1829410	1731336
26	3	10	3109712	3827473	717761	10	3126602	3857488	730886
27	30	10	4828820	8117161	3288341	10	4910451	8164982	3254531
28	115	10	11047259	38691844	27644585	10	11165519	38947999	27782480
29	27	10	42903616	47174093	4270477	10	42244194	46549398	4305204
30	4	10	51187938	51732941	545003	10	45825574	46097081	271507
31	7	10	51944621	54531460	2586839	10	48583733	51193559	2609826
32	23	10	59951278	65384883	5433605	10	56526417	62003336	5476919
33	93	10	67679719	78318978	10639259	10	64329627	75166061	10836434
34	4	10	79550549	79816570	266021	10	76400088	76657383	257295
35	3	10	80828792	81205383	376591	10	77829578	78079379	249801
36	8	10	81315608	82406316	1090708	10	79486503	79980957	494454
37	7	10	85899196	86278273	379077	10	83530758	83914630	383872
38	180	10	87359312	106214848	18855536	10	85020461	103702946	18682485
39	11	10	111624524	112840658	1216134	10	109214577	110450370	1235793
40	97	10	113909624	129924649	16015025	10	111539471	127804261	16264790
41	29	10	131265448	135383462	4118014	10	129158874	133406912	4248038
42	265	11	167784	22851845	22684061	11	232227	22670686	22438459
43	11	11	26210829	28355054	2144225	11	26190284	28215986	2025702
44	43	11	30031288	36694823	6663535	11	29927497	36649075	6721578
45	64	11	43333513	50004071	6670558	11	43411621	49577464	6165843
46	357	11	59244846	79151992	19907146	11	57261237	76881924	19620687
47	8	11	82443053	85338966	2895913	11	80645012	82181317	1536305
48	21	11	85339629	89956532	4616903	11	83552193	87973993	4421800
49	27	11	92085262	96123087	4037825	11	90143545	94179651	4036106
50	100	11	98891683	115375675	16483992	11	97406471	113120946	15714475
51	75	11	116618886	121504387	4885501	11	114647073	119596964	4949891
52	64	11	122526383	126873355	4346972	11	120608275	124992978	4384703
53	32	11	128328656	134281812	5953156	11	126470892	132491454	6020562
54	190	12	175931	16430619	16254688	12	75728	16577698	16501970
55	7	12	18233803	20906320	2672517	12	18395685	21115953	2720268
56	16	12	21200113	22838646	1638533	12	65835344	67734544	1899210
57	44	12	24964295	34182629	9218334	12	54325079	63709777	9384698
58	275	12	38710380	60176395	21466015	12	34942232	51166275	16224043
59	27	12	62102040	68059186	5957146	12	21558062	27585758	6027696
60	31	12	68548548	73059422	4510874	12	67911294	72463555	4552261
61	28	12	74931551	83528649	8597098	12	74411570	83189422	8777852
62	7	12	85253492	86889092	1635600	12	84944324	86163228	1218904
63	8	12	88373816	90103077	1729261	12	88131288	89796196	1664908
64	37	12	91299399	97347129	6047730	12	91117561	97201027	6083466
65	227	12	98909290	126146917	27237627	12	98792446	126532207	27739761
66	28	12	128751948	133812681	5060733	12	129166689	134236139	5069450
67	22	13	19759362	22278637	2519275	13	18705925	21391835	2685910
68	77	13	23755091	39460074	15704983	13	22718688	38549340	15830652
69	3	13	47127303	47471169	343866	13	46241338	46586279	344941
70	39	13	48627459	53626196	4998737	13	47781457	52705376	4923919
71	2	13	57715052	58303445	588393	13	56812067	57401756	589689
72	3	13	60239717	62002220	1762503	13	59353915	61114370	1760455
73	12	13	72012098	76457947	4445849	13	71290430	75811391	4520961
74	13	13	77454312	80915086	3460774	13	76815492	80353568	3538076
75	40	13	92050929	103719196	11668267	13	91571594	103364201	11792607
76	18	13	107142093	112324955	5182862	13	106838757	112073314	5234557
77	18	13	113030633	114898086	1867453	13	113108832	115031492	1922660
78	132	14	19553365	25519503	5966138	14	18442820	23901615	5458795

79	48	14	29235050	39901704	10666654	14	27619066	38432267	10813201
80	8	14	44973545	45722743	749198	14	43396809	44148945	752136
81	2	14	47120222	48144157	1023935	14	45543638	46573901	1030263
82	228	14	50043390	82000205	31956815	14	48479108	80897906	32418798
83	73	14	88304164	97398059	9093895	14	87393433	96518530	9125097
84	17	14	99177950	101201539	2023589	14	98385280	100425460	2040180
85	50	14	102228135	106391825	4163690	14	101460052	105632369	4172317
86	18	15	22833395	30261068	7427673	15	20472928	27418736	6945808
87	22	15	31775329	35838394	4063065	15	28280890	32379549	4098659
88	111	15	36871812	45968512	9096700	15	33416143	42838688	9422545
89	78	15	47476298	61521518	14045220	15	44895102	58008232	13113130
90	209	15	62144588	87572283	25427695	15	59249198	83996448	24747250
91	49	15	88402982	93632433	5229451	15	85205634	90465309	5259675
92	2	15	96869167	97328845	459678	15	93780945	94246089	465144
93	22	15	98462784	102359350	3896566	15	95391096	99375445	3984349
94	101	16	96407	2827298	2730891	16	37424	2847909	2810485
95	2	16	2867164	2888967	21803	9	133315392	133329296	13904
96	110	16	2902728	17564738	14662010	16	2919123	17372962	14453839
97	65	16	18792617	25269252	6476635	16	18650152	25377654	6727502
98	103	16	27214807	31928668	4713861	16	27416956	32124012	4707056
99	28	16	46614466	51185278	4570812	16	45517830	50144173	4626343
100	67	16	52471917	58768261	6296344	16	51445882	57857870	6411988
101	116	16	66400533	73178346	6777813	16	65602000	72504329	6902329
102	46	16	74330673	82203831	7873158	16	73665888	81744926	8079038
103	82	16	83841448	90158480	6317032	16	83410349	89822464	6412115
104	181	17	5810	7923657	7917847	17	89071	8091996	8002925
105	56	17	7942335	15408394	7466059	17	40544884	48098556	7553672
106	45	17	16832849	21477781	4644932	17	34428940	39035334	4606394
107	395	17	25621102	47592379	21971277	17	8127541	29801692	21674151
108	32	17	47676246	51902573	4226327	17	48167287	52466220	4298933
109	124	17	52976748	68176189	15199441	17	53560982	68967853	15406871
110	182	17	70117161	81009686	10892525	17	70943191	82566147	11622956
111	10	18	158383	912173	753790	18	15799453	16565031	765578
112	10	18	2537524	4455335	1917811	18	12753183	14142377	1389194
113	8	18	5145284	7232045	2086761	18	9364441	11481146	2116705
114	32	18	8609443	14543584	5934141	18	1930320	7995430	6065110
115	29	18	18529701	25757410	7227709	18	16598966	23930093	7331127
116	37	18	28569974	35146000	6576026	18	26721789	33375206	6653417
117	3	18	39535171	40857615	1322444	18	37792080	39150850	1358770
118	39	18	42260138	48744674	6484536	18	40593707	47131750	6538043
119	27	18	49866542	58040001	8173459	18	48281561	56466425	8184864
120	19	18	59000815	61672278	2671463	18	57595818	60091570	2495752
121	3	18	63417488	65184217	1766729	18	61876556	63710044	1833488
122	6	18	66340925	67997436	1656511	18	64899462	66566683	1667221
123	2	18	70203915	70535381	331466	18	68798645	69138680	340035
124	15	18	71740588	74980858	3240270	18	70372318	73606408	3234090
125	11	18	76740275	78005429	1265154	18	75312888	76576998	1264110
126	375	19	281043	14640049	14359006	19	219757	14816939	14597182
127	2	19	14640382	14682886	42504	15	39414158	39423564	9406
128	162	19	14693896	23941693	9247797	19	14896729	23765791	8869062
129	9	19	29698173	31840453	2142280	19	34296510	36395837	2099327
130	691	19	32836500	59066486	26229986	19	37480059	63611938	26131879
131	8	2	38814	2335032	2296218	2A	29573	2271864	2242291
132	3	2	3192696	3523507	330811	2A	4746812	5072805	325993
133	3	2	3642426	3836122	193696	2A	3105271	3301296	196025
134	3	2	6980701	7208417	227716	2A	6945207	7133541	188334
135	29	2	8818975	12882860	4063885	2A	8778192	12854621	4076429
136	24	2	14772810	21366144	6593334	2A	14790134	21401769	6611635
137	102	2	23608088	33824449	10216361	2A	23688702	34099841	10411139
138	31	2	36583069	40838193	4255124	2A	36887117	41060022	4172905
139	42	2	42162508	49381676	7219168	2A	42612498	49937427	7324929
140	17	2	53759810	55921045	2161235	2A	54512123	56558173	2046050
141	2	2	58134786	58468507	333721	2A	58909612	59101488	191876
142	30	2	60678302	65659771	4981469	2A	61307684	66335141	5027457
143	92	2	66660584	75938115	9277531	2A	67342830	76710511	9367681
144	4	2	79252812	79386879	134067	2A	80091290	80219828	128538
145	39	2	84650647	89050427	4399780	2A	85589130	89057920	3468790
146	72	2	95537178	103460352	7923174	2A	95048279	103145610	8097331
147	9	2	105654441	107503564	1849123	2A	105389978	107255026	1865048
148	37	2	108602979	114036527	5433548	2A	108250238	113306556	5056318
149	2	2	114462588	114720173	257585	2B	114181843	114437239	255396
150	22	2	118572226	122525429	3953203	2B	118359329	122363411	4004082
151	16	2	127413509	129076151	1662642	2B	127323603	129059383	1735780
152	15	2	130831108	132291239	1460131	2B	131080183	132141651	1061468
153	2	2	133174147	133429152	255005	2B	136329202	136591731	262529
154	17	2	134877554	139537918	4660364	2B	138191246	142787647	4596401
155	4	2	143635067	145282147	1647080	2B	146927352	148567537	1640185
156	24	2	148602086	155714863	7112777	2B	151919172	159072819	7153647
157	117	2	157180944	180871840	23690896	2B	160547365	184506950	23959585
158	11	2	182321929	184026408	1704479	2B	185994990	187721666	1726676

159	32	2	185463093	193060435	7597342	2B	189182761	196853317	7670556
160	17	2	196440701	199437305	2996604	2B	200331872	202840474	2508602
161	66	2	200134223	209719227	9585004	2B	203977788	213381410	9403622
162	80	2	210288782	220506702	10217920	2B	214488291	224725084	10236793
163	17	2	222282747	226518734	4235987	2B	226508896	230794884	4285988
164	126	2	227599757	242743623	15143866	2B	231885579	247143835	15258256
165	92	20	68351	6760910	6692559	20	79635	6662702	6583067
166	13	20	7863628	11907257	4043629	20	7765249	11819829	4054580
167	39	20	12989627	21696620	8706993	20	12984881	21836622	8851741
168	27	20	23016057	25677477	2661420	20	23165430	25867515	2702085
169	120	20	29845467	37668366	7822899	20	27989916	35920188	7930272
170	114	20	39314488	53267710	13953222	20	37615545	51788275	14172730
171	41	20	54572496	58648008	4075512	20	53139735	57315070	4175335
172	58	20	59827559	62926855	3099296	20	58492775	61678038	3185263
173	6	21	15481134	17252377	1771243	21	551011	2332066	1781055
174	6	21	18811208	19858197	1046989	21	3906155	4869207	963052
175	8	21	26957968	28338832	1380864	21	11773827	13146959	1373132
176	181	21	30244513	48085036	17840523	21	15051771	32708415	17656644
177	108	22	17071667	27026636	9954969	22	15479930	25293944	9814014
178	23	22	28202413	31521442	3319029	22	26530920	28714497	2183577
179	45	22	30476163	34318829	3842666	22	28757076	32599385	3842309
180	164	22	35462129	47882860	12420731	22	33754948	46323828	12568880
181	28	22	48885272	51183762	2298490	22	47325660	49676597	2350937
182	2	3	238279	1445901	1207622	3	250450	1448501	1198051
183	11	3	2140497	5261642	3121145	3	2632316	5303322	2671006
184	84	3	6811688	20227784	13416096	3	6989216	20504163	13514947
185	37	3	23847394	33911194	10063800	3	24205616	34424032	10218416
186	275	3	35680437	61237133	25556696	3	36205754	60917577	24711823
187	16	3	61547243	67061634	5514391	3	62489267	68227370	5738103
188	18	3	68053359	74570291	6516932	3	69238726	75870095	6631369
189	2	3	75955846	79816965	3861119	3	78884541	80457553	1573012
190	10	3	85008132	89531284	4523152	3	87293029	91112483	3819454
191	5	3	93591881	93847389	255508	3	96874911	97133839	258928
192	35	3	96533425	102198685	5665260	3	99880733	105668656	5787923
193	2	3	105085753	105588396	502643	3	108597194	109098787	501593
194	15	3	107096188	109056419	1960231	3	110667270	112633733	1966463
195	38	3	110607231	117716095	7108864	3	114211627	119442860	5231233
196	182	3	118619404	143767561	25148157	3	122277916	147583664	25305748
197	8	3	145782358	147228080	1445722	3	149678116	151049285	1371169
198	72	3	148415571	161221730	12806159	3	152339819	165288214	12948395
199	3	3	164696686	165555260	858574	3	168784484	169641992	857508
200	34	3	166958075	172859058	5900983	3	171057395	176992395	5935000
201	2	3	173114074	175523428	2409354	3	178156566	179727674	1571108
202	17	3	177990720	181432221	3441501	3	182775544	185705910	2930366
203	117	3	182511288	197896723	15385435	3	186798181	202253044	15454863
204	76	4	53179	8308838	8255659	4	62277	8480379	8418102
205	8	4	9385743	11431389	2045646	4	9115356	11135845	2020489
206	3	4	13362978	13629347	266369	4	13138198	13392970	254772
207	19	4	15004298	18023499	3019201	4	14824846	17866698	3041852
208	4	4	20254883	22517677	2262794	4	20323276	22415896	2092620
209	16	4	23756664	27027003	3270339	4	23720255	26998925	3278670
210	37	4	36067620	43032675	6965055	4	36032092	43111458	7079366
211	4	4	44175926	44728612	552686	4	44553770	44831977	278207
212	20	4	46037786	49064098	3026312	4	82970530	86044903	3074373
213	32	4	52709166	57976551	5267385	4	72852544	78192869	5340325
214	126	4	68337521	85887544	17550023	4	44993594	62444295	17450701
215	39	4	86396267	96762625	10366358	4	87833294	98238260	10404966
216	61	4	98105244	111563279	13458035	4	99992796	113242576	13249780
217	11	4	113066553	116035032	2968479	4	114773398	117823753	3050355
218	29	4	118954773	124324910	5370137	4	120704838	125861696	5156858
219	2	4	125585207	126414087	828880	4	127141015	127978528	837513
220	11	4	128544426	130034487	1490061	4	130136281	131634038	1497757
221	44	4	138440072	149365850	10925778	4	140205477	151451492	11246015
222	45	4	150999426	160281321	9281895	4	153087413	162464849	9377436
223	6	4	164031225	165305202	1273977	4	166232683	166971137	738454
224	19	4	165875598	171012850	5137252	4	168030416	173293945	5263529
225	21	4	172733405	178911904	6178499	4	175034348	181255201	6220853
226	34	4	183065140	187647876	4582736	4	185651850	190057760	4405910
227	2	4	188916925	189030757	113832	4	191355813	191459858	104045
228	26	5	140373	3601517	3461144	5	273441	3619296	3345855
229	7	5	5140443	6757161	1616718	5	5211498	6841224	1629726
230	3	5	7830491	7906138	75647	5	7903517	7974422	70905
231	10	5	9035138	11904155	2869017	5	9126679	12038840	2912161
232	11	5	13690440	17276943	3586503	5	13831535	17465851	3634316
233	41	5	31193857	39462402	8268545	5	75710778	83978168	8267390
234	26	5	40679600	45696253	5016653	5	69316816	74424520	5107704
235	41	5	49692026	59817947	10125921	5	55996264	65164834	9168570
236	10	5	59892739	61924409	2031670	5	52679660	54731695	2052035
237	17	5	63256183	66492627	3236444	5	48079545	51339386	3259841
238	15	5	67485704	70320941	2835237	5	45501223	47067588	1566365

239	70	5	70751442	83680611	12929169	5	31008301	44101618	13093317
240	6	5	85913721	88199922	2286201	5	26558087	28863736	2305649
241	6	5	89688078	90679176	991098	5	24018657	25029157	1010500
242	16	5	92919043	95769847	2850804	5	18899530	21790509	2890979
243	8	5	95865525	96519354	653829	5	96952505	97443718	491213
244	2	5	98104354	98262240	157886	5	99050175	99208686	158511
245	2	5	99871009	100238970	367961	5	100831743	101193111	361368
246	7	5	101569690	102898494	1328804	5	102576490	103919409	1342919
247	33	5	106712590	115910630	9198040	5	107791448	117079960	9288512
248	5	5	118173017	118971517	798500	5	119380595	120194843	814248
249	10	5	121187650	122952739	1765089	5	122444410	124218878	1774468
250	164	5	125695824	143856944	18161120	5	127045101	145238134	18193033
251	66	5	144851362	151812929	6961567	5	146576048	153271131	6695083
252	11	5	152869175	154348971	1479796	5	154321508	155828329	1506821
253	33	5	155297354	161326975	6029621	5	157192064	162846071	5654007
254	4	5	162864575	162946342	81767	5	164406429	164487952	81523
255	113	5	166711804	180688119	13976315	5	168282885	182422803	14139918
256	45	6	292097	8435794	8143697	6	246256	8499708	8253452
257	28	6	9596343	14137149	4540806	6	9749434	14245014	4495580
258	15	6	15246527	18469105	3222578	6	15408084	18657493	3249409
259	7	6	19837617	22571892	2734275	6	20024403	22792730	2768327
260	398	6	24126350	48036425	23910075	6	24362003	48750040	24388037
261	49	6	49398073	57087078	7689005	6	50128123	57959932	7831809
262	9	6	69345259	72011973	2666714	6	68926425	71603300	2676875
263	9	6	73331520	74538040	1206520	6	73315555	74138398	822843
264	5	6	75962640	76782395	819755	6	75599947	76441036	841089
265	8	6	79577189	81055987	1478798	6	79307881	80864638	1556757
266	18	6	82201156	86445786	4244630	6	82275897	86323234	4047337
267	27	6	87647024	91296764	3649740	6	87527878	91240761	3712883
268	8	6	96025419	97731093	1705674	6	96462993	98171628	1708635
269	12	6	99282580	102517958	3235378	6	99729656	103012158	3282502
270	50	6	105175968	112672498	7496530	6	105722226	113471603	7749377
271	3	6	114178541	114664209	485668	6	114953404	115160107	206703
272	29	6	116262693	119670926	3408233	6	117074216	120507902	3433686
273	80	6	121400640	139695757	18295117	6	122266239	140759967	18493728
274	55	6	142379467	153452384	11072917	6	143503529	154740126	11236597
275	7	6	154331631	155777037	1445406	6	155759174	157196804	1437630
276	33	6	157099063	163999628	6900565	6	158519793	165392420	6872627
277	29	6	165693153	170893780	5200627	6	167123530	172559299	5435769
278	56	7	855528	8792593	7937065	7	191357	7357924	7166567
279	7	7	10971578	12730559	1758981	7	9534895	11294668	1759773
280	119	7	13930853	39532694	25601841	7	12486844	38034188	25547344
281	5	7	39605975	40900362	1294387	7	41687420	42618159	930739
282	11	7	41724712	43993166	2268454	7	38265999	40497414	2231415
283	35	7	44084232	48687092	4602860	7	42705519	47290769	4585250
284	8	7	49813257	51384515	1571258	7	48421300	50004215	1582915
285	14	7	54610018	56174269	1564251	7	53185269	54764703	1579434
286	14	7	62809239	66460635	3651396	7	61985597	65499935	3514338
287	2	7	69063905	71178585	2114680	7	69220179	70236023	1015844
288	3	7	72349936	72476445	126509	7	70766460	70894919	128459
289	28	7	72716514	74490064	1773550	7	72127882	73813767	1685885
290	34	7	75162621	84816171	9653550	7	75606451	85625647	10019196
291	154	7	86273230	102312088	16038858	7	87157619	103181323	16023704
292	39	7	102389418	108524644	6135226	7	104220670	110377005	6156335
293	29	7	110303110	117882785	7579675	7	112162637	119737312	7574675
294	21	7	120427376	124570037	4142661	7	122318388	126459993	4141605
295	144	7	126986844	144533488	17546644	7	128908796	146104718	17195922
296	53	7	148287657	152552463	4264806	7	149885738	154017618	4131880
297	19	7	153584182	157062066	3477884	7	155487207	158620013	3132806
298	11	8	182137	4852494	4670357	8	137027	3237108	3100081
299	13	8	6264113	7740186	1476073	8	6393233	7530572	1137339
300	33	8	11831446	20161474	8330028	8	8240093	16390649	8150556
301	84	8	21547915	31031285	9483370	8	17868719	27592647	9723928
302	6	8	31496902	33457624	1960722	8	28980999	30050530	1069531
303	54	8	36641842	43057998	6416156	8	33279864	39705681	6425817
304	36	8	48173167	57906403	9733236	8	44903260	54758614	9855354
305	6	8	58907068	60031767	1124699	8	55738119	56849269	1111150
306	8	8	61099906	63998612	2898706	8	57922079	60904778	2982699
307	47	8	65500320	76479078	10978758	8	62412695	73559020	11146325
308	2	8	77593454	77913280	319826	8	74685324	75013400	328076
309	20	8	79428374	82755101	3326727	8	76554775	79920389	3365614
310	19	8	85095022	89340254	4245232	8	82813504	86500888	3687384
311	93	8	90769975	110988076	20218101	8	87948698	108417970	20469272
312	2	8	113235157	114629187	1394030	8	110694677	112138010	1443333
313	23	8	116420724	122653630	6232906	8	113947754	120290673	6342919
314	23	8	123793633	126450647	2657014	8	121447171	124121162	2673991
315	14	8	130760442	134584183	3823741	8	128411889	132181449	3769560
316	2	8	135490031	136668965	1178934	8	133211720	134384405	1172685
317	88	8	139142266	146281416	7139150	8	136854550	143953294	7098744
318	5	9	214854	1057552	842698	9	259145	1061932	802787

319	29	9	2621834	7175648	4553814	9	2631960	7247275	4615315
320	45	9	12685439	22452472	9767033	9	12924022	22769468	9845446
321	2	9	23690102	24545944	855842	9	24036234	24902386	866152
322	10	9	26840683	28670283	1829600	9	27224515	28324241	1099726
323	95	9	32384618	39288456	6903838	9	32764920	39516477	6751557
324	21	9	70971815	75785309	4813494	9	66901298	71725774	4824476
325	16	9	77112281	80945009	3832728	9	73072036	76966697	3894661
326	34	9	84198598	92221470	8022872	9	80222605	88381328	8158723
327	82	9	93372114	104500862	11128748	9	89421078	100575100	11154022
328	15	9	106856541	108538893	1682352	9	102948312	104662280	1713968
329	3	9	109625378	110252763	627385	9	105755649	106382686	627037
330	61	9	111616871	120177348	8560477	9	107773966	116386969	8613003
331	254	9	123151147	141019076	17867929	9	119415173	137724693	18309520
332	2	X	1522032	1656000	133968	X	1510123	1556183	46060
333	4	X	2670091	3761934	1091843	X	2664885	3757708	1092823
334	5	X	5758678	9132680	3374002	X	5720010	8919922	3199912
335	57	X	9983602	20159962	10176360	X	9978822	20334963	10356141
336	16	X	21392536	25015103	3622567	X	21582757	25217782	3635025
337	2	X	26156460	26213763	57303	X	26377019	26421368	44349
338	3	X	27608499	27999566	391067	X	27979573	28191472	211899
339	8	X	28605516	31090170	2484654	X	30117029	31285760	1168731
340	17	X	34645181	38665790	4020609	X	34941219	39170333	4229114
341	8	X	39909068	41782716	1873648	X	40436948	42259306	1822358
342	8	X	43515467	45060146	1544679	X	44101789	45668961	1567172
343	96	X	46306292	57937067	11630775	X	46964202	58691930	11727728
344	15	X	62567107	68061990	5494883	X	62886871	68574824	5687953
345	43	X	68835911	75651744	6815833	X	69674773	76261393	6586620
346	16	X	76709648	80554046	3844398	X	77290773	81094452	3803679
347	11	X	82763269	86925050	4161781	X	83373977	87670978	4297001
348	2	X	90689594	91878229	1188635	X	91435803	92671469	1235666
349	94	X	99546642	112084043	12537401	X	100655278	113361667	12706389
350	9	X	113818551	115594164	1775613	X	115166582	116934811	1768229
351	25	X	117629861	120095337	2465476	X	119012531	121485782	2473251
352	6	X	122318006	124097666	1779660	X	123690986	125521458	1830472
353	3	X	125298337	125955769	657432	X	126760003	127416652	656649
354	47	X	128673826	136113833	7440007	X	130124965	137728347	7603382
355	13	X	137713735	142968355	5254620	X	139337416	144653680	5316264
356	2	X	144328348	144911368	583020	X	146015773	146591079	575306
357	83	X	146993469	155251689	8258220	X	148650537	156837705	8187168
358	3	Y	2654896	2850547	195651	Y	26002620	26203382	200762
359	2	Y	6733959	6959977	226018	Y	25417875	25815068	397193
360	2	Y	9236076	9344176	108100	Y	10551240	10865897	314657
361	4	Y	14475147	15817904	1342757	Y	19893476	21263627	1370151
362	3	Y	21865751	22942918	1077167	Y	17324247	18113958	789711
