# Supplementary material for: The impact of the protein interactome on the syntenic structure of mammalian genomes
Source: PLoS One. 2017 Sep 14;12(9):e0179112. doi: 10.1371/journal.pone.0179112 (PMC5598925; doi:10.1371/journal.pone.0179112)
Supplement: S2 Table — The blocks are ordered by the location on the human genome. There are 581 blocks in total containing 16,231 orthologous protein-coding genes. (PDF) [file pone.0179112.s004.pdf]

Block No	Gene No	Human Chr	Human Start	Human End	Human Length	Mouse Chr	Mouse Start	Mouse End	Mouse Length
1	67	1	860260	4843850	3983590	4	153373221	156255338	2882117
2	73	1	5922871	12092102	6169231	4	147860778	152563183	4702405
3	8	1	12123434	13498260	1374826	4	144391674	145315164	923490
4	194	1	13801445	29653325	15851880	4	131768457	143371032	11602575
5	118	1	31184124	40563375	9379251	4	122836242	130955475	8119233
6	106	1	40627045	48462567	7835522	4	114406724	121215084	6808360
7	2	1	48688357	48937845	249488	4	111719984	111902918	182934
8	57	1	50513686	55681039	5167353	4	106316213	110351909	4035696
9	5	1	56960419	57431813	471394	4	104766317	105232764	466447
10	36	1	59120411	67600639	8480228	4	94942040	103290863	8348823
11	5	1	67632083	68299150	667067	6	66896397	67491855	595458
12	3	1	68564142	68962904	398762	3	159495433	159935833	440400
13	8	1	70034081	72748417	2714336	3	156561794	158562221	2000427
14	27	1	74491699	79472403	4980704	3	151437887	155194278	3756391
15	27	1	84330711	87812788	3482077	3	144188530	146984009	2795479
16	6	1	89149905	89738544	588639	3	142496934	142882004	385070
17	25	1	89829617	93828149	3998532	5	105014153	108280526	3266373
18	13	1	93913658	95712781	1799123	3	121155400	122619667	1464267
19	24	1	99127236	104239302	5112066	3	113555710	117868936	4313226
20	119	1	107599267	120612240	13012973	3	98013538	110250998	12237460
21	23	1	145413095	147381393	1968298	3	96525185	97673812	1148627
22	86	1	149754245	153283194	3528949	3	92014583	96279001	4264418
23	114	1	153302596	158154686	4852090	3	86995834	90741517	3745683
24	92	1	158516918	163325554	4808636	1	169497934	174449602	4951668
25	139	1	164524821	186958113	22433292	1	149829618	168432270	18602652
26	9	1	192127587	193223031	1095444	1	143598797	144775435	1176638
27	11	1	196621008	198726545	2105537	1	138062861	139560258	1497397
28	101	1	199996730	207534311	7537581	1	130439027	136960427	6521400
29	5	1	207627575	208417665	790090	1	194619829	195176715	556886
30	92	1	209757062	227506175	17749113	1	179960472	193370297	13409825
31	18	1	227916240	228700004	783764	11	58917908	59458567	540659
32	37	1	228870824	235324772	6453948	8	123653929	126971079	3317150
33	15	1	235294949	238129359	2834410	13	11553103	14199603	2646500
34	27	1	240177648	247095280	6917632	1	174501825	179803680	5301855
35	3	1	247460714	247615308	154594	11	59485520	59571813	86293
36	3	1	247835320	247887345	52025	7	86306185	86364335	58150
37	5	1	248004199	248525929	521730	11	58625073	58787269	162196
38	3	1	248550910	248617130	66220	14	14175095	14408198	233103
39	4	1	248902716	249153343	250627	11	58307069	58390728	83659
40	7	10	225953	1779670	1553717	13	8202866	9765321	1562455
41	3	10	3109712	3827473	717761	13	5861489	6648777	787288
42	10	10	4828820	5884095	1055275	13	3538075	4609174	1071099
43	14	10	5903689	8117161	2213472	2	9857078	11790325	1933247
44	30	10	11047259	15413061	4365802	2	3114224	7081302	3967078
45	16	10	15555948	18970568	3414620	2	12106632	15082456	2975824
46	26	10	20105168	27531059	7425891	2	16356304	23199260	6842956
47	16	10	27793197	32667726	4874529	18	3507957	7973547	4465590
48	3	10	32735068	33625190	890122	8	128359073	129183732	824659
49	2	10	35297479	35501886	204407	18	3266048	3436377	170329
50	3	10	35535953	35930362	394409	18	9212856	9450150	237294
51	2	10	38091751	38265561	173810	6	118427319	118479320	52001
52	17	10	43278249	46168228	2889979	6	116264222	118419417	2155195
53	27	10	46310876	51732941	5422065	14	32142026	34355433	2213407
54	6	10	52065360	54531460	2466100	19	30232928	32389714	2156786
55	22	10	59951278	65384883	5433605	10	67009189	71433327	4424138
56	59	10	68685764	74856732	6170968	10	59323296	64090277	4766981
57	34	10	74870217	79816570	4946353	14	20294690	24496146	4201456
58	3	10	80828792	81205383	376591	14	25459185	25768856	309671
59	2	10	81315608	81742370	426762	14	41131782	41185198	53416
60	2	10	81891477	81965433	73956	14	25842183	25902882	60699
61	6	10	82031576	82406316	374740	14	40813789	41124412	310623
62	7	10	85899196	86278273	379077	14	36874936	37135322	260386
63	8	10	87359312	88951225	1591913	14	34237041	35583379	1346338
64	172	10	89264632	107024993	17760361	19	32485769	48805505	16319736
65	10	10	111624524	112840658	1216134	19	52991183	54048982	1057799
66	44	10	113909624	121215131	7305507	19	55069734	61092553	6022819
67	53	10	121259340	129924649	8665309	7	128373621	135716379	7342758
68	30	10	131265448	135382916	4117468	7	136894611	140787854	3893243
69	74	11	167784	3253616	3085832	7	140845565	143784500	2938935
70	137	11	3659733	17371521	13711788	7	102096879	116540584	14443705
71	19	11	17407406	18814268	1406862	7	46098725	47133684	1034959
72	15	11	19138646	22881972	3743326	7	48789003	52015716	3226713
73	11	11	26210829	28355054	2144225	2	109092297	110950923	1858626
74	44	11	30031288	36694823	6663535	2	101560781	107298502	5737721
75	60	11	43333513	48511332	5177819	2	89835023	94438136	4603113
76	53	11	55135360	57587018	2451658	2	86977829	89364865	2387036

77	264	11	57885972	68708070	10822098	19	3260924	13815658	10554734
78	15	11	68747490	71239227	2491737	7	143795584	145324059	1528475
79	74	11	71639747	79151992	7512245	7	96171244	102065469	5894225
80	6	11	82534544	82997450	462906	7	92561149	92934573	373424
81	19	11	85339629	89322779	3983150	7	87246096	90476001	3229905
82	2	11	89864683	89956532	91849	9	18292267	18402995	110728
83	28	11	92085262	96240738	4155476	9	13242790	16378231	3135441
84	31	11	100558384	107436472	6878088	9	3335478	9239013	5903535
85	58	11	107461817	115375675	7913858	9	47530173	53975301	6445128
86	72	11	116618886	121504387	4885501	9	41964720	46298783	4334063
87	44	11	122526383	125551018	3024635	9	36693220	41161697	4468477
88	16	11	125616188	126873355	1257167	9	34486126	35690318	1204192
89	15	11	128328656	130786404	2457748	9	30427329	32757820	2330491
90	10	11	133710526	134281812	571286	9	26733728	27401710	667982
91	17	12	175931	2802108	2626177	6	118592319	121473678	2881359
92	69	12	2904119	7371170	4467051	6	124396816	128438677	4041861
93	18	12	7801996	9268825	1466829	6	122577792	123289870	712078
94	14	12	9747147	10562356	815209	6	129045901	129678973	633072
95	5	12	10758612	10978957	220345	6	131284388	131687495	403107
96	4	12	11060525	11339543	279018	6	132762131	132957919	195788
97	38	12	11802788	16763528	4960740	6	134035700	138581968	4546268
98	60	12	18233803	32536567	14302764	6	139493182	149563329	10070147
99	4	12	32552463	33049774	497311	16	16213318	16600549	387231
100	2	12	33527173	34182629	655456	15	89782393	90230554	448161
101	178	12	39040624	54982443	15941819	15	90487482	103537992	13050510
102	102	12	55341802	60176395	4834593	10	125226464	130362642	5136178
103	55	12	62102040	73059422	10957382	10	114398823	123741204	9342381
104	13	12	74931551	77459360	2527809	10	110745439	112929026	2183587
105	13	12	79257773	83528649	4270876	10	105187663	109010982	3823319
106	6	12	85253492	86889092	1635600	10	101681487	103419378	1737891
107	9	12	88373816	90103077	1729261	10	98915152	100618391	1703239
108	28	12	91299399	97347129	6047730	10	92684746	97694926	5010180
109	4	12	98909290	99129204	219914	10	90989311	91171582	182271
110	52	12	100422233	108155049	7732816	10	82619851	89819869	7200018
111	26	12	108523248	110477568	1954320	5	113490447	114806200	1315753
112	47	12	110562140	115121969	4559829	5	119670669	122614518	2943849
113	39	12	116395711	121454305	5058594	5	114942158	118765435	3823277
114	59	12	121570622	126146917	4576295	5	122643911	125792583	3148672
115	11	12	128751948	132336328	3584380	5	127241808	129608875	2367067
116	15	12	132379196	133532892	1153696	5	110110092	110810097	700005
117	18	13	20207788	22278637	2070849	14	56668248	58112337	1444089
118	6	13	23755091	24896096	1141005	14	60634001	61258490	624489
119	4	13	24995064	25497018	501954	14	56365068	56659794	294726
120	5	13	25735822	26625198	889376	14	59625281	60381003	755722
121	39	13	26706253	34540695	7834442	5	146209192	151651242	5442050
122	23	13	35516424	41240734	5724310	3	52268337	56183701	3915364
123	36	13	41506056	47471169	5965113	14	74640840	79582476	4941636
124	7	13	48627459	49783888	1156429	14	72537946	73518545	980599
125	5	13	49822047	50159719	337672	14	59201209	59597836	396627
126	12	13	50202435	52334135	2131700	14	61309753	62956886	1647133
127	8	13	52436117	53050485	614368	8	21969775	22261334	291559
128	4	13	53226844	53626196	399352	14	79587691	80021930	434239
129	3	13	60239717	62002220	1762503	14	86655367	88471396	1816029
130	7	13	72012098	74708394	2696296	14	97786853	100149764	2362911
131	4	13	75858808	76434004	575196	14	101442360	101934710	492350
132	12	13	77522632	80915086	3392454	14	103046977	105896819	2849842
133	30	13	95091741	103054124	7962383	14	118012792	124677127	6664335
134	7	13	103249353	103528345	278992	1	43934002	44181260	247258
135	40	13	107142093	115092796	7950703	8	8617434	13881639	5264205
136	48	14	20215587	22134238	1918651	14	49894260	52451402	2557142
137	71	14	23033805	25103473	2069668	14	54235479	56159579	1924100
138	45	14	29235050	39901704	10666654	12	49382693	59219725	9837032
139	8	14	44973545	45722743	749198	12	64471333	65172604	701271
140	2	14	47120222	48144157	1023935	12	66283381	67222549	939168
141	23	14	50043390	52197445	2154055	12	69157722	70902232	1744510
142	3	14	52292913	52535712	242799	14	19751257	19977627	226370
143	32	14	52734431	58764857	6030426	14	44851235	49783383	4932148
144	164	14	58666798	82000205	23333407	12	70937857	91849157	20911300
145	67	14	88304164	97398059	9093895	12	98202304	106077410	7875106
146	71	14	99635624	105996539	6360915	12	107910403	113189520	5279117
147	2	15	22368478	22383507	15029	14	50281228	50320868	39640
148	4	15	22833395	23100005	266610	7	55794148	56019954	225806
149	3	15	23810454	23932450	121996	7	62348277	62420139	71862
150	9	15	25068794	28567298	3498504	7	56050155	60140219	4090064
151	9	15	29129629	32162992	3033363	7	63444751	65371239	1926488
152	19	15	32907345	35838394	2931049	2	112239468	114654964	2415496
153	6	15	36871812	38857776	1985964	2	115581716	117343001	1761285

154	105	15	39873280	45968512	6095232	2	118111876	122809569	4697693
155	24	15	47476298	51298097	3821799	2	124089969	127067909	2977940
156	4	15	51348795	51915030	566235	9	54025606	54501760	476154
157	201	15	51973550	79012628	27039078	9	54538984	75683996	21145012
158	8	15	79051545	80216096	1164551	9	89199379	90208071	1008692
159	35	15	80351910	85682376	5330466	7	80860920	84679361	3818441
160	3	15	85923802	87572283	1648481	7	75455534	76766608	1311074
161	42	15	88402982	91565833	3162851	7	78192362	80803331	2610969
162	5	15	92396925	93632433	1235508	7	73375520	74554780	1179260
163	19	15	98462784	102264807	3802023	7	65644898	68749241	3104343
164	2	15	102345902	102359350	13448	2	111983134	112009210	26076
165	7	16	103010	231180	128170	11	32205415	32300873	95458
166	122	16	238968	3199964	2960996	17	23551073	26285504	2734431
167	39	16	3254247	5116111	1861864	16	3592398	5222299	1629901
168	46	16	8619502	16236931	7617429	16	8409276	14475737	6066461
169	2	16	16242785	16388668	145883	7	45976380	46084212	107832
170	65	16	18792617	26149009	7356392	7	118104378	124398989	6294611
171	98	16	27214807	31520630	4305823	7	125444676	128298170	2853494
172	25	16	46690054	51185278	4495224	8	85260397	89044162	3783765
173	60	16	52471917	58768261	6296344	8	90247040	95888547	5641507
174	90	16	66400533	69975644	3575111	8	104101625	107558594	3456969
175	29	16	70147529	73093597	2946068	8	108714644	111137074	2422430
176	42	16	74442529	82203831	7761302	8	111069498	117801929	6732431
177	75	16	83841448	90111383	6269935	8	119344538	123536649	4192111
178	69	17	5810	4511614	4505804	11	72389164	76698664	4309500
179	150	17	4534197	12921504	8387307	11	64979038	72362442	7383404
180	14	17	15133095	16472520	1339425	11	62248984	63159547	910563
181	23	17	16945859	18266856	1320997	11	59661305	60811718	1150413
182	24	17	18561742	21323179	2761437	11	60830631	62850808	2020177
183	115	17	25621102	36105237	10484135	11	76726602	84916366	8189764
184	282	17	36337717	50237377	13899660	11	93098028	104753962	11655934
185	59	17	52976748	60142643	7165895	11	84957786	90688366	5730580
186	57	17	60447579	68176189	7728610	11	105063592	111076821	6013229
187	183	17	70117161	81052864	10935703	11	112782224	121716306	8934082
188	4	18	158383	581524	423141	18	9615524	10045119	429595
189	28	18	2571510	9960018	7388508	17	65580056	71526857	5946801
190	3	18	10454625	11148587	693962	18	62922327	63387183	464856
191	17	18	11882621	13915706	2033085	18	67225530	68429251	1203721
192	28	18	18529701	25757410	7227709	18	10064401	16809246	6744845
193	34	18	28569974	35146000	6576026	18	19960930	25753983	5793053
194	3	18	39535171	40857615	1322444	18	30272747	31447415	1174668
195	36	18	42260138	48744674	6484536	18	73573044	79109391	5536347
196	8	18	51679079	53332018	1652939	18	69344146	70626131	1281985
197	19	18	54264439	58040001	3775562	18	63662801	66860472	3197671
198	17	18	59000815	61672278	2671463	1	104768529	107608909	2840380
199	3	18	63417488	65184217	1766729	1	109982431	111864909	1882478
200	4	18	67068291	67997436	929145	18	88665224	89769528	1104304
201	2	18	70203915	70535381	331466	18	86394952	86718283	323331
202	9	18	71740588	73001905	1261317	18	84011627	84981376	969749
203	4	18	74069644	74980858	911214	18	82392496	83005314	612818
204	11	18	76740275	78005429	1265154	18	80046895	80986578	939683
205	123	19	281043	4224811	3943768	10	79526430	81647610	2121180
206	57	19	4229540	6940463	2710923	17	55952623	57483529	1530906
207	30	19	7112266	8327305	1215039	8	3150922	4525757	1374835
208	12	19	8367011	8809172	442161	17	33432897	33849774	416877
209	69	19	8920382	11689823	2769441	9	18455575	22135746	3680171
210	58	19	12721732	14682886	1961154	8	83566671	85119637	1552966
211	8	19	14938094	15236596	298502	10	78574500	78970612	396112
212	12	19	15270445	16060768	790323	17	32120820	33269676	1148856
213	102	19	16177831	19774502	3596671	8	69791163	72763874	2972711
214	6	19	30094924	31201777	1106853	7	37472135	38271348	799213
215	25	19	32836500	34997258	2160758	7	34109543	35802989	1693446
216	139	19	35491227	41314336	5823109	7	27158658	31151050	3992392
217	54	19	41699115	45004576	3305461	7	24081924	25816913	1734989
218	53	19	45116940	46544274	1427334	7	18176494	19950743	1774249
219	34	19	46800303	48364769	1564466	7	15865947	17062427	1196480
220	6	19	48373723	48700877	327154	7	13258967	14446769	1187802
221	120	19	48799714	52005043	3205329	7	43527456	45948956	2421500
222	3	19	52216365	52273760	57395	17	17843328	17971677	128349
223	71	19	54296857	57746915	3450058	7	3221510	7002945	3781435
224	2	19	58095508	58190519	95011	7	10487229	11166182	678953
225	17	19	58193357	59084942	891585	7	12415153	13054764	639611
226	16	2	38814	3836122	3797308	12	28516284	31079937	2563653
227	3	2	6980701	7208417	227716	12	26306797	26479835	173038
228	3	2	8818975	9143942	324967	12	24831599	25096092	264493
229	6	2	9346894	9771143	424249	12	21111748	21417637	305889
230	5	2	9983483	10271545	288062	12	24498581	24714146	215565

231	14	2	10443015	12882860	2439845	12	15791727	17791926	2000199
232	25	2	14772810	21266945	6494135	12	7977648	14152038	6174390
233	23	2	23608088	26360323	2752235	12	3247430	5375682	2128252
234	51	2	26395960	29025806	2629846	5	30105161	32517433	2412272
235	24	2	29005383	33824449	4819066	17	71552061	75551946	3999885
236	30	2	36583069	40838193	4255124	17	78200248	81649607	3449359
237	41	2	42275160	49381676	7106516	17	83215292	89200612	5985320
238	19	2	53759810	56613308	2853498	11	28385685	31102704	2717019
239	2	2	58134786	58468507	333721	11	26386135	26593999	207864
240	30	2	60678302	65659771	4981469	11	19924375	24174123	4249748
241	6	2	67624451	68694390	1069939	11	16951375	17953875	1002500
242	22	2	68694693	71017775	2323082	6	86017191	87672168	1654977
243	7	2	71035775	71306935	271160	6	83644542	83803420	158878
244	2	2	71336814	71377231	40417	7	64376576	64412121	35545
245	52	2	71409869	75938115	4528246	6	81923669	85961667	4037998
246	2	2	79312156	79386879	74723	6	78405155	78468872	63717
247	31	2	84650647	87089047	2438400	6	71322836	73276911	1954075
248	7	2	88326724	89050427	723703	6	70765720	71322233	556513
249	15	2	95691422	97039583	1348161	2	127103809	127656695	552886
250	50	2	97202480	103460352	6257872	1	36307733	40855944	4548211
251	9	2	105471969	106810795	1338826	1	42697146	43827800	1130654
252	3	2	108602979	108926371	323392	17	53829637	54299034	469397
253	5	2	109065017	110371783	1306766	10	58255497	59221847	966350
254	18	2	110841447	113594480	2753033	2	127704386	129371139	1666753
255	7	2	113730780	114036527	305747	2	24186476	24475599	289123
256	2	2	114462588	114720173	257585	1	125392905	125595684	202779
257	22	2	118572226	122525429	3953203	1	118298517	121567989	3269472
258	14	2	127413509	128785694	1372185	18	31634371	32560034	925663
259	2	2	128848774	129076151	227377	1	36068400	36244305	175905
260	8	2	131095814	132111282	1015468	1	34436671	34879580	442909
261	17	2	133174147	138435287	5261140	1	125676995	130219278	4542283
262	3	2	138721590	139537918	816328	2	23321246	24049394	728148
263	4	2	143635067	145282147	1647080	2	43555329	45117395	1562066
264	23	2	148602086	155714863	7112777	2	48814109	55598145	6784036
265	133	2	157180944	184026408	26845464	2	57106830	80658906	23552076
266	8	2	185463093	188430487	2967394	2	82053222	84476775	2423553
267	5	2	189156396	190448484	1292088	1	44551511	45925594	1374083
268	17	2	190611386	193060435	2449049	1	50900647	53326343	2425696
269	2	2	196440701	196933536	492835	1	46066738	46854046	787308
270	15	2	196998290	199437305	2439015	1	53755506	55751802	1996296
271	145	2	200134223	220506702	20372479	1	56793986	75562172	18768186
272	16	2	222282747	226518734	4235987	1	77367185	81341764	3974579
273	119	2	227599757	242801060	15201303	1	82233101	94052553	11819452
274	25	20	68351	1448417	1380066	2	151494182	152511692	1017510
275	58	20	1520790	6760910	5240120	2	129592835	133562885	3970050
276	11	20	7863628	10654608	2790980	2	134497361	137116644	2619283
277	59	20	12989627	25566153	12576526	2	139493913	151039382	11545469
278	117	20	29845467	37668366	7822899	2	152518255	158858214	6339959
279	112	20	39314488	53267710	13953222	2	160363677	170879769	10516092
280	31	20	54572496	57901047	3328551	2	172036233	174784042	2747809
281	5	20	58152564	58609066	456502	2	178118975	178487366	368391
282	57	20	59827559	62926855	3099296	2	179442431	181857461	2415030
283	5	21	15588451	17252377	1663926	16	75592844	77116779	1523935
284	4	21	18884700	19858197	973497	16	78301496	79091097	789601
285	8	21	26957968	28338832	1380864	16	84717576	85901125	1183549
286	91	21	30244513	43430496	13185983	16	87354185	97962621	10608436
287	19	21	43483068	45115958	1632890	17	30954679	32062865	1108186
288	38	21	45138975	48085036	2946061	10	76207222	78464975	2257753
289	11	22	17565844	18614498	1048654	6	120463197	121226854	763657
290	53	22	18893541	22599927	3706386	16	16868402	18970309	2101907
291	23	22	23401593	25005947	1604354	10	74957477	75949657	992180
292	15	22	25202236	27026636	1824400	5	112246493	113310786	1064293
293	5	22	28144265	29153503	1009238	5	110829070	111457033	627963
294	19	22	29168662	31521442	2352780	11	4480868	5542187	1061319
295	29	22	30476163	32014572	1538409	11	3131850	4441105	1309255
296	4	22	32072242	32509016	436774	5	32789218	33162872	373654
297	5	22	32783569	33454358	670789	10	85938637	86498896	560259
298	5	22	35462129	35950048	487919	8	74872719	75224113	351394
299	159	22	36002811	47571336	11568525	15	77015489	86498503	9483014
300	35	22	48885272	51222091	2336819	15	87625230	89591923	1966693
301	12	3	238279	5261642	5023363	6	103510876	108859356	5348480
302	49	3	6811688	12913415	6101727	6	110645581	115808747	5163166
303	16	3	12938719	15140670	2201951	6	90659598	92214925	1555327
304	9	3	15247659	16273499	1025840	14	31336638	32062197	725559
305	11	3	16357352	20227784	3870432	17	49992257	53689333	3697076
306	5	3	23244511	24536773	1292262	14	17660960	18894267	1233307
307	7	3	25215823	27525911	2310088	14	14703025	16575472	1872447

308	4	3	27757440	28579613	822173	9	117876000	118486132	610132
309	20	3	30647994	33911194	3263200	9	113651744	116175363	2523619
310	7	3	35680437	37225180	1544743	9	111118111	112235938	1117827
311	76	3	37284668	46402419	9117751	9	118506318	124113557	5607239
312	128	3	46448654	52329272	5880618	9	106152857	111057270	4904413
313	43	3	52350335	57914895	5564560	14	26413168	31323896	4910728
314	22	3	57994127	64009658	6015531	14	7817957	14120984	6303027
315	26	3	64079543	74570291	10490748	6	92370892	102464667	10093775
316	7	3	86987119	88207118	1219999	16	64751751	65863057	1111306
317	3	3	93591881	93774512	182631	16	62793685	62929346	135661
318	30	3	96533425	102198685	5665260	16	55225175	60605531	5380356
319	2	3	105085753	105588396	502643	16	52031549	52454074	422525
320	12	3	107096188	109056419	1960231	16	48283735	50591154	2307419
321	34	3	110788918	117716095	6927177	16	41532858	46498525	4965667
322	60	3	118619404	125313934	6694530	16	33185071	39027159	5842088
323	32	3	125725198	129035120	3309922	6	87730869	90646412	2915543
324	6	3	129149787	129612419	462632	6	115840697	116193486	352789
325	74	3	130064359	143767561	13703202	9	94517864	105960643	11442779
326	5	3	145787227	147228080	1440853	9	91358058	92608428	1250370
327	6	3	148508889	148939842	430953	3	19957054	20275733	318679
328	35	3	149086809	155658457	6571648	3	57285611	64022579	6736968
329	27	3	155755490	161221730	5466240	3	65109384	70028708	4919324
330	3	3	164696686	165555260	858574	3	72888557	73708415	819858
331	6	3	166958075	167813763	855688	3	75037907	75956949	919042
332	14	3	168801287	170578169	1776882	3	29951299	31310378	1359079
333	12	3	170582664	172859058	2276394	3	26637620	28807352	2169732
334	17	3	177990720	181432221	3441501	3	31902507	34652461	2749954
335	3	3	182511288	182833863	322575	3	35754134	36000678	246544
336	111	3	182840001	197770591	14930590	16	19653381	33127695	13474314
337	13	4	492989	1107350	614361	5	108312609	108749448	436839
338	73	4	1160720	11431389	10270669	5	33213518	39755475	6541957
339	3	4	13362978	13629347	266369	5	41624976	41844168	219192
340	17	4	15004298	18023499	3019201	5	43233463	45857615	2624152
341	3	4	20254883	21950422	1695539	5	47983155	49524904	1541749
342	16	4	23756664	27027003	3270339	5	51454249	54121057	2666808
343	36	4	36067620	43032675	6965055	5	62602445	68166398	5563953
344	4	4	44175926	44728612	552686	5	69109285	69592337	483052
345	19	4	46037786	49064098	3026312	5	70751047	73453435	2702388
346	34	4	52709166	57976551	5267385	5	73481002	77408045	3927043
347	2	4	65140975	66536213	1395238	5	83278145	84417382	1139237
348	69	4	68337521	78354542	10017021	5	86012043	93276231	7264188
349	50	4	78432907	89152474	10719567	5	95956928	104982718	9025790
350	3	4	89183315	89444964	261649	6	57506502	57692078	185576
351	5	4	89444961	90229161	784200	6	58831465	59426290	594825
352	3	4	90645250	92523064	1877814	6	60731573	62382865	1651292
353	3	4	94750042	95264027	513985	6	64729146	65144908	415762
354	3	4	95373037	96470357	1097320	3	141465564	142395696	930132
355	60	4	98105244	111563279	13458035	3	129199878	139710299	10510421
356	12	4	113066553	116035032	2968479	3	125404091	127896323	2492232
357	11	4	118004718	120550146	2545428	3	122729158	124323260	1594102
358	5	4	120980577	122302214	1321637	6	65381105	66541033	1159928
359	14	4	122589110	124324910	1735800	3	36448925	37644598	1195673
360	11	4	128544426	130034487	1490061	3	40531286	41759389	1228103
361	11	4	138440072	141075338	2635266	3	49743296	52104891	2361595
362	9	4	141178440	142655140	1476700	8	82331637	83458391	1126754
363	22	4	144106070	149365850	5259780	8	76899442	81014906	4115464
364	42	4	150999426	160281321	9281895	3	79062516	86920884	7858368
365	12	4	164031225	167025047	2993822	8	64014770	66890564	2875794
366	10	4	169013666	171012850	1999184	8	60506124	62123118	1616994
367	19	4	172733405	178363657	5630252	8	53511702	57962564	4450862
368	33	4	183065140	187647876	4582736	8	44950208	48674690	3724482
369	3	4	188916925	189068897	151972	8	43129807	43307009	177202
370	25	5	191626	3601517	3409891	13	71957921	74365783	2407862
371	11	5	5140443	7906138	2765695	13	68560780	70841811	2281031
372	9	5	9035138	10761384	1726246	15	31224385	32696341	1471956
373	11	5	13690440	17276943	3586503	15	25363285	28472045	3108760
374	43	5	31193857	39462402	8268545	15	6299788	13173639	6873851
375	14	5	40679600	42887494	2207894	15	3268547	5244187	1975640
376	12	5	43039335	45696253	2656918	13	117602480	120027011	2424531
377	3	5	49692026	50690564	998538	13	116298281	117274098	975817
378	31	5	52083730	58155213	6071483	13	110054187	115101964	5047777
379	9	5	59892739	61924409	2031670	13	106794439	108407782	1613343
380	18	5	63256183	66492627	3236444	13	102693558	105448133	2754575
381	11	5	67511548	68890550	1379002	13	100460218	101768217	1307999
382	71	5	70264310	83680611	13416301	13	88821472	100446406	11624934
383	5	5	85913721	88199922	2286201	13	83504034	86046904	2542870
384	6	5	89688078	90679176	991098	13	80883384	81797143	913759

385	17	5	92919043	96143803	3224760	13	74639872	78199757	3559885
386	3	5	96271098	96518964	247866	17	17374332	17624489	250157
387	2	5	98104354	98262240	157886	17	15704967	15826586	121619
388	2	5	99871009	100238970	367961	1	95313628	95667594	353966
389	6	5	101569690	102614361	1044671	1	96818784	98095646	1276862
390	6	5	106712590	110074657	3362067	17	62604184	65540782	2936598
391	9	5	110405760	112258236	1852476	18	32815383	34373415	1558032
392	16	5	112312399	115910630	3598231	18	44380500	47368870	2988370
393	6	5	118173017	120023027	1850010	18	49696145	51304641	1608496
394	11	5	121187650	122952739	1765089	18	52331536	53955684	1624148
395	17	5	125695824	129522327	3826503	18	56432132	59410446	2978314
396	38	5	130494720	134063513	3568793	11	51692264	54870501	3178237
397	20	5	134074191	137090039	3015848	13	55623005	58128556	2505551
398	85	5	137223657	143856944	6633287	18	34409423	40531168	6121745
399	19	5	144851362	147594700	2743338	18	41875696	44083610	2207914
400	32	5	147647743	150176296	2528553	18	60474193	62741387	2267194
401	14	5	150400124	151812929	1412805	11	54902453	56041010	1138557
402	10	5	152869175	154348971	1479796	11	57011387	58179565	1168178
403	31	5	156346293	161582542	5236249	11	41910195	46844332	4934137
404	4	5	162864575	162946342	81767	11	40679314	40755311	75997
405	21	5	167718656	171881527	4162871	11	32347820	35980527	3632707
406	7	5	172068269	172662360	594091	17	26414965	26841565	426600
407	4	5	172741716	173670504	928788	11	31357307	32059202	701895
408	41	5	174151536	177037348	2885812	13	53466881	55610443	2143562
409	40	5	177419236	180675096	3255860	11	48800332	51650842	2850510
410	44	6	292097	8435794	8143697	13	30659999	38960875	8300876
411	23	6	9596343	14137149	4540806	13	40001882	43803130	3801248
412	16	6	15246527	18469105	3222578	13	44729474	47247991	2518517
413	4	6	20100935	21598847	1497912	13	28948919	30246717	1297798
414	34	6	24126350	26659980	2533630	13	23551258	25270502	1719244
415	16	6	27215480	29013017	1797537	13	21754123	22009742	255619
416	127	6	29141311	33297046	4155735	17	33909414	38305460	4396046
417	64	6	33378176	39055519	5677343	17	26933127	30936510	4003383
418	3	6	39071840	39290744	218904	14	20075646	20269162	193516
419	99	6	39297766	48036425	8738659	17	42315947	49909847	7593900
420	7	6	49398073	49755053	356980	17	40207018	40961989	754971
421	13	6	49801970	52551386	2749416	1	18115191	21230167	3114976
422	14	6	52866077	55740362	2874285	9	75775364	78172107	2396743
423	5	6	56322785	57087078	764293	1	33719882	34308650	588768
424	3	6	63985856	64489229	503373	1	30802342	31204656	402314
425	8	6	70385694	72011973	1626279	1	23366424	24766299	1399875
426	2	6	73331520	73972919	641399	1	21368331	21961942	593611
427	7	6	74078278	74538040	459762	9	78376109	78716253	340144
428	7	6	75794042	76782395	988353	9	79598991	80465438	866447
429	8	6	79577189	81055987	1478798	9	82829806	84124239	1294433
430	18	6	82201156	86353510	4152354	9	85320439	88482574	3162135
431	25	6	87795216	91296764	3501548	4	31964097	34907370	2943273
432	8	6	96025419	97731093	1705674	4	24496451	26346891	1850440
433	8	6	99282580	100063454	780874	4	21677480	22488366	810886
434	2	6	100832891	101329248	496357	10	50592669	50989152	396483
435	50	6	105175968	112672498	7496530	10	38820541	45712345	6891804
436	3	6	114178541	114664209	485668	10	36506814	37138920	632106
437	13	6	116262693	116989957	727264	10	33905111	34611226	706115
438	14	6	117073363	119670926	2597563	10	51585420	54075796	2490376
439	2	6	121400640	121770873	370233	10	56014298	56390419	376121
440	5	6	122720691	123130865	410174	10	57486385	57811830	325445
441	74	6	123317116	139695757	16378641	10	17723228	33624769	15901541
442	51	6	142379467	154568001	12188534	10	3366150	14770583	11404433
443	5	6	155054459	155777037	722578	17	3114972	3696261	581289
444	15	6	157099063	159693141	2594078	17	4994332	7934897	2940565
445	14	6	160100096	161695093	1594997	17	12118704	13018119	899415
446	2	6	163148164	163999628	851464	17	10206471	10840311	633840
447	4	6	165693153	166721936	1028783	17	8340406	9008319	667913
448	4	6	166822852	167553184	730332	17	6978860	8257127	1278267
449	14	6	168227602	170893780	2666178	17	13760539	15528379	1767840
450	64	7	192969	6866401	6673432	5	138754514	144014853	5260339
451	8	7	7196565	8792593	1596028	6	7844842	9249032	1404190
452	4	7	10971578	12276886	1305308	6	11900373	13089269	1188896
453	2	7	12610203	12730559	120356	12	40005447	40134228	128781
454	19	7	13930853	19748710	5817857	12	33429624	38868215	5438591
455	9	7	19758933	22396763	2637830	12	117516479	120018706	2502227
456	4	7	22852251	23240630	388379	5	23838944	24184013	345069
457	58	7	23275586	33102409	9826823	6	49036518	56923932	7887414
458	11	7	33134409	36493400	3358991	9	22331214	25604110	3272896
459	20	7	36552456	43602938	7050482	13	14226438	21024252	6797814
460	27	7	43906157	45961473	2055316	11	5703983	7213923	1509940
461	7	7	47314752	48687092	1372340	11	8431652	9684259	1252607

462	8	7	49813257	51384515	1571258	11	11114223	12464960	1350737
463	3	7	54610018	55324313	714295	11	16257724	16918158	660434
464	2	7	55433141	55640681	207540	6	57702601	57825154	122553
465	9	7	55861237	56184093	322856	5	129683402	129910396	226994
466	9	7	65338254	66704501	1366247	5	129942109	130341563	399454
467	2	7	70597155	71912148	1314993	5	130369476	131308078	938602
468	43	7	72716514	76648340	3931826	5	134099711	136045303	1945592
469	7	7	76751751	77586818	835067	5	20758663	21424677	666014
470	6	7	79763271	82073114	2309843	5	15934691	18360413	2425722
471	3	7	82387442	84122040	1734598	5	13396784	14863457	1466673
472	15	7	86273230	88966346	2693116	5	6769030	9725352	2956322
473	18	7	89783689	92465908	2682219	5	3343893	5749326	2405433
474	25	7	92759368	97501854	4742486	6	3372257	7693254	4320997
475	22	7	97736197	99463718	1727521	5	144100436	145469723	1369287
476	59	7	99520892	102232891	2711999	5	136054492	138280005	2225513
477	15	7	102389418	105208124	2818706	5	21424958	23820369	2395411
478	17	7	105205567	107643700	2438133	12	31265234	33401269	2136035
479	3	7	107788082	108215294	427212	12	44205897	44601846	395949
480	6	7	110303110	112130942	1827832	12	40176386	41955588	1779202
481	7	7	112405787	114659256	2253469	6	13580687	15802165	2221478
482	13	7	115575202	117882785	2307583	6	16833381	18879586	2046205
483	23	7	119913722	124405681	4491959	6	21215503	25690729	4475226
484	137	7	126078652	144533488	18454836	6	27275121	43666278	16391157
485	25	7	148395006	150558592	2163586	6	47453398	48909188	1455790
486	24	7	150642049	152552463	1910414	5	24319589	25850342	1530753
487	12	7	154735397	157062066	2326669	5	27740667	29676077	1935410
488	5	7	157331750	158937649	1605899	12	116077726	117278167	1200441
489	9	8	356428	4852494	4496066	8	13907806	17535586	3627780
490	3	8	6264113	6617184	353071	8	18595131	18891361	296230
491	2	8	6728097	6914256	186159	8	21618183	21795185	177002
492	2	8	7705398	7740186	34788	8	19140759	19195212	54453
493	6	8	8175258	9639856	1464598	8	34829179	36147787	1318608
494	17	8	9911778	12175825	2264047	14	62998102	64455903	1457801
495	19	8	12579403	17942494	5363091	8	36216064	41374773	5158709
496	9	8	18248755	20161474	1912719	8	67490758	69140953	1650195
497	70	8	21547915	29120641	7572726	14	64652531	70979838	6327307
498	13	8	29190581	31031285	1840704	8	33234384	34819894	1585510
499	6	8	31496902	33457624	1960722	8	31089471	31918203	828732
500	9	8	37553269	37917883	364614	8	26977336	27275656	298320
501	35	8	37962760	42408151	4445391	8	22462615	25847694	3385079
502	2	8	42552519	42651535	99016	8	27368711	27413944	45233
503	6	8	42691817	43057998	366181	8	25944459	26162940	218481
504	5	8	48173167	49834299	1661132	16	14705852	16146851	1440999
505	13	8	52730140	55543394	2813254	1	4343507	7173626	2830119
506	10	8	56608983	57906403	1297420	4	3549041	4793355	1244314
507	6	8	58907068	60031767	1124699	4	5644090	6991557	1347467
508	5	8	61099906	62627155	1527249	4	8143367	9669344	1525977
509	3	8	63161150	63998612	837462	4	20007938	20778866	770928
510	8	8	65492814	67090960	1598146	3	18054325	19695396	1641071
511	38	8	67341263	75946793	8605530	1	9545408	17766344	8220936
512	2	8	77593454	77913280	319826	3	5218526	5576239	357713
513	20	8	79428374	82755101	3326727	3	7366604	10440124	3073520
514	7	8	85095022	86393693	1298671	3	13946372	14900769	954397
515	7	8	86999552	88627447	1627895	4	18860454	19922605	1062151
516	12	8	90769975	93115514	2345539	4	13743436	16163647	2420211
517	17	8	94710789	96281429	1570640	4	10874498	12172015	1297517
518	3	8	97238148	97349223	111075	13	66900617	66998401	97784
519	57	8	97505579	110988076	13482497	15	32920723	45114934	12194211
520	21	8	116420724	122653630	6232906	15	50654761	56694539	6039778
521	23	8	123793633	126450647	2657014	15	57694665	59656550	1961885
522	18	8	130760442	136668965	5908523	15	63891265	69093512	5202247
523	12	8	139142266	142528837	3386571	15	71445678	73839908	2394230
524	69	8	143530791	146281416	2750625	15	74516196	76950729	2434533
525	32	9	214854	6645650	6430796	19	24999529	30175418	5175889
526	3	9	6720863	10612723	3891860	4	74242497	78211961	3969464
527	34	9	12685439	22452472	9767033	4	80834123	89694772	8860649
528	2	9	23690102	24545944	855842	4	91250763	92147234	896471
529	6	9	26840683	27297137	456454	4	94500081	94928843	428762
530	4	9	27325207	28670283	1345076	4	34949074	36951747	2002673
531	89	9	32384618	38424444	6039826	4	40143081	45826923	5683842
532	14	9	70175707	73029540	2853833	19	23141226	24901309	1760083
533	9	9	74298282	75785309	1487027	19	20373428	21858327	1484899
534	16	9	77112281	80945009	3832728	19	15904678	19111196	3206518
535	2	9	85594500	86153461	558961	4	73714579	74202214	487635
536	17	9	86237964	90346308	4108344	13	58157649	61040631	2882982
537	8	9	91003334	92221470	1218136	13	51100880	51848468	747588
538	6	9	93372114	94877690	1505576	13	52504375	53377365	872990

539	19	9	94972489	97063736	2091247	13	48487647	49734267	1246620
540	14	9	97321002	99801925	2480923	13	62836884	65112982	2276098
541	38	9	100000765	104500862	4500097	4	45890303	49845744	3955441
542	12	9	106856541	108538893	1682352	4	52439243	53862019	1422776
543	3	9	109625378	110252763	627385	4	54945048	55532466	587418
544	33	9	111616871	115653193	4036322	4	56740007	59915056	3175049
545	26	9	115800660	117880536	2079876	4	62189540	64047015	1857475
546	2	9	118916083	120177348	1261265	4	65124174	66404611	1280437
547	2	9	123151147	123476748	325601	4	70216856	70534995	318139
548	251	9	123514256	141019076	17504820	2	24603887	39226451	14622564
549	2	X	192989	230886	37897	5	110099969	110108197	8228
550	2	X	9693386	9917483	224097	X	152609509	152808646	199137
551	4	X	10413350	11793870	1380520	X	168654117	170005736	1351619
552	45	X	12809474	20285523	7476049	X	159210307	167382749	8172442
553	7	X	21392536	22269427	876891	X	157162075	158043294	881219
554	4	X	23352133	23804343	452210	X	155213132	155623814	410682
555	5	X	24001837	25034065	1032228	X	93286507	94277724	991217
556	2	X	26210557	26236387	25830	X	91392500	91635671	243171
557	6	X	30233677	31090170	856493	X	85249679	86305093	1055414
558	3	X	35816459	36163187	346728	X	79401625	79671435	269810
559	11	X	37430822	38665790	1234968	X	9199902	10719690	1519788
560	10	X	39909068	41782716	1873648	X	12036740	13851367	1814627
561	8	X	43515467	45060146	1544679	X	16619698	18461397	1841699
562	17	X	46433219	47869126	1435907	X	20059560	21077963	1018403
563	41	X	48316920	51151687	2834767	X	6081219	8280179	2198960
564	2	X	51486481	51645453	158972	X	94535474	94643244	107770
565	24	X	53078273	56593443	3515170	X	150408584	153501537	3092953
566	65	X	62567107	75651744	13084637	X	95022507	105123909	10101402
567	17	X	76709648	80554046	3844398	X	105764279	109197873	3433594
568	11	X	82763269	86925050	4161781	X	110814390	114560829	3746439
569	82	X	99546642	112084043	12537401	X	133582863	145505181	11922318
570	3	X	113818551	114468635	650084	X	146962513	147554081	591568
571	2	X	115301975	115592625	290650	X	21484544	21742355	257811
572	2	X	117031776	117583924	552148	X	23219271	23806025	586754
573	25	X	117629861	119709649	2079788	X	35888832	38576196	2687364
574	5	X	122318006	124097666	1779660	X	41400854	43429126	2028272
575	2	X	125683369	125955769	272400	X	44365458	45092791	727333
576	17	X	128580480	130533677	1953197	X	47809368	49797749	1988381
577	16	X	131157293	134305322	3148029	X	50841047	53609132	2768085
578	14	X	134478721	136659850	2181129	X	56346400	58041736	1695336
579	7	X	137713735	139866723	2152988	X	59062145	61185558	2123413
580	75	X	146993469	154493874	7500405	X	68678541	75578231	6899690
581	3	Y	14813160	15592553	779393	Y	1096861	1459782	362921
