# Supplementary material for: The impact of the protein interactome on the syntenic structure of mammalian genomes
Source: PLoS One. 2017 Sep 14;12(9):e0179112. doi: 10.1371/journal.pone.0179112 (PMC5598925; doi:10.1371/journal.pone.0179112)
Supplement: S3 Table — The blocks are ordered by the location on the human genome. There are 600 blocks in total containing 14,048 orthologous protein-coding genes. (PDF) [file pone.0179112.s005.pdf]

Block No	Gene No	Human Chr	Human Start	Human End	Human Length	Pig Chr	Pig Start	Pig End	Pig Length
1	50	1	879584	3816857	2937273	6	57819109	60163771	2344662
2	19	1	5922871	6761984	839113	6	61659254	62451639	792385
3	50	1	7979907	13944452	5964545	6	63023405	67253156	4229751
4	156	1	14925200	29653325	14728125	6	68659247	80106273	11447026
5	45	1	31184124	34684732	3500608	6	81047913	84361009	3313096
6	17	1	35734568	38512450	2777882	6	85026747	86800967	1774220
7	23	1	39303870	40783488	1479618	6	87836131	89434966	1598835
8	81	1	40810522	48937845	8127323	6	150809986	157721955	6911969
9	48	1	50883222	55681039	4797817	6	145323643	149325034	4001391
10	6	1	56960419	59012406	2051987	6	142972837	143732390	759553
11	31	1	58881056	68915642	10034586	6	133492012	141544308	8052296
12	8	1	70034081	72748417	2714336	6	130680416	132436579	1756163
13	23	1	74491699	79472403	4980704	6	124520030	128766182	4246152
14	6	1	84330711	85462796	1132085	6	119212826	120062240	849414
15	16	1	85464830	87812788	2347958	4	141341709	143464005	2122296
16	37	1	89149905	95712781	6562876	4	133994711	139885791	5891080
17	21	1	99127236	104239302	5112066	4	126313702	130857116	4543414
18	111	1	107682629	120439118	12756489	4	111216207	123210247	11994040
19	19	1	145413095	147245484	1832389	4	108972376	110219875	1247499
20	137	1	149856010	155532598	5676588	4	104902529	108845456	3942927
21	145	1	155657751	163325554	7667803	4	94875738	103077786	8202048
22	44	1	164524821	170708560	6183739	4	87980699	93629359	5648660
23	6	1	170904612	171311223	406611	9	69851162	70221216	370054
24	82	1	171454651	186958113	15503462	9	125431812	140623439	15191627
25	7	1	192127587	193223031	1095444	10	2258881	3305931	1047050
26	10	1	196194909	198726545	2531636	10	23896560	26332284	2435724
27	30	1	199996730	202936408	2939678	10	27281168	29375782	2094614
28	7	1	202976514	203242769	266255	9	125173393	125399889	226496
29	54	1	203274619	207968858	4694239	9	70327445	74604181	4276736
30	2	1	208057594	208417665	360071	9	148127165	148470659	343494
31	35	1	209757062	215410436	5653374	9	141099509	146616210	5516701
32	24	1	215740735	222924147	7183412	10	7630324	13772545	6142221
33	2	1	223282748	223537544	254796	10	21886309	22050197	163888
34	2	1	223967601	224349749	382148	10	24461166	24606907	145741
35	19	1	224363458	227175246	2811788	10	15003501	17115434	2111933
36	9	1	227916240	228369958	453718	2	53824455	54420126	595671
37	2	1	228395831	228700004	304173	2	78798040	78909313	111273
38	50	1	228870824	238129359	9258535	14	57496100	65517687	8021587
39	6	1	240652873	241965435	1312562	10	14012392	14937373	924981
40	16	1	242011269	246831886	4820617	10	17632831	21749788	4116957
41	17	1	247460714	248437138	976424	2	55656567	58026156	2369589
42	4	1	248524883	248790491	265608	2	161501749	161931129	429380
43	4	1	248902716	249153343	250627	2	55188353	55357982	169629
44	7	10	225953	1779670	1553717	10	75201376	76366335	1164959
45	3	10	3109712	3827473	717761	10	72992245	73711208	718963
46	21	10	4828820	8117161	3288341	10	69581080	72022986	2441906
47	10	10	11047259	13141652	2094393	10	64772795	66405361	1632566
48	2	10	13628927	14504141	875214	10	52757463	52821675	64212
49	22	10	14861249	18940551	4079302	10	47746354	51686809	3940455
50	12	10	21068902	23633774	2564872	10	57355457	59713946	2358489
51	16	10	23983675	28591995	4608320	10	52979467	56317981	3338514
52	12	10	28821422	32667726	3846304	10	44120645	47541566	3420921
53	7	10	33189247	35897863	2708616	10	61450695	63683060	2232365
54	6	10	43278249	43904614	626365	14	65917857	66498593	580736
55	11	10	44051792	46168228	2116436	14	98686346	100717407	2031061
56	29	10	46310876	51732941	5422065	14	95170381	98625342	3454961
57	6	10	52065360	54077802	2012442	14	106114971	108105320	1990349
58	18	10	60272900	65384883	5111983	14	66692886	72357184	5664298
59	78	10	67679719	77161664	9481945	14	75080133	84025215	8945082
60	3	10	78637355	79789303	1151948	14	85796582	87003286	1206704
61	13	10	80828792	82406316	1577524	14	88066512	89072555	1006043
62	7	10	85899196	86278273	379077	14	92190363	92533125	342762
63	8	10	88195013	88951225	756212	14	94748436	95597448	849012
64	99	10	89264632	102124591	12859959	14	108506965	120980914	12473949
65	4	10	102222798	102309763	86965	4	63896390	63960356	63966
66	41	10	102495360	105050108	2554748	14	121492419	124181132	2688713

67	2	10	105148798	105206049	57251	14	50545515	50564960	19445
68	12	10	105253736	107024993	1771257	14	124318583	126181245	1862662
69	10	10	111624524	112840658	1216134	14	131108869	132405246	1296377
70	57	10	113909624	124274424	10364800	14	133473306	143558530	10085224
71	2	10	124320181	124459338	139157	6	43611343	43753137	141794
72	31	10	124608594	129924649	5316055	14	143940236	149198585	5258349
73	26	10	131265448	135382876	4117428	14	150607123	153798758	3191635
74	11	11	167784	417455	249671	2	162119619	162319222	199603
75	22	11	537527	1036706	499179	2	39211	368934	329723
76	5	11	2920951	3187969	267018	2	428408	612728	184320
77	104	11	3659733	9550071	5890338	9	4109782	7212089	3102307
78	61	11	9595228	20530840	10935612	2	41911669	53244088	11332419
79	4	11	22214722	22851845	637123	2	39939040	40466246	527206
80	9	11	26210829	28355054	2144225	2	34616656	36430350	1813694
81	42	11	30031288	36619829	6588541	2	26712072	33074381	6362309
82	58	11	43333513	48328704	4995191	2	15272577	20698709	5426132
83	2	11	49075266	49230222	154956	9	25556418	25687747	131329
84	2	11	55339604	55371874	32270	2	14957207	15401209	444002
85	5	11	55405834	55703876	298042	2	158558893	158793346	234453
86	7	11	55734975	56238014	503039	1	312203726	312753489	549763
87	2	11	56309746	56380978	71232	2	159304160	159342322	38162
88	274	11	56510303	70053496	13543193	2	1563459	14031853	12468394
89	2	11	71139239	71239227	99988	2	725643	864461	138818
90	70	11	71639747	79151992	7512245	9	7293518	14562328	7268810
91	8	11	82443053	85338966	2895913	9	18783535	19503322	719787
92	11	11	85339629	86666433	1326804	9	21817760	23006976	1189216
93	7	11	87846431	89956532	2110101	9	23798576	26060761	2262185
94	26	11	92085262	96123087	4037825	9	28144709	32435117	4290408
95	28	11	98891683	105969437	7077754	9	35088725	39984846	4896121
96	55	11	107197071	114466484	7269413	9	40006179	46990896	6984717
97	69	11	116618886	121504387	4885501	9	49208934	53907681	4698747
98	54	11	122526383	126873355	4346972	9	54975989	59461175	4485186
99	14	11	128328656	130786404	2457748	9	61508892	63829935	2321043
100	10	11	133710526	134281812	571286	9	66934893	67639708	704815
101	126	12	175931	16763528	16587597	5	59455717	71817700	12361983
102	18	12	18233803	22218608	3984805	5	54719247	58242816	3523569
103	30	12	24964295	30907885	5943590	5	46786251	52594736	5808485
104	2	12	31079362	31257725	178363	5	69160419	69629666	469247
105	8	12	32112304	34182629	2070325	5	43574116	45582548	2008432
106	36	12	39040624	49076021	10035397	5	72799795	82779984	9980189
107	189	12	49082247	60176395	11094148	5	14923946	26867980	11944034
108	45	12	62654119	73059422	10405303	5	29581112	39518064	9936952
109	11	12	74931551	76953589	2022038	5	41332680	43256417	1923737
110	3	12	77157368	77459360	301992	5	109254252	109529012	274760
111	11	12	79257773	83528649	4270876	5	103774482	107014135	3239653
112	5	12	85253492	86889092	1635600	5	100820116	102136116	1316000
113	7	12	88373816	90103077	1729261	5	97596830	99207198	1610368
114	5	12	91299399	91576900	277501	5	96109666	96364864	255198
115	24	12	92536286	97347129	4810843	5	91290870	95091191	3800321
116	3	12	98987369	99129204	141835	5	89443057	89615244	172187
117	38	12	100422233	105630016	5207783	5	82604179	88227198	5623019
118	13	12	106457118	108170421	1713303	5	12409905	14290637	1880732
119	25	12	108523248	110477568	1954320	14	43479365	45179160	1699795
120	18	12	110562140	112194903	1632763	14	33384232	34971540	1587308
121	27	12	112204691	115121969	2917278	14	40012557	42409687	2397130
122	19	12	116395711	120315095	3919384	14	34836041	38661754	3825713
123	21	12	120427673	121477045	1049372	14	42566601	43431814	865213
124	58	12	121570622	126146917	4576295	14	29391629	33340456	3948827
125	3	12	128751948	129469509	717561	14	27626127	28290323	664196
126	19	12	130647004	133589154	2942150	14	23993466	26524159	2530693
127	3	13	20207788	20437776	229988	11	86858326	87004308	145982
128	9	13	20977806	22278637	1300831	11	5725	719167	713442
129	6	13	23755091	24896096	1141005	11	1619169	2256282	637113
130	4	13	24995064	25497018	501954	11	87072253	87426844	354591
131	63	13	25735822	41345309	15609487	11	2479151	15775714	13296563
132	31	13	41506056	47471169	5965113	11	20896282	26610523	5714241
133	30	13	48510622	53050485	4539863	11	16183285	20123486	3940201

134	4	13	53226844	53626196	399352	11	26394101	27052064	657963
135	2	13	60239717	61148012	908295	11	34600373	35171272	570899
136	6	13	72012098	73651676	1639578	11	48302561	49870148	1567587
137	3	13	75858808	76434004	575196	11	52159564	52743325	583761
138	13	13	77454312	80915086	3460774	11	53668524	56944768	3276244
139	6	13	93879095	95953687	2074592	11	69611870	70827852	1215982
140	32	13	96085858	103719196	7633338	11	71983268	78629176	6645908
141	14	13	107142093	111996596	4854503	11	81565082	85021609	3456527
142	16	13	113139325	114898086	1758761	11	85779294	86763491	984197
143	23	14	20691791	22134238	1442447	7	82869105	83701704	832599
144	67	14	23033805	25519503	2485698	7	79510868	81693978	2183110
145	33	14	31028329	39578850	8550521	7	65656356	73541815	7885459
146	4	14	39583427	39901704	318277	1	188204952	188461143	256191
147	8	14	44973545	45722743	749198	1	194527560	195286683	759123
148	2	14	47120222	48144157	1023935	1	196774308	197065367	291059
149	78	14	50087489	62568431	12480942	1	199864396	212460318	12595922
150	6	14	63670832	64804830	1133998	1	215715909	216799044	1083135
151	110	14	64854749	78401355	13546606	7	94846565	106901426	12054861
152	4	14	80663873	82000205	1336332	7	109298356	110446103	1147747
153	64	14	88304164	97398059	9093895	7	116348931	125310472	8961541
154	10	14	99635624	100996640	1361016	7	127764460	128710199	945739
155	2	14	101192042	101351184	159142	7	132097258	132425665	328407
156	25	14	102027688	105647660	3619972	7	128824303	131347987	2523684
157	2	15	23810454	23891175	80721	1	158314803	158363477	48674
158	4	15	25582381	27194354	1611973	1	156791277	157881006	1089729
159	2	15	28000021	28567298	567277	15	63775197	64060069	284872
160	9	15	29129629	32162992	3033363	1	159292514	161062878	1770364
161	2	15	32933877	33026870	92993	1	152695780	152746405	50625
162	8	15	34260921	34659479	398558	7	86137946	86422239	284293
163	5	15	35043233	35838394	795161	1	151769327	152455031	685704
164	6	15	36871812	38857776	1985964	1	148187752	150211065	2023313
165	99	15	39873280	45968512	6095232	1	140423061	146923333	6500272
166	40	15	47476298	53083273	5606975	1	131981654	138096757	6115103
167	11	15	55473004	57210769	1737765	1	127597935	129169275	1571340
168	18	15	57884139	61521518	3637379	1	123140555	126509527	3368972
169	25	15	62144588	65321977	3177389	1	117634396	121480032	3845636
170	42	15	65409717	72410918	7001201	1	180681323	188148673	7467350
171	47	15	72452148	76020029	3567881	7	65167884	65619545	451661
172	11	15	76196200	78113242	1917042	7	60852114	62379646	1527532
173	46	15	78276378	84708594	6432216	7	52770271	57867976	5097705
174	2	15	85923802	86338261	414459	7	94532329	94624727	92398
175	6	15	88402982	89199714	796732	1	212617012	213258768	641756
176	31	15	89346674	91506349	2159675	7	58161247	60674313	2513066
177	5	15	92396925	93632433	1235508	7	91815033	93045715	1230682
178	15	15	98980391	101817705	2837314	1	153304674	156066216	2761542
179	4	15	101821715	102264807	443092	1	311022067	311644920	622853
180	121	16	96407	4389598	4293191	3	40613487	42882669	2269182
181	20	16	4390252	5116111	725859	3	38352516	39040299	687783
182	27	16	8619502	12061925	3442423	3	32126183	34979051	2852868
183	4	16	14014014	14763093	749079	3	29633734	30376792	743058
184	36	16	18995256	23392620	4397364	3	23320380	27263572	3943192
185	7	16	23399814	23681195	281381	10	2661	254079	251418
186	6	16	23847322	25269252	1421930	3	21828112	22999871	1171759
187	69	16	27214807	31520630	4305823	3	17341206	19886801	2545595
188	12	16	46614466	51185278	4570812	6	30222481	33224708	3002227
189	11	16	55357672	56687116	1329444	6	26353888	27671946	1318058
190	30	16	57220049	58768261	1548212	6	17159491	18505569	1346078
191	33	16	67193834	68482591	1288757	6	25074671	26204933	1130262
192	12	16	69165194	69788843	623649	6	16181441	16748636	567195
193	23	16	70147529	73093597	2946068	6	13204581	15160499	1955918
194	25	16	74442529	79246564	4804035	6	9640139	13345308	3705169
195	2	16	81478775	81991899	513124	6	6994097	7278279	284182
196	53	16	84087368	90114181	6026813	6	98891	5142052	5043161
197	54	17	260118	4511614	4251496	12	48713393	52601851	3888458
198	45	17	4534197	7019019	2484822	12	52525591	54796676	2271085
199	2	17	7076750	7123369	46619	3	139811184	139840794	29610
200	3	17	7138347	7155810	17463	12	54835376	54848052	12676

201	5	17	7184986	7232712	47726	3	139903946	139956595	52649
202	78	17	7239848	12921504	5681656	12	54904770	60195261	5290491
203	17	17	15207128	17495022	2287894	12	61886697	63150665	1263968
204	8	17	18561742	19652256	1090514	12	62105943	62958954	853011
205	9	17	25621102	26708716	1087614	12	45668721	46601915	933194
206	2	17	26691378	26734215	42837	18	9613	42053	32440
207	36	17	26782770	28854610	2071840	12	46617996	48683128	2065132
208	21	17	29096406	31324895	2228489	12	44417875	45511888	1094013
209	33	17	32582237	36105237	3523000	12	39554707	42516128	2961421
210	3	17	36508111	36762183	254072	12	63386243	63584674	198431
211	162	17	37219556	45124520	7904964	12	20671168	23586953	2915785
212	4	17	45195069	45518678	323609	12	16621157	16905553	284396
213	71	17	45726842	50237377	4510535	12	23785575	28059896	4274321
214	55	17	52976748	60142643	7165895	12	31684057	39642292	7958235
215	47	17	60447579	67539472	7091893	12	11139492	16175744	5036252
216	80	17	70117161	75496678	5379517	12	4003723	9033210	5029487
217	46	17	76670130	81052864	4382734	12	2251	3520927	3518676
218	2	18	158383	912173	753790	6	98533727	98685782	152055
219	8	18	2537524	4455335	1917811	6	96171011	97088976	917965
220	6	18	8705659	9862553	1156894	6	91619320	92436829	817509
221	16	18	11882621	13915706	2033085	6	89575890	90833719	1257829
222	26	18	18529701	25757410	7227709	6	98713997	105518711	6804714
223	31	18	28709199	35146000	6436801	6	107663227	113867827	6204600
224	11	18	42260138	44236996	1976858	1	104402849	106509127	2106278
225	4	18	44497455	44775554	278099	1	309491900	309659930	168030
226	19	18	45357922	48744674	3386752	1	106901465	110614839	3713374
227	7	18	51679079	53332018	1652939	1	113952131	115215091	1262960
228	6	18	54264439	55289445	1025006	1	116724201	117509485	785284
229	11	18	55711599	58040001	2328402	1	178553488	180067851	1514363
230	14	18	59000815	61603345	2602530	1	175328930	177367336	2038406
231	2	18	63417488	64271375	853887	1	172944636	173664142	719506
232	5	18	66340925	67997436	1656511	1	168992150	170789634	1797484
233	2	18	70203915	70535381	331466	1	167023318	167199122	175804
234	7	18	71740588	73001905	1261317	1	164832382	166087358	1254976
235	4	18	74069644	74980858	911214	1	162981003	163953127	972124
236	3	18	76740275	77289325	549050	1	161403213	161853942	450729
237	7	18	77623668	78005429	381761	1	309824502	310328195	503693
238	76	19	281043	3047633	2766590	2	77474233	79297163	1822930
239	119	19	3094408	8933565	5839157	2	70947716	76090633	5142917
240	124	19	9004870	14889353	5884483	2	64748409	70553132	5804723
241	90	19	15052301	19774502	4722201	2	58562359	62932887	4370528
242	131	19	29698173	39692522	9994349	6	34521339	43484853	8963514
243	8	19	39693562	39900045	206483	14	143677755	143796256	118501
244	252	19	39936186	52730687	12794501	6	43804625	52352096	8547471
245	71	19	54135310	58951589	4816279	6	52438796	57741493	5302697
246	13	2	264140	3836122	3571982	3	140350715	142396888	2046173
247	3	2	6980701	7208417	227716	3	137582368	137704282	121914
248	18	2	8818975	11967535	3148560	3	133660275	135804369	2144094
249	23	2	15307032	21266945	5959913	3	125228051	130685391	5457340
250	89	2	23608088	33824449	10216361	3	112931652	122272144	9340492
251	26	2	36583069	40006407	3423338	3	106662022	110477211	3815189
252	38	2	42275160	49381676	7106516	3	97500987	104408929	6907942
253	18	2	53759810	56613308	2853498	3	89688777	92657477	2968700
254	2	2	58134786	58468507	333721	3	87936628	88122236	185608
255	29	2	60678302	65659771	4981469	3	80411733	85374450	4962717
256	34	2	66660584	71306935	4646351	3	74949400	79099762	4150362
257	2	2	71336814	71377231	40417	1	159918114	159968311	50197
258	48	2	71409869	75938115	4528246	3	70994304	74940726	3946422
259	2	2	79412357	80875905	1463548	3	66597266	66678001	80735
260	30	2	84650647	87089047	2438400	3	60577395	63174815	2597420
261	6	2	88367299	89050427	683128	3	59834045	60627147	793102
262	15	2	95691422	97218375	1526953	3	48092634	49210879	1118245
263	29	2	97371666	101613291	4241625	3	59227039	59522834	295795
264	15	2	101624079	103460352	1836273	3	53861567	55834903	1973336
265	10	2	105471969	107503564	2031595	3	50724567	52015651	1291084
266	9	2	108602979	110262207	1659228	3	49326029	50189368	863339
267	16	2	110300559	113594480	3293921	3	45180185	48041032	2860847

268	2	2	114462588	114720173	257585	15	22548114	22788058	239944
269	2	2	118572226	118771709	199483	15	26805697	26974155	168458
270	6	2	119599766	120130126	530360	15	28042612	28584893	542281
271	10	2	120197419	122525429	2328010	15	34655273	36697791	2042518
272	5	2	127413509	128186822	773313	15	28724895	29403883	678988
273	10	2	128293378	129076151	782773	15	65656864	66453016	796152
274	2	2	130908981	130956034	47053	14	54210056	54252347	42291
275	2	2	131099798	131132982	33184	15	35772237	35799078	26841
276	2	2	131513008	132111282	598274	15	156295907	156708966	413059
277	2	2	132222473	132291239	68766	14	54221716	54265646	43930
278	3	2	133174147	134326034	1151887	15	21862472	22413236	550764
279	12	2	134877554	136875735	1998181	15	18124941	20247084	2122143
280	3	2	138721590	139537918	816328	15	15214569	16096556	881987
281	4	2	143635067	145282147	1647080	15	7946301	9821863	1875562
282	9	2	148602086	151395525	2793439	15	1003620	3911122	2907502
283	3	2	152266397	153032506	766109	15	156931032	157424086	493054
284	4	2	153191751	154335322	1143571	15	66699447	67859763	1160316
285	27	2	157180944	163695240	6514296	15	70671007	76838573	6167566
286	11	2	165349322	167350757	2001435	15	79274182	81569418	2295236
287	69	2	169312372	180871840	11559468	15	84270149	95161817	10891668
288	9	2	182321929	184026408	1704479	15	96521134	98210606	1689472
289	7	2	186603355	188430487	1827132	15	100858019	102599793	1741774
290	24	2	189156396	193060435	3904039	15	103617254	108396912	4779658
291	15	2	196998290	199437305	2439015	15	111029414	113017866	1988452
292	36	2	200134223	204826300	4692077	15	114024846	118717532	4692686
293	28	2	206858445	213403565	6545120	15	120861246	126283983	5422737
294	2	2	213864429	215275225	1410796	15	127685830	128078907	393077
295	11	2	215275789	217347776	2071987	15	129321537	131506684	2185147
296	43	2	218664512	220440435	1775923	15	133026569	134635226	1608657
297	12	2	222282747	226518734	4235987	15	136746506	140641040	3894534
298	10	2	227599757	229046361	1446604	15	142019659	143341643	1321984
299	45	2	230222345	235405697	5183352	15	144455470	149243454	4787984
300	29	2	237073879	241518149	4444270	15	150464860	154622103	4157243
301	3	2	241653181	241836306	183125	15	157512036	157640428	128392
302	10	2	241938255	242576864	638609	15	154683000	155097235	414235
303	59	20	68351	4229721	4161370	17	35851523	39817216	3965693
304	16	20	4666882	6760910	2094028	17	14563017	17412718	2849701
305	11	20	7863628	10654608	2790980	17	18814427	22104506	3290079
306	7	20	12989627	14318262	1328635	17	24339952	25684519	1344567
307	46	20	16252749	25604811	9352062	17	28115797	35296195	7180398
308	100	20	29845467	37668366	7822899	17	39835620	47536983	7701363
309	103	20	39314488	53267710	13953222	17	48698399	62548050	13849651
310	37	20	54572496	58523735	3951239	17	63734700	67414765	3680065
311	7	20	59827559	61051026	1223467	17	68895866	69243438	347572
312	6	21	15481134	17252377	1771243	13	189333225	191009147	1675922
313	5	21	18884700	19858197	973497	13	192284841	193141213	856372
314	6	21	26957968	28338832	1380864	13	199001550	200449758	1448208
315	40	21	30244513	37357047	7112534	13	202557997	208304419	5746422
316	24	21	37406839	41174023	3767184	13	209715199	213582070	3866871
317	46	21	42539728	47706211	5166483	13	214758853	218632146	3873293
318	9	22	17565844	18614498	1048654	5	71739300	72527225	787925
319	50	22	19023795	22599927	3576132	14	54387653	55217799	830146
320	26	22	22838767	25005947	2167180	14	52311349	53377927	1066578
321	16	22	25115001	27026636	1911635	14	45567981	47098866	1530885
322	20	22	28144265	31521442	3377177	14	47987093	50062681	2075588
323	35	22	30476163	32651328	2175165	14	50118092	52149499	2031407
324	5	22	32783569	33454358	670789	5	11965791	12393033	427242
325	135	22	35462129	47571336	12109207	5	3524038	9974070	6450032
326	22	22	50296867	51001334	704467	5	110441061	111032812	591751
327	9	3	2140497	5261642	3121145	13	65908132	68154058	2245926
328	54	3	8543393	15140670	6597277	13	72215848	78986997	6771149
329	14	3	15247659	18486309	3238650	13	2379271	4870240	2490969
330	4	3	19920964	20227784	306820	13	7728155	8106544	378389
331	3	3	23933151	24536773	603622	13	12134512	12493717	359205
332	9	3	25215823	28390618	3174795	13	14021718	16535156	2513438
333	13	3	29322473	33911194	4588721	13	18080863	21240142	3159279
334	249	3	35680437	59035810	23355373	13	22755570	44783481	22027911

335	26	3	61547243	70017488	8470245	13	47722779	56474630	8751851
336	8	3	71003844	73674091	2670247	13	57861430	60441890	2580460
337	4	3	86987119	88042919	1055800	13	178816054	179719567	903513
338	3	3	93591881	93774512	182631	13	176513526	176780830	267304
339	11	3	97483365	98312567	829202	13	170521799	172821180	2299381
340	20	3	99357319	102198685	2841366	13	166070148	169091372	3021224
341	11	3	107241783	109056419	1814636	13	159270727	160875310	1604583
342	30	3	110607231	114866118	4258887	13	155134436	159012121	3877685
343	2	3	115342171	117716095	2373924	13	153563024	153883669	320645
344	55	3	118619404	125313934	6694530	13	144365411	150614293	6248882
345	4	3	125725198	126236616	511418	7	58628304	58820798	192494
346	25	3	126243126	129035120	2791994	13	79004139	80942657	1938518
347	6	3	129158968	129696781	537813	13	76112280	76787021	674741
348	6	3	130064359	132004254	1939895	13	583648	1881007	1297359
349	18	3	132036211	134370478	2334267	13	81003246	83308188	2304942
350	25	3	135684515	139396859	3712344	13	84409959	88111928	3701969
351	21	3	139654027	143767561	4113534	13	89149072	92390877	3241805
352	5	3	145787227	147228080	1440853	13	94176353	95454575	1278222
353	64	3	148415571	161221730	12806159	13	96933073	109559415	12626342
354	3	3	164696686	165555260	858574	13	112716748	113471750	755002
355	28	3	166958075	172429008	5470933	13	114708594	120052855	5344261
356	2	3	173114074	175523428	2409354	13	121518226	122650063	1131837
357	14	3	177990720	181432221	3441501	13	126138399	128980735	2842336
358	52	3	182511288	187463515	4952227	13	130081512	134684897	4603385
359	50	3	188665003	197770591	9105588	13	135947773	144300744	8352971
360	33	4	667369	3770251	3102882	8	523	2050084	2049561
361	7	4	4190530	5021199	830669	8	5246772	5824750	577978
362	20	4	5712924	8873543	3160619	8	2284188	4651143	2366955
363	2	4	9212383	9390709	178326	15	43115233	43392052	276819
364	2	4	9783258	10459034	675776	8	5694771	5949418	254647
365	3	4	13362978	13629347	266369	8	8643796	8791714	147918
366	18	4	15004298	18023499	3019201	8	9813426	12766041	2952615
367	3	4	20254883	21950422	1695539	8	15355493	15609158	253665
368	14	4	24519064	27027003	2507939	8	18853594	21105502	2251908
369	36	4	36067620	43032675	6965055	8	29136986	35531573	6394587
370	3	4	44624086	44728612	104526	8	36841354	36925325	83971
371	19	4	46037786	49064098	3026312	8	38019084	41120417	3101333
372	14	4	52709166	56239263	3530097	8	41159617	44156726	2997109
373	16	4	56262124	57976551	1714427	8	57440559	59139438	1698879
374	2	4	65140975	66536213	1395238	8	66284558	67540672	1256114
375	10	4	68424446	70653679	2229233	8	69756947	71084462	1327515
376	5	4	70796799	71117145	320346	8	148201997	148465034	263037
377	20	4	71384257	74609433	3225176	8	71220968	74086750	2865782
378	3	4	74718906	74853914	135008	5	18218054	18319181	101127
379	30	4	74861359	79465423	4604064	8	74159970	78205882	4045912
380	54	4	79697496	92523064	12825568	8	137999912	148031534	10031622
381	6	4	94750042	96470357	1720315	8	133740991	135105541	1364550
382	58	4	98105244	111563279	13458035	8	119920838	131215185	11294347
383	4	4	113739265	115599380	1860115	8	115965682	117671790	1706108
384	27	4	118954773	124324910	5370137	8	108000816	113071813	5070997
385	11	4	128544426	130034487	1490061	8	102652237	104118511	1466274
386	18	4	139936943	144478639	4541696	8	89340815	93503333	4162518
387	14	4	145567173	148993931	3426758	8	85707729	88941208	3233479
388	25	4	150999426	156138230	5138804	8	78338073	83721850	5383777
389	14	4	156587863	160281321	3693458	8	46456920	50684112	4227192
390	5	4	164031225	164441691	410466	8	55108280	55498954	390674
391	5	4	165997256	167025047	1027791	8	44856265	45947825	1091560
392	10	4	167654535	171012850	3358315	14	21067835	23503718	2435883
393	8	4	174089904	175899331	1809427	14	16572910	17927120	1354210
394	8	4	176554085	178363657	1809572	15	43983562	45623938	1640376
395	29	4	183065140	187179625	4114485	15	50897901	53941519	3043618
396	10	5	1050499	1887350	836851	16	85559014	86038151	479137
397	11	5	5140443	7906138	2765695	16	80297617	82792519	2494902
398	8	5	14143811	16936372	2792561	16	4222391	6492682	2270291
399	38	5	31193857	39462402	8268545	16	18351301	25907473	7556172
400	22	5	40759481	45696253	4936772	16	26857519	31324163	4466644
401	3	5	49692026	50690564	998538	16	31747651	32559002	811351

402	27	5	52083730	56560505	4476775	16	33941071	38569322	4628251
403	4	5	57749809	59817947	2068138	16	40067323	40654842	587519
404	7	5	59892739	61924409	2031670	16	42378460	44231575	1853115
405	12	5	63256183	65167553	1911370	16	45548623	47681209	2132586
406	2	5	65892176	66492627	600451	16	49018143	49229137	210994
407	11	5	67511548	69374349	1862801	16	50367103	51724239	1357136
408	8	5	70751442	72212560	1461118	16	51748146	53288151	1540005
409	62	5	72251808	83680611	11428803	2	83782139	94406585	10624446
410	5	5	85913721	88199922	2286201	2	96864357	98885272	2020915
411	6	5	89688078	90679176	991098	2	100651659	101583976	932317
412	7	5	92919043	94890711	1971668	2	103838947	105861092	2022145
413	4	5	94890778	95034415	143637	2	95606107	95732849	126742
414	7	5	95220802	96518964	1298162	2	106406384	107655950	1249566
415	2	5	98104354	98262240	157886	2	108896622	109061538	164916
416	2	5	99871009	100238970	367961	2	110536479	110825901	289422
417	7	5	101569690	102898494	1328804	2	111910757	113216199	1305442
418	28	5	108083523	115910630	7827107	2	118230893	125529074	7298181
419	6	5	118173017	120023027	1850010	2	128198591	129849716	1651125
420	9	5	121187650	122952739	1765089	2	130685730	132482857	1797127
421	15	5	125695824	129102425	3406601	2	134893210	137766861	2873651
422	148	5	130494720	142815077	12320357	2	138700892	151158579	12457687
423	40	5	144851362	149779870	4928508	2	153598273	158486259	4887986
424	12	5	150409506	151812929	1403423	16	76523080	78338637	1815557
425	11	5	152869175	154348971	1479796	16	74152533	75275676	1123143
426	30	5	155297354	161582542	6285188	16	66516842	72424942	5908100
427	4	5	162864575	162946342	81767	16	65096661	65197123	100462
428	33	5	166711804	173670504	6958700	16	54665137	60526928	5861791
429	60	5	174904065	180076624	5172559	2	79479773	83679567	4199794
430	33	6	485133	8435794	7950661	7	48090	5810301	5762211
431	19	6	9596343	14137149	4540806	7	7035743	10817827	3782084
432	14	6	15246527	18469105	3222578	7	12068700	14860574	2791874
433	7	6	19837617	22571892	2734275	7	16178089	18657420	2479331
434	153	6	24126350	33180499	9054149	7	20156906	29798122	9641216
435	158	6	33244917	47010099	13765182	7	34146955	48497395	14350440
436	3	6	47445525	47689757	244232	3	91976869	92084641	107772
437	18	6	49398073	52272575	2874502	7	49962812	52682127	2719315
438	8	6	52285106	53013627	728521	7	133966694	134516666	549972
439	10	6	53362139	55740362	2378223	7	29873202	31747437	1874235
440	5	6	56322785	57087078	764293	7	33091878	33751812	659934
441	16	6	69345259	74127292	4782033	1	55298662	59414361	4115699
442	5	6	74123238	74538040	414802	1	103132170	103557331	425161
443	6	6	75794042	76782395	988353	1	100869647	101823433	953786
444	8	6	79577189	81055987	1478798	1	96603030	98090751	1487721
445	11	6	82201156	84567234	2366078	1	92676866	95109056	2432190
446	7	6	84569362	86353510	1784148	1	59360910	61027408	1666498
447	25	6	87647024	91006627	3359603	1	61986692	65186708	3200016
448	8	6	96025419	97731093	1705674	1	71027087	72852209	1825122
449	11	6	99282580	102517958	3235378	1	74386223	77554767	3168544
450	47	6	105175968	112672498	7496530	1	80233232	88134607	7901375
451	3	6	114178541	114664209	485668	1	89851192	90023575	172383
452	9	6	116262693	116918838	656145	1	91721730	92403435	681705
453	15	6	116956781	119670926	2714145	1	47894684	50234411	2339727
454	2	6	121400640	121770873	370233	1	45543878	45819794	275916
455	7	6	122720691	123958238	1237547	1	43787655	44473486	685831
456	17	6	124125286	128841870	4716584	1	38212863	42198726	3985863
457	23	6	129897277	133138703	3241426	1	34439083	37147471	2708388
458	28	6	134210276	139695757	5485481	1	28295773	33478283	5182510
459	22	6	142379467	148058683	5679216	1	20431406	25823914	5392508
460	31	6	149539777	155635627	6095850	1	13558885	18903792	5344907
461	29	6	157099063	161695093	4596030	1	8311643	11894228	3582585
462	20	6	165693153	170716153	5023000	1	228294	4776214	4547920
463	28	7	192969	2883958	2690989	3	112279	2286576	2174297
464	18	7	3341080	6201195	2860115	3	3634288	5206824	1572536
465	2	7	6713376	6746554	33178	3	144201593	144241852	40259
466	2	7	6793740	6866401	72661	3	5209362	5289335	79973
467	6	7	7196565	8792593	1596028	9	85078137	86609535	1531398
468	7	7	10971578	12730559	1758981	9	88555977	90368637	1812660

469	2	7	13930853	15014402	1083549	9	91500795	92484202	983407
470	32	7	15239943	23684327	8444384	9	93546140	101924111	8377971
471	5	7	23749786	25021253	1271467	18	52262153	53339529	1077376
472	54	7	26240782	37873390	11632608	18	40252702	50979509	10726807
473	4	7	37723446	38671167	947721	9	119105762	119751221	645459
474	6	7	38762563	40900362	2137799	18	59557084	60730263	1173179
475	9	7	41724712	43846939	2122227	18	56025224	58009109	1983885
476	5	7	43906157	44109055	202898	18	53423634	53541606	117972
477	23	7	44111846	45961473	1849627	18	54851504	55984105	1132601
478	8	7	49813257	51384515	1571258	9	149509307	150715070	1205763
479	2	7	54610018	54827667	217649	9	153209620	153355773	146153
480	9	7	55861237	56184093	322856	3	17059600	17317249	257649
481	8	7	65338254	66704501	1366247	3	16269122	17016837	747715
482	3	7	69063905	71912148	2848243	3	14855655	16190523	1334868
483	32	7	72716514	76648340	3931826	3	9609480	11669064	2059584
484	5	7	76751751	77586818	835067	9	112759755	113208758	449003
485	3	7	79763271	80308593	545322	9	110040077	110592594	552517
486	5	7	81328322	84122040	2793718	9	106573372	108839094	2265722
487	10	7	86273230	87538856	1265626	9	101840273	103237238	1396965
488	9	7	87563458	90142716	2579258	9	74844064	77083335	2239271
489	33	7	91500243	97501854	6001611	9	78583165	84898643	6315478
490	62	7	97736197	102312088	4575891	3	5427990	9726972	4298982
491	29	7	102389418	108210110	5820692	9	113274169	119035097	5760928
492	2	7	108194987	108215294	20307	18	40219849	40232714	12865
493	26	7	110303110	117882785	7579675	18	29833815	37489340	7655525
494	20	7	120427376	124570037	4142661	18	24315318	27717509	3402191
495	39	7	126986844	131242976	4256132	18	18531185	21962952	3431767
496	14	7	132937829	135433594	2495765	18	14307713	16346564	2038851
497	57	7	136912088	143633372	6721284	18	6832520	12981639	6149119
498	4	7	143929004	144533488	604484	9	124255189	124854373	599184
499	6	7	148395006	148880116	485110	9	120110665	120463997	353332
500	6	7	148892577	149470568	577991	18	60758338	61140739	382401
501	31	7	150147718	152552463	2404745	18	4937318	6906676	1969358
502	17	7	153584182	158937649	5353467	18	663352	3448158	2784806
503	5	8	1449532	2113475	663943	15	37654773	38120615	465842
504	5	8	6264113	7274385	1010272	15	42685042	43357071	672029
505	2	8	8993765	9639856	646091	15	62933578	63437375	503797
506	13	8	9911778	11853821	1942043	14	14627025	16365270	1738245
507	5	8	12579403	15095848	2516445	17	710903	2067952	1357049
508	13	8	15274724	17942494	2667770	17	3407245	6333861	2926616
509	2	8	18248755	18942240	693485	17	13663699	14244810	581111
510	2	8	19261672	19709594	447922	17	9711448	10276260	564812
511	3	8	20002366	20161474	159108	14	4279273	4748277	469004
512	65	8	21547915	29120641	7572726	14	6064459	14342690	8278231
513	12	8	29190581	31031285	1840704	15	61331276	62923416	1592140
514	5	8	31496902	33457624	1960722	15	59482758	60041096	558338
515	23	8	36641842	39142430	2500588	15	54483937	56538254	2054317
516	19	8	39759794	42752433	2992639	17	10806299	13197225	2390926
517	6	8	48173167	49834299	1661132	4	86680287	87610320	930033
518	22	8	52730140	57906403	5176263	4	82110550	85388312	3277762
519	5	8	58907068	60031767	1124699	4	80758283	81524165	765882
520	4	8	61099906	62414204	1314298	4	78506791	79581189	1074398
521	2	8	63927638	63998612	70974	4	77238391	77303727	65336
522	22	8	65492814	69731257	4238443	4	72446096	75988707	3542611
523	22	8	70378859	75946793	5567934	4	66613220	71419852	4806632
524	2	8	77593454	77913280	319826	4	64727532	65030338	302806
525	14	8	79428374	82755101	3326727	4	60005444	63181989	3176545
526	13	8	85095022	87755903	2660881	4	55102478	57058815	1956337
527	5	8	90769975	91107703	337728	4	51177232	51539016	361784
528	4	8	91803778	92410378	606600	4	49029945	49861211	831266
529	16	8	94710789	96281429	1570640	4	44707161	46415639	1708478
530	43	8	97251626	105601417	8349791	4	35438363	43578498	8140135
531	14	8	107282473	110988076	3705603	4	29810607	33474850	3664243
532	7	8	117654369	119124092	1469723	4	22089714	23448887	1359173
533	11	8	119935796	121825513	1889717	4	19423081	20949267	1526186
534	20	8	123793633	126104082	2310449	4	15177104	17252337	2075233
535	2	8	130760442	131029375	268933	4	10801066	11113125	312059

536	13	8	132916335	136668965	3752630	4	6128156	9130605	3002449
537	6	8	140742586	142377367	1634781	4	1915228	3021011	1105783
538	48	8	144239331	146281416	2042085	4	8466	1445383	1436917
539	4	9	214854	1057552	842698	1	246943572	247752069	808497
540	28	9	2015342	7175648	5160306	1	240665778	245666198	5000420
541	2	9	7796490	10612723	2816233	1	238795293	239643734	848441
542	8	9	12685439	16061661	3376222	1	230579087	233679586	3100499
543	2	9	17134980	17797127	662147	1	228802214	229435694	633480
544	18	9	19049372	22452472	3403100	1	223545107	227420996	3875889
545	6	9	26840683	27297137	456454	1	218064215	218722657	658442
546	3	9	27524312	28670283	1145971	10	43166357	43587307	420950
547	32	9	32384618	34710121	2325503	10	36233267	38240593	2007326
548	46	9	34832642	38424444	3591802	1	263169264	267190286	4021022
549	21	9	70175707	75785309	5609602	1	247834116	253007803	5173687
550	15	9	77112281	80945009	3832728	1	254616170	257834732	3218562
551	2	9	84198598	84610171	411573	1	261797375	262087247	289872
552	15	9	85857905	88969369	3111464	10	33038181	35792553	2754372
553	2	9	90112143	90346308	234165	10	31867740	32028811	161071
554	7	9	90581356	92221470	1640114	14	137245	1422690	1285445
555	5	9	93372114	94877690	1505576	14	2406324	4012424	1606100
556	18	9	94972489	97063736	2091247	3	42962864	44960556	1997692
557	11	9	97321002	99382112	2061110	10	29514379	31900686	2386307
558	4	9	99401859	99801925	400066	10	76568591	77003512	434921
559	37	9	100000765	104500862	4500097	1	267252681	272030735	4778054
560	12	9	106856541	108538893	1682352	1	274584790	276818432	2233642
561	3	9	109625378	110252763	627385	1	278643318	279061593	418275
562	51	9	111616871	117880536	6263665	1	280407367	286608866	6201499
563	3	9	118916083	120177348	1261265	1	287904367	288860125	955758
564	14	9	123151147	125157982	2006835	1	292326416	294780705	2454289
565	2	9	125239220	125274022	34802	1	295976681	296617370	640689
566	2	9	125288637	125316493	27856	1	315136835	315276021	139186
567	2	9	125437315	125487204	49889	1	295594399	295732011	137612
568	148	9	125551150	138799074	13247924	1	297143794	308882338	11738544
569	31	9	139553308	140336268	782960	1	313727477	314164867	437390
570	2	X	3226606	3631649	405043	X	143912804	144164818	252014
571	11	X	6966961	11793870	4826909	X	4016053	9308558	5292505
572	45	X	12156585	20135035	7978450	X	10326535	17690099	7363564
573	16	X	21392536	25034065	3641529	X	18898825	22474529	3575704
574	3	X	26156460	26236387	79927	X	23661151	23774058	112907
575	2	X	27826107	27999566	173459	X	25345682	25399269	53587
576	6	X	30233677	30993201	759524	X	28294877	29007936	713059
577	4	X	35816459	36403431	586972	X	35174593	35706901	532308
578	8	X	37545012	38665790	1120778	X	37205479	38730034	1524555
579	10	X	39909068	41782716	1873648	X	39823458	41763430	1939972
580	8	X	43515467	45060146	1544679	X	43132739	44804691	1671952
581	64	X	46357162	51935364	5578202	X	45938667	50894068	4955401
582	27	X	53078273	57623906	4545633	X	51384537	56144179	4759642
583	8	X	62854847	65488709	2633862	X	56579850	58976654	2396804
584	49	X	66764465	75398039	8633574	X	60313335	69317294	9003959
585	15	X	76709648	80554046	3844398	X	70359020	74239655	3880635
586	3	X	82763269	83442933	679664	X	76477789	77276820	799031
587	4	X	84258832	85302566	1043734	X	78284663	79243045	958382
588	2	X	92925929	92967273	41344	X	92719586	92754360	34774
589	15	X	99546642	100641183	1094541	X	89912424	91037097	1124673
590	43	X	100870110	107682727	6812617	X	97041530	103136037	6094507
591	14	X	107975712	112084043	4108331	X	103860295	107012675	3152380
592	8	X	113818551	115594164	1775613	X	108566657	110139744	1573087
593	26	X	117031776	120009779	2978003	X	112411816	114992223	2580407
594	5	X	122318006	124097666	1779660	X	115591395	117279312	1687917
595	2	X	125683369	125955769	272400	X	118321510	118825784	504274
596	44	X	128580480	136659850	8079370	X	121756240	128557446	6801206
597	7	X	137713735	140271310	2557575	X	129613236	131905204	2291968
598	45	X	146993469	153402578	6409109	X	137792661	142349420	4556759
599	23	X	153665266	155012121	1346855	X	142640577	143693195	1052618
600	2	Y	14813160	15032390	219230	Y	502882	1132911	630029
