# Supplementary material for: The impact of the protein interactome on the syntenic structure of mammalian genomes
Source: PLoS One. 2017 Sep 14;12(9):e0179112. doi: 10.1371/journal.pone.0179112 (PMC5598925; doi:10.1371/journal.pone.0179112)
Supplement: S4 Table — The blocks are ordered by the location on the human genome. There are 499 blocks in total containing 15,773 orthologous protein-coding genes. (PDF) [file pone.0179112.s006.pdf]

Block No	Gene No	Human Chr	Human Start	Human End	Human Length	Dog Chr	Dog Start	Dog End	Dog Length
1	71	1	860260	4843850	3983590	5	55972417	59015062	3042645
2	40	1	5922871	10441661	4518790	5	59805955	63516385	3710430
3	220	1	10458649	29653325	19194676	2	71284674	85408755	14124081
4	48	1	31184124	33961995	2777871	2	67940747	70234420	2293673
5	101	1	33979609	43424530	9444921	15	463917	8192009	7728092
6	75	1	43629846	49242641	5612795	15	11914056	16987599	5073543
7	23	1	50513686	53293014	2779328	15	8597738	10975005	2377267
8	31	1	53308183	55681039	2372856	5	54086868	56066573	1979705
9	6	1	56960419	59012406	2051987	5	52231141	53006368	775227
10	37	1	58881056	67896098	9015042	5	43169994	51213976	8043982
11	5	1	68150744	68962904	812160	6	76848106	77396500	548394
12	7	1	70034081	72748417	2714336	6	74164157	76004571	1840414
13	26	1	74491699	79472403	4980704	6	68077795	72305780	4227985
14	23	1	84330711	87812788	3482077	6	60892486	63922412	3029926
15	40	1	89149905	95712781	6562876	6	54077495	59638888	5561393
16	23	1	99127236	104097854	4970618	6	47016178	51094551	4078373
17	53	1	107599267	111895635	4296368	6	40730080	44478160	3748080
18	18	1	111956936	114228545	2271609	17	62054619	63983370	1928751
19	48	1	114239453	120612240	6372787	17	51537804	57490239	5952435
20	23	1	145413095	147381393	1968298	17	57617385	58795843	1178458
21	75	1	149822643	153191793	3369150	17	59120172	61939063	2818891
22	113	1	153330330	157868046	4537716	7	40515318	43569673	3054355
23	93	1	157963063	163325554	5362491	38	19573978	23594205	4020227
24	8	1	164524821	166136206	1611385	38	17292263	18703810	1411547
25	83	1	166808681	179785333	12976652	7	20151401	31546905	11395504
26	43	1	179809102	186958113	7149011	7	13411792	19955853	6544061
27	10	1	192127587	193223031	1095444	38	5826209	6743512	917303
28	2	1	196194909	196716634	521725	38	2875854	3382572	506718
29	11	1	196946667	198726545	1779878	7	4156687	5752812	1596125
30	38	1	199996730	203155877	3159147	7	26316	3047521	3021205
31	42	1	203274619	206785904	3511285	38	71179	2871948	2800769
32	19	1	206808881	208417665	1608784	7	5816899	7049250	1232351
33	36	1	209757062	215410436	5653374	7	8240557	13093442	4852885
34	25	1	215740735	222924147	7183412	38	11005428	17005255	5999827
35	4	1	222988406	223568812	580406	38	23632557	23871773	239216
36	24	1	223711349	227175246	3463897	7	38086075	40451710	2365635
37	15	1	227916240	228700004	783764	14	467363	1109848	642485
38	52	1	228870824	238129359	9258535	4	2324208	10406578	8082370
39	24	1	240652873	247095280	6442407	7	32048183	37627869	5579686
40	4	1	247460714	247740992	280278	8	168413	549584	381171
41	18	1	247835320	248845629	1010309	14	1184208	2849348	1665140
42	5	1	248902716	249214145	311429	16	13633979	13822017	188038
43	6	10	225953	1779670	1553717	2	33756763	34570523	813760
44	3	10	3109712	3827473	717761	2	31654125	32254413	600288
45	23	10	4828820	8117161	3288341	2	28067975	30706801	2638826
46	43	10	11047259	18970568	7923309	2	18447643	25164128	6716485
47	29	10	21068902	27831143	6762241	2	6072586	12229221	6156635
48	11	10	28966271	32667726	3701455	2	14591247	17459703	2868456
49	9	10	32735068	35930362	3195294	2	1319268	4175222	2855954
50	4	10	38091751	38412276	320525	4	153460	436743	283283
51	18	10	43278249	46168228	2889979	28	1956700	4234052	2277352
52	10	10	46310876	48439138	2128262	4	34619615	35031077	411462
53	17	10	49272334	51732941	2460607	28	67356	1825612	1758256
54	5	10	52065360	54077802	2012442	26	35696876	37278085	1581209
55	20	10	60094735	65384883	5290148	4	10521607	15427228	4905621
56	88	10	67679719	79789303	12109584	4	18321039	27883436	9562397
57	13	10	80828792	82406316	1577524	4	28930798	29734014	803216
58	6	10	85899196	86278273	379077	4	32401616	32734060	332444
59	8	10	87359312	88951225	1591913	4	33514876	34740698	1225822
60	15	10	89264632	91174382	1909750	26	37586729	38918435	1331706
61	3	10	91061712	91163745	102033	4	63038	134065	71027
62	150	10	91174343	107024993	15850650	28	4318292	17491098	13172806
63	10	10	111624524	112840658	1216134	28	21267052	22370782	1103730
64	93	10	113909624	129924649	16015025	28	23261542	36939437	13677895
65	27	10	131265448	135382916	4117468	28	38203296	41157357	2954061
66	26	11	167784	790123	622339	18	25351515	25782909	431394
67	36	11	799180	3240043	2440863	18	45168052	47136486	1968434
68	172	11	3659733	22881972	19222239	21	26203333	44440917	18237584
69	10	11	26210829	28355054	2144225	21	47046020	48759266	1713246
70	2	11	30031288	30256808	225520	21	50115506	50281642	166136
71	40	11	30344598	36694823	6350225	18	31556038	36967499	5411461
72	4	11	43333513	43941816	608303	18	25859820	26355723	495903
73	54	11	44087475	48511332	4423857	18	41100902	45159295	4058393
74	2	11	49075266	49230222	154956	21	10382458	10463683	81225
75	2	11	49973943	50004071	30128	18	40499425	41209339	709914
76	75	11	55135360	59481337	4345977	18	37071666	41059797	3988131

77	11	11	59480929	60238233	757304	21	50309936	50733704	423768
78	234	11	60282886	71239227	10956341	18	47216500	55763036	8546536
79	73	11	71639747	79151992	7512245	21	19732924	26163553	6430629
80	8	11	82443053	85338966	2895913	21	15005602	16357249	1351647
81	21	11	85339629	89956532	4616903	21	10129931	13820314	3690383
82	26	11	92085262	96240738	4155476	21	4782389	8213266	3430877
83	2	11	100558384	101001255	442871	21	447197	820680	373483
84	83	11	101322295	115375675	14053380	5	17865290	30008523	12143233
85	70	11	116618886	121504387	4885501	5	12493152	16908278	4415126
86	58	11	122526383	126873355	4346972	5	7999874	11530167	3530293
87	17	11	128328656	133402414	5073758	5	2446084	6190184	3744100
88	10	11	133710526	134281812	571286	5	740979	1362320	621341
89	165	12	175931	16763528	16587597	27	30153135	44746429	14593294
90	22	12	18233803	22838646	4604843	27	24567392	28953337	4385945
91	29	12	24964295	30907885	5943590	27	17518966	22636850	5117884
92	2	12	31079362	31257725	178363	27	41478505	42101649	623144
93	11	12	31535157	34182629	2647472	27	15121987	17366084	2244097
94	177	12	39040624	55689016	16648392	27	59750	14837435	14777685
95	2	12	55820038	55846936	26898	3	31338938	31363485	24547
96	85	12	56075330	60176395	4101065	10	89177	3495594	3406417
97	53	12	62102040	73059422	10957382	10	5035601	14118059	9082458
98	7	12	74931551	75905416	973865	10	15361415	15992333	630918
99	20	12	76419227	83528649	7109422	15	19128105	25019828	5891723
100	7	12	85253492	86889092	1635600	15	26484385	27493140	1008755
101	8	12	88373816	90103077	1729261	15	29140309	30674528	1534219
102	28	12	91357456	97347129	5989673	15	31670414	36592199	4921785
103	35	12	98909290	104532067	5622777	15	37932891	42618306	4685415
104	20	12	104609557	108170421	3560864	10	31053744	34021549	2967805
105	26	12	108523248	110477568	1954320	26	17137382	18691280	1553898
106	44	12	110562140	115121969	4559829	26	8017187	11708211	3691024
107	40	12	117176096	121454305	4278209	26	13410185	16869102	3458917
108	54	12	121570622	126146917	4576295	26	4453646	7983848	3530202
109	31	12	128751948	133812681	5060733	26	14270	3112942	3098672
110	16	13	20248896	22278637	2029741	25	16630210	18359517	1729307
111	6	13	23755091	24896096	1141005	25	14650639	15403258	752619
112	4	13	24995064	25497018	501954	25	18392706	18780420	387714
113	67	13	25735822	41345309	15609487	25	521476	14287525	13766049
114	33	13	41506056	47371367	5865311	22	4548798	9670979	5122181
115	28	13	48510622	52603800	4093178	22	151837	3479763	3327926
116	3	13	52951305	53050485	99180	25	135617	224306	88689
117	4	13	53226844	53626196	399352	22	9699206	10075745	376539
118	7	13	72012098	74708394	2696296	22	25689557	28123013	2433456
119	4	13	75858808	76434004	575196	22	29109011	29620538	511527
120	12	13	77454312	80915086	3460774	22	30507649	33367878	2860229
121	37	13	95091741	103719196	8627455	22	45271735	52541974	7270239
122	2	13	107142093	107220512	78419	22	55567672	55639924	72252
123	35	13	107822318	115092796	7270478	22	56755710	61370523	4614813
124	2	14	20187174	20296531	109357	15	17225363	17351919	126556
125	3	14	20344391	20404842	60451	30	60403	427908	367505
126	35	14	20665495	22134238	1468743	15	17567892	18642119	1074227
127	69	14	23033805	25519503	2485698	8	2984501	4805102	1820601
128	41	14	29235050	39901704	10666654	8	8278048	17528249	9250201
129	7	14	44973545	45722743	749198	8	22004789	22668507	663718
130	2	14	47120222	48144157	1023935	8	23672388	24277159	604771
131	209	14	50065415	80330762	30265347	8	26229018	51608600	25379582
132	6	14	80663873	82000205	1336332	8	52685085	53824492	1139407
133	19	14	88304164	91976898	3672734	8	59266312	62344147	3077835
134	11	14	92047040	93582665	1535625	8	908999	2136332	1227333
135	33	14	93651296	97398059	3746763	8	62394771	65551402	3156631
136	69	14	99635624	105996539	6360915	8	67609102	72810277	5201175
137	2	15	22368478	22383507	15029	15	17248135	17337230	89095
138	4	15	22833395	23100005	266610	3	32003457	32339303	335846
139	3	15	23810454	23932450	121996	3	36314104	36417613	103509
140	7	15	25582381	28567298	2984917	3	31723022	35440245	3717223
141	8	15	29129629	32162992	3033363	3	37348109	39233349	1885240
142	18	15	32907345	35838394	2931049	30	745557	3142500	2396943
143	6	15	36871812	38857776	1985964	30	4129882	5903830	1773948
144	101	15	39873280	45968512	6095232	30	6770135	12005386	5235251
145	74	15	47476298	61521518	14045220	30	14003477	25360401	11356924
146	125	15	62144588	76020029	13875441	30	26602109	38513500	11911391
147	12	15	76135622	78113242	1977620	30	38735335	40189135	1453800
148	5	15	78276378	78592136	315758	3	58226813	58418744	191931
149	5	15	78729773	79012628	282855	13	38312372	38441192	128820
150	93	15	79051545	93632433	14580888	3	47044073	58222984	11178911
151	21	15	98462784	102264807	3802023	3	39346505	42664423	3317918
152	157	16	96407	5116111	5019704	6	36388166	40419589	4031423
153	45	16	8619502	16388668	7769166	6	27738221	33550988	5812767

154	58	16	18995256	25269252	6273996	6	20958395	25951172	4992777
155	96	16	27214807	31540124	4325317	6	16744861	19389567	2644706
156	6	16	46614466	46965209	350743	15	8238125	8491138	253013
157	22	16	46989299	51185278	4195979	2	64244574	67915238	3670664
158	56	16	52471917	58719008	6247091	2	58001215	63087737	5086522
159	85	16	66400533	69975644	3575111	5	79808300	82864030	3055730
160	28	16	70147529	73093597	2946068	5	76170042	78609987	2439945
161	24	16	74442529	79246564	4804035	5	72942927	76271270	3328343
162	13	16	80574631	82203831	1629200	5	69887187	71176593	1289406
163	73	16	83841448	90114181	6272733	5	63588208	68479086	4890878
164	57	17	5810	3867736	3861926	9	44564314	47727453	3163139
165	8	17	3907739	4511614	603875	5	30132570	30583456	450886
166	135	17	4534197	12921504	8387307	5	30600894	36978673	6377779
167	16	17	15133095	16557170	1424075	5	38806663	39908214	1101551
168	46	17	16832849	21323179	4490330	5	39936787	42860899	2924112
169	110	17	25621102	35969544	10348442	9	36378332	44532127	8153795
170	180	17	36481413	43511787	7030374	9	19104311	23826087	4721776
171	13	17	43699267	45518678	1819411	9	9127978	10249267	1121289
172	72	17	45726842	50237377	4510535	9	24004290	27862083	3857793
173	56	17	52976748	60142643	7165895	9	30025021	36336845	6311824
174	54	17	60447579	68176189	7728610	9	10583986	16475722	5891736
175	160	17	70642088	80275478	9633390	9	45995	7245119	7199124
176	8	18	158383	812547	654164	7	67035843	67598467	562624
177	8	18	2537524	3278282	740758	7	69231132	69894878	663746
178	33	18	5145284	13125051	7979767	7	71554548	78543416	6988868
179	5	18	13217497	13915706	698209	1	24388280	24881665	493385
180	27	18	18529701	24765281	6235580	7	61803624	67031827	5228203
181	34	18	28569974	34811481	6241507	7	53027372	58457593	5430221
182	2	18	40323192	40857615	534423	7	47555068	48064244	509176
183	16	18	42260138	45457515	3197377	7	43720229	46379498	2659269
184	12	18	46065417	48258194	2192777	7	78591511	80801406	2209895
185	5	18	48321491	48744674	423183	1	23799794	24093913	294119
186	45	18	51679079	61672278	9993199	1	13213440	21286926	8073486
187	3	18	63417488	65184217	1766729	1	10345379	11813909	1468530
188	6	18	66340925	67997436	1656511	1	8082389	9436811	1354422
189	2	18	70203915	70535381	331466	1	6114120	6382024	267904
190	10	18	71740588	73001905	1261317	1	4226210	5231417	1005207
191	4	18	74069644	74980858	911214	1	2729331	3387600	658269
192	11	18	76740275	78005429	1265154	1	509125	1428328	919203
193	457	19	281043	19774502	19493459	20	43618589	58096983	14478394
194	455	19	29698173	52730687	23032514	1	104975848	122037759	17061911
195	112	19	54024235	59084942	5060707	1	100742008	103724523	2982515
196	14	2	38814	3836122	3797308	17	89772	2307207	2217435
197	3	2	6980701	7208417	227716	17	4469593	4633483	163890
198	25	2	8865408	12882860	4017452	17	5980375	9252452	3272077
199	25	2	14772810	21366144	6593334	17	10659777	16002611	5342834
200	98	2	23608088	33824449	10216361	17	17945215	26688010	8742795
201	29	2	36583069	40838193	4255124	17	28857650	32307690	3450040
202	5	2	42275160	42984087	708927	17	33659290	34297179	637889
203	38	2	42989642	49381676	6392034	10	45363188	50938464	5575276
204	18	2	53759810	56613308	2853498	10	54672303	57122395	2450092
205	2	2	58134786	58468507	333721	10	58432010	58632360	200350
206	29	2	60678302	65659771	4981469	10	60588917	64815953	4227036
207	37	2	66660584	71222075	4561491	10	65764726	69301919	3537193
208	2	2	71336814	71377231	40417	3	38057223	38108131	50908
209	52	2	71409869	75938115	4528246	17	47629577	51413076	3783499
210	33	2	84650647	87089047	2438400	17	38342989	40429257	2086268
211	6	2	88326724	88927094	600370	17	37886601	38322193	435592
212	15	2	95691422	97218375	1526953	17	34319978	35000278	680300
213	45	2	97258907	103460352	6201445	10	40224566	45361150	5136584
214	10	2	105471969	107503564	2031595	10	36941370	38590302	1648932
215	11	2	108443388	110376563	1933175	10	34245321	35906868	1661547
216	25	2	110841447	114036527	3195080	17	35035854	37363225	2327371
217	2	2	114462588	114720173	257585	19	35099043	35308217	209174
218	23	2	118572226	122525429	3953203	19	28469474	32031133	3561659
219	16	2	127413509	129076151	1662642	19	22368942	23988413	1619471
220	3	2	131095814	131132982	37168	25	18998535	19035177	36642
221	5	2	131278770	132111282	832512	19	20348758	20847873	499115
222	2	2	132222473	132291239	68766	26	30369482	30427840	58358
223	16	2	133174147	136875735	3701588	19	35385836	38877889	3492053
224	3	2	138721590	139537918	816328	19	40655555	41318904	663349
225	4	2	143635067	145282147	1647080	19	45037354	46466691	1429337
226	20	2	148602086	153617688	5015602	19	49642632	53734669	4092037
227	2	2	154728426	155714863	986437	36	642898	1497142	854244
228	124	2	157180944	184026408	26845464	36	2546244	26173037	23626793
229	11	2	185463093	190044605	4581512	36	27454454	30677658	3223204
230	20	2	190306159	193060435	2754276	37	157467	2635499	2478032

231	151	2	196440701	220506702	24066001	37	5484005	26149312	20665307
232	12	2	222282747	224904036	2621289	37	27677566	29730784	2053218
233	4	2	225243415	226518734	1275319	25	37659677	38771407	1111730
234	56	2	227599757	235964358	8364601	25	39684358	46131191	6446833
235	54	2	237073879	242815975	5742096	25	47005515	51628676	4623161
236	82	20	68351	6760910	6692559	24	15197393	20757512	5560119
237	11	20	7863628	10654608	2790980	24	11657442	14252191	2594749
238	7	20	12989627	14318262	1328635	24	8364115	9597602	1233487
239	38	20	16252749	23476655	7223906	24	110236	6565250	6455014
240	12	20	23583047	25566153	1983106	23	1794	1734932	1733138
241	112	20	29891015	37668366	7777351	24	20771397	27392841	6621444
242	108	20	39314488	52790512	13476024	24	28770749	39878155	11107406
243	37	20	54572496	58609066	4036570	24	41260101	44608678	3348577
244	56	20	59827559	62926855	3099296	24	45794449	47672361	1877912
245	6	21	15481134	17252377	1771243	31	11139273	12691510	1552237
246	5	21	18884700	19858197	973497	31	14044703	14725973	681270
247	8	21	26957968	28338832	1380864	31	21089307	22289512	1200205
248	141	21	30244513	48085036	17840523	31	23897688	39838962	15941274
249	11	22	17565844	18614498	1048654	27	44798995	45725660	926665
250	47	22	18893541	22337213	3443672	26	29032190	31215133	2182943
251	25	22	22838767	24989175	2150408	26	27074456	28758276	1683820
252	15	22	25202236	27026636	1824400	26	18976904	20280368	1303464
253	23	22	28144265	31521442	3377177	26	21216846	23185614	1968768
254	35	22	30476163	32651328	2175165	26	23262226	25026600	1764374
255	6	22	32783569	34318829	1535260	10	29878086	31033207	1155121
256	158	22	35462129	47571336	12109207	10	19193656	29011623	9817967
257	32	22	48885272	51222091	2336819	10	16558706	18154222	1595516
258	10	3	2140497	5261642	3121145	20	12443916	14878553	2434637
259	46	3	8543393	12913415	4370022	20	5758437	9583120	3824683
260	15	3	12938719	15140670	2201951	20	3240980	5182490	1941510
261	13	3	15247659	18486309	3238650	23	25411414	27377687	1966273
262	6	3	19189946	20227784	1037838	23	23144405	23984735	840330
263	4	3	23933151	24536773	603622	23	19547145	19861170	314025
264	11	3	25215823	28579613	3363790	23	15777546	18377660	2600114
265	9	3	30647994	32612366	1964372	23	12234454	13945343	1710889
266	10	3	32726637	33911194	1184557	23	3252118	4199535	947417
267	55	3	35680437	43147568	7467131	23	5722509	12115744	6393235
268	7	3	43328004	44552128	1224124	23	1864664	2966102	1101438
269	160	3	44754135	52826078	8071943	20	37038213	43568517	6530304
270	3	3	52828784	52874278	45494	AAEX03020161.1	1469	42649	41180
271	35	3	52867137	59035810	6168673	20	31789154	37028638	5239484
272	33	3	61547243	73674091	12126848	20	18838303	29072250	10233947
273	6	3	86987119	88199035	1211916	31	118012	1077628	959616
274	3	3	93591881	93774512	182631	33	1624908	1763400	138492
275	35	3	96533425	102198685	5665260	33	4326594	8680870	4354276
276	2	3	105085753	105588396	502643	33	10983060	11470772	487712
277	10	3	107096188	108836989	1740801	33	12684585	14302947	1618362
278	33	3	110788918	114866118	4077200	33	15909408	18571299	2661891
279	2	3	115342171	117716095	2373924	33	19668237	20136897	468660
280	62	3	118619404	125313934	6694530	33	22640775	28586937	5946162
281	33	3	125725198	129024146	3298948	20	114558	3205407	3090849
282	8	3	129120164	129696781	576617	20	5219465	5756140	536675
283	73	3	130064359	143767561	13703202	23	27596435	39496407	11899972
284	5	3	145787227	147228080	1440853	23	41289828	42538734	1248906
285	54	3	148415571	158450485	10034914	23	43615159	52019047	8403888
286	15	3	158449987	161221730	2771743	34	25069775	27454449	2384674
287	3	3	164696686	165555260	858574	34	30284794	30977729	692935
288	33	3	166958075	175523428	8565353	34	32170825	39631299	7460474
289	16	3	178735011	181432221	2697210	34	12535080	14854028	2318948
290	72	3	182511288	193310900	10799612	34	15829063	25015885	9186822
291	9	3	194060494	195311076	1250582	33	30488508	31360330	871822
292	27	3	195343316	197770591	2427275	33	28651763	30427629	1775866
293	15	4	492989	1243741	750752	3	91385805	91849577	463772
294	32	4	1283639	3770251	2486612	3	60646132	62718181	2072049
295	14	4	4190530	6202318	2011788	3	69640750	71280761	1640011
296	20	4	6322305	8873543	2551238	3	58477559	60506799	2029240
297	6	4	9772777	11431389	1658612	3	68335170	69623158	1287988
298	2	4	13542454	13629347	86893	3	66464942	66546782	81840
299	15	4	15004298	17783135	2778837	3	62753440	65264049	2510609
300	2	4	17812525	18023499	210974	3	91175778	91322803	147025
301	3	4	20254883	21950422	1695539	3	88665407	89311605	646198
302	16	4	23756664	27027003	3270339	3	83560619	86285748	2725129
303	30	4	36067620	41270472	5202852	3	71395592	75833437	4437845
304	8	4	41361624	43032675	1671051	13	38518631	39882002	1363371
305	4	4	44175926	44728612	552686	13	40736878	41229180	492302
306	19	4	46037786	49064098	3026312	13	42200387	44782852	2582465
307	33	4	52709166	57976551	5267385	13	44823142	49207360	4384218

308	2	4	65140975	66536213	1395238	13	55272657	56401188	1128531
309	44	4	68337521	75254468	6916947	13	58013153	62880508	4867355
310	2	4	75669969	75975325	305356	15	63941688	64160884	219196
311	80	4	76404247	92523064	16118817	32	67694	14091961	14024267
312	6	4	94750042	96470357	1720315	32	16807336	18332959	1525623
313	56	4	98105244	111563279	13458035	32	19937903	30715919	10778016
314	12	4	113066553	116035032	2968479	32	31989841	34606491	2616650
315	11	4	118004718	120550146	2545428	32	36488434	38407235	1918801
316	19	4	120980577	124324910	3344333	19	16970914	19867779	2896865
317	2	4	125585207	126414087	828880	19	15155137	15908431	753294
318	11	4	128544426	130034487	1490061	19	11969864	13276496	1306632
319	22	4	138440072	144395721	5955649	19	9936	4983273	4973337
320	16	4	145567173	149365850	3798677	15	43432325	46710131	3277806
321	43	4	150999426	160281321	9281895	15	48118940	56351566	8232626
322	6	4	162305049	165305202	3000153	15	57985074	60036122	2051048
323	7	4	165997256	169239958	3242702	15	61088335	63741300	2652965
324	8	4	169418217	171012850	1594633	25	19147311	20944451	1797140
325	9	4	174252846	175899331	1646485	25	23938507	25442459	1503952
326	8	4	176554085	178363657	1809572	16	52314901	53844809	1529908
327	32	4	183065140	187647876	4582736	16	44113051	47883237	3770186
328	3	4	188916925	189068897	151972	16	42953884	43092536	138652
329	26	5	140373	3601517	3461144	34	9538498	12042856	2504358
330	11	5	5140443	7906138	2765695	34	6068583	8346222	2277639
331	8	5	9035138	10650308	1615170	34	3704756	5082084	1377328
332	4	5	13690440	14699820	1009380	34	122610	1040307	917697
333	7	5	14704910	17276943	2572033	4	86361608	88274129	1912521
334	41	5	31193857	39462402	8268545	4	69625850	75999226	6373376
335	24	5	40679600	45696253	5016653	4	64776433	68676418	3899985
336	3	5	49692026	50690564	998538	4	63638337	64459145	820808
337	9	5	52083730	54330398	2246668	4	60559232	62455650	1896418
338	16	5	54398476	56560505	2162029	2	42490492	44318494	1828002
339	4	5	57749809	59817947	2068138	2	45359712	46125553	765841
340	9	5	59892739	61924409	2031670	2	47295859	48938626	1642767
341	17	5	63256183	66492627	3236444	2	50005277	52660823	2655546
342	11	5	67511548	69374349	1862801	2	53456217	54637259	1181042
343	18	5	70751442	74162776	3411334	2	54654481	57361584	2707103
344	48	5	74323289	83680611	9357322	3	23417821	31218926	7801105
345	4	5	86563705	88199922	1636217	3	19778971	21177493	1398522
346	6	5	89688078	90679176	991098	3	17691961	18557804	865843
347	20	5	92953775	96518964	3565189	3	12548947	15673955	3125008
348	2	5	98104354	98262240	157886	3	11288582	11430311	141729
349	2	5	99871009	100238970	367961	3	9708161	10008958	300797
350	7	5	101569690	102898494	1328804	3	7696510	8828020	1131510
351	14	5	108083523	112258236	4174713	3	7992	3295999	3288007
352	2	5	112312399	112824527	512128	4	35256743	35592684	335941
353	13	5	112849380	115910630	3061250	11	3536963	6177817	2640854
354	5	5	118173017	118971517	798500	11	8264323	8860396	596073
355	10	5	121297656	122952739	1655083	11	11940144	13466798	1526654
356	93	5	125695824	138667360	12971536	11	15661886	26793507	11131621
357	56	5	138677276	143856944	5179668	2	34691026	39141265	4450239
358	19	5	144851362	147594700	2743338	2	40222441	42425609	2203168
359	47	5	147691982	151812929	4120947	4	56982495	60473800	3491305
360	11	5	152869175	154348971	1479796	4	54856186	56103537	1247351
361	30	5	155297354	161582542	6285188	4	48796947	53642810	4845863
362	4	5	162864575	162946342	81767	4	47709922	47806942	97020
363	72	5	166711804	176981542	10269738	4	35818873	43871097	8052224
364	38	5	177019159	180688119	3668960	11	234623	3513476	3278853
365	38	6	292097	8435794	8143697	35	678154	8327624	7649470
366	22	6	9596343	14137149	4540806	35	9527303	13511296	3983993
367	16	6	15246527	18469105	3222578	35	14477098	17222051	2744953
368	6	6	19837617	22297730	2460113	35	18357826	20704309	2346483
369	75	6	24126350	30181204	6054854	35	22216358	26461381	4245023
370	271	6	30294256	48036425	17742169	12	319406	16095432	15776026
371	40	6	49398073	57087078	7689005	12	17305774	24421842	7116068
372	4	6	63985856	66417118	2431262	12	26852156	27258754	406598
373	18	6	69345259	74538040	5192781	12	31616697	35838842	4222145
374	7	6	75794042	76782395	988353	12	36722874	37615194	892320
375	8	6	79577189	81055987	1478798	12	39857057	41225028	1367971
376	18	6	82201156	86353510	4152354	12	42269998	45645456	3375458
377	27	6	87647024	91296764	3649740	12	46550731	49739850	3189119
378	8	6	96025419	97731093	1705674	12	54170184	55714048	1543864
379	12	6	99282580	102517958	3235378	12	57104016	59992091	2888075
380	52	6	105175968	112672498	7496530	12	62229417	68756802	6527385
381	2	6	114254192	114664209	410017	12	70053181	70148119	94938
382	3	6	116262693	116566855	304162	12	71659397	71910400	251003
383	23	6	116782533	119670926	2888393	1	56894631	59273494	2378863
384	32	6	121400640	131604675	10204035	1	60667860	69483139	8815279

385	3	6	131894284	132068553	174269	12	97371	255247	157876
386	3	6	132269316	132834337	565021	1	25018443	25606609	588166
387	2	6	132909731	132945414	35683	AAEX03020335.1	34175	42220	8045
388	33	6	133002729	139695757	6693028	1	25678412	31524620	5846208
389	51	6	142379467	153452384	11072917	1	33772726	43264285	9491559
390	5	6	155054459	155777037	722578	1	44562567	45186510	623943
391	31	6	157099063	163999628	6900565	1	46370636	51956788	5586152
392	18	6	165693153	170102159	4409006	1	53414695	56717976	3303281
393	9	6	170102233	170893780	791547	12	71953729	72489391	535662
394	61	7	192969	6866401	6673432	6	11318953	16498806	5179853
395	7	7	7196565	8792593	1596028	14	22885720	24249219	1363499
396	5	7	11013499	12730559	1717060	14	25916867	27524751	1607884
397	100	7	13930853	37873390	23942537	14	28669824	48853758	20183934
398	12	7	37723446	40900362	3176916	18	9371868	12087626	2715758
399	8	7	41724712	43846939	2122227	18	6423999	8284321	1860322
400	3	7	45613739	45961473	347734	16	1021225	1167086	145861
401	3	7	47314752	48019178	704426	16	93713	432969	339256
402	4	7	48026745	48687092	660347	18	51035	353975	302940
403	8	7	49813257	51384515	1571258	18	1257377	2358902	1101525
404	5	7	54610018	55640681	1030663	18	5344269	6330466	986197
405	8	7	56019486	56184093	164607	6	445431	590706	145275
406	9	7	65338254	66704501	1366247	6	719498	1469960	750462
407	2	7	69063905	71178585	2114680	6	2132919	2831960	699041
408	41	7	72349936	76648340	4298404	6	5606042	7652431	2046389
409	8	7	76751751	79082890	2331139	18	17305494	18699053	1393559
410	10	7	79763271	84816171	5052900	18	19924450	24429758	4505308
411	54	7	86273230	97501854	11228624	14	12980180	22734459	9754279
412	80	7	97736197	102312088	4575891	6	7662680	11201626	3538946
413	35	7	102389418	108210110	5820692	18	12134354	17286192	5151838
414	2	7	108194987	108215294	20307	14	48875045	48886206	11161
415	24	7	110303110	117514193	7211083	14	50925878	56646219	5720341
416	11	7	119913722	123175131	3261409	14	58897164	60965138	2067974
417	8	7	123207064	124570037	1362973	14	10986871	11958359	971488
418	46	7	126078652	134144036	8065384	14	2991938	9719899	6727961
419	82	7	134331560	144533488	10201928	16	5257889	13418432	8160543
420	3	7	148395006	148725733	330727	16	1797420	2087075	289655
421	48	7	148766735	152552463	3785728	16	14141642	16555897	2414255
422	19	7	153584182	158937649	5353467	16	17573692	20987823	3414131
423	3	8	182137	495781	313644	25	37245728	37525780	280052
424	3	8	564746	1734738	1169992	37	30103210	30886347	783137
425	2	8	1772142	4852494	3080352	16	54309504	55827671	1518167
426	6	8	6264113	7740186	1476073	16	58306761	59011878	705117
427	5	8	8175258	9639856	1464598	16	34831826	36103987	1272161
428	15	8	9911778	11832108	1920330	25	25963267	28043507	2080240
429	18	8	12579403	17942494	5363091	16	36217906	41323077	5105171
430	4	8	18384811	19709594	1324783	16	21336735	22638086	1301351
431	4	8	19759228	20161474	402246	25	36764074	37103255	339181
432	68	8	21547915	29120641	7572726	25	28284386	35521771	7237385
433	12	8	29190581	31031285	1840704	16	33162514	34817289	1654775
434	5	8	33228342	33457624	229282	16	31115591	31264963	149372
435	50	8	36641842	43057998	6416156	16	22642609	28478440	5835831
436	5	8	48685669	49834299	1148630	29	26553	1156653	1130100
437	25	8	50822349	57906403	7084054	29	2523061	8215868	5692807
438	5	8	58907068	60031767	1124699	29	9000588	9842346	841758
439	5	8	61099906	62627155	1527249	29	10698640	12022539	1323899
440	2	8	63927638	63998612	70974	29	13141092	13201883	60791
441	46	8	65500320	76479078	10978758	29	14444860	23803458	9358598
442	2	8	77593454	77913280	319826	29	24665797	24945299	279502
443	17	8	79428374	82755101	3326727	29	26272055	28922783	2650728
444	15	8	85095022	88627447	3532425	29	31420777	33475169	2054392
445	12	8	90769975	93115514	2345539	29	35282548	37297859	2015311
446	23	8	94710789	98740998	4030209	29	38603942	41729971	3126029
447	48	8	98787285	110988076	12200791	13	40949	10450005	10409056
448	21	8	116420724	122653630	6232906	13	15022797	20341775	5318978
449	23	8	123793633	126450647	2657014	13	21377316	23346359	1969043
450	18	8	130760442	136668965	5908523	13	26907195	31574637	4667442
451	80	8	139142266	146281416	7139150	13	33399208	38223967	4824759
452	24	9	214854	5833117	5618263	1	89151933	93886762	4734829
453	9	9	5881596	10612723	4731127	11	26901020	29501600	2600580
454	29	9	12685439	22452472	9767033	11	33317645	41571634	8253989
455	2	9	23690102	24545944	855842	11	42503925	43195409	691484
456	10	9	26840683	28670283	1829600	11	45060428	46000069	939641
457	84	9	32384618	38424444	6039826	11	49698740	54594533	4895793
458	23	9	70971815	75785309	4813494	1	84743276	88994735	4251459
459	16	9	77112281	80945009	3832728	1	80377576	83529710	3152134
460	18	9	85594500	90346308	4751808	1	72215924	76468578	4252654
461	9	9	90581356	92221470	1640114	1	96744758	97629090	884332

462	5	9	93372114	94877690	1505576	1	94997230	96078806	1081576
463	19	9	94972489	96872138	1899649	1	97942204	99166337	1224133
464	14	9	97321002	99637905	2316903	1	70049818	72168485	2118667
465	33	9	100000765	104500862	4500097	11	54615071	58421761	3806690
466	13	9	106856541	108538893	1682352	11	60091547	61560530	1468983
467	3	9	109625378	110252763	627385	11	62401720	62886879	485159
468	52	9	111616871	117880536	6263665	11	64028093	69169707	5141614
469	3	9	118916083	120177348	1261265	11	70015167	70977896	962729
470	9	9	123151147	124132531	981384	11	73545859	74284185	738326
471	135	9	124329336	136039332	11709996	9	51274700	60919039	9644339
472	98	9	136205160	141019076	4813916	9	47813492	51215764	3402272
473	21	X	192989	3631649	3438660	X	239125	1982997	1743872
474	4	X	6966961	8700227	1733266	X	4248224	5601745	1353521
475	55	X	9754496	20285523	10531027	X	6499352	16103360	9604008
476	4	X	21392536	21903542	511006	X	17186806	17693480	506674
477	10	X	23352133	25034065	1681932	X	19096559	20556450	1459891
478	2	X	26156460	26236387	79927	X	21525932	21654736	128804
479	2	X	27826107	27999566	173459	X	22924486	22994762	70276
480	5	X	30233677	30993201	759524	X	25321459	25979729	658270
481	13	X	35816459	38665790	2849331	X	30061345	33549572	3488227
482	10	X	39909068	41782716	1873648	X	34620470	36296928	1676458
483	8	X	43515467	45060146	1544679	X	37677493	39039315	1361822
484	62	X	46433219	51935364	5502145	X	40210096	44682895	4472799
485	26	X	53078273	57623906	4545633	X	44872812	48380845	3508033
486	10	X	62567107	65488709	2921602	X	48808215	51100785	2292570
487	49	X	66764465	75005079	8240614	X	51969785	58879293	6909508
488	8	X	76709648	78427726	1718078	X	59739155	61420672	1681517
489	4	X	79591003	80554046	963043	X	61852894	62599412	746518
490	11	X	82763269	86925050	4161781	X	64427325	68110330	3683005
491	42	X	99546642	103499614	3952972	X	74184772	77612469	3427697
492	39	X	103810996	112084043	8273047	X	78654502	85789759	7135257
493	8	X	113818551	115594164	1775613	X	87342810	88968884	1626074
494	23	X	117031776	120009779	2978003	X	90138373	92832328	2693955
495	5	X	122318006	124097666	1779660	X	94731023	96338532	1607509
496	2	X	125683369	125955769	272400	X	97399564	98041159	641595
497	49	X	128580480	136659850	8079370	X	100677888	107833569	7155681
498	7	X	137713735	140271310	2557575	X	108658047	111066498	2408451
499	81	X	146993469	155173433	8179964	X	116248890	123783984	7535094
