# Supplementary material for: The impact of the protein interactome on the syntenic structure of mammalian genomes
Source: PLoS One. 2017 Sep 14;12(9):e0179112. doi: 10.1371/journal.pone.0179112 (PMC5598925; doi:10.1371/journal.pone.0179112)
Supplement: S5 Table — The blocks were defined based on the overlap of the four sets of pairwise blocks. There are 829 blocks in total containing 17,555 orthologous protein-coding genes. The blocks are ordered by the location on the human genome. The counts of cis- and trans-PPIs are given for the 192 blocks with minimum one high-confident cis- and one trans-PPI. “Obs/Theo (Cis) = NA” indicates that there was one gene in the block after removing tandem duplicates. “Obs/Theo (Trans) = NA” indicate that there were no genes in the block after removing tandem duplicates. (PDF) [file pone.0179112.s007.pdf]

Block No	Gene No	Chr.	Start	End	Obs. Cis	Obs. Trans	Obs/Theo (Cis)	Obs/Theo (Trans)	Norm. Cis/trans
1	7	1	879584	991496	0	339	0	0,00243323	0
2	70	1	1109264	3816857	2	1516	0,001209921	0,00131664	0,459473221
3	18	1	6161853	6761984	0	528	0	0,001658792	0
4	23	1	7979907	10441661	0	998	0	0,002389452	0
5	31	1	10458649	12092102	1	942	0,002849003	0,001754709	0,811816103
6	26	1	12123434	13498260	0	208	0	0,001492955	0
7	2	1	13801445	13944452	0	2	0	5,02E-05	0
8	224	1	14925200	29653325	52	7244	0,003022377	0,001974561	0,765328995
9	54	1	31184124	33961995	4	2896	0,003401361	0,002975784	0,571506676
10	3	1	33979609	34684732	0	75	0	0,00125584	0
11	44	1	35734568	38512450	1	1606	0,001282051	0,002020634	0,317239838
12	21	1	39303870	40563375	0	819	0	0,002287352	0
13	4	1	40627045	40783488	0	99	0	0,001243344	0
14	31	1	40810522	43424530	1	1163	0,002849003	0,002166377	0,657550103
15	81	1	43629846	48462567	5	2553	0,001754386	0,001693663	0,517926622
16	2	1	48688357	48937845	0	0	0	0	0
17	22	1	50883222	53293014	2	517	0,00952381	0,001237822	3,847001934
18	40	1	53308183	55681039	0	622	0	0,000920412	0
19	5	1	56960419	57431813	0	145	0	0,002427957	0
20	6	1	59120411	60539442	0	306	0	0,002562299	0
21	33	1	61330931	67600639	2	696	0,004597701	0,001167002	1,96987713
22	3	1	67632083	67896098	0	267	0	0,004470789	0
23	2	1	68150744	68299150	0	83	0	0,002084589	0
24	2	1	68564142	68915642	0	6	0	0,000150693	0
25	9	1	70034081	72748417	0	314	0	0,001753122	0
26	29	1	74491699	79472403	0	551	0	0,001108373	0
27	11	1	84330711	85462796	0	215	0	0,001080402	0
28	17	1	85464830	87812788	0	183	0	0,00083604	0
29	10	1	89149905	89738544	0	292	0	0,002933936	0
30	25	1	89829617	93828149	0	935	0	0,002136967	0
31	14	1	93913658	95712781	0	204	0	0,00073238	0
32	22	1	99127236	102462586	0	381	0	0,000912206	0
33	33	1	107682629	110208118	1	1055	0,002645503	0,00189511	0,697981394
34	26	1	110230436	111895635	1	562	0,005263158	0,00141277	1,862708372
35	21	1	111956936	114228545	0	1094	0	0,002750126	0
36	51	1	114239453	120439118	2	1531	0,002020202	0,001712672	0,589780892
37	23	1	145456236	147245484	0	54	0	0,000193865	0
38	113	1	149856010	153191793	3	1731	0,001081081	0,0011636	0,464541665
39	77	1	153330330	155532598	4	2477	0,002115283	0,002012879	0,525437302
40	55	1	155657751	157868046	4	1880	0,003401361	0,001931793	0,880363475
41	2	1	157963063	158154686	0	31	NA	0,001557085	0
42	106	1	158516918	163325554	9	1689	0,00362173	0,001199089	1,510200457
43	10	1	164524821	166136206	0	133	0	0,000742565	0
44	37	1	166808681	170708560	1	497	0,001680672	0,000714465	1,176174695
45	6	1	170904612	171311223	0	0	NA	0	0
46	47	1	171454651	179785333	1	1098	0,00116144	0,001315827	0,441334577
47	50	1	179809102	186958113	2	1791	0,002114165	0,002048955	0,515912898
48	10	1	192127587	193223031	0	248	0	0,002491836	0
49	11	1	196946667	198726545	0	84	0	0,000422111	0
50	39	1	199996730	202936408	3	1130	0,004267425	0,001496419	1,425878976
51	6	1	202976514	203155877	0	66	0	0,00066315	0
52	47	1	203274619	206785904	0	926	0	0,001226269	0
53	15	1	206808881	207534311	1	234	0,012820513	0,000904659	7,085826211
54	4	1	207627575	207968858	1	39	0,166666667	0,000489802	170,1367521
55	2	1	208057594	208417665	0	24	0	0,000602773	0
56	38	1	209757062	215410436	1	758	0,001501502	0,00103087	0,728268836
57	27	1	215740735	222924147	0	655	0	0,001432004	0
58	2	1	223282748	223537544	0	12	0	0,000301386	0
59	3	1	223967601	224349749	0	111	0	0,002787824	0
60	26	1	224363458	227175246	0	1195	0	0,003337467	0
61	12	1	227916240	228369958	0	498	0	0,002275126	0
62	7	1	228395831	228700004	1	492	0,166666667	0,006179041	13,48644986
63	40	1	228870824	235324772	0	1511	0	0,001901107	0
64	17	1	235324773	238129359	1	651	0,007352941	0,001925005	1,90985023
65	7	1	240652873	241965435	0	170	0	0,001220204	0
66	21	1	242011269	246831886	0	934	0	0,002471364	0
67	3	1	247460714	247615308	0	16	0	0,000401849	0
68	3	1	247835320	247887345	0	0	NA	NA	0
69	16	1	248004199	248437138	0	1	NA	5,02E-05	0
70	4	1	248550910	248617130	0	0	NA	0	0
71	7	2	264140	2335032	0	99	0	0,000710589	0
72	3	2	3192696	3523507	0	60	0	0,001004672	0
73	3	2	3642426	3836122	0	0	0	0	0
74	3	2	6980701	7208417	0	11	0	0,00018419	0
75	2	2	8865408	9143942	0	52	0	0,001306008	0
76	6	2	9346894	9771143	0	1042	0	0,008725214	0

77	6	2	9983483	10271545	0	120	0	0,001004823	0
78	15	2	10443015	11967535	0	456	0	0,00190974	0
79	26	2	15307032	21266945	1	831	0,004329004	0,001899272	1,139648158
80	27	2	23608088	26360323	0	746	0	0,001563076	0
81	57	2	26395960	29025806	5	2543	0,003246753	0,002287233	0,709755836
82	25	2	29025807	33824449	0	495	0	0,001082201	0
83	32	2	36583069	40006407	0	887	0	0,001487257	0
84	5	2	42275160	42984087	0	164	0	0,001647827	0
85	44	2	42989642	49381676	3	1860	0,004504505	0,002529576	0,890367384
86	19	2	53759810	55921045	1	1409	0,006535948	0,003935139	0,83045965
87	2	2	58134786	58468507	0	39	0	0,000979506	0
88	30	2	60678302	65659771	7	2540	0,016091954	0,004258887	1,889220744
89	9	2	67624451	68694390	0	190	0	0,001060807	0
90	25	2	68694693	71017775	4	1260	0,014492754	0,002640048	2,74478951
91	6	2	71035775	71222075	0	34	0	0,0002847	0
92	2	2	71336814	71377231	0	100	0	0,002511553	0
93	57	2	71409869	75938115	2	1778	0,001298701	0,001599174	0,406053789
94	36	2	84650647	87089047	2	1310	0,003787879	0,001997131	0,948330153
95	5	2	88367299	88927094	0	91	0	0,000914343	0
96	19	2	95691422	97039583	1	715	0,010989011	0,002566919	2,140505648
97	40	2	97371666	101613291	0	1116	0	0,001810956	0
98	17	2	101624079	103460352	0	90	0	0,000376922	0
99	10	2	105654441	106810795	0	263	0	0,001651844	0
100	3	2	108602979	108926371	0	4	NA	0,000200914	0
101	6	2	109065017	110262207	0	325	0	0,003265511	0
102	23	2	110841447	113594480	2	526	0,011695906	0,001391796	4,20173215
103	2	2	114462588	114720173	0	625	0	0,015697207	0
104	2	2	118572226	118771709	0	305	0	0,007660237	0
105	5	2	119599766	120130126	0	23	0	0,000231098	0
106	14	2	120197419	122525429	0	500	0	0,001933032	0
107	7	2	127413509	128186822	0	267	0	0,001916438	0
108	8	2	128293378	128785694	0	479	0	0,003008492	0
109	2	2	128848774	129076151	0	26	0	0,000653004	0
110	2	2	131099798	131132982	0	161	0	0,004043601	0
111	6	2	131513008	132111282	0	25	0	0,000313976	0
112	2	2	133174147	133429152	0	3	0	7,53E-05	0
113	13	2	134877554	136875735	0	496	0	0,001917568	0
114	3	2	138721590	139537918	0	7	0	0,000117212	0
115	4	2	143635067	145282147	0	155	0	0,001946649	0
116	9	2	148602086	151395525	0	224	0	0,001250635	0
117	5	2	152266397	153032506	0	237	0	0,002381311	0
118	2	2	153191751	153617688	1	238	1	0,005977496	83,64705882
119	32	2	157180944	163695240	3	717	0,007389163	0,001243606	2,970860729
120	11	2	165349322	167350757	0	78	0	0,000653135	0
121	81	2	169312372	180871840	5	2308	0,001956182	0,001615866	0,605304269
122	11	2	182321929	184026408	1	312	0,018181818	0,00142538	6,377884615
123	9	2	186603355	188430487	0	118	0	0,000846965	0
124	4	2	189156396	190044605	0	13	0	0,000326502	0
125	2	2	190306159	190448484	0	24	0	0,000602773	0
126	18	2	190611386	193060435	0	265	0	0,000887995	0
127	18	2	196998290	199437305	3	1765	0,019607843	0,004929397	1,988868522
128	44	2	200134223	204826300	3	1948	0,003658537	0,002391273	0,764976899
129	25	2	206858445	209719227	0	843	0	0,002119155	0
130	9	2	210288782	213403565	0	208	0	0,001161304	0
131	2	2	213864429	215275225	0	27	0	0,000678119	0
132	11	2	215275789	217347776	1	797	0,018181818	0,003641115	2,496737767
133	53	2	218664512	220440435	1	1338	0,000784314	0,001321078	0,296846039
134	13	2	222282747	224904036	0	435	0	0,001681738	0
135	4	2	225243415	226518734	0	173	0	0,002172712	0
136	11	2	227599757	229046361	0	62	0	0,000346158	0
137	62	2	230222345	235405697	2	1466	0,001850139	0,001570331	0,58909188
138	45	2	237073879	241518149	0	521	0	0,000689942	0
139	3	2	241653181	241836306	0	39	0	0,000653037	0
140	12	2	241938255	242576864	0	361	0	0,001649238	0
141	13	3	2140497	5261642	0	458	0	0,002092385	0
142	51	3	8543393	12913415	3	1993	0,002659574	0,002090466	0,636120037
143	18	3	12938719	15140670	1	660	0,007352941	0,001951618	1,883806818
144	9	3	15247659	16273499	0	183	0	0,001021724	0
145	3	3	16357352	18486309	0	44	0	0,000736759	0
146	5	3	19920964	20227784	0	412	0	0,004139663	0
147	4	3	23933151	24536773	0	496	0	0,006229278	0
148	8	3	25215823	27525911	0	117	0	0,000734851	0
149	3	3	27757440	28390618	0	68	0	0,001138628	0
150	10	3	30647994	32612366	0	223	0	0,001120603	0
151	11	3	32726637	33911194	0	414	0	0,002080402	0
152	7	3	35680437	37225180	0	176	0	0,00126327	0
153	56	3	37284668	43147568	2	2052	0,002114165	0,002347547	0,450292398

154	7	3	43328004	44552128	0	50	0	0,000502386	0
155	23	3	44754135	46402419	0	219	0	0,000733853	0
156	144	3	46448654	52329272	5	3226	0,000645161	0,001304423	0,247297662
157	15	3	52350335	52826078	0	476	0	0,001840247	0
158	28	3	52874279	57914895	1	701	0,002849003	0,001305787	1,090914079
159	12	3	57994127	59035810	0	675	0	0,002826917	0
160	10	3	61547243	64009658	0	393	0	0,001974874	0
161	6	3	64079543	67061634	0	102	0	0,0008541	0
162	9	3	68053359	70017488	0	85	0	0,000610102	0
163	10	3	71003844	73674091	0	144	0	0,000723618	0
164	4	3	86987119	88042919	0	47	0	0,000590274	0
165	3	3	93591881	93774512	0	12	0	0,000200934	0
166	17	3	97483365	98312567	0	174	0	0,001248914	0
167	22	3	99357319	102198685	0	792	0	0,001810137	0
168	11	3	107241783	108836989	0	106	0	0,000484264	0
169	35	3	110788918	114866118	1	518	0,001893939	0,000789705	1,19914334
170	2	3	115342171	117716095	0	43	0	0,001079968	0
171	64	3	118619404	125313934	6	1359	0,003072197	0,001086886	1,413302001
172	6	3	125725198	126236616	0	34	0	0,0002847	0
173	31	3	126243126	129024146	7	2493	0,017241379	0,004324004	1,99368231
174	5	3	129158968	129612419	0	37	0	0,000371766	0
175	9	3	130064359	132004254	0	298	0	0,001663791	0
176	20	3	132036211	134370478	0	579	0	0,001455505	0
177	29	3	135684515	139396859	1	979	0,003623188	0,002051276	0,88315495
178	22	3	139654027	143767561	1	797	0,004329004	0,001821564	1,18826552
179	7	3	145787227	147228080	0	26	NA	0,001305942	0
180	6	3	148508889	148939842	0	105	0	0,001318698	0
181	40	3	149086809	155658457	1	494	0,002463054	0,000856822	1,43731926
182	14	3	155755490	158450485	0	156	0	0,000603106	0
183	17	3	158450486	161221730	0	346	0	0,001337658	0
184	3	3	164696686	165555260	0	3	0	5,02E-05	0
185	6	3	166958075	167813763	0	62	0	0,00077866	0
186	14	3	168801287	170578169	0	414	0	0,0014863	0
187	14	3	170582664	172429008	0	404	0	0,002030151	0
188	16	3	178735011	181432221	1	1183	0,00952381	0,003964145	1,201243811
189	3	3	182511288	182833863	0	80	0	0,001339562	0
190	61	3	182840001	187463515	6	2787	0,004192872	0,00259927	0,806547921
191	17	3	188665003	193310900	0	124	0	0,000415515	0
192	11	3	194060494	195311076	0	126	0	0,000575634	0
193	32	3	195343316	197770591	1	1311	0,002016129	0,00206101	0,489111981
194	11	4	667369	1107350	0	108	0	0,000493401	0
195	2	4	1160720	1243741	0	134	0	0,003365481	0
196	36	4	1283639	3770251	0	765	0	0,00109973	0
197	8	4	4190530	5021199	0	220	0	0,001381771	0
198	4	4	5712924	6202318	0	59	0	0,000740983	0
199	19	4	6322305	8308838	1	356	0,008333333	0,001118428	3,725468165
200	3	4	9783258	10459034	0	53	0	0,00088746	0
201	2	4	13542454	13629347	0	22	0	0,000552542	0
202	17	4	15004298	17783135	0	208	0	0,000615055	0
203	2	4	17812525	18023499	0	83	0	0,002084589	0
204	3	4	20254883	21950422	0	56	0	0,000937694	0
205	15	4	24519064	27027003	0	901	0	0,003019184	0
206	31	4	36067620	41270472	0	803	0	0,001442439	0
207	9	4	41361624	43032675	0	98	0	0,000547153	0
208	3	4	44624086	44728612	0	6	0	0,000100467	0
209	20	4	46037786	49064098	0	203	0	0,00072879	0
210	17	4	52709166	56239263	0	233	0	0,000836493	0
211	20	4	56262124	57976551	0	976	0	0,002582496	0
212	22	4	68424446	70653679	0	116	0	0,000971329	0
213	9	4	70796799	71117145	1	36	0,047619048	0,000258396	92,14351852
214	20	4	71384257	74609433	0	243	0	0,000763421	0
215	4	4	74718906	74853914	0	0	NA	NA	0
216	7	4	74861359	75254468	0	2	0	3,35E-05	0
217	2	4	75669969	75975325	0	0	0	0	0
218	25	4	76404247	78354542	0	711	0	0,001489742	0
219	4	4	78432907	79465423	0	150	0	0,001883854	0
220	30	4	79697496	85887544	0	468	0	0,000840674	0
221	18	4	86396267	89152474	0	358	0	0,001124711	0
222	4	4	89183315	89444964	0	1	0	2,51E-05	0
223	4	4	89444965	90229161	0	7	0	8,79E-05	0
224	4	4	90645250	92523064	0	156	0	0,001959208	0
225	3	4	94750042	95264027	0	103	0	0,001724686	0
226	3	4	95373037	96470357	0	159	0	0,00266238	0
227	65	4	98105244	111563279	4	2156	0,002419843	0,001872477	0,646160857
228	4	4	113739265	115599380	0	163	0	0,002047121	0
229	11	4	118954773	120550146	0	87	0	0,000437186	0
230	4	4	120980577	122302214	0	80	0	0,001004722	0

231	16	4	122589110	124324910	1	676	0,00952381	0,002265226	2,102176669
232	10	4	128544426	130034487	0	243	0	0,001221106	0
233	9	4	139936943	141075338	0	297	0	0,001658208	0
234	9	4	141178440	142655140	0	78	0	0,000435489	0
235	2	4	144106070	144395721	0	67	0	0,001682741	0
236	17	4	145567173	148993931	0	513	0	0,001516939	0
237	27	4	150999426	156138230	3	1195	0,008547009	0,002225985	1,919826199
238	18	4	156587863	160281321	1	236	0,006535948	0,000659115	4,958125623
239	5	4	164031225	164441691	0	97	0	0,000974629	0
240	4	4	165997256	167025047	0	93	0	0,00116799	0
241	2	4	169013666	169239958	0	0	NA	0	0
242	8	4	169418217	171012850	0	131	0	0,000822782	0
243	9	4	174252846	175899331	0	226	0	0,001261801	0
244	9	4	176554085	178363657	0	115	0	0,000642067	0
245	34	4	183065140	187179625	1	862	0,002150538	0,001398785	0,768716164
246	10	5	1050499	1887350	0	213	0	0,001783561	0
247	7	5	5140443	6757161	0	190	0	0,001363757	0
248	3	5	7830491	7906138	0	3	0	5,02E-05	0
249	3	5	14143811	14699820	0	18	NA	0,000904114	0
250	6	5	14704910	16936372	0	92	0	0,000770364	0
251	48	5	31193857	39462402	1	1654	0,00116144	0,00198213	0,292977851
252	13	5	40759481	42887494	1	414	0,012820513	0,001600551	4,005032206
253	12	5	43039335	45696253	0	218	0	0,00091299	0
254	3	5	49692026	50690564	0	22	0	0,00036838	0
255	12	5	52083730	54330398	0	250	0	0,001395798	0
256	19	5	54398476	56560505	1	677	0,007352941	0,002001887	1,836502954
257	4	5	57749809	58155213	0	19	0	0,000238622	0
258	13	5	59892739	61924409	0	317	0	0,001448223	0
259	14	5	63256183	65167553	1	440	0,010989011	0,001579643	3,478321678
260	2	5	65892176	66492627	0	110	0	0,002762708	0
261	12	5	67511548	68890550	0	749	0	0,003421826	0
262	8	5	70751442	72212560	0	514	0	0,003228319	0
263	14	5	72251808	74162776	0	309	0	0,001194614	0
264	55	5	74323289	83680611	1	961	0,000886525	0,001007997	0,439745832
265	4	5	86563705	88199922	0	292	0	0,003667236	0
266	6	5	89688078	90679176	0	106	0	0,001331257	0
267	7	5	92953775	94890711	0	139	0	0,000997696	0
268	4	5	94890778	95034415	0	0	0	0	0
269	2	5	95220802	95769847	0	77	0	0,001933896	0
270	3	5	96271098	96518964	0	81	0	0,002034358	0
271	2	5	98104354	98262240	0	64	0	0,001607394	0
272	2	5	99871009	100238970	0	1	0	2,51E-05	0
273	7	5	101569690	102614361	0	87	0	0,000728497	0
274	5	5	108083523	110074657	0	111	0	0,001115298	0
275	13	5	110405760	112258236	0	351	0	0,001469997	0
276	3	5	112312399	112824527	0	297	0	0,004973125	0
277	17	5	112849380	115910630	0	178	0	0,000688159	0
278	4	5	118173017	118971517	0	5	0	6,28E-05	0
279	10	5	121297656	122952739	0	159	0	0,000798995	0
280	16	5	125695824	129102425	0	269	0	0,000845104	0
281	43	5	130494720	134063513	3	2289	0,004048583	0,002953667	0,685348693
282	22	5	134074191	137090039	0	696	0	0,001590726	0
283	19	5	137223657	138667360	3	1807	0,01754386	0,004781321	1,834624608
284	104	5	138677276	142815077	2	1574	0,001932367	0,001722583	0,560892277
285	19	5	144851362	147594700	0	383	0	0,001013418	0
286	25	5	147691982	149779870	1	687	0,003623188	0,001439455	1,258527941
287	13	5	150409506	151812929	0	364	0	0,001829146	0
288	11	5	152869175	154348971	0	453	0	0,002276382	0
289	33	5	156346293	161326975	2	693	0,004597701	0,001161972	1,978404737
290	5	5	162864575	162946342	0	203	0	0,002549483	0
291	25	5	167718656	171881527	0	974	0	0,001959266	0
292	8	5	172068269	172662360	0	248	0	0,001557632	0
293	5	5	172741716	173670504	0	30	0	0,000301432	0
294	40	5	174904065	176981542	2	972	0,00284495	0,001287185	1,105105105
295	2	5	177019159	177037348	0	20	0	0,000502311	0
296	37	5	177419236	180076624	3	2148	0,007389163	0,003725616	0,991669992
297	48	6	485133	8435794	2	970	0,003003003	0,001319188	1,138201604
298	31	6	9596343	14137149	0	249	0	0,00050088	0
299	16	6	15246527	18469105	0	575	0	0,001806449	0
300	4	6	20100935	21598847	0	87	0	0,001092635	0
301	64	6	24126350	26659980	0	299	0	0,000715878	0
302	40	6	27215480	29013017	0	2	0	1,67E-05	0
303	25	6	29141311	30181204	0	46	0	0,000231156	0
304	132	6	30294256	33180499	3	2125	0,000716675	0,001165497	0,307454428
305	7	6	33244917	33297046	0	121	0	0,001013197	0
306	73	6	33378176	39055519	5	2611	0,002194908	0,001935141	0,567118448
307	4	6	39071840	39290744	0	1	0	2,51E-05	0

308	101	6	39297766	47010099	16	3801	0,003583427	0,002019204	0,887336479
309	3	6	47445525	47689757	0	86	NA	0,004319654	0
310	8	6	49398073	49755053	0	128	0	0,001071811	0
311	13	6	49801970	52272575	0	251	0	0,001801595	0
312	3	6	52285106	52551386	0	8	0	0,000133956	0
313	3	6	52866077	53013627	0	51	0	0,000853971	0
314	10	6	53362139	55740362	0	45	0	0,000226131	0
315	6	6	56322785	57087078	0	215	0	0,001800308	0
316	7	6	70385694	72011973	0	39	0	0,000391861	0
317	3	6	73331520	73972919	0	5	NA	0,000251143	0
318	2	6	74078278	74127292	0	2	0	5,02E-05	0
319	4	6	74127293	74538040	0	1088	0	0,013664222	0
320	5	6	75962640	76782395	0	61	0	0,000612911	0
321	8	6	79577189	81055987	0	175	0	0,001099136	0
322	13	6	82201156	84567234	0	279	0	0,001274619	0
323	8	6	84569362	86353510	0	563	0	0,003536077	0
324	29	6	87795216	91006627	0	537	0	0,001038717	0
325	8	6	96025419	97731093	0	61	0	0,000383127	0
326	8	6	99282580	100063454	0	152	0	0,000954678	0
327	2	6	100832891	101329248	0	45	0	0,001130199	0
328	51	6	105175968	112672498	2	992	0,001632653	0,000998993	0,81714944
329	2	6	114254192	114664209	0	552	0	0,013863773	0
330	2	6	116262693	116566855	0	2	NA	0,000100457	0
331	5	6	116782533	116918838	0	19	0	0,000190907	0
332	15	6	117073363	119670926	0	211	0	0,000707045	0
333	2	6	121400640	121770873	0	63	0	0,001582278	0
334	5	6	122720691	123130865	0	48	0	0,000482291	0
335	2	6	123317116	123958238	0	6	0	0,000150693	0
336	18	6	124125286	128841870	0	207	0	0,000578122	0
337	8	6	129897277	131604675	0	130	0	0,000933097	0
338	5	6	131894284	132068553	0	159	0	0,001597589	0
339	3	6	132269316	132834337	0	51	0	0,000853971	0
340	5	6	133002729	133138703	0	247	0	0,004135899	0
341	33	6	134210276	139695757	2	1289	0,003787879	0,001965116	0,963780062
342	26	6	142379467	148058683	0	527	0	0,001060096	0
343	32	6	149539777	153452384	1	1213	0,003076923	0,002346301	0,65569662
344	4	6	155054459	155635627	0	116	0	0,001456847	0
345	17	6	157099063	159693141	0	495	0	0,001555117	0
346	14	6	160100096	161695093	0	999	0	0,004563957	0
347	5	6	165693153	166721936	0	17	0	0,000213503	0
348	6	6	166822852	167553184	0	87	0	0,001456774	0
349	7	6	168227602	170102159	0	155	0	0,001112539	0
350	6	6	170102233	170716153	0	86	0	0,000720123	0
351	30	7	855528	2883958	1	854	0,002298851	0,001431925	0,802713397
352	23	7	3341080	6201195	0	1853	0	0,00465812	0
353	2	7	6713376	6746554	0	0	NA	NA	0
354	2	7	6793740	6866401	0	0	NA	NA	0
355	8	7	7196565	8792593	0	193	0	0,00121219	0
356	2	7	11013499	12276886	0	47	0	0,00118043	0
357	2	7	12610203	12730559	0	64	0	0,001607394	0
358	2	7	13930853	15014402	0	14	0	0,000351617	0
359	18	7	15239943	19748710	0	254	0	0,000851135	0
360	9	7	19758933	22396763	0	105	0	0,000586235	0
361	5	7	22852251	23240630	0	65	0	0,000816337	0
362	5	7	23275586	23684327	0	280	0	0,002813363	0
363	5	7	23749786	25021253	0	129	0	0,001296157	0
364	51	7	26240782	33102409	0	752	0	0,000901186	0
365	14	7	33134409	36493400	1	181	0,012820513	0,000699758	9,1606814
366	3	7	36552456	37873390	0	40	0	0,000669781	0
367	4	7	37873391	38671167	0	157	0	0,001971767	0
368	2	7	38762563	39532694	0	30	0	0,000753466	0
369	5	7	39605975	40900362	0	86	0	0,000864104	0
370	6	7	41724712	43602938	0	153	0	0,001921531	0
371	4	7	45613739	45961473	0	54	0	0,000904205	0
372	8	7	49813257	51384515	0	109	0	0,000684605	0
373	2	7	54610018	54827667	0	24	0	0,000602773	0
374	7	7	56019486	56174269	0	669	0	0,005601889	0
375	11	7	65338254	66460635	0	61	0	0,000612911	0
376	29	7	72716514	74490064	0	134	0	0,000336853	0
377	15	7	75162621	76648340	1	1192	0,00952381	0,003994303	1,192174017
378	7	7	76751751	77586818	0	92	0	0,000660346	0
379	3	7	79763271	80308593	0	38	NA	0,001908685	0
380	2	7	81328322	82073114	0	42	0	0,001054852	0
381	2	7	82387442	84122040	0	0	NA	NA	0
382	10	7	86273230	87538856	0	90	0	0,00056527	0
383	5	7	87563458	88966346	0	128	0	0,001607556	0
384	5	7	89783689	90142716	0	35	0	0,000439566	0

385	11	7	91500243	92465908	0	351	0	0,001603552	0
386	28	7	92759368	97501854	0	445	0	0,00117747	0
387	30	7	97736197	99463718	0	756	0	0,001810046	0
388	72	7	99520892	102232891	6	1986	0,003172924	0,001613878	0,983012233
389	16	7	102389418	105208124	0	700	0	0,002345648	0
390	17	7	105208125	107643700	0	704	0	0,002211722	0
391	5	7	110303110	112130942	0	51	0	0,000512434	0
392	7	7	112405787	114659256	0	69	0	0,000577773	0
393	13	7	115575202	117514193	1	645	0,015151515	0,002701277	2,804510218
394	14	7	120427376	123175131	0	157	0	0,000717259	0
395	7	7	123207064	124405681	0	109	0	0,001095202	0
396	44	7	126986844	131242976	1	1350	0,001501502	0,001835983	0,408909465
397	4	7	132937829	134144036	0	65	0	0,001088394	0
398	13	7	134331560	135433594	0	283	0	0,001185211	0
399	72	7	136912088	143633372	1	896	0,00085034	0,000920684	0,461797805
400	7	7	143929004	144533488	0	11	0	0,00018419	0
401	3	7	148395006	148725733	0	409	0	0,006848512	0
402	3	7	148766735	148880116	0	0	NA	NA	0
403	7	7	148892577	149470568	0	6	0	7,54E-05	0
404	10	7	150147718	150558592	0	2	0	2,51E-05	0
405	25	7	150642049	152552463	0	571	0	0,001196403	0
406	20	7	154735397	157062066	0	219	0	0,000579474	0
407	2	8	1449532	1734738	0	28	0	0,000703235	0
408	3	8	1772142	2113475	0	8	0	0,000133956	0
409	3	8	6264113	6617184	0	38	0	0,000636292	0
410	7	8	6728097	6914256	0	6	NA	0,000301371	0
411	5	8	12579403	15095848	0	22	0	0,00022105	0
412	14	8	15274724	17942494	0	203	0	0,00072879	0
413	2	8	19261672	19709594	0	31	0	0,000778581	0
414	3	8	20002366	20161474	0	386	0	0,006463388	0
415	77	8	21547915	29120641	6	1615	0,002414487	0,001146554	1,052932331
416	13	8	29190581	31031285	0	486	0	0,001878907	0
417	5	8	33228342	33457624	0	75	0	0,00075358	0
418	10	8	37553269	37917883	0	336	0	0,001688442	0
419	16	8	37962760	39142430	0	396	0	0,001326967	0
420	18	8	39759794	42408151	0	581	0	0,001825299	0
421	2	8	42552519	42651535	0	0	MA	NA	0
422	2	8	42691817	42752433	0	28	0	0,000703235	0
423	5	8	48685669	49834299	1	961	0,1	0,009655865	5,178199792
424	14	8	52730140	55543394	0	307	0	0,001186882	0
425	10	8	56608983	57906403	0	585	0	0,002939698	0
426	6	8	58907068	60031767	0	114	0	0,001145441	0
427	4	8	61099906	62414204	0	129	0	0,001620115	0
428	2	8	63927638	63998612	0	19	0	0,000477195	0
429	7	8	65500320	67090960	0	75	0	0,000628014	0
430	18	8	67341263	69731257	0	658	0	0,002067206	0
431	31	8	70378859	75946793	0	936	0	0,001743533	0
432	2	8	77593454	77913280	0	34	0	0,000853928	0
433	20	8	79428374	82755101	0	385	0	0,001290106	0
434	9	8	85095022	86393693	0	77	0	0,000644762	0
435	7	8	86999552	87755903	0	215	0	0,001543199	0
436	5	8	90769975	91107703	0	215	0	0,002160261	0
437	6	8	91803778	92410378	0	8	0	6,70E-05	0
438	19	8	94710789	96281429	0	320	0	0,000946239	0
439	2	8	97251626	97349223	0	7	0	0,000175809	0
440	4	8	97505579	98740998	0	91	0	0,001142871	0
441	42	8	98787285	105601417	6	3226	0,006968641	0,003865992	0,901274704
442	15	8	107282473	110988076	0	416	0	0,001393985	0
443	7	8	117654369	119124092	0	530	0	0,003804164	0
444	12	8	119935796	121825513	0	279	0	0,001168459	0
445	23	8	123793633	126104082	0	408	0	0,001079568	0
446	2	8	130760442	131029375	0	30	0	0,000753466	0
447	13	8	132916335	134584183	0	136	0	0,000621319	0
448	2	8	135490031	136668965	0	25	0	0,000627888	0
449	9	8	140742586	142377367	0	323	0	0,001803371	0
450	76	8	144239331	146281416	2	1345	0,001397624	0,001254402	0,557087746
451	5	9	214854	1057552	0	78	0	0,000783723	0
452	24	9	2621834	5833117	1	513	0,008333333	0,001611667	2,58531514
453	8	9	5881596	6645650	0	178	0	0,001277625	0
454	11	9	12685439	16061661	0	209	0	0,000954822	0
455	2	9	17134980	17797127	0	136	0	0,003415712	0
456	32	9	19049372	22452472	0	591	0	0,002475123	0
457	6	9	26840683	27297137	0	85	0	0,00071175	0
458	3	9	27524312	28670283	0	39	0	0,000653037	0
459	43	9	32384618	34710121	1	1003	0,001422475	0,001328238	0,535474657
460	57	9	34832642	38424444	2	1594	0,00177305	0,001671953	0,530233055
461	14	9	70971815	73029540	0	202	0	0,000780945	0

462	9	9	74298282	75785309	0	324	0	0,001808954	0
463	16	9	77112281	80945009	0	222	0	0,000858266	0
464	15	9	86237964	88969369	1	1038	0,00952381	0,003478261	1,369047619
465	2	9	90112143	90346308	0	96	0	0,002411091	0
466	9	9	91003334	92221470	0	163	0	0,00091006	0
467	6	9	93372114	94877690	0	352	0	0,002947481	0
468	21	9	94972489	96872138	0	440	0	0,001106083	0
469	12	9	97321002	99382112	0	186	0	0,001038474	0
470	3	9	99401859	99637905	0	0	NA	0	0
471	39	9	100000765	104500862	0	1109	0	0,001550043	0
472	17	9	106856541	108538893	0	178	0	0,001277625	0
473	4	9	109625378	110252763	0	316	0	0,003968653	0
474	35	9	111616871	115653193	0	507	0	0,001019864	0
475	27	9	115800660	117880536	1	587	0,003623188	0,001229927	1,472927931
476	4	9	118916083	120177348	0	68	0	0,001138628	0
477	2	9	123151147	123476748	0	30	0	0,000753466	0
478	10	9	123514256	124132531	0	583	0	0,003255001	0
479	9	9	124329336	125157982	0	191	0	0,001370935	0
480	2	9	125239220	125274022	0	0	NA	NA	0
481	2	9	125288637	125316493	0	0	NA	NA	0
482	2	9	125437315	125487204	0	0	NA	NA	0
483	142	9	125551150	136039332	37	5875	0,004412642	0,002284748	0,965673759
484	36	9	136205160	138799074	0	1218	0	0,001914805	0
485	56	9	139553308	140336268	0	2053	0	0,00195074	0
486	9	10	225953	1779670	0	472	0	0,003952304	0
487	2	10	3109712	3827473	0	41	0	0,001029737	0
488	14	10	4828820	5884095	0	138	0	0,001386586	0
489	14	10	5903689	8117161	0	948	0	0,004330962	0
490	11	10	11047259	13141652	0	260	0	0,001187817	0
491	3	10	13628927	14504141	0	2	NA	0,000100457	0
492	11	10	14861249	15413061	0	96	0	0,000438578	0
493	18	10	15555948	18940551	0	767	0	0,002753604	0
494	17	10	21068902	23633774	0	113	0	0,000378655	0
495	16	10	23983675	27531059	0	368	0	0,001233141	0
496	13	10	28966271	32667726	0	418	0	0,001909644	0
497	2	10	33189247	33625190	1	292	1	0,007333735	68,17808219
498	2	10	35297479	35501886	0	130	0	0,003265019	0
499	2	10	35535953	35897863	0	0	NA	0	0
500	6	10	43278249	43904614	0	837	0	0,007008641	0
501	14	10	44051792	46168228	0	57	0	0,000318242	0
502	7	10	46310876	47174093	0	3	0	3,77E-05	0
503	7	10	51187938	51732941	0	0	NA	NA	0
504	8	10	52065360	54077802	0	84	0	0,000703376	0
505	19	10	60272900	65384883	1	664	0,005847953	0,001756944	1,664240295
506	61	10	68685764	74856732	2	1457	0,001298701	0,001310459	0,49551382
507	33	10	74870217	77161664	1	1332	0,002298851	0,0022334	0,514652584
508	3	10	79550549	79789303	0	108	0	0,002712477	0
509	3	10	80828792	81205383	0	130	0	0,002176789	0
510	5	10	81315608	81742370	0	5	NA	0,000251143	0
511	6	10	82031576	82406316	0	304	0	0,002545552	0
512	7	10	85899196	86278273	0	17	0	0,00012202	0
513	10	10	88195013	88951225	0	291	0	0,001462312	0
514	124	10	89264632	102124591	7	2853	0,001386139	0,001425994	0,486025237
515	4	10	102222798	102309763	0	21	0	0,000527426	0
516	49	10	102495360	105050108	2	1617	0,00177305	0,001696078	0,522691088
517	2	10	105148798	105206049	0	196	0	0,004922644	0
518	11	10	105253736	106214848	0	154	0	0,000859812	0
519	12	10	111624524	112840658	1	530	0,018181818	0,002421319	3,754528302
520	46	10	113909624	121215131	1	1125	0,001219512	0,001380997	0,441533333
521	17	10	121259340	124274424	0	458	0	0,001354304	0
522	2	10	124320181	124459338	0	16	0	0,000401849	0
523	37	10	124608594	129924649	1	981	0,001782531	0,001451647	0,61396843
524	35	10	131265448	135382876	0	461	0	0,0008281	0
525	15	11	167784	417455	0	310	0	0,001298288	0
526	15	11	537527	790123	0	245	0	0,000820977	0
527	9	11	799180	1036706	0	881	0	0,004918792	0
528	6	11	2920951	3187969	0	82	0	0,000823914	0
529	138	11	3659733	9550071	0	1712	0	0,00156773	0
530	40	11	9595228	17371521	3	1925	0,004504505	0,002617975	0,86030303
531	27	11	17407406	18814268	2	777	0,010526316	0,001953243	2,694574274
532	7	11	19138646	20530840	0	64	0	0,000459371	0
533	6	11	22214722	22851845	0	43	0	0,000540038	0
534	11	11	26210829	28355054	0	244	0	0,001226131	0
535	2	11	30031288	30256808	0	22	0	0,000552542	0
536	43	11	30344598	36619829	3	1825	0,004048583	0,002354933	0,859596251
537	4	11	43333513	43941816	0	146	0	0,001833618	0
538	57	11	44087475	48328704	1	1722	0,000784314	0,001700222	0,230650407

539	6	11	59244846	59481337	0	86	0	0,002159936	0
540	15	11	59481338	60238233	0	129	0	0,000540255	0
541	269	11	60282886	68708070	85	9626	0,003118007	0,002090707	0,745682518
542	12	11	68747490	70053496	0	209	0	0,001166887	0
543	3	11	71139239	71239227	0	52	0	0,001306008	0
544	83	11	71639747	79151992	4	2298	0,00170503	0,001678562	0,507884094
545	6	11	82534544	82997450	0	213	0	0,001783561	0
546	14	11	85339629	86666433	0	237	0	0,000916257	0
547	4	11	87846431	89322779	0	27	0	0,000339094	0
548	2	11	89864683	89956532	0	23	0	0,000577657	0
549	29	11	92085262	96123087	0	392	0	0,000730198	0
550	3	11	100558384	101001255	0	65	0	0,001088394	0
551	33	11	101322295	105969437	0	356	0	0,001052691	0
552	2	11	107197071	107436472	0	11	0	0,000276271	0
553	72	11	107461817	114466484	1	1065	0,000784314	0,001051531	0,372938967
554	82	11	116618886	121504387	12	2489	0,004210526	0,001651205	1,274985938
555	56	11	122526383	125551018	2	1496	0,003003003	0,002034541	0,738005051
556	17	11	125616188	126873355	0	215	0	0,000635754	0
557	19	11	128328656	130786404	0	358	0	0,001285255	0
558	10	11	133710526	134281812	0	96	0	0,000602954	0
559	17	12	175931	2802108	1	318	0,00952381	0,001065594	4,468778077
560	74	12	2904119	7371170	12	3814	0,00511509	0,002785916	0,918026466
561	25	12	7801996	9268825	0	172	0	0,000540364	0
562	20	12	9747147	10562356	1	30	0,015151515	0,000125641	60,2969697
563	7	12	10758612	10978957	0	310	0	0,005190804	0
564	12	12	11060525	11339543	0	0	NA	NA	0
565	39	12	11802788	16430619	1	1094	0,001680672	0,001572686	0,534331649
566	8	12	18233803	20906320	0	107	0	0,000895967	0
567	13	12	21200113	22218608	1	428	0,015151515	0,001792475	4,22642311
568	35	12	24964295	30907885	1	732	0,002016129	0,00115077	0,875991539
569	2	12	31079362	31257725	0	30	0	0,000753466	0
570	2	12	32112304	32536567	0	63	0	0,001582278	0
571	4	12	32552463	33049774	0	181	0	0,002273184	0
572	2	12	33527173	34182629	0	16	0	0,000401849	0
573	54	12	39040624	49076021	1	1156	0,001010101	0,001293173	0,390551274
574	152	12	49082247	54982443	16	4407	0,002821372	0,002079832	0,678269134
575	6	12	55341802	55689016	0	2	0	5,02E-05	0
576	2	12	55820038	55846936	0	0	NA	NA	0
577	102	12	56075330	60176395	25	4423	0,006839945	0,002594342	1,318242875
578	30	12	62654119	68059186	0	725	0	0,001302327	0
579	35	12	68548548	73059422	3	1918	0,005347594	0,002838185	0,942079818
580	7	12	74931551	75905416	0	145	0	0,00145692	0
581	4	12	76419227	76953589	0	431	0	0,005412941	0
582	3	12	77157368	77459360	0	37	0	0,000619548	0
583	14	12	79257773	83528649	0	475	0	0,00217005	0
584	6	12	85253492	86889092	0	17	0	0,00014235	0
585	10	12	88373816	90103077	0	150	0	0,000753769	0
586	4	12	91357456	91576900	0	68	0	0,001707856	0
587	33	12	92536286	97347129	0	953	0	0,001452875	0
588	3	12	98987369	99129204	0	365	0	0,006111753	0
589	35	12	100422233	104532067	1	1939	0,001680672	0,002787421	0,30147438
590	8	12	104609557	105630016	0	109	0	0,000684605	0
591	13	12	106457118	108155049	0	480	0	0,001855711	0
592	27	12	108523248	110477568	1	881	0,002849003	0,001641082	0,868025845
593	20	12	110562140	112194903	0	1207	0	0,003034188	0
594	28	12	112204691	115121969	0	1315	0	0,00264521	0
595	20	12	117176096	120315095	0	768	0	0,001930618	0
596	25	12	120427673	121454305	5	2419	0,021645022	0,005528688	1,957518849
597	64	12	121570622	126146917	0	1420	0	0,00132435	0
598	3	12	128751948	129469509	0	44	0	0,001105083	0
599	10	12	130647004	132336328	0	613	0	0,003850116	0
600	18	12	132379196	133532892	0	642	0	0,00201694	0
601	3	13	20248896	20437776	0	77	0	0,001933896	0
602	12	13	20977806	22278637	0	194	0	0,000812477	0
603	9	13	23755091	24896096	0	86	0	0,000720123	0
604	4	13	24995064	25497018	0	93	0	0,00116799	0
605	7	13	25735822	26625198	0	115	0	0,001155489	0
606	42	13	26706253	34540695	0	1934	0	0,002317678	0
607	20	13	35516424	39460074	0	441	0	0,001231651	0
608	2	13	47127303	47371367	0	29	0	0,00072835	0
609	6	13	48627459	49783888	0	446	0	0,004481286	0
610	6	13	49822047	50159719	0	49	0	0,000615392	0
611	12	13	50202435	52334135	0	143	0	0,000598888	0
612	3	13	52436117	52603800	0	14	0	0,000234423	0
613	2	13	52951305	53050485	0	26	0	0,000653004	0
614	4	13	53226844	53626196	0	75	0	0,00125584	0
615	6	13	72012098	73651676	0	130	0	0,001306204	0

616	4	13	75858808	76434004	0	160	0	0,002009444	0
617	12	13	77522632	80915086	0	207	0	0,000866921	0
618	4	13	95091741	95953687	0	46	0	0,000577715	0
619	27	13	96085858	103054124	0	641	0	0,001289414	0
620	8	13	103249353	103528345	0	79	0	0,000661509	0
621	15	13	107822318	111996596	0	368	0	0,001541193	0
622	21	13	113139325	114898086	0	431	0	0,001203722	0
623	49	14	20691791	22134238	0	1339	0	0,002172823	0
624	91	14	23033805	25103473	4	1750	0,002048131	0,001399596	0,731686636
625	44	14	31028329	39578850	0	1298	0	0,001593363	0
626	7	14	39583427	39901704	0	191	0	0,002398774	0
627	8	14	44973545	45722743	0	116	0	0,00072857	0
628	3	14	47120222	48144157	0	24	0	0,000602773	0
629	25	14	50087489	52197445	2	943	0,008658009	0,002155251	2,008584558
630	5	14	52292913	52535712	0	231	0	0,002901135	0
631	40	14	52734431	58764857	2	1360	0,003174603	0,001900864	0,835042017
632	29	14	58764858	62568431	0	583	0	0,001085983	0
633	6	14	63670832	64804830	0	228	0	0,001909164	0
634	141	14	64854749	78401355	14	3743	0,001896762	0,001550451	0,611680647
635	6	14	80663873	82000205	0	211	0	0,001766814	0
636	23	14	88304164	91976898	0	638	0	0,001527525	0
637	13	14	92047040	93582665	0	408	0	0,001577354	0
638	45	14	93651296	97398059	0	546	0	0,000832392	0
639	14	14	99635624	100996640	0	349	0	0,001252944	0
640	48	14	102228135	105647660	7	2963	0,006763285	0,003242703	1,042846964
641	2	15	23810454	23891175	0	4	0	0,000100462	0
642	4	15	25582381	27194354	0	81	0	0,001356307	0
643	2	15	28000021	28567298	0	31	0	0,000778581	0
644	4	15	29129629	30261068	0	170	0	0,002135035	0
645	2	15	32933877	33026870	0	50	0	0,001255777	0
646	9	15	34260921	34659479	0	44	0	0,00024566	0
647	6	15	35043233	35838394	0	763	0	0,007666415	0
648	6	15	36871812	38857776	0	59	0	0,000494038	0
649	115	15	39873280	45968512	5	2645	0,000990099	0,001322031	0,374461248
650	25	15	47476298	51298097	1	614	0,003333333	0,001235102	1,349416395
651	4	15	51348795	51915030	0	32	0	0,000401889	0
652	13	15	51973550	53083273	0	288	0	0,001607959	0
653	13	15	55473004	57210769	0	395	0	0,001527095	0
654	21	15	57884139	61521518	0	807	0	0,002135322	0
655	32	15	62144588	65321977	0	787	0	0,001465983	0
656	51	15	65409717	72410918	5	2281	0,004432624	0,00239255	0,926338765
657	61	15	72452148	76020029	4	2005	0,002795248	0,001869945	0,747414483
658	12	15	76196200	78113242	1	377	0,015151515	0,001578886	4,79816735
659	7	15	78276378	78592136	0	347	0	0,002490651	0
660	6	15	78729773	79012628	0	260	0	0,004353577	0
661	10	15	79051545	80216096	0	164	0	0,001030047	0
662	39	15	80351910	84708594	0	393	0	0,000658954	0
663	2	15	85923802	86338261	0	28	0	0,000703235	0
664	6	15	88402982	89199714	1	211	0,066666667	0,001766814	18,86635071
665	38	15	89346674	91506349	0	901	0	0,001333266	0
666	6	15	92396925	93632433	0	102	0	0,0008541	0
667	20	15	98980391	101817705	0	198	0	0,000622047	0
668	4	15	101821715	102264807	0	333	0	0,004182156	0
669	9	16	103010	231180	0	76	0	0,000954486	0
670	115	16	238968	2827298	13	3308	0,002574257	0,001653414	0,778467352
671	2	16	2867164	2888967	0	8	NA	0,000401828	0
672	18	16	2902728	3199964	0	243	0	0,000872394	0
673	23	16	3254247	4389598	0	868	0	0,002296728	0
674	23	16	4390252	5116111	0	399	0	0,000911925	0
675	32	16	8619502	12061925	0	1274	0	0,002002842	0
676	5	16	14014014	14763093	0	179	0	0,002248066	0
677	51	16	18995256	23392620	0	394	0	0,000495722	0
678	7	16	23399814	23681195	0	187	0	0,001342224	0
679	9	16	23847322	25269252	0	302	0	0,001686124	0
680	132	16	27214807	31520630	12	3422	0,002805049	0,001856774	0,755355373
681	5	16	46690054	46965209	0	243	0	0,002441598	0
682	22	16	46989299	51185278	0	907	0	0,00228004	0
683	20	16	55357672	56687116	0	680	0	0,003106597	0
684	32	16	57220049	58719008	0	1192	0	0,001934283	0
685	53	16	67193834	68482591	1	1150	0,000816327	0,001158107	0,352440106
686	16	16	69165194	69788843	0	367	0	0,001537005	0
687	38	16	70147529	73093597	2	1289	0,004926108	0,002235716	1,101684584
688	34	16	74442529	79246564	0	748	0	0,001446853	0
689	2	16	81478775	81991899	0	77	0	0,001933896	0
690	86	16	84087368	90111383	1	1662	0,000316456	0,001047655	0,15103048
691	69	17	260118	3867736	10	3723	0,007256894	0,003537558	1,02569268
692	9	17	3907739	4511614	0	570	0	0,004091271	0

693	60	17	4534197	7019019	3	1874	0,00244898	0,00188721	0,648835842
694	2	17	7076750	7123369	0	205	NA	0,010296851	0
695	4	17	7138347	7155810	0	56	0	0,000703306	0
696	6	17	7184986	7232712	0	331	0	0,002771637	0
697	43	17	7239848	7923657	5	2219	0,007507508	0,003017812	1,243866106
698	57	17	7942335	12921504	1	847	0,001349528	0,001092947	0,617380227
699	2	17	15207128	15408394	0	6	0	0,000150693	0
700	9	17	16945859	17495022	0	277	0	0,001546544	0
701	22	17	18561742	19652256	0	237	0	0,000850853	0
702	16	17	25621102	26708716	0	276	0	0,001067034	0
703	42	17	26782770	28854610	0	1517	0	0,001817951	0
704	27	17	29096406	31324895	0	769	0	0,001611268	0
705	65	17	32582237	35969544	0	483	0	0,001734017	0
706	3	17	36508111	36762183	0	0	NA	NA	0
707	220	17	37219556	43511787	33	5387	0,00316092	0,001879672	0,840816626
708	15	17	43699267	45124520	0	538	0	0,003861586	0
709	5	17	45195069	45518678	0	124	0	0,002076322	0
710	43	17	45726842	47592379	4	2367	0,0056899	0,003134535	0,907614839
711	37	17	47676246	50237377	0	1026	0	0,001518237	0
712	69	17	52976748	60142643	3	2404	0,001488095	0,001892699	0,39311465
713	69	17	60447579	67539472	3	2587	0,001753361	0,002208829	0,396898285
714	99	17	70642088	75496678	7	3520	0,001709402	0,001951729	0,437919823
715	77	17	76670130	80275478	21	3666	0,009218613	0,002717053	1,696435987
716	4	18	158383	581524	0	173	0	0,002172712	0
717	7	18	2571510	3278282	0	182	0	0,001828686	0
718	7	18	8705659	9862553	0	213	0	0,001528843	0
719	14	18	11882621	13125051	0	646	0	0,002319203	0
720	5	18	13217497	13915706	0	57	0	0,000954438	0
721	28	18	18529701	24765281	0	1053	0	0,001891517	0
722	34	18	28709199	34811481	1	376	0,003076923	0,000727295	2,115319149
723	11	18	42260138	44236996	0	840	0	0,003837562	0
724	9	18	44497455	44775554	0	33	0	0,000552569	0
725	15	18	46065417	48258194	1	378	0,012820513	0,001461372	4,386463845
726	6	18	48321491	48744674	0	282	0	0,002361334	0
727	8	18	51679079	53332018	0	197	0	0,001237313	0
728	7	18	54264439	55289445	0	192	0	0,001378112	0
729	12	18	55711599	58040001	0	377	0	0,001578886	0
730	20	18	59000815	61603345	0	338	0	0,001887119	0
731	2	18	63417488	64271375	0	0	NA	0	0
732	4	18	67068291	67997436	0	72	0	0,00090425	0
733	2	18	70203915	70535381	0	11	0	0,000276271	0
734	10	18	71740588	73001905	0	112	0	0,000703447	0
735	7	18	74069644	74980858	0	160	0	0,001607636	0
736	4	18	76740275	77289325	0	75	0	0,00125584	0
737	9	18	77623668	78005429	0	266	0	0,001485129	0
738	101	19	281043	3047633	10	3101	0,00261233	0,001777754	0,734727767
739	33	19	3094408	4224811	0	1594	0	0,002672703	0
740	67	19	4229540	6940463	4	2493	0,002419843	0,00216516	0,558813801
741	38	19	7112266	8327305	1	978	0,001893939	0,001490988	0,63512909
742	14	19	8367011	8809172	0	776	0	0,003249908	0
743	87	19	9004870	11689823	10	2998	0,006049607	0,002603751	1,161710146
744	59	19	12721732	14640049	7	2244	0,004545455	0,002018305	1,126057365
745	7	19	15052301	15236596	0	138	0	0,001386586	0
746	17	19	15270445	16060768	1	406	0,035714286	0,002549995	7,002814919
747	114	19	16177831	19774502	8	3149	0,001522939	0,001543536	0,493328103
748	5	19	30094924	31201777	0	158	0	0,001587541	0
749	28	19	32836500	34997258	1	597	0,003333333	0,001200905	1,387841988
750	129	19	35491227	39692522	4	1947	0,001147447	0,0011691	0,490739538
751	8	19	39693562	39900045	0	146	0	0,001466968	0
752	40	19	39936186	41314336	3	1747	0,009230769	0,003379215	1,365815684
753	94	19	41699115	45004576	1	1323	0,000816327	0,001332326	0,306353834
754	61	19	45116940	46544274	4	1692	0,002419843	0,001469495	0,823358633
755	40	19	46800303	48364769	1	879	0,001893939	0,00134006	0,7066624
756	7	19	48373723	48700877	0	63	0	0,000633007	0
757	149	19	48799714	52005043	19	4184	0,003002528	0,001870311	0,802681559
758	122	19	54296857	57746915	2	1826	0,000961538	0,001415586	0,339625548
759	3	19	58095508	58190519	0	0	NA	0	0
760	36	19	58193357	58951589	0	399	0	0,001432449	0
761	29	20	68351	1448417	0	999	0	0,001860886	0
762	49	20	1520790	4229721	0	1241	0	0,001601354	0
763	17	20	4666882	6760910	0	604	0	0,001786026	0
764	10	20	7863628	10654608	0	224	0	0,001406894	0
765	7	20	12989627	14318262	0	126	0	0,000904386	0
766	33	20	16252749	21696620	2	962	0,004032258	0,00151235	1,333109785
767	10	20	23016057	23476655	0	138	0	0,000990518	0
768	18	20	23583047	25566153	0	205	0	0,000936548	0
769	127	20	29891015	37668366	18	4576	0,00239904	0,001880187	0,637979336

770	128	20	39314488	52790512	25	4645	0,003950695	0,002076385	0,951339766
771	38	20	54572496	57901047	1	1043	0,002016129	0,00163969	0,614789843
772	4	20	58152564	58523735	0	111	0	0,001394052	0
773	14	20	59827559	61051026	1	743	0,010989011	0,002667442	2,059840563
774	6	21	15588451	17252377	0	108	0	0,000904341	0
775	5	21	18884700	19858197	0	29	0	0,000291384	0
776	8	21	26957968	28338832	0	337	0	0,002116621	0
777	88	21	30244513	37357047	1	1546	0,000725689	0,001468994	0,247002189
778	31	21	37406839	41174023	1	364	0,002849003	0,000678041	2,100908707
779	9	21	42539728	43430496	0	100	0	0,000628078	0
780	21	21	43483068	45115958	0	730	0	0,001835093	0
781	57	21	45138975	47706211	0	1306	0	0,002189805	0
782	12	22	17565844	18614498	0	246	0	0,001030254	0
783	61	22	19023795	22337213	2	2109	0,001568627	0,002082327	0,376652442
784	33	22	23401593	24989175	2	482	0,008658009	0,001101624	3,92965817
785	16	22	25202236	27026636	0	156	0	0,000653332	0
786	4	22	28202413	29153503	0	110	0	0,001381493	0
787	46	22	29168662	31521442	2	1711	0,00284495	0,002265817	0,627797874
788	9	22	31521443	32014572	0	375	0	0,002355291	0
789	5	22	32072242	32509016	0	430	0	0,005400382	0
790	5	22	32783569	33454358	0	255	0	0,00256217	0
791	6	22	35462129	35950048	0	322	0	0,002696275	0
792	181	22	36002811	47571336	43	7429	0,003380503	0,002350949	0,718965536
793	27	22	50296867	51001334	0	426	0	0,000892588	0
794	5	X	10413350	11793870	0	31	0	0,00031148	0
795	50	X	12809474	20135035	1	1010	0,001010101	0,001129849	0,447007201
796	5	X	21392536	21903542	0	47	0	0,000472243	0
797	4	X	23352133	23804343	0	179	0	0,002248066	0
798	6	X	24001837	25015103	0	429	0	0,003592243	0
799	8	X	30233677	30993201	0	57	0	0,000715865	0
800	3	X	35816459	36163187	0	0	0	0	0
801	10	X	37545012	38665790	0	84	0	0,000468988	0
802	10	X	39909068	41782716	1	960	0,022222222	0,004824121	2,303240741
803	8	X	43515467	45060146	0	94	0	0,000787111	0
804	23	X	46433219	47869126	0	731	0	0,00204158	0
805	63	X	48316920	51151687	0	1209	0	0,001484111	0
806	2	X	51486481	51645453	0	141	0	0,00354129	0
807	33	X	53078273	56593443	0	949	0	0,001767749	0
808	10	X	62854847	65488709	0	208	0	0,001045226	0
809	5	X	66764465	68061990	0	384	0	0,003858327	0
810	53	X	68835911	75005079	4	2151	0,003864734	0,002354051	0,820868847
811	13	X	76709648	78427726	1	644	0,027777778	0,003595576	3,862771739
812	4	X	79591003	80554046	0	47	0	0,000590274	0
813	3	X	82763269	83442933	0	28	0	0,000468847	0
814	5	X	84258832	85302566	0	94	0	0,000944486	0
815	16	X	99546642	100641183	0	589	0	0,001850432	0
816	43	X	100870110	103499614	0	172	0	0,000540364	0
817	24	X	103810996	107682727	0	244	0	0,000584195	0
818	18	X	107975712	112084043	0	537	0	0,001499765	0
819	4	X	113818551	114468635	0	96	0	0,001205667	0
820	2	X	115301975	115592625	0	11	NA	0,000552514	0
821	27	X	117629861	119709649	2	1487	0,006666667	0,002991199	1,114380184
822	6	X	122318006	124097666	0	348	0	0,002913987	0
823	2	X	125683369	125955769	0	0	NA	0	0
824	17	X	128673826	130533677	0	442	0	0,001306992	0
825	21	X	131157293	134305322	0	145	0	0,000455539	0
826	21	X	134478721	136113833	0	639	0	0,002294072	0
827	8	X	137713735	139866723	0	44	0	0,000315817	0
828	74	X	146993469	153402578	0	1054	0	0,001233786	0
829	25	X	153665266	154493874	1	849	0,006535948	0,002371137	1,378230444
