# Supplementary material for: The impact of the protein interactome on the syntenic structure of mammalian genomes
Source: PLoS One. 2017 Sep 14;12(9):e0179112. doi: 10.1371/journal.pone.0179112 (PMC5598925; doi:10.1371/journal.pone.0179112)
Supplement: S6 Table — In total 192 blocks had at least one high-confident cis- and trans-PPI. Here are information on these blocks in regard to chromosome position, CR, CR difference from the median of the randomization, number of genes in block, gene family information from HGNC [30] and the first level GO terms. (PDF) [file pone.0179112.s008.pdf]

Block no.	Chromosome	Connectivity ratio	Connectivity difference (log2)	No. Of genes i	Gene Name	HGNC gene family	GO term
54	1	170,137	3,659	4	CR1L	Complement system	membrane; extracellular region
54	1	170,137	3,659	4	CD46	Complement system	membrane; immune system process
54	1	170,137	3,659	4	CR2	Complement system	membrane; immune system process
54	1	170,137	3,659	4	CR1	Complement system	membrane; immune system process
213	4	92,144	4,382	9	CSN2	-	transporter activity; extracellular region
213	4	92,144	4,382	9	STATH	-	extracellular region
213	4	92,144	4,382	9	CSN1S1	-	transporter activity; extracellular region
213	4	92,144	4,382	9	FDCSP	-	extracellular region
213	4	92,144	4,382	9	PRR27	-	extracellular region
213	4	92,144	4,382	9	ODAM	-	extracellular region
213	4	92,144	4,382	9	CSN3	-	molecular_function; extracellular region
118	2	83,647	0,82	2	PRPF40A	-	membrane
118	2	83,647	0,82	2	FMNL2	-	-
497	10	68,178	0,556	2	ITGB1	Integrins	membrane; synapse; cell junction
497	10	68,178	0,556	2	NRP1	CD molecules	membrane; extracellular region
562	12	60,297	5,204	20	KLRB1	Killer cell lectin-like receptors	membrane
562	12	60,297	5,204	20	CLECL1	-	membrane
562	12	60,297	5,204	20	KLRD1	Killer cell lectin-like receptors	membrane
562	12	60,297	5,204	20	CLEC9A	C-type lectin domain containing	membrane
562	12	60,297	5,204	20	OLR1	C-type lectin domain containing	membrane; extracellular region; immune system process
562	12	60,297	5,204	20	KLRK1	Killer cell lectin-like receptors	membrane; immune system process
562	12	60,297	5,204	20	CD69	C-type lectin domain containing	membrane
562	12	60,297	5,204	20	KLRF2	Killer cell lectin-like receptors	membrane
562	12	60,297	5,204	20	TMEM52B	-	biological_process; membrane; molecular_function
562	12	60,297	5,204	20	GABARAPL1	-	membrane
562	12	60,297	5,204	20	KLRF1	Killer cell lectin-like receptors	membrane
562	12	60,297	5,204	20	CLEC2D	C-type lectin domain containing	membrane
664	15	18,866	1,128	6	MRPS11	Mitochondrial ribosomal proteins / small subunits	-
664	15	18,866	1,128	6	ISG20	-	immune system process
664	15	18,866	1,128	6	AEN	-	-
664	15	18,866	1,128	6	DET1	-	-
664	15	18,866	1,128	6	NTRK3	Immunoglobulin superfamily / I-set domain containing	membrane
664	15	18,866	1,128	6	MRPL46	Mitochondrial ribosomal proteins / large subunits	biological_process; cellular_component; molecular_function; cell junction
62	1	13,486	0,929	7	RNF187	RING-type (C3HC4) zinc fingers	-
62	1	13,486	0,929	7	OBSCN	Immunoglobulin superfamily / I-set domain containing	-
62	1	13,486	0,929	7	HIST3H2BB	Histones / Replication-dependent	molecular_function
62	1	13,486	0,929	7	HIST3H2A	Histones / Replication-dependent	-
365	7	9,161	1,82	14	Aug-03	Septins	-
365	7	9,161	1,82	14	KIAA0895	-	-
365	7	9,161	1,82	14	HERPUD2	-	membrane
365	7	9,161	1,82	14	EEPD1	-	-
365	7	9,161	1,82	14	DPY19L1	-	membrane
365	7	9,161	1,82	14	BBS9	-	membrane; molecular_function; response to stimulus
365	7	9,161	1,82	14	ANLN	Pleckstrin homology (PH) domain containing	-
365	7	9,161	1,82	14	AC083864.4	-	-
365	7	9,161	1,82	14	BMPER	-	extracellular region
365	7	9,161	1,82	14	TBX20	T-boxes	-
365	7	9,161	1,82	14	NPSR1	GPCR / Class A : Neuropeptide receptors : S	membrane; signal transducer activity
365	7	9,161	1,82	14	RP1189N17.1	-	-
365	7	9,161	1,82	14	RP9	-	-
53	1	7,086	1,555	15	C4BPA	-	extracellular region; immune system process
53	1	7,086	1,555	15	PIGR	Immunoglobulin superfamily / V-set domain containing	membrane; extracellular region
53	1	7,086	1,555	15	FAIM3	Immunoglobulin superfamily / Immunoglobulin-like domain containing	-
53	1	7,086	1,555	15	CD55	Blood group antigens	membrane; extracellular region; immune system process
53	1	7,086	1,555	15	DYRK3	-	-
53	1	7,086	1,555	15	YOD1	OTU domain containing	-
53	1	7,086	1,555	15	MAPKAPK2	-	signal transducer activity
53	1	7,086	1,555	15	C1ORF116	-	-
53	1	7,086	1,555	15	C4BPB	-	extracellular region; immune system process
53	1	7,086	1,555	15	IL24	Interleukins and interleukin receptors	extracellular region
53	1	7,086	1,555	15	IL10	Interleukins and interleukin receptors	extracellular region
53	1	7,086	1,555	15	PFKFB2	-	catalytic activity; metabolic process
53	1	7,086	1,555	15	FCAMR	Immunoglobulin superfamily / V-set domain containing	membrane; immune system process
746	19	7,003	1,799	17	AKAP8	A-kinase anchor proteins	membrane; immune system process
746	19	7,003	1,799	17	CYP4F12	Cytochrome P450s	membrane
746	19	7,003	1,799	17	RASAL3	-	membrane
746	19	7,003	1,799	17	EPHX3	Abhydrolase domain containing	biological_process; cellular_component; membrane; molecular_function; catalytic activity; extracellular region
746	19	7,003	1,799	17	PGLYRP2	-	membrane; extracellular region; immune system process
746	19	7,003	1,799	17	WIZ	Zinc fingers, C2H2-type	-

746	19	7,003	1,799	17	AKAP8L	-	biological_process
746	19	7,003	1,799	17	BRD4	-	-
122	2	6,378	0,709	11	NCKAP1	-	membrane
122	2	6,378	0,709	11	DNAJC10	Protein disulfide isomerases	cell; membrane
122	2	6,378	0,709	11	NUP35	-	membrane
122	2	6,378	0,709	11	DUSP19	Protein tyrosine phosphatases / Class I Cys-based PTPs : Atypical dual specificity phosphatases	-
122	2	6,378	0,709	11	PDE1A	Phosphodiesterases	-
122	2	6,378	0,709	11	FRZB	Secreted frizzled-related proteins	membrane; extracellular region
122	2	6,378	0,709	11	NEUROD1	Basic helix-loop-helix proteins	-
122	2	6,378	0,709	11	CERKL	-	-
122	2	6,378	0,709	11	ITGA4	Integrins	membrane
122	2	6,378	0,709	11	SSFA2	-	-
122	2	6,378	0,709	11	PPP1R1C	Serine/threonine phosphatases / Protein phosphatase 1, regulatory subunits	-
423	8	5,178	0,379	5	UBE2V2	Ubiquitin-conjugating enzymes E2	-
423	8	5,178	0,379	5	EFCAB1	EF-hand domain containing	-
423	8	5,178	0,379	5	MCM4	-	membrane
423	8	5,178	0,379	5	SNAI2	Zinc fingers, C2H2-type	-
423	8	5,178	0,379	5	PRKDC	-	membrane; rhythmic process
238	4	4,958	1,359	18	ASIC5	-	membrane
238	4	4,958	1,359	18	TDO2	-	-
238	4	4,958	1,359	18	CTSO	Cathepsins	-
238	4	4,958	1,359	18	C4ORF46	-	-
238	4	4,958	1,359	18	GLRB	-	membrane; synapse; cell junction
238	4	4,958	1,359	18	GUCY1A3	-	membrane
238	4	4,958	1,359	18	ETFDH	-	membrane; electron carrier activity
238	4	4,958	1,359	18	PPID	Tetratricopeptide (TTC) repeat domain containing	-
238	4	4,958	1,359	18	FNIP2	-	-
238	4	4,958	1,359	18	PDGFC	-	membrane; extracellular region
238	4	4,958	1,359	18	GRIA2	Ligand-gated ion channels / Glutamate receptors, ionotropic	membrane; synapse; cell junction
238	4	4,958	1,359	18	FAM198B	-	membrane
238	4	4,958	1,359	18	C4ORF45	-	-
238	4	4,958	1,359	18	GUCY1B3	-	-
238	4	4,958	1,359	18	RP11171N4.2	-	-
238	4	4,958	1,359	18	TMEM144	-	membrane
238	4	4,958	1,359	18	RAPGEF2	-	membrane; synapse; cell junction; signal transducer activity
238	4	4,958	1,359	18	RXFP1	GPCR / Class A : Relaxin family peptide receptors	membrane; signal transducer activity
658	15	4,798	0,467	12	LINGO1	Immunoglobulin superfamily / I-set domain containing	membrane
658	15	4,798	0,467	12	PEAK1	-	cell junction
658	15	4,798	0,467	12	HMG20A	High mobility group / Non-canonical	-
658	15	4,798	0,467	12	ETFA	-	electron carrier activity
658	15	4,798	0,467	12	NRG4	-	membrane; extracellular region
658	15	4,798	0,467	12	TSPAN3	Tetraspanins	biological_process; membrane; molecular_function
658	15	4,798	0,467	12	RCN2	EF-hand domain containing	-
658	15	4,798	0,467	12	SCAPER	Zinc fingers, C2H2-type	-
658	15	4,798	0,467	12	C15ORF27	-	-
658	15	4,798	0,467	12	FBXO22	F-boxes / "other"	-
658	15	4,798	0,467	12	ISL2	Homeoboxes / LIM class	-
658	15	4,798	0,467	12	PSTPIP1	-	membrane; immune system process
559	12	4,469	1,086	17	DCP1B	-	membrane
559	12	4,469	1,086	17	IQSEC3	-	-
559	12	4,469	1,086	17	WNK1	-	membrane
559	12	4,469	1,086	17	NINJ2	-	membrane
559	12	4,469	1,086	17	ADIPOR2	GPCR / Unclassified : Adiponectin receptors	membrane
559	12	4,469	1,086	17	CACNA2D4	Calcium channel subunits	membrane
559	12	4,469	1,086	17	CACNA1C	Voltage-gated ion channels / Calcium channels	membrane
559	12	4,469	1,086	17	CCDC77	-	membrane
559	12	4,469	1,086	17	B4GALNT3	Beta 4-glycosyltransferases	membrane
559	12	4,469	1,086	17	ERC1	-	membrane; synapse
559	12	4,469	1,086	17	KDM5A	Zinc fingers, PHD-type	rhythmic process
559	12	4,469	1,086	17	RAD52	-	-
559	12	4,469	1,086	17	WNT5B	Wingless-type MMTV integration sites	extracellular region
559	12	4,469	1,086	17	LRTM2	-	membrane
559	12	4,469	1,086	17	FBXL14	F-boxes / Leucine-rich repeats	-
725	18	4,386	0,791	15	MAPK4	Mitogen-activated protein kinase cascade / Kinases	-
725	18	4,386	0,791	15	SKA1	-	-
725	18	4,386	0,791	15	LIPG	-	extracellular region
725	18	4,386	0,791	15	DYM	-	membrane
725	18	4,386	0,791	15	C18ORF32	-	-
725	18	4,386	0,791	15	RP11886H22.1	-	-
725	18	4,386	0,791	15	SMAD7	SMADs	-

725	18	4,386	0,791	15	CTIF	-	-
725	18	4,386	0,791	15	MYO5B	Myosins / Myosin superfamily : Class V	-
725	18	4,386	0,791	15	ACAA2	-	catalytic activity; metabolic process
725	18	4,386	0,791	15	MBD1	-	-
725	18	4,386	0,791	15	CFAP53	-	biological_process; cellular_component; molecular_function
725	18	4,386	0,791	15	CXXC1	Zinc fingers, PHD-type	-
567	12	4,226	0,449	13	LDHB	-	membrane; catalytic activity
567	12	4,226	0,449	13	PYROXD1	-	-
567	12	4,226	0,449	13	KCNJ8	Voltage-gated ion channels / Potassium channels, Inwardly rectifying	membrane
567	12	4,226	0,449	13	CMAS	-	membrane
567	12	4,226	0,449	13	SPX	-	extracellular region
567	12	4,226	0,449	13	SLCO1A2	Solute carriers	membrane; transporter activity
567	12	4,226	0,449	13	GYS2	Glycosyltransferase group 1 domain containing	catalytic activity; metabolic process
567	12	4,226	0,449	13	GOLT1B	-	membrane; signal transducer activity
567	12	4,226	0,449	13	RP11125O5.2	-	-
567	12	4,226	0,449	13	ABCC9	ATP binding cassette transporters / subfamily C	membrane; transporter activity
567	12	4,226	0,449	13	RECQL	-	membrane
567	12	4,226	0,449	13	IAPP	Endogenous ligands	extracellular region
102	2	4,202	1,171	23	SLC20A1	Solute carriers	membrane; signal transducer activity
102	2	4,202	1,171	23	BCL2L11	-	membrane
102	2	4,202	1,171	23	TMEM87B	-	membrane
102	2	4,202	1,171	23	ZC3H8	Zinc fingers, CCCH-type domain containing	-
102	2	4,202	1,171	23	MALL	-	membrane
102	2	4,202	1,171	23	IL1A	Interleukins and interleukin receptors	extracellular region
102	2	4,202	1,171	23	CKAP2L	-	-
102	2	4,202	1,171	23	POLR1B	RNA polymerase subunits	-
102	2	4,202	1,171	23	FBLN7	Fibulins	extracellular region
102	2	4,202	1,171	23	TTL	-	-
102	2	4,202	1,171	23	ANAPC1	Anaphase promoting complex subunits	-
102	2	4,202	1,171	23	ACOXL	-	biological_process; cellular_component; molecular_function; electron carrier activity; metabolic process
102	2	4,202	1,171	23	ZC3H6	Zinc fingers, CCCH-type domain containing	biological_process; cellular_component; molecular_function
102	2	4,202	1,171	23	IL1B	Interleukins and interleukin receptors	extracellular region
102	2	4,202	1,171	23	MERTK	Immunoglobulin superfamily / I-set domain containing	membrane
102	2	4,202	1,171	23	NPHP1	-	membrane; cell junction; structural molecule activity
102	2	4,202	1,171	23	NT5DC4	-	-
102	2	4,202	1,171	23	CHCHD5	Coiled-coil-helix-coiled-coil-helix domain containing	-
102	2	4,202	1,171	23	BUB1	-	membrane
252	5	4,005	0,38	13	GHR	Fibronectin type III domain containing	membrane; extracellular region
252	5	4,005	0,38	13	C5ORF51	-	-
252	5	4,005	0,38	13	C6	Complement system	extracellular region; immune system process
252	5	4,005	0,38	13	PLCXD3	-	signal transducer activity
252	5	4,005	0,38	13	FBXO4	F-boxes / "other"	-
252	5	4,005	0,38	13	OXCT1	-	metabolic process
252	5	4,005	0,38	13	C7	Complement system	extracellular region; immune system process
252	5	4,005	0,38	13	CCDC152	-	-
252	5	4,005	0,38	13	PRKAA1	-	rhythmic process
252	5	4,005	0,38	13	RPL37	L ribosomal proteins	-
252	5	4,005	0,38	13	MROH2B	maestro heat-like repeat containing	-
252	5	4,005	0,38	13	SEPP1	-	-
252	5	4,005	0,38	13	CARD6	-	-
784	22	3,93	1,614	33	MIFAS1	-	-
784	22	3,93	1,614	33	UPB1	-	catalytic activity; metabolic process
784	22	3,93	1,614	33	AP000350.10	-	membrane; transporter activity
784	22	3,93	1,614	33	GNAZ	-	membrane; signal transducer activity
784	22	3,93	1,614	33	DRICH1	-	-
784	22	3,93	1,614	33	DERL3	-	membrane
784	22	3,93	1,614	33	ZNF70	Zinc fingers, C2H2-type	-
784	22	3,93	1,614	33	SLC2A11	Solute carriers	membrane; transporter activity; cell junction
784	22	3,93	1,614	33	CHCHD10	Coiled-coil-helix-coiled-coil-helix domain containing	molecular_function
784	22	3,93	1,614	33	RSPH14	-	biological_process; cellular_component; molecular_function
784	22	3,93	1,614	33	SMARCB1	-	-
784	22	3,93	1,614	33	LRRC75B	-	-
784	22	3,93	1,614	33	BCR	Pleckstrin homology (PH) domain containing	membrane; synapse; cell junction
784	22	3,93	1,614	33	CABIN1	-	-
784	22	3,93	1,614	33	SUSD2	-	membrane
784	22	3,93	1,614	33	VPREB3	Immunoglobulin superfamily / V-set domain containing	-
784	22	3,93	1,614	33	ADORA2A	GPCR / Class A : Adenosine receptors	membrane; signal transducer activity
784	22	3,93	1,614	33	RGL4	-	-
784	22	3,93	1,614	33	MMP11	-	extracellular region
784	22	3,93	1,614	33	GUCD1	-	-

784	22	3,93	1,614	33	C22ORF15	-	-
784	22	3,93	1,614	33	RAB36	RAB, member RAS oncogene	membrane
811	X	3,863	0,999	13	FGF16	-	metabolic process; extracellular region
811	X	3,863	0,999	13	PGAM4	-	catalytic activity; metabolic process
811	X	3,863	0,999	13	PGK1	-	membrane
811	X	3,863	0,999	13	TAF9B	-	-
811	X	3,863	0,999	13	COX7B	Mitochondrial respiratory chain complex / Complex IV	membrane
811	X	3,863	0,999	13	MAGT1	-	membrane
811	X	3,863	0,999	13	ATP7A	ATPases / P-type	membrane
811	X	3,863	0,999	13	ATRX	-	-
811	X	3,863	0,999	13	ZCCHC5	Zinc fingers, CCHC domain containing	-
17	1	3,847	1,416	22	TXNDC12	Protein disulfide isomerases	-
17	1	3,847	1,416	22	EPS15	EF-hand domain containing	membrane
17	1	3,847	1,416	22	FAM159A	-	membrane
17	1	3,847	1,416	22	KTI12	-	-
17	1	3,847	1,416	22	CC2D1B	-	-
17	1	3,847	1,416	22	BTF3L4	-	-
17	1	3,847	1,416	22	DMRTA2	-	-
17	1	3,847	1,416	22	GPX7	-	extracellular region
17	1	3,847	1,416	22	OSBPL9	Pleckstrin homology (PH) domain containing	membrane
17	1	3,847	1,416	22	CDKN2C	Ankyrin repeat domain containing	-
17	1	3,847	1,416	22	TTC39A	Tetratricopeptide (TTC) repeat domain containing	biological_process; cellular_component; molecular_function
17	1	3,847	1,416	22	PRPF38A	-	-
17	1	3,847	1,416	22	C1ORF185	-	-
17	1	3,847	1,416	22	COA7	Mitochondrial respiratory chain complex assembly factors	-
17	1	3,847	1,416	22	NRD1	-	-
17	1	3,847	1,416	22	ORC1	ATPases / AAA-type	-
17	1	3,847	1,416	22	RNF11	RING-type (C3HC4) zinc fingers	-
17	1	3,847	1,416	22	FAF1	UBX domain containing	membrane
17	1	3,847	1,416	22	RAB3B	RAB, member RAS oncogene	membrane
17	1	3,847	1,416	22	ZCCHC11	Zinc fingers, CCHC domain containing	-
17	1	3,847	1,416	22	ZFYVE9	Zinc fingers, FYVE domain containing	membrane
519	10	3,755	0,163	12	SHOC2	-	-
519	10	3,755	0,163	12	SMC3	Proteoglycans / Extracellular Matrix : Other	-
519	10	3,755	0,163	12	DUSP5	Protein tyrosine phosphatases / Class I Cys-based PTPs : MAP kinase phosphatases	-
519	10	3,755	0,163	12	RBM20	RNA binding motif (RRM) containing	-
519	10	3,755	0,163	12	BBIP1	-	-
519	10	3,755	0,163	12	PDCD4	-	-
519	10	3,755	0,163	12	ADD3	-	membrane; structural molecule activity
519	10	3,755	0,163	12	SMNDC1	Tudor domain containing	-
519	10	3,755	0,163	12	RP11451M19.3	-	-
519	10	3,755	0,163	12	MXI1	MAX dimerization proteins	-
519	10	3,755	0,163	12	XPNPEP1	-	-
199	4	3,725	0,163	19	CCDC96	-	-
199	4	3,725	1,014	19	S100P	S100 calcium binding proteins	membrane
199	4	3,725	1,014	19	BLOC1S4	Biogenesis of lysosomal organelles complex-1 subunits	-
199	4	3,725	1,014	19	SORCS2	-	membrane
199	4	3,725	1,014	19	PSAPL1	-	extracellular region
199	4	3,725	1,014	19	KIAA0232	-	-
199	4	3,725	1,014	19	SH3TC1	Tetratricopeptide (TTC) repeat domain containing	biological_process; cellular_component
199	4	3,725	1,014	19	GRPEL1	-	-
199	4	3,725	1,014	19	TBC1D14	-	-
199	4	3,725	1,014	19	MAN2B2	-	catalytic activity; metabolic process; extracellular region
199	4	3,725	1,014	19	AC097381.1	-	-
199	4	3,725	1,014	19	PPP2R2C	WD repeat domain containing	developmental process
199	4	3,725	1,014	19	TADA2B	-	-
199	4	3,725	1,014	19	HTRA3	-	extracellular region
199	4	3,725	1,014	19	AFAP1	Pleckstrin homology (PH) domain containing	-
199	4	3,725	1,014	19	ABLIM2	-	-
259	5	3,478	0,446	14	CTC534A2.2	-	-
259	5	3,478	0,446	14	SGTB	Tetratricopeptide (TTC) repeat domain containing	-
259	5	3,478	0,446	14	TRAPPC13	-	-
259	5	3,478	0,446	14	ADAMTS6	ADAM metallopeptidases with thrombospondin type 1 motif	extracellular region
259	5	3,478	0,446	14	CENPK	-	-
259	5	3,478	0,446	14	NLN	-	-
259	5	3,478	0,446	14	SREK1IP1	-	-
259	5	3,478	0,446	14	TRIM23	Tripartite motif containing / Tripartite motif containing	membrane
259	5	3,478	0,446	14	FAM159B	-	membrane
259	5	3,478	0,446	14	HTR1A	5-HT (serotonin) receptors	behavior; membrane; signal transducer activity
259	5	3,478	0,446	14	RNF180	RING-type (C3HC4) zinc fingers	membrane

259	5	3,478	0,446	14	PPWD1	WD repeat domain containing	-
259	5	3,478	0,446	14	CWC27	-	-
259	5	3,478	0,446	14	RGS7BP	-	membrane
119	2	2,971	1,766	32	BAZ2B	Zinc fingers, PHD-type	-
119	2	2,971	1,766	32	DAPL1	-	membrane
119	2	2,971	1,766	32	NR4A2	Nuclear hormone receptors	-
119	2	2,971	1,766	32	LY75	C-type lectin domain containing	membrane
119	2	2,971	1,766	32	TANC1	Tetratricopeptide (TTC) repeat domain containing	membrane; synapse; cell junction
119	2	2,971	1,766	32	UPP2	-	catalytic activity
119	2	2,971	1,766	32	CYTIP	-	-
119	2	2,971	1,766	32	TANK	-	-
119	2	2,971	1,766	32	LY75CD302	-	-
119	2	2,971	1,766	32	GALNT5	Glycosyltransferase family 2 domain containing	cellular_component; membrane
119	2	2,971	1,766	32	IFIH1	-	immune system process
119	2	2,971	1,766	32	RBMS1	RNA binding motif (RRM) containing	-
119	2	2,971	1,766	32	PKP4	Armadillo repeat containing	membrane; cell junction
119	2	2,971	1,766	32	TBR1	T-boxes	-
119	2	2,971	1,766	32	ITGB6	Integrins	membrane; cell junction
119	2	2,971	1,766	32	FAP	-	membrane; cell junction; extracellular region
119	2	2,971	1,766	32	PSMD14	Proteasome (prosome, macropain) subunits	-
119	2	2,971	1,766	32	KCNH7	Voltage-gated ion channels / Potassium channels	membrane
119	2	2,971	1,766	32	ERMN	-	-
119	2	2,971	1,766	32	PLA2R1	C-type lectin domain containing	membrane; extracellular region
119	2	2,971	1,766	32	SLC4A10	Solute carriers	membrane; transporter activity
119	2	2,971	1,766	32	GPD2	EF-hand domain containing	-
119	2	2,971	1,766	32	CCDC148	-	-
119	2	2,971	1,766	32	DPP4	CD molecules	membrane; cell junction; extracellular region
119	2	2,971	1,766	32	Feb-03	RING-type (C3HC4) zinc fingers	-
119	2	2,971	1,766	32	GCG	Endogenous ligands	extracellular region
119	2	2,971	1,766	32	GCA	EF-hand domain containing	membrane
119	2	2,971	1,766	32	CD302	C-type lectin domain containing	membrane
119	2	2,971	1,766	32	WDSUB1	WD repeat domain containing	-
393	7	2,805	0,003	13	AC106873.4	-	-
393	7	2,805	0,003	13	ST7OT4	Long non-coding RNAs	-
393	7	2,805	0,003	13	TFEC	Basic helix-loop-helix proteins	-
393	7	2,805	0,003	13	TES	-	cell junction
393	7	2,805	0,003	13	CAV1	-	membrane; structural molecule activity
393	7	2,805	0,003	13	CFTR	Ion channels / Chloride channels : Cystic fibrosis transmembrane conductance regulators	membrane
393	7	2,805	0,003	13	CAPZA2	-	membrane; extracellular region
393	7	2,805	0,003	13	ST7	-	membrane
393	7	2,805	0,003	13	ASZ1	Sterile alpha motif (SAM) domain containing	signal transducer activity
393	7	2,805	0,003	13	CAV2	-	membrane
393	7	2,805	0,003	13	CTTNBP2	Ankyrin repeat domain containing	-
393	7	2,805	0,003	13	MET	-	membrane; extracellular region
90	2	2,745	1,161	25	FIGLA	Basic helix-loop-helix proteins	-
90	2	2,745	1,161	25	GFPT1	-	rhythmic process
90	2	2,745	1,161	25	SNRPG	-	-
90	2	2,745	1,161	25	APLF	-	-
90	2	2,745	1,161	25	PCBP1	-	membrane
90	2	2,745	1,161	25	PROKR1	GPCR / Class A : Prokineticin receptors	membrane; signal transducer activity
90	2	2,745	1,161	25	TIA1	RNA binding motif (RRM) containing	-
90	2	2,745	1,161	25	FAM136A	-	-
90	2	2,745	1,161	25	BMP10	Endogenous ligands	growth; extracellular region
90	2	2,745	1,161	25	SNRNP27	-	biological_process; cellular_component
90	2	2,745	1,161	25	MXD1	MAX dimerization proteins	-
90	2	2,745	1,161	25	GKN2	BRICHOS domain containing	extracellular region
90	2	2,745	1,161	25	ANXA4	Annexins	-
90	2	2,745	1,161	25	NFU1	-	-
90	2	2,745	1,161	25	ARHGAP25	Pleckstrin homology (PH) domain containing	-
90	2	2,745	1,161	25	TGFA	-	membrane; extracellular region
90	2	2,745	1,161	25	ANTXR1	-	membrane; reproductive process
90	2	2,745	1,161	25	ADD2	-	membrane; structural molecule activity
90	2	2,745	1,161	25	C2ORF42	-	-
90	2	2,745	1,161	25	ASPRV1	-	membrane
90	2	2,745	1,161	25	AAK1	-	membrane
90	2	2,745	1,161	25	GKN1	BRICHOS domain containing	cellular_component; molecular_function; extracellular region
90	2	2,745	1,161	25	GMCL1	BTB/POZ domain containing	-
90	2	2,745	1,161	25	PCYOX1	-	-
531	11	2,695	1,275	27	PTPN5	Protein tyrosine phosphatases / Class I Cys-based PTPs : Non-receptor	membrane
531	11	2,695	1,275	27	USH1C	-	synapse

531	11	2,695	1,275	27	ABCC8	ATP binding cassette transporters / subfamily C	membrane
531	11	2,695	1,275	27	RP11113D6.10	-	-
531	11	2,695	1,275	27	TSG101	-	membrane
531	11	2,695	1,275	27	OTOG	-	membrane; structural molecule activity; extracellular region
531	11	2,695	1,275	27	SERGEF	-	-
531	11	2,695	1,275	27	IGSF22	Immunoglobulin superfamily / I-set domain containing	-
531	11	2,695	1,275	27	RP11113D6.6	-	-
531	11	2,695	1,275	27	MYOD1	Basic helix-loop-helix proteins	-
531	11	2,695	1,275	27	KCNC1	Voltage-gated ion channels / Potassium channels	membrane
531	11	2,695	1,275	27	HPS5	-	-
531	11	2,695	1,275	27	GTF2H1	General transcription factor IIH complex subunits	-
531	11	2,695	1,275	27	SAAL1	-	-
531	11	2,695	1,275	27	UEVLD	-	biological_process; molecular_function; catalytic activity
531	11	2,695	1,275	27	SPTY2D1	-	cellular_component
531	11	2,695	1,275	27	LDHA	-	membrane; catalytic activity
531	11	2,695	1,275	27	TPH1	-	metabolic process
531	11	2,695	1,275	27	TMEM86A	-	membrane
531	11	2,695	1,275	27	KCNJ11	Voltage-gated ion channels / Potassium channels, Inwardly rectifying	membrane
452	9	2,585	0,956	24	INSL4	-	extracellular region
452	9	2,585	0,956	24	RFX3	-	-
452	9	2,585	0,956	24	KCNV2	Voltage-gated ion channels / Potassium channels	membrane
452	9	2,585	0,956	24	ERMP1	-	membrane
452	9	2,585	0,956	24	RIC1	-	membrane
452	9	2,585	0,956	24	KIAA0020	-	-
452	9	2,585	0,956	24	INSL6	-	biological_process; cellular_component; extracellular region
452	9	2,585	0,956	24	CDC37L1	-	extracellular region
452	9	2,585	0,956	24	SPATA6L	-	-
452	9	2,585	0,956	24	SLC1A1	Solute carriers	membrane
452	9	2,585	0,956	24	PPAPDC2	-	-
452	9	2,585	0,956	24	PLGRKT	-	membrane
452	9	2,585	0,956	24	JAK2	SH2 domain containing	membrane; immune system process
452	9	2,585	0,956	24	RCL1	-	biological_process; catalytic activity
452	9	2,585	0,956	24	VLDLR	Low density lipoprotein receptors	membrane
452	9	2,585	0,956	24	GLIS3	Zinc fingers, C2H2-type	-
132	2	2,497	0,124	11	ATIC	-	membrane; catalytic activity; metabolic process
132	2	2,497	0,124	11	SMARCAL1	-	-
132	2	2,497	0,124	11	XRCC5	-	membrane
132	2	2,497	0,124	11	FN1	Fibronectin type III domain containing	extracellular region
132	2	2,497	0,124	11	VWC2L	-	synapse; cell junction; extracellular region
132	2	2,497	0,124	11	PECR	Short chain dehydrogenase/reductase superfamily / Classical SDR fold cluster 1	-
132	2	2,497	0,124	11	MREG	-	membrane
132	2	2,497	0,124	11	TMEM169	-	membrane
132	2	2,497	0,124	11	ABCA12	ATP binding cassette transporters / subfamily A	membrane; transporter activity
132	2	2,497	0,124	11	BARD1	Ankyrin repeat domain containing	-
132	2	2,497	0,124	11	Feb-00	RING-type (C3HC4) zinc fingers	-
802	X	2,303	0,259	10	BCOR	Ankyrin repeat domain containing	-
802	X	2,303	0,259	10	DDX3X	DEAD-boxes	membrane; immune system process
802	X	2,303	0,259	10	CASK	-	membrane; synapse
802	X	2,303	0,259	10	USP9X	Ubiquitin-specific peptidases	membrane
802	X	2,303	0,259	10	NYX	-	biological_process; molecular_function; extracellular region; response to stimulus
802	X	2,303	0,259	10	MED14	-	membrane
802	X	2,303	0,259	10	ATP6AP2	-	membrane; catalytic activity; metabolic process
802	X	2,303	0,259	10	CXORF38	-	-
802	X	2,303	0,259	10	GPR82	GPCR / Class A : Orphans	membrane; signal transducer activity
802	X	2,303	0,259	10	GPR34	GPCR / Class A : Orphans	membrane; signal transducer activity
96	2	2,141	0,197	19	TMEM127	-	membrane; molecular_function
96	2	2,141	0,197	19	ITPRIPL1	-	membrane
96	2	2,141	0,197	19	SNRNP200	-	membrane
96	2	2,141	0,197	19	STARD7	StAR-related lipid transfer (START) domain containing	transporter activity
96	2	2,141	0,197	19	TRIM43	Tripartite motif containing / Tripartite motif containing	-
96	2	2,141	0,197	19	CIAO1	WD repeat domain containing	-
96	2	2,141	0,197	19	PROM2	-	membrane
96	2	2,141	0,197	19	GPAT2	-	membrane; metabolic process
96	2	2,141	0,197	19	MRPS5	Mitochondrial ribosomal proteins / small subunits	-
96	2	2,141	0,197	19	ASTL	-	membrane
96	2	2,141	0,197	19	DUSP2	Protein tyrosine phosphatases / Class I Cys-based PTPs : MAP kinase phosphatases	-
96	2	2,141	0,197	19	NCAPH	-	membrane
96	2	2,141	0,197	19	MAL	-	membrane
96	2	2,141	0,197	19	KCNIP3	EF-hand domain containing	membrane
722	18	2,115	1,341	34	FHOD3	-	-

722	18	2,115	1,341	34	RNF125	RING-type (C3HC4) zinc fingers	membrane; immune system process
722	18	2,115	1,341	34	RNF138	RING-type (C3HC4) zinc fingers	-
722	18	2,115	1,341	34	CCDC178	-	-
722	18	2,115	1,341	34	INO80C	INO80 complex subunits	biological_process; molecular_function
722	18	2,115	1,341	34	GALNT1	Glycosyltransferase family 2 domain containing	membrane; extracellular region
722	18	2,115	1,341	34	ZSCAN30	Zinc fingers, C2H2-type	-
722	18	2,115	1,341	34	MAPRE2	-	-
722	18	2,115	1,341	34	TRAPPC8	Trafficking protein particle complex	-
722	18	2,115	1,341	34	B4GALT6	Beta 4-glycosyltransferases	membrane
722	18	2,115	1,341	34	GAREM	-	-
722	18	2,115	1,341	34	ELP2	WD repeat domain containing	-
722	18	2,115	1,341	34	MEP1B	-	membrane; extracellular region
722	18	2,115	1,341	34	RPRD1A	-	-
722	18	2,115	1,341	34	NOL4	-	-
722	18	2,115	1,341	34	SLC25A52	Solute carriers	membrane
722	18	2,115	1,341	34	TPGS2	-	-
722	18	2,115	1,341	34	RP11322E11.6	-	-
722	18	2,115	1,341	34	C18ORF21	-	-
722	18	2,115	1,341	34	ASXL3	-	-
722	18	2,115	1,341	34	MOCOS	-	cellular_component; catalytic activity
722	18	2,115	1,341	34	TTR	-	extracellular region
722	18	2,115	1,341	34	KLHL14	Kelch-like	membrane
722	18	2,115	1,341	34	SLC39A6	Solute carriers	membrane
722	18	2,115	1,341	34	AC012123.1	-	-
722	18	2,115	1,341	34	DTNA	-	membrane; synapse; cell junction
231	4	2,102	0,008	16	SPATA5	ATPases / AAA-type	-
231	4	2,102	0,008	16	SPRY1	-	membrane
231	4	2,102	0,008	16	CCNA2	-	-
231	4	2,102	0,008	16	ANXA5	Annexins	membrane
231	4	2,102	0,008	16	BBS7	-	membrane; response to stimulus
231	4	2,102	0,008	16	BBS12	Heat Shock Proteins / Chaperonins	-
231	4	2,102	0,008	16	IL2	Interleukins and interleukin receptors	cell; extracellular region; immune system process
231	4	2,102	0,008	16	NUDT6	Nudix motif containing	biological_process; cellular_component
231	4	2,102	0,008	16	EXOSC9	-	-
231	4	2,102	0,008	16	IL21	Interleukins and interleukin receptors	extracellular region
231	4	2,102	0,008	16	FGF2	Endogenous ligands	chemoattractant activity; extracellular region
231	4	2,102	0,008	16	ADAD1	-	-
231	4	2,102	0,008	16	KIAA1109	-	membrane
231	4	2,102	0,008	16	TMEM155	-	extracellular region
231	4	2,102	0,008	16	TRPC3	Voltage-gated ion channels / Transient receptor potential cation channels	membrane
778	21	2,101	1,158	31	ERG	-	signal transducer activity
778	21	2,101	1,158	31	DOPEY2	-	molecular_function
778	21	2,101	1,158	31	DYRK1A	-	-
778	21	2,101	1,158	31	HLCS	-	catalytic activity; metabolic process
778	21	2,101	1,158	31	WRB	-	membrane
778	21	2,101	1,158	31	RIPPLY3	-	biological_process
778	21	2,101	1,158	31	BRWD1	WD repeat domain containing	molecular_function
778	21	2,101	1,158	31	LCA5L	-	-
778	21	2,101	1,158	31	SIM2	Basic helix-loop-helix proteins	-
778	21	2,101	1,158	31	B3GALT5	Beta 3-glycosyltransferases	membrane
778	21	2,101	1,158	31	DSCR3	-	biological_process; molecular_function
778	21	2,101	1,158	31	TTC3	Tetratricopeptide (TTC) repeat domain containing	-
778	21	2,101	1,158	31	KCNJ6	Voltage-gated ion channels / Potassium channels, Inwardly rectifying	membrane
778	21	2,101	1,158	31	IGSF5	Immunoglobulin superfamily / I-set domain containing	membrane; cell junction
778	21	2,101	1,158	31	PSMG1	-	-
778	21	2,101	1,158	31	DSCR8	-	biological_process; cellular_component; molecular_function
778	21	2,101	1,158	31	SH3BGR	-	-
778	21	2,101	1,158	31	HMGN1	High-mobility group / Canonical	-
778	21	2,101	1,158	31	SETD4	-	membrane; cell junction
778	21	2,101	1,158	31	PIGP	Phosphatidylinositol glycan anchor biosynthesis	membrane
778	21	2,101	1,158	31	MORC3	-	-
778	21	2,101	1,158	31	CLDN14	Claudins	membrane; cell junction; structural molecule activity
778	21	2,101	1,158	31	CHAF1B	WD repeat domain containing	-
778	21	2,101	1,158	31	B3GALT5AS1	-	-
778	21	2,101	1,158	31	DSCR4	-	biological_process; cellular_component; molecular_function
778	21	2,101	1,158	31	KCNJ15	Voltage-gated ion channels / Potassium channels, Inwardly rectifying	membrane
778	21	2,101	1,158	31	ETS2	-	-
773	20	2,06	0,106	14	MTG2	-	membrane
773	20	2,06	0,106	14	GATA5	GATA zinc finger domain containing	-
773	20	2,06	0,106	14	TAF4	-	-

773	20	2,06	0,106	14	ADRM1	-	membrane
773	20	2,06	0,106	14	LSM14B	-	-
773	20	2,06	0,106	14	PSMA7	Proteasome (prosome, macropain) subunits	-
773	20	2,06	0,106	14	SS18L1	-	-
773	20	2,06	0,106	14	HRH3	GPCR / Class A : Histamine receptors	membrane; signal transducer activity
773	20	2,06	0,106	14	OSBPL2	Oxysterol binding proteins	-
773	20	2,06	0,106	14	RBBP8NL	-	-
773	20	2,06	0,106	14	RPS21	S ribosomal proteins	-
773	20	2,06	0,106	14	LAMA5	Laminins	structural molecule activity; extracellular region
773	20	2,06	0,106	14	RP11429E11.3	-	-
773	20	2,06	0,106	14	CABLES2	-	-
629	14	2,009	0,655	25	TMX1	Protein disulfide isomerases	cell; membrane
629	14	2,009	0,655	25	MAP4K5	Mitogen-activated protein kinase cascade / Kinase kinase kinase kinases	-
629	14	2,009	0,655	25	C14ORF182	-	-
629	14	2,009	0,655	25	NEMF	-	-
629	14	2,009	0,655	25	L2HGDH	-	-
629	14	2,009	0,655	25	TRIM9	Tripartite motif containing / Tripartite motif containing	synapse; cell junction
629	14	2,009	0,655	25	PYGL	Glycogen phosphorylases	catalytic activity; metabolic process
629	14	2,009	0,655	25	VCPKMT	-	cellular_component
629	14	2,009	0,655	25	MGAT2	-	membrane
629	14	2,009	0,655	25	DNAAF2	-	-
629	14	2,009	0,655	25	FRMD6	-	membrane
629	14	2,009	0,655	25	KLHDC2	-	-
629	14	2,009	0,655	25	KLHDC1	-	-
629	14	2,009	0,655	25	NIN	EF-hand domain containing	-
629	14	2,009	0,655	25	POLE2	DNA polymerases	-
629	14	2,009	0,655	25	ABHD12B	Abhydrolase domain containing	-
629	14	2,009	0,655	25	SOS2	Pleckstrin homology (PH) domain containing	-
629	14	2,009	0,655	25	CDKL1	Cyclin-dependent kinases	-
629	14	2,009	0,655	25	SAV1	-	-
629	14	2,009	0,655	25	ATL1	-	membrane
629	14	2,009	0,655	25	C14ORF183	-	-
629	14	2,009	0,655	25	ARF6	ADP-ribosylation factors	membrane
173	3	1,994	1,07	31	CNBP	Zinc fingers, CCHC domain containing	-
173	3	1,994	1,07	31	RAB43	RAB, member RAS oncogene	membrane
173	3	1,994	1,07	31	RP11723O4.6	-	-
173	3	1,994	1,07	31	LINC01565	-	-
173	3	1,994	1,07	31	TXNRD3	-	cell; electron carrier activity
173	3	1,994	1,07	31	SEC61A1	-	membrane
173	3	1,994	1,07	31	CHCHD6	Serine/threonine phosphatases / Protein phosphatase 1, regulatory subunits	membrane
173	3	1,994	1,07	31	COPG1	-	membrane; structural molecule activity
173	3	1,994	1,07	31	EFCC1	EF-hand domain containing	-
173	3	1,994	1,07	31	RUVBL1	INO80 complex subunits	membrane
173	3	1,994	1,07	31	MGLL	-	membrane
173	3	1,994	1,07	31	C3ORF56	-	-
173	3	1,994	1,07	31	PODXL2	-	membrane
173	3	1,994	1,07	31	RPN1	-	membrane
173	3	1,994	1,07	31	CHST13	Sulfotransferases, membrane-bound	membrane
173	3	1,994	1,07	31	MCM2	-	-
173	3	1,994	1,07	31	DNAJB8	Heat shock proteins / DNAJ (HSP40)	-
173	3	1,994	1,07	31	KBTBD12	BTB/POZ domain containing	-
173	3	1,994	1,07	31	PLXNA1	Plexins	membrane
173	3	1,994	1,07	31	ABTB1	BTB/POZ domain containing	-
173	3	1,994	1,07	31	EEFSEC	-	-
173	3	1,994	1,07	31	ACAD9	Mitochondrial respiratory chain complex assembly factors	electron carrier activity; metabolic process
173	3	1,994	1,07	31	TXNRD3NB	-	-
173	3	1,994	1,07	31	GATA2	GATA zinc finger domain containing	-
173	3	1,994	1,07	31	GP9	CD molecules	membrane
173	3	1,994	1,07	31	TPRA1	GPCR / Unclassified : 7TM orphan receptors	membrane
173	3	1,994	1,07	31	C3ORF22	-	-
173	3	1,994	1,07	31	KIAA1257	-	-
173	3	1,994	1,07	31	RAB7A	RAB, member RAS oncogene	membrane
127	2	1,989	0,028	18	GTF3C3	Tetratricopeptide (TTC) repeat domain containing	membrane
127	2	1,989	0,028	18	ANKRD44	Serine/threonine phosphatases / Protein phosphatase 6, regulatory subunits	-
127	2	1,989	0,028	18	RFTN2	-	membrane
127	2	1,989	0,028	18	HECW2	-	-
127	2	1,989	0,028	18	HSPD1	Heat Shock Proteins / Chaperonins	membrane
127	2	1,989	0,028	18	PLCL1	-	signal transducer activity
127	2	1,989	0,028	18	PGAP1	-	membrane
127	2	1,989	0,028	18	COQ10B	-	membrane

127	2	1,989	0,028	18	SF3B1	-	-
127	2	1,989	0,028	18	STK17B	-	membrane
127	2	1,989	0,028	18	HSPE1MOB4	-	-
127	2	1,989	0,028	18	BOLL	RNA binding motif (RRM) containing	-
127	2	1,989	0,028	18	CCDC150	-	-
127	2	1,989	0,028	18	MOB4	MOB kinase activators	membrane
127	2	1,989	0,028	18	MARS2	Aminoacyl tRNA synthetases / Class I	-
127	2	1,989	0,028	18	HSPE1	Heat Shock Proteins / Chaperonins	membrane
127	2	1,989	0,028	18	AC011997.1	-	-
127	2	1,989	0,028	18	C2ORF66	-	-
289	5	1,978	1,177	33	ITK	SH2 domain containing	immune system process
289	5	1,978	1,177	33	MED7	-	-
289	5	1,978	1,177	33	ADRA1B	GPCR / Class A : Adrenoceptors : alpha	membrane; signal transducer activity
289	5	1,978	1,177	33	HAVCR2	Immunoglobulin superfamily / V-set domain containing	membrane; cell junction; immune system process
289	5	1,978	1,177	33	EBF1	-	-
289	5	1,978	1,177	33	PWWP2A	-	-
289	5	1,978	1,177	33	ZBED8	Zinc fingers, BED-type	-
289	5	1,978	1,177	33	PTTG1	-	-
289	5	1,978	1,177	33	SLU7	-	membrane
289	5	1,978	1,177	33	CYFIP2	-	membrane; synapse; cell junction
289	5	1,978	1,177	33	IL12B	Interleukins and interleukin receptors	membrane; extracellular region
289	5	1,978	1,177	33	ADAM19	ADAM metallopeptidase domain containing	membrane
289	5	1,978	1,177	33	ATP10B	ATPases / P-type	membrane
289	5	1,978	1,177	33	HAVCR1	Immunoglobulin superfamily / V-set domain containing	membrane
289	5	1,978	1,177	33	LSM11	-	-
289	5	1,978	1,177	33	TTC1	Tetratricopeptide (TTC) repeat domain containing	-
289	5	1,978	1,177	33	FAM71B	-	-
289	5	1,978	1,177	33	SOX30	SRY (sex determining region Y)-boxes	-
289	5	1,978	1,177	33	C5ORF52	-	-
289	5	1,978	1,177	33	CLINT1	-	membrane
289	5	1,978	1,177	33	TIMD4	Immunoglobulin superfamily / V-set domain containing	membrane
289	5	1,978	1,177	33	GABRA6	Ligand-gated ion channels / GABA(A) receptors	membrane; synapse; cell junction
289	5	1,978	1,177	33	C1QTNF2	-	extracellular region
289	5	1,978	1,177	33	CCNJL	-	-
289	5	1,978	1,177	33	FNDC9	Fibronectin type III domain containing	membrane
289	5	1,978	1,177	33	UBLCP1	Serine/threonine phosphatases / CTD aspartate-based phosphatases	-
289	5	1,978	1,177	33	GABRB2	Ligand-gated ion channels / GABA(A) receptors	membrane; synapse; cell junction
289	5	1,978	1,177	33	FABP6	Fatty acid binding protein family	membrane; transporter activity
289	5	1,978	1,177	33	THG1L	-	-
289	5	1,978	1,177	33	NIPAL4	-	membrane
21	1	1,97	1,17	33	ITGB3BP	-	membrane; signal transducer activity
21	1	1,97	1,17	33	PDE4B	Phosphodiesterases	membrane
21	1	1,97	1,17	33	TCTEX1D1	-	-
21	1	1,97	1,17	33	KANK4	KN motif and ankyrin repeat domain containing	-
21	1	1,97	1,17	33	USP1	Ubiquitin-specific peptidases	-
21	1	1,97	1,17	33	SLC35D1	Solute carriers	membrane
21	1	1,97	1,17	33	EFCAB7	EF-hand domain containing	-
21	1	1,97	1,17	33	ANGPTL3	Fibrinogen C domain containing	extracellular region
21	1	1,97	1,17	33	MIER1	-	signal transducer activity
21	1	1,97	1,17	33	ATG4C	-	extracellular region
21	1	1,97	1,17	33	FOXD3	Forkhead boxes	-
21	1	1,97	1,17	33	JAK1	-	membrane
21	1	1,97	1,17	33	ROR1	Immunoglobulin superfamily / I-set domain containing	membrane
21	1	1,97	1,17	33	WDR78	WD repeat domain containing	-
21	1	1,97	1,17	33	LEPR	Immunoglobulin superfamily / Immunoglobulin-like domain containing	membrane; extracellular region
21	1	1,97	1,17	33	NFIA	-	cell junction
21	1	1,97	1,17	33	DOCK7	-	-
21	1	1,97	1,17	33	RAVER2	RNA binding motif (RRM) containing	-
21	1	1,97	1,17	33	ALG6	-	membrane
21	1	1,97	1,17	33	UBE2U	Ubiquitin-conjugating enzymes E2	-
21	1	1,97	1,17	33	CACHD1	-	membrane
21	1	1,97	1,17	33	LEPROT	-	membrane
21	1	1,97	1,17	33	DNAJC6	Heat shock proteins / DNAJ (HSP40)	synapse
21	1	1,97	1,17	33	TM2D1	-	membrane
21	1	1,97	1,17	33	SGIP1	-	membrane
21	1	1,97	1,17	33	INADL	-	-
21	1	1,97	1,17	33	PGM1	-	-
21	1	1,97	1,17	33	L1TD1	-	-
21	1	1,97	1,17	33	INSL5	Endogenous ligands	biological_process; cellular_component; extracellular region
21	1	1,97	1,17	33	AK4	Adenylate kinases	-

596	12	1,958	0,376	25	TRIAP1	-	-
596	12	1,958	0,376	25	RPLP0	L ribosomal proteins	membrane
596	12	1,958	0,376	25	PXN	-	cell junction
596	12	1,958	0,376	25	UNC119B	-	-
596	12	1,958	0,376	25	RNF10	RING-type (C3HC4) zinc fingers	-
596	12	1,958	0,376	25	CCDC64	-	-
596	12	1,958	0,376	25	SPPL3	-	membrane
596	12	1,958	0,376	25	MLEC	-	membrane
596	12	1,958	0,376	25	DYNLL1	Cytoplasmic dyneins	membrane
596	12	1,958	0,376	25	MSI1	RNA binding motif (RRM) containing	-
596	12	1,958	0,376	25	COQ5	-	membrane
596	12	1,958	0,376	25	CABP1	EF-hand domain containing	membrane; synapse; cell junction
596	12	1,958	0,376	25	HNF1A	Homeoboxes / HNF class	-
596	12	1,958	0,376	25	PLA2G1B	-	extracellular region
596	12	1,958	0,376	25	SRSF9	Serine/arginine-rich splicing factors	-
596	12	1,958	0,376	25	C12ORF43	-	-
596	12	1,958	0,376	25	RAB35	RAB, member RAS oncogene	membrane
596	12	1,958	0,376	25	POP5	-	-
596	12	1,958	0,376	25	GCN1L1	-	-
596	12	1,958	0,376	25	ACADS	-	electron carrier activity; metabolic process
596	12	1,958	0,376	25	SIRT4	-	-
596	12	1,958	0,376	25	COX6A1	Mitochondrial respiratory chain complex / Complex IV	membrane
237	4	1,92	0,735	27	NPY2R	GPCR / Class A : Neuropeptide receptors : Y	membrane; signal transducer activity
237	4	1,92	0,735	27	MAB21L2	-	-
237	4	1,92	0,735	27	LRAT	-	membrane; response to stimulus
237	4	1,92	0,735	27	TLR2	CD molecules	membrane; immune system process
237	4	1,92	0,735	27	MND1	-	-
237	4	1,92	0,735	27	RBM46	RNA binding motif (RRM) containing	-
237	4	1,92	0,735	27	RNF175	RING-type (C3HC4) zinc fingers	membrane
237	4	1,92	0,735	27	RPS3A	S ribosomal proteins	-
237	4	1,92	0,735	27	ARFIP1	-	-
237	4	1,92	0,735	27	GATB	-	-
237	4	1,92	0,735	27	FGG	Fibrinogen C domain containing	structural molecule activity; extracellular region
237	4	1,92	0,735	27	FGA	Fibrinogen C domain containing	structural molecule activity; extracellular region; immune system process
237	4	1,92	0,735	27	DCHS2	Cadherins / Cadherin-related	biological_process; cellular_component; membrane; molecular_function
237	4	1,92	0,735	27	PRSS48	Serine peptidases / Serine peptidases	extracellular region
237	4	1,92	0,735	27	FAM160A1	-	-
237	4	1,92	0,735	27	PLRG1	WD repeat domain containing	membrane
237	4	1,92	0,735	27	DCLK2	-	-
237	4	1,92	0,735	27	TMEM154	-	membrane
237	4	1,92	0,735	27	FBXW7	WD repeat domain containing	-
237	4	1,92	0,735	27	FHDC1	-	-
237	4	1,92	0,735	27	KIAA0922	-	membrane
237	4	1,92	0,735	27	SFRP2	Secreted frizzled-related proteins	extracellular region
237	4	1,92	0,735	27	TRIM2	Tripartite motif containing / Tripartite motif containing	-
237	4	1,92	0,735	27	FGB	Fibrinogen C domain containing	structural molecule activity; extracellular region; immune system process
237	4	1,92	0,735	27	SH3D19	-	-
237	4	1,92	0,735	27	LRBA	WD repeat domain containing	biological_process; membrane; molecular_function
237	4	1,92	0,735	27	TIGD4	-	-
64	1	1,91	0,026	17	GPR137B	-	membrane
64	1	1,91	0,026	17	EDARADD	-	-
64	1	1,91	0,026	17	ZP4	Zona pellucida glycoproteins	membrane; extracellular region; signal transducer activity
64	1	1,91	0,026	17	LYST	WD repeat domain containing	-
64	1	1,91	0,026	17	RYR2	Ion channels / Ryanodine receptors	cell; membrane
64	1	1,91	0,026	17	MTR	-	-
64	1	1,91	0,026	17	ACTN2	EF-hand domain containing	extracellular region
64	1	1,91	0,026	17	LGALS8	Lectins, galactoside-binding	membrane
64	1	1,91	0,026	17	NID1	-	extracellular region
64	1	1,91	0,026	17	GGPS1	-	-
64	1	1,91	0,026	17	TBCE	-	-
64	1	1,91	0,026	17	GNG4	-	membrane; signal transducer activity
64	1	1,91	0,026	17	ERO1LB	-	-
64	1	1,91	0,026	17	HEATR1	-	membrane
64	1	1,91	0,026	17	MT1HL1	Metallothioneins	-
64	1	1,91	0,026	17	B3GALNT2	Beta 3-glycosyltransferases	membrane
64	1	1,91	0,026	17	MTRNR2L11	-	extracellular region
88	2	1,889	0,932	30	SPRED2	-	membrane
88	2	1,889	0,932	30	XPO1	Exportins	membrane; transporter activity
88	2	1,889	0,932	30	AHSA2	-	-
88	2	1,889	0,932	30	REL	-	-

88	2	1,889	0,932	30	ACTR2	-	membrane
88	2	1,889	0,932	30	EHBP1	-	membrane
88	2	1,889	0,932	30	PAPOLG	-	membrane
88	2	1,889	0,932	30	VPS54	-	growth
88	2	1,889	0,932	30	AFTPH	-	-
88	2	1,889	0,932	30	LGALSL	-	-
88	2	1,889	0,932	30	RAB1A	RAB, member RAS oncogene	membrane
88	2	1,889	0,932	30	COMMD1	-	membrane
88	2	1,889	0,932	30	PUS10	-	-
88	2	1,889	0,932	30	BCL11A	Zinc fingers, C2H2-type	-
88	2	1,889	0,932	30	SERTAD2	-	-
88	2	1,889	0,932	30	TMEM17	-	membrane
88	2	1,889	0,932	30	C2ORF74	-	-
88	2	1,889	0,932	30	CCT4	Heat Shock Proteins / Chaperonins	-
88	2	1,889	0,932	30	OTX1	Homeoboxes / PRD class	-
88	2	1,889	0,932	30	WDPCP	-	membrane
88	2	1,889	0,932	30	CEP68	-	cell junction
88	2	1,889	0,932	30	KIAA1841	-	-
88	2	1,889	0,932	30	USP34	Ubiquitin-specific peptidases	-
88	2	1,889	0,932	30	UGP2	-	metabolic process
88	2	1,889	0,932	30	PEX13	-	membrane
88	2	1,889	0,932	30	B3GNT2	Beta 3-glycosyltransferases	membrane
88	2	1,889	0,932	30	FAM161A	-	response to stimulus
88	2	1,889	0,932	30	SLC1A4	Solute carriers	membrane
88	2	1,889	0,932	30	MDH1	-	catalytic activity
88	2	1,889	0,932	30	PELI1	Pellino homologs	-
143	3	1,884	0,01	18	FBLN2	Fibulins	extracellular region
143	3	1,884	0,01	18	RP11434D12.1	-	-
143	3	1,884	0,01	18	LSM3	-	-
143	3	1,884	0,01	18	XPC	-	-
143	3	1,884	0,01	18	HDAC11	-	-
143	3	1,884	0,01	18	FGD5	Zinc fingers, FYVE domain containing	membrane
143	3	1,884	0,01	18	CHCHD4	Coiled-coil-helix-coiled-coil-helix domain containing	-
143	3	1,884	0,01	18	NR2C2	Nuclear hormone receptors	-
143	3	1,884	0,01	18	TPRXL	Homeoboxes / PRD class	-
143	3	1,884	0,01	18	ZFYVE20	-	-
143	3	1,884	0,01	18	C3ORF20	-	-
143	3	1,884	0,01	18	NUP210	-	membrane
143	3	1,884	0,01	18	IQSEC1	-	membrane
143	3	1,884	0,01	18	WNT7A	Wingless-type MMTV integration sites	synapse; extracellular region
143	3	1,884	0,01	18	CCDC174	-	-
143	3	1,884	0,01	18	MRPS25	Mitochondrial ribosomal proteins / small subunits	-
143	3	1,884	0,01	18	TMEM43	-	membrane
34	1	1,863	0,593	26	SLC6A17	Solute carriers	membrane; synapse; cell junction
34	1	1,863	0,593	26	STRIP1	-	molecular_function
34	1	1,863	0,593	26	PROK1	Endogenous ligands	extracellular region
34	1	1,863	0,593	26	AHCYL1	-	biological_process; membrane
34	1	1,863	0,593	26	ALX3	Homeoboxes / PRD class	-
34	1	1,863	0,593	26	DRAM2	-	membrane
34	1	1,863	0,593	26	UBL4B	-	-
34	1	1,863	0,593	26	CHIA	-	metabolic process; extracellular region; immune system process
34	1	1,863	0,593	26	DENND2D	DENN/MADD domain containing	-
34	1	1,863	0,593	26	CSF1	-	membrane; extracellular region; immune system process
34	1	1,863	0,593	26	CEPT1	-	membrane
34	1	1,863	0,593	26	KCNC4	Voltage-gated ion channels / Potassium channels	membrane
34	1	1,863	0,593	26	PIFO	-	-
34	1	1,863	0,593	26	CD53	Tetraspanins	membrane; cell junction
34	1	1,863	0,593	26	RBM15	RNA binding motif (RRM) containing	membrane
34	1	1,863	0,593	26	EPS8L3	-	-
34	1	1,863	0,593	26	CHI3L2	-	extracellular region
34	1	1,863	0,593	26	LAMTOR5	-	-
34	1	1,863	0,593	26	SLC16A4	Solute carriers	membrane
34	1	1,863	0,593	26	LRIF1	-	-
256	5	1,837	0,045	19	GPX8	-	membrane
256	5	1,837	0,045	19	MIER3	-	-
256	5	1,837	0,045	19	MAP3K1	Mitogen-activated protein kinase cascade / Kinase kinase kinases	-
256	5	1,837	0,045	19	SETD9	-	-
256	5	1,837	0,045	19	AC022431.2	-	-
256	5	1,837	0,045	19	SKIV2L2	-	-
256	5	1,837	0,045	19	CDC20B	WD repeat domain containing	-

256	5	1,837	0,045	19	PPAP2A	-	-
256	5	1,837	0,045	19	IL6ST	Interleukins and interleukin receptors	membrane; extracellular region
256	5	1,837	0,045	19	DDX4	DEAD-boxes	-
256	5	1,837	0,045	19	ANKRD55	Ankyrin repeat domain containing	-
256	5	1,837	0,045	19	CCNO	-	-
256	5	1,837	0,045	19	CTC236F12.4	-	-
256	5	1,837	0,045	19	IL31RA	Interleukins and interleukin receptors	membrane; synapse; cell junction; immune system process
256	5	1,837	0,045	19	DHX29	DEAH-boxes	-
256	5	1,837	0,045	19	MCIDAS	-	-
256	5	1,837	0,045	19	SLC38A9	Solute carriers	membrane
283	5	1,835	0,044	19	ETF1	-	-
283	5	1,835	0,044	19	GFRA3	-	membrane
283	5	1,835	0,044	19	HSPA9	Heat shock proteins / HSP70	-
283	5	1,835	0,044	19	EGR1	Zinc fingers, C2H2-type	-
283	5	1,835	0,044	19	CTNNA1	-	membrane; cell junction; structural molecule activity
283	5	1,835	0,044	19	CDC23	Tetratricopeptide (TTC) repeat domain containing	-
283	5	1,835	0,044	19	REEP2	Receptor accessory proteins	membrane
283	5	1,835	0,044	19	KIF20A	Kinesins	transporter activity
283	5	1,835	0,044	19	PKD2L2	Voltage-gated ion channels / Transient receptor potential cation channels	biological_process; membrane
283	5	1,835	0,044	19	LRRTM2	-	membrane; synapse; cell junction
283	5	1,835	0,044	19	BRD8	-	-
283	5	1,835	0,044	19	KDM3B	Chromatin-modifying enzymes / K-demethylases	antioxidant activity
283	5	1,835	0,044	19	FAM13B	Rho GTPase activating proteins	-
283	5	1,835	0,044	19	WNT8A	Wingless-type MMTV integration sites	extracellular region
283	5	1,835	0,044	19	CDC25C	Protein tyrosine phosphatases / Class III Cys-based PTPs	-
283	5	1,835	0,044	19	NME5	-	cellular_component
283	5	1,835	0,044	19	SIL1	-	-
283	5	1,835	0,044	19	FAM53C	-	-
283	5	1,835	0,044	19	MATR3	-	membrane; structural molecule activity
715	17	1,696	1,803	77	DUS1L	-	catalytic activity
715	17	1,696	1,803	77	CBX4	-	-
715	17	1,696	1,803	77	C1QTNF1	-	extracellular region
715	17	1,696	1,803	77	PDE6G	Phosphodiesterases	response to stimulus
715	17	1,696	1,803	77	RAC3	Endogenous ligands	membrane
715	17	1,696	1,803	77	CBX8	-	-
715	17	1,696	1,803	77	RPTOR	WD repeat domain containing	-
715	17	1,696	1,803	77	STRA13	-	-
715	17	1,696	1,803	77	P4HB	Protein disulfide isomerases	cell; membrane; extracellular region
715	17	1,696	1,803	77	FSCN2	Fascins	-
715	17	1,696	1,803	77	CEP131	-	molecular_function
715	17	1,696	1,803	77	C17ORF89	-	-
715	17	1,696	1,803	77	USP36	Ubiquitin-specific peptidases	-
715	17	1,696	1,803	77	CCDC40	-	molecular_function
715	17	1,696	1,803	77	CHMP6	Charged multivesicular body proteins	membrane
715	17	1,696	1,803	77	LRRC45	-	-
715	17	1,696	1,803	77	ENPP7	-	membrane; catalytic activity; metabolic process
715	17	1,696	1,803	77	TMEM105	-	membrane
715	17	1,696	1,803	77	TBC1D16	-	-
715	17	1,696	1,803	77	CCDC57	-	-
715	17	1,696	1,803	77	SLC26A11	Solute carriers	membrane
715	17	1,696	1,803	77	RNF213	RING-type (C3HC4) zinc fingers	membrane
715	17	1,696	1,803	77	CCDC137	-	-
715	17	1,696	1,803	77	CD7	Immunoglobulin superfamily / V-set domain containing	membrane; immune system process
715	17	1,696	1,803	77	CSNK1D	-	membrane; rhythmic process
715	17	1,696	1,803	77	ARHGDIA	-	membrane
715	17	1,696	1,803	77	ENGASE	-	metabolic process
715	17	1,696	1,803	77	NPTX1	-	-
715	17	1,696	1,803	77	AATK	-	membrane
715	17	1,696	1,803	77	LGALS3BP	Endogenous ligands	membrane; extracellular region
715	17	1,696	1,803	77	ENTHD2	-	-
715	17	1,696	1,803	77	OXLD1	-	-
715	17	1,696	1,803	77	C17ORF70	-	-
715	17	1,696	1,803	77	ALYREF	THO complex subunits	-
715	17	1,696	1,803	77	ANAPC11	Anaphase promoting complex subunits	-
715	17	1,696	1,803	77	RBFOX3	RNA binding motif (RRM) containing	-
715	17	1,696	1,803	77	HGS	Zinc fingers, FYVE domain containing	membrane
715	17	1,696	1,803	77	GPS1	-	-
715	17	1,696	1,803	77	PCYT2	-	cellular_component; catalytic activity
715	17	1,696	1,803	77	CBX2	-	-
715	17	1,696	1,803	77	SLC38A10	Solute carriers	membrane

715	17	1,696	1,803	77	NPLOC4	-	-
715	17	1,696	1,803	77	SIRT7	-	-
715	17	1,696	1,803	77	CANT1	-	membrane; signal transducer activity
715	17	1,696	1,803	77	CARD14	-	-
715	17	1,696	1,803	77	DCXR	Short chain dehydrogenase/reductase superfamily / Classical SDR fold cluster 1	membrane
715	17	1,696	1,803	77	EIF4A3	DEAD-boxes	membrane
715	17	1,696	1,803	77	CYTH1	Pleckstrin homology (PH) domain containing	membrane
715	17	1,696	1,803	77	BAIAP2	-	membrane
715	17	1,696	1,803	77	ARL16	ADP-ribosylation factors-like	-
715	17	1,696	1,803	77	PYCR1	-	-
715	17	1,696	1,803	77	RFNG	Beta 3-glycosyltransferases	membrane; molecular_function; extracellular region
715	17	1,696	1,803	77	FAM195B	-	-
715	17	1,696	1,803	77	GCGR	GPCR / Class B : Glucagon receptors	membrane; signal transducer activity
715	17	1,696	1,803	77	FASN	Short chain dehydrogenase/reductase superfamily / Atypical members	membrane; catalytic activity; metabolic process
715	17	1,696	1,803	77	ACTG1	-	membrane
715	17	1,696	1,803	77	ENDOV	-	-
715	17	1,696	1,803	77	KIAA1731NL	-	-
715	17	1,696	1,803	77	ASPSCR1	UBX domain containing	biological_process; cellular_component; membrane; molecular_function
715	17	1,696	1,803	77	GAA	-	membrane; catalytic activity; metabolic process
715	17	1,696	1,803	77	NPB	Endogenous ligands	extracellular region
715	17	1,696	1,803	77	SLC16A3	Solute carriers	membrane
715	17	1,696	1,803	77	PPP1R27	Serine/threonine phosphatases / Protein phosphatase 1, regulatory subunits	-
715	17	1,696	1,803	77	SGSH	-	catalytic activity; metabolic process
715	17	1,696	1,803	77	MYADML2	-	membrane
715	17	1,696	1,803	77	MAFG	-	-
715	17	1,696	1,803	77	TIMP2	-	extracellular region
715	17	1,696	1,803	77	NOTUM	-	extracellular region
505	10	1,664	0,029	19	NRBF2	-	-
505	10	1,664	0,029	19	TMEM26	-	membrane
505	10	1,664	0,029	19	RHOBTB1	BTB/POZ domain containing	-
505	10	1,664	0,029	19	CCDC6	-	biological_process
505	10	1,664	0,029	19	C10ORF107	-	-
505	10	1,664	0,029	19	EGR2	Zinc fingers, C2H2-type	-
505	10	1,664	0,029	19	ADO	-	-
505	10	1,664	0,029	19	JMJD1C	-	-
505	10	1,664	0,029	19	ARID5B	-	-
505	10	1,664	0,029	19	BICC1	Sterile alpha motif (SAM) domain containing	-
505	10	1,664	0,029	19	SLC16A9	Solute carriers	membrane
505	10	1,664	0,029	19	REEP3	Receptor accessory proteins	membrane
505	10	1,664	0,029	19	CDK1	Cyclin-dependent kinases	membrane
505	10	1,664	0,029	19	RTKN2	Pleckstrin homology (PH) domain containing	-
505	10	1,664	0,029	19	PHYHIPL	Fibronectin type III domain containing	-
505	10	1,664	0,029	19	FAM13C	-	-
505	10	1,664	0,029	19	LINC01553	-	-
505	10	1,664	0,029	19	ANK3	Ankyrin repeat domain containing	membrane; synapse; cell junction
505	10	1,664	0,029	19	ZNF365	Zinc fingers, C2H2-type	-
42	1	1,51	1,677	106	RP11544M22.1[illegible]	-	-
42	1	1,51	1,677	106	HSPA6	Heat shock proteins / HSP70	-
42	1	1,51	1,677	106	RP11565P22.6	-	-
42	1	1,51	1,677	106	CD244	Immunoglobulin superfamily / Immunoglobulin-like domain containing	membrane; immune system process
42	1	1,51	1,677	106	PPOX	-	membrane
42	1	1,51	1,677	106	F11R	Immunoglobulin superfamily / V-set domain containing	membrane; cell junction
42	1	1,51	1,677	106	CRP	-	extracellular region
42	1	1,51	1,677	106	PFDN2	-	-
42	1	1,51	1,677	106	SDHC	Mitochondrial respiratory chain complex / Complex II	membrane; electron carrier activity
42	1	1,51	1,677	106	NDUFS2	Mitochondrial respiratory chain complex / Complex I	membrane; electron carrier activity
42	1	1,51	1,677	106	FCRLB	Immunoglobulin superfamily / Immunoglobulin-like domain containing	-
42	1	1,51	1,677	106	CFAP45	-	-
42	1	1,51	1,677	106	SPTA1	EF-hand domain containing	membrane
42	1	1,51	1,677	106	ADAMTS4	ADAM metallopeptidases with thrombospondin type 1 motif	membrane; extracellular region
42	1	1,51	1,677	106	PEA15	-	-
42	1	1,51	1,677	106	C1ORF204	-	-
42	1	1,51	1,677	106	TOMM40L	-	biological_process; membrane; molecular_function
42	1	1,51	1,677	106	DEDD	-	-
42	1	1,51	1,677	106	C1ORF110	-	-
42	1	1,51	1,677	106	SLAMF6	Immunoglobulin superfamily / V-set domain containing	membrane; immune system process
42	1	1,51	1,677	106	HSD17B7	Short chain dehydrogenase/reductase superfamily / Classical SDR fold cluster 2	membrane
42	1	1,51	1,677	106	RP11190A12.7	-	-
42	1	1,51	1,677	106	SH2D1B	SH2 domain containing	immune system process
42	1	1,51	1,677	106	VANGL2	-	membrane

42	1	1,51	1,677	106	UAP1	-	metabolic process
42	1	1,51	1,677	106	PCP4L1	-	-
42	1	1,51	1,677	106	ACKR1	Blood group antigens	membrane; signal transducer activity
42	1	1,51	1,677	106	NR1I3	Nuclear hormone receptors	-
42	1	1,51	1,677	106	VSIG8	Immunoglobulin superfamily / V-set domain containing	membrane
42	1	1,51	1,677	106	C1ORF226	-	-
42	1	1,51	1,677	106	IGSF8	Immunoglobulin superfamily / V-set domain containing	membrane
42	1	1,51	1,677	106	FCRL6	Immunoglobulin superfamily / Immunoglobulin-like domain containing	membrane
42	1	1,51	1,677	106	IGSF9	Immunoglobulin superfamily / I-set domain containing	membrane; synapse; cell junction
42	1	1,51	1,677	106	UHMK1	RNA binding motif (RRM) containing	-
42	1	1,51	1,677	106	COPA	WD repeat domain containing	membrane; structural molecule activity; extracellular region
42	1	1,51	1,677	106	NUF2	-	membrane; molecular_function
42	1	1,51	1,677	106	TAGLN2	-	-
42	1	1,51	1,677	106	USP21	Ubiquitin-specific peptidases	cellular_component
42	1	1,51	1,677	106	PIGM	Phosphatidylinositol glycan anchor biosynthesis	membrane
42	1	1,51	1,677	106	FCER1G	-	membrane
42	1	1,51	1,677	106	DDR2	-	membrane
42	1	1,51	1,677	106	NHLH1	Basic helix-loop-helix proteins	-
42	1	1,51	1,677	106	ARHGAP30	Rho GTPase activating proteins	-
42	1	1,51	1,677	106	UFC1	-	-
42	1	1,51	1,677	106	KLHDC9	-	-
42	1	1,51	1,677	106	OLFML2B	-	extracellular region
42	1	1,51	1,677	106	SLAMF1	Immunoglobulin superfamily / Immunoglobulin-like domain containing	membrane; extracellular region; immune system process
42	1	1,51	1,677	106	CASQ1	Protein disulfide isomerases	membrane
42	1	1,51	1,677	106	CFAP126	-	membrane
42	1	1,51	1,677	106	LY9	Immunoglobulin superfamily / Immunoglobulin-like domain containing	membrane; molecular_function; immune system process
42	1	1,51	1,677	106	FCER1A	Immunoglobulin superfamily / Immunoglobulin-like domain containing	membrane
42	1	1,51	1,677	106	NIT1	-	biological_process
42	1	1,51	1,677	106	PVRL4	Immunoglobulin superfamily / V-set domain containing	-
42	1	1,51	1,677	106	NCSTN	-	membrane
42	1	1,51	1,677	106	B4GALT3	Beta 4-glycosyltransferases	membrane
42	1	1,51	1,677	106	NOS1AP	-	-
42	1	1,51	1,677	106	CADM3	Immunoglobulin superfamily / V-set domain containing	membrane; cell junction
42	1	1,51	1,677	106	FCRLA	Immunoglobulin superfamily / Immunoglobulin-like domain containing	-
42	1	1,51	1,677	106	C1ORF111	-	-
42	1	1,51	1,677	106	APCS	-	extracellular region
42	1	1,51	1,677	106	APOA2	Apolipoproteins	extracellular region
42	1	1,51	1,677	106	DUSP23	Protein tyrosine phosphatases / Class I Cys-based PTPs : Atypical dual specificity phosphatases	-
42	1	1,51	1,677	106	TSTD1	-	-
42	1	1,51	1,677	106	MPZ	Immunoglobulin superfamily / V-set domain containing	membrane; structural molecule activity
42	1	1,51	1,677	106	ATF6	basic leucine zipper proteins	membrane
42	1	1,51	1,677	106	SLAMF8	Immunoglobulin superfamily / Immunoglobulin-like domain containing	membrane
42	1	1,51	1,677	106	USF1	Basic helix-loop-helix proteins	-
42	1	1,51	1,677	106	AIM2	-	immune system process
42	1	1,51	1,677	106	SLAMF9	Immunoglobulin superfamily / V-set domain containing	membrane
42	1	1,51	1,677	106	CD48	Immunoglobulin superfamily / V-set domain containing	membrane
42	1	1,51	1,677	106	DUSP12	Protein tyrosine phosphatases / Class I Cys-based PTPs : Atypical dual specificity phosphatases	-
475	9	1,473	1,164	27	AKNA	-	membrane
475	9	1,473	1,164	27	BSPRY	-	membrane
475	9	1,473	1,164	27	POLE3	DNA polymerases	-
475	9	1,473	1,164	27	DFNB31	-	-
475	9	1,473	1,164	27	FKBP15	Serine/threonine phosphatases / Protein phosphatase 1, regulatory subunits	membrane
475	9	1,473	1,164	27	TNFSF8	Tumor necrosis factor (ligand) superfamily	membrane
475	9	1,473	1,164	27	TNC	Fibronectin type III domain containing	membrane; extracellular region
475	9	1,473	1,164	27	HDHD3	-	metabolic process
475	9	1,473	1,164	27	ATP6V1G1	ATPases / V-type	-
475	9	1,473	1,164	27	WDR31	WD repeat domain containing	-
475	9	1,473	1,164	27	C9ORF91	-	-
475	9	1,473	1,164	27	KIF12	Kinesins	-
475	9	1,473	1,164	27	CDC26	Anaphase promoting complex subunits	-
475	9	1,473	1,164	27	AMBP	Lipocalins	extracellular region
475	9	1,473	1,164	27	PRPF4	WD repeat domain containing	-
475	9	1,473	1,164	27	ZNF618	Zinc fingers, C2H2-type	-
475	9	1,473	1,164	27	COL27A1	Collagens	extracellular region
475	9	1,473	1,164	27	RGS3	Regulators of G-protein signaling	membrane
475	9	1,473	1,164	27	RNF183	RING-type (C3HC4) zinc fingers	membrane
475	9	1,473	1,164	27	ALAD	-	catalytic activity; metabolic process
475	9	1,473	1,164	27	SLC31A2	Solute carriers	membrane
475	9	1,473	1,164	27	TNFSF15	Tumor necrosis factor (ligand) superfamily	membrane; extracellular region
475	9	1,473	1,164	27	C9ORF43	-	-

475	9	1,473	1,164	27	SLC31A1	Solute carriers	membrane
181	3	1,437	0,972	40	DHX36	DEAH-boxes	membrane
181	3	1,437	0,972	40	GPR149	GPCR / Class A : Orphans	membrane; signal transducer activity
181	3	1,437	0,972	40	MED12L	-	-
181	3	1,437	0,972	40	WWTR1	-	-
181	3	1,437	0,972	40	RNF13	RING-type (C3HC4) zinc fingers	membrane
181	3	1,437	0,972	40	RP11166N6.3	-	-
181	3	1,437	0,972	40	RP11166N6.1	-	-
181	3	1,437	0,972	40	TMEM14E	-	-
181	3	1,437	0,972	40	MME	CD molecules	membrane; synapse
181	3	1,437	0,972	40	SERP1	-	membrane
181	3	1,437	0,972	40	COMMD2	-	-
181	3	1,437	0,972	40	C3ORF79	-	-
181	3	1,437	0,972	40	P2RY1	Purinergic receptors	membrane; signal transducer activity
181	3	1,437	0,972	40	SUCNR1	GPCR / Class A : Orphans	biological_process; membrane; signal transducer activity
181	3	1,437	0,972	40	SIAH2	-	-
181	3	1,437	0,972	40	C3ORF33	-	-
181	3	1,437	0,972	40	MBNL1	Zinc fingers, CCCH-type domain containing	-
181	3	1,437	0,972	40	PFN2	-	membrane
181	3	1,437	0,972	40	SLC33A1	Solute carriers	membrane
181	3	1,437	0,972	40	ANKUB1	Ankyrin repeat domain containing	-
181	3	1,437	0,972	40	IGSF10	Immunoglobulin superfamily / I-set domain containing	extracellular region
181	3	1,437	0,972	40	SELT	-	-
181	3	1,437	0,972	40	CLRN1	-	membrane; response to stimulus
181	3	1,437	0,972	40	TSC22D2	-	-
181	3	1,437	0,972	40	EIF2A	-	-
181	3	1,437	0,972	40	ERICH6	-	-
181	3	1,437	0,972	40	RAP2B	-	membrane
181	3	1,437	0,972	40	PLCH1	EF-hand domain containing	membrane; signal transducer activity
181	3	1,437	0,972	40	ARHGEF26	Pleckstrin homology (PH) domain containing	-
50	1	1,426	0,932	39	RNPEP	-	extracellular region
50	1	1,426	0,932	39	PTPN7	Protein tyrosine phosphatases / Class I Cys-based PTPs : Non-receptor	-
50	1	1,426	0,932	39	LAD1	-	structural molecule activity; extracellular region
50	1	1,426	0,932	39	KLHL12	Kelch-like	-
50	1	1,426	0,932	39	TMEM9	-	biological_process; membrane; molecular_function
50	1	1,426	0,932	39	KDM5B	Zinc fingers, PHD-type	rhythmic process
50	1	1,426	0,932	39	LMOD1	-	membrane
50	1	1,426	0,932	39	SHISA4	Shisa homologs	membrane
50	1	1,426	0,932	39	IPO9	Importins	membrane
50	1	1,426	0,932	39	PPP1R12B	Serine/threonine phosphatases / Protein phosphatase 1, regulatory subunits	-
50	1	1,426	0,932	39	UBE2T	Ubiquitin-conjugating enzymes E2	-
50	1	1,426	0,932	39	PKP1	Armadillo repeat containing	cell junction; signal transducer activity
50	1	1,426	0,932	39	CSRP1	-	-
50	1	1,426	0,932	39	NAV1	-	-
50	1	1,426	0,932	39	RABIF	-	-
50	1	1,426	0,932	39	GPR25	GPCR / Class A : Orphans	membrane; signal transducer activity
50	1	1,426	0,932	39	LGR6	GPCR / Class A : Orphans	membrane; signal transducer activity
50	1	1,426	0,932	39	ADIPOR1	GPCR / Unclassified : Adiponectin receptors	membrane
50	1	1,426	0,932	39	KIF14	Kinesins	membrane
50	1	1,426	0,932	39	IGFN1	Immunoglobulin superfamily / I-set domain containing	biological_process
50	1	1,426	0,932	39	ASCL5	Basic helix-loop-helix proteins	biological_process; cellular_component; molecular_function
50	1	1,426	0,932	39	TNNT2	-	-
50	1	1,426	0,932	39	ZNF281	Zinc fingers, C2H2-type	-
50	1	1,426	0,932	39	PHLDA3	-	membrane
50	1	1,426	0,932	39	ARL8A	ADP-ribosylation factors-like	membrane
50	1	1,426	0,932	39	CAMSAP2	-	-
50	1	1,426	0,932	39	TNNI1	-	-
50	1	1,426	0,932	39	SYT2	Synaptotagmins	membrane; synapse; cell junction
50	1	1,426	0,932	39	C1ORF106	-	-
50	1	1,426	0,932	39	TIMM17A	-	membrane
50	1	1,426	0,932	39	DDX59	Zinc fingers, HIT-type	membrane
50	1	1,426	0,932	39	ELF3	-	-
50	1	1,426	0,932	39	KIF21B	WD repeat domain containing	-
50	1	1,426	0,932	39	GPR37L1	GPCR / Class A : Orphans	membrane; signal transducer activity
50	1	1,426	0,932	39	NR5A2	Nuclear hormone receptors	-
50	1	1,426	0,932	39	MGAT4EP	-	-
50	1	1,426	0,932	39	CACNA1S	Voltage-gated ion channels / Calcium channels	membrane
50	1	1,426	0,932	39	CYB5R1	-	biological_process; membrane; molecular_function
171	3	1,413	1,466	64	MAATS1	-	membrane
171	3	1,413	1,466	64	PTPLB	-	-

171	3	1,413	1,466	64	RABL3	-	-
171	3	1,413	1,466	64	CD80	Immunoglobulin superfamily / V-set domain containing	membrane
171	3	1,413	1,466	64	COX17	Mitochondrial respiratory chain complex assembly factors	-
171	3	1,413	1,466	64	STXBP5L	WD repeat domain containing	membrane
171	3	1,413	1,466	64	PARP15	Poly (ADP-ribose) polymerases	-
171	3	1,413	1,466	64	POLQ	DNA polymerases	-
171	3	1,413	1,466	64	CD86	Immunoglobulin superfamily / V-set domain containing	membrane; immune system process
171	3	1,413	1,466	64	SEC22A	-	membrane; transporter activity
171	3	1,413	1,466	64	TIMMDC1	-	membrane
171	3	1,413	1,466	64	HSPBAP1	-	-
171	3	1,413	1,466	64	TMEM39A	-	membrane
171	3	1,413	1,466	64	OSBPL11	Pleckstrin homology (PH) domain containing	membrane
171	3	1,413	1,466	64	GTF2E1	General transcription factors	-
171	3	1,413	1,466	64	ADPRH	-	-
171	3	1,413	1,466	64	LRRC58	-	-
171	3	1,413	1,466	64	GSK3B	-	membrane; rhythmic process
171	3	1,413	1,466	64	ITGB5	Integrins	membrane
171	3	1,413	1,466	64	RP11484M3.5	-	-
171	3	1,413	1,466	64	ZNF148	Zinc fingers, C2H2-type	-
171	3	1,413	1,466	64	MUC13	Mucins	membrane; extracellular region
171	3	1,413	1,466	64	MYLK	Immunoglobulin superfamily / I-set domain containing	-
171	3	1,413	1,466	64	KPNA1	Importins	-
171	3	1,413	1,466	64	NR1I2	Nuclear hormone receptors	-
171	3	1,413	1,466	64	ADCY5	Adenylate cyclases	membrane
171	3	1,413	1,466	64	POGLUT1	-	-
171	3	1,413	1,466	64	CSTA	-	structural molecule activity
171	3	1,413	1,466	64	FSTL1	EF-hand domain containing	extracellular region
171	3	1,413	1,466	64	UPK1B	Tetraspanins	membrane; structural molecule activity
171	3	1,413	1,466	64	FBXO40	F-boxes / "other"	molecular_function
171	3	1,413	1,466	64	PARP9	Poly (ADP-ribose) polymerases	membrane
171	3	1,413	1,466	64	PDIA5	Protein disulfide isomerases	cell
171	3	1,413	1,466	64	ARHGAP31	Rho GTPase activating proteins	cell junction
171	3	1,413	1,466	64	SLC15A2	Solute carriers	membrane; transporter activity
171	3	1,413	1,466	64	POPDC2	-	biological_process; membrane; molecular_function
171	3	1,413	1,466	64	NDUFB4	Mitochondrial respiratory chain complex / Complex I	membrane
171	3	1,413	1,466	64	FAM162A	-	membrane
171	3	1,413	1,466	64	SEMA5B	Semaphorins	membrane; chemorepellent activity
171	3	1,413	1,466	64	ARGFX	Homeoboxes / PRD class	-
171	3	1,413	1,466	64	IQCB1	-	-
171	3	1,413	1,466	64	HEG1	-	membrane; cell junction; extracellular region
171	3	1,413	1,466	64	SLC12A8	Solute carriers	membrane
171	3	1,413	1,466	64	HCLS1	-	membrane
171	3	1,413	1,466	64	C3ORF30	-	-
171	3	1,413	1,466	64	DIRC2	Solute carriers	membrane
171	3	1,413	1,466	64	HGD	-	-
171	3	1,413	1,466	64	PARP14	Poly (ADP-ribose) polymerases	membrane
171	3	1,413	1,466	64	EAF2	-	-
171	3	1,413	1,466	64	SNX4	Sorting nexins	membrane
171	3	1,413	1,466	64	CASR	GPCR / Class C : Calcium-sensing receptors	membrane; signal transducer activity
171	3	1,413	1,466	64	GOLGB1	-	membrane
171	3	1,413	1,466	64	IGSF11	Immunoglobulin superfamily / I-set domain containing	membrane
171	3	1,413	1,466	64	WDR5B	WD repeat domain containing	-
171	3	1,413	1,466	64	UMPS	-	catalytic activity; metabolic process
171	3	1,413	1,466	64	GPR156	GPCR / Class C : Orphans	membrane; signal transducer activity
171	3	1,413	1,466	64	B4GALT4	Beta 4-glycosyltransferases	membrane
171	3	1,413	1,466	64	CCDC14	-	-
171	3	1,413	1,466	64	DTX3L	RING-type (C3HC4) zinc fingers	-
171	3	1,413	1,466	64	ILDR1	-	membrane
171	3	1,413	1,466	64	CCDC58	-	-
171	3	1,413	1,466	64	PLA1A	-	extracellular region
171	3	1,413	1,466	64	KALRN	Immunoglobulin superfamily / I-set domain containing	-
749	19	1,388	0,297	28	KCTD15	-	-
749	19	1,388	0,297	28	DPY19L3	-	membrane
749	19	1,388	0,297	28	PEPD	-	-
749	19	1,388	0,297	28	LRP3	Low density lipoprotein receptors	membrane
749	19	1,388	0,297	28	TDRD12	Tudor domain containing	biological_process; cellular_component; molecular_function
749	19	1,388	0,297	28	RGS9BP	-	membrane; response to stimulus
749	19	1,388	0,297	28	CEBPG	basic leucine zipper proteins	-
749	19	1,388	0,297	28	PDCD5	-	-
749	19	1,388	0,297	28	C19ORF40	-	-

749	19	1,388	0,297	28	CHST8	Sulfotransferases, membrane-bound	membrane
749	19	1,388	0,297	28	RHPN2	-	-
749	19	1,388	0,297	28	NUDT19	Nudix motif containing	biological_process; cellular_component
749	19	1,388	0,297	28	ANKRD27	Ankyrin repeat domain containing	membrane
749	19	1,388	0,297	28	KIAA0355	-	-
749	19	1,388	0,297	28	UBA2	Ubiquitin-like modifier activating enzymes	-
749	19	1,388	0,297	28	GPI	-	membrane; extracellular region
749	19	1,388	0,297	28	CEP89	-	-
749	19	1,388	0,297	28	ZNF507	Zinc fingers, C2H2-type	-
749	19	1,388	0,297	28	SLC7A10	Solute carriers	membrane
749	19	1,388	0,297	28	SLC7A9	Solute carriers	membrane
749	19	1,388	0,297	28	CEBPA	basic leucine zipper proteins	-
749	19	1,388	0,297	28	WTIP	-	cell junction
749	19	1,388	0,297	28	GPATCH1	G patch domain containing	-
749	19	1,388	0,297	28	WDR88	WD repeat domain containing	-
749	19	1,388	0,297	28	LSM14A	-	-
829	X	1,378	0,183	25	PLXNA3	Plexins	membrane; cell junction
829	X	1,378	0,183	25	SMIM9	-	membrane
829	X	1,378	0,183	25	RAB39B	RAB, member RAS oncogene	membrane
829	X	1,378	0,183	25	FAM50A	-	-
829	X	1,378	0,183	25	GAB3	Pleckstrin homology (PH) domain containing	-
829	X	1,378	0,183	25	SLC10A3	Solute carriers	membrane
829	X	1,378	0,183	25	FAM3A	-	biological_process; cellular_component; membrane; molecular_function; extracellular region
829	X	1,378	0,183	25	VBP1	-	-
829	X	1,378	0,183	25	BRCC3	-	-
829	X	1,378	0,183	25	MPP1	-	membrane
829	X	1,378	0,183	25	MTCP1	-	-
829	X	1,378	0,183	25	GDI1	-	-
829	X	1,378	0,183	25	FUNDC2	-	membrane
829	X	1,378	0,183	25	DKC1	-	membrane
829	X	1,378	0,183	25	CMC4	-	-
829	X	1,378	0,183	25	G6PD	-	membrane
829	X	1,378	0,183	25	F8	-	extracellular region
829	X	1,378	0,183	25	UBL4A	-	membrane
464	9	1,369	0,1	15	C9ORF153	-	-
464	9	1,369	0,1	15	NTRK2	Immunoglobulin superfamily / I-set domain containing	membrane
464	9	1,369	0,1	15	ISCA1	-	structural molecule activity
464	9	1,369	0,1	15	HNRNPK	-	membrane; cell junction
464	9	1,369	0,1	15	AGTPBP1	-	-
464	9	1,369	0,1	15	RMI1	-	-
464	9	1,369	0,1	15	UBQLN1	Ubiquilin family	membrane
464	9	1,369	0,1	15	ZCCHC6	Zinc fingers, CCHC domain containing	-
464	9	1,369	0,1	15	KIF27	Kinesins	-
464	9	1,369	0,1	15	C9ORF64	-	-
464	9	1,369	0,1	15	SLC28A3	Solute carriers	membrane
464	9	1,369	0,1	15	IDNK	-	biological_process; molecular_function
464	9	1,369	0,1	15	NAA35	N(alpha)-acetyltransferase subunits	-
464	9	1,369	0,1	15	GOLM1	-	membrane
464	9	1,369	0,1	15	GKAP1	-	-
752	19	1,366	0,322	40	MIA	-	extracellular region
752	19	1,366	0,322	40	SHKBP1	WD repeat domain containing	-
752	19	1,366	0,322	40	TTC9B	Tetratricopeptide (TTC) repeat domain containing	biological_process; cellular_component; molecular_function
752	19	1,366	0,322	40	ADCK4	-	-
752	19	1,366	0,322	40	LTBP4	Latent transforming growth factor, beta binding proteins	extracellular region
752	19	1,366	0,322	40	PRX	-	membrane; molecular_function; cell junction
752	19	1,366	0,322	40	AKT2	Pleckstrin homology (PH) domain containing	membrane
752	19	1,366	0,322	40	PLD3	-	membrane; catalytic activity
752	19	1,366	0,322	40	SELV	-	-
752	19	1,366	0,322	40	SPTBN4	Pleckstrin homology (PH) domain containing	membrane; reproductive process
752	19	1,366	0,322	40	CNTD2	-	-
752	19	1,366	0,322	40	MIARAB4B	-	-
752	19	1,366	0,322	40	C19ORF54	-	-
752	19	1,366	0,322	40	HIPK4	-	-
752	19	1,366	0,322	40	FBL	-	membrane
752	19	1,366	0,322	40	BLVRB	Short chain dehydrogenase/reductase superfamily / Atypical members	-
752	19	1,366	0,322	40	C19ORF47	-	-
752	19	1,366	0,322	40	LEUTX	Homeoboxes / PRD class	-
752	19	1,366	0,322	40	MAP3K10	Mitogen-activated protein kinase cascade / Kinase kinase kinases	-
752	19	1,366	0,322	40	NUMBL	-	-
752	19	1,366	0,322	40	ITPKC	-	-

752	19	1,366	0,322	40	TIMM50	Serine/threonine phosphatases / CTD aspartate-based phosphatases	membrane
752	19	1,366	0,322	40	DLL3	-	membrane
752	19	1,366	0,322	40	DYRK1B	-	-
752	19	1,366	0,322	40	SNRPA	RNA binding motif (RRM) containing	-
752	19	1,366	0,322	40	PSMC4	Proteasome (prosome, macropain) subunits	membrane
650	15	1,349	0,547	25	SLC24A5	Solute carriers	membrane; response to stimulus
650	15	1,349	0,547	25	TRPM7	Voltage-gated ion channels / Transient receptor potential cation channels	cell; membrane
650	15	1,349	0,547	25	CTXN2	-	membrane
650	15	1,349	0,547	25	SECISBP2L	-	-
650	15	1,349	0,547	25	SEMA6D	Semaphorins	membrane; chemorepellent activity
650	15	1,349	0,547	25	SLC27A2	Solute carriers	membrane; catalytic activity; metabolic process
650	15	1,349	0,547	25	FAM227B	-	-
650	15	1,349	0,547	25	DTWD1	-	-
650	15	1,349	0,547	25	HDC	-	catalytic activity
650	15	1,349	0,547	25	SHC4	SH2 domain containing	membrane; synapse; cell junction
650	15	1,349	0,547	25	ATP8B4	ATPases / P-type	membrane
650	15	1,349	0,547	25	USP8	Ubiquitin-specific peptidases	membrane
650	15	1,349	0,547	25	AP4E1	-	membrane
650	15	1,349	0,547	25	COPS2	-	signal transducer activity
650	15	1,349	0,547	25	CEP152	-	-
650	15	1,349	0,547	25	MYEF2	RNA binding motif (RRM) containing	-
650	15	1,349	0,547	25	SLC12A1	Solute carriers	membrane; transporter activity
650	15	1,349	0,547	25	DUT	-	-
650	15	1,349	0,547	25	GALK2	-	metabolic process
650	15	1,349	0,547	25	SPPL2A	-	membrane
650	15	1,349	0,547	25	FBN1	-	extracellular region
650	15	1,349	0,547	25	FGF7	Endogenous ligands	chemoattractant activity; extracellular region
650	15	1,349	0,547	25	USP50	Ubiquitin-specific peptidases	-
650	15	1,349	0,547	25	EID1	-	-
650	15	1,349	0,547	25	GABPB1	Ankyrin repeat domain containing	-
766	20	1,333	0,544	33	CRNKL1	-	-
766	20	1,333	0,544	33	SNRPB2	RNA binding motif (RRM) containing	-
766	20	1,333	0,544	33	PCSK2	-	membrane
766	20	1,333	0,544	33	SEC23B	-	membrane
766	20	1,333	0,544	33	POLR3F	RNA polymerase subunits	immune system process
766	20	1,333	0,544	33	DSTN	-	-
766	20	1,333	0,544	33	SLC24A3	Solute carriers	membrane
766	20	1,333	0,544	33	CSRP2BP	-	-
766	20	1,333	0,544	33	SNX5	Sorting nexins	membrane
766	20	1,333	0,544	33	KIF16B	Kinesins	membrane
766	20	1,333	0,544	33	PAX1	Paired boxes	-
766	20	1,333	0,544	33	C20ORF78	-	-
766	20	1,333	0,544	33	ZNF133	Zinc fingers, C2H2-type	-
766	20	1,333	0,544	33	XRN2	-	membrane
766	20	1,333	0,544	33	RRBP1	-	membrane
766	20	1,333	0,544	33	INSM1	-	-
766	20	1,333	0,544	33	CFAP61	-	-
766	20	1,333	0,544	33	OVOL2	Zinc fingers, C2H2-type	-
766	20	1,333	0,544	33	MGME1	-	-
766	20	1,333	0,544	33	OTOR	-	extracellular region
766	20	1,333	0,544	33	RBBP9	-	-
766	20	1,333	0,544	33	BFSP1	Intermediate filaments type VI, eye lens intermediate filaments	biological_process; membrane; structural molecule activity
766	20	1,333	0,544	33	NKX24	Homeoboxes / ANTP class : NKL subclass	-
766	20	1,333	0,544	33	NKX22	Homeoboxes / ANTP class : NKL subclass	-
766	20	1,333	0,544	33	NAA20	N(alpha)-acetyltransferase subunits	-
766	20	1,333	0,544	33	DZANK1	Ankyrin repeat domain containing	-
766	20	1,333	0,544	33	DTD1	-	membrane
766	20	1,333	0,544	33	PET117	-	-
766	20	1,333	0,544	33	BANF2	-	-
766	20	1,333	0,544	33	RIN2	-	cellular_component
766	20	1,333	0,544	33	RALGAPA2	-	-
766	20	1,333	0,544	33	SCP2D1	-	-
577	12	1,318	1,306	102	COQ10A	-	membrane
577	12	1,318	1,306	102	STAT2	SH2 domain containing	signal transducer activity
577	12	1,318	1,306	102	ZBTB39	Zinc fingers, C2H2-type	-
577	12	1,318	1,306	102	METTL21B	-	-
577	12	1,318	1,306	102	RP11272B17.2	-	-
577	12	1,318	1,306	102	CD63	Tetraspanins	membrane; extracellular region
577	12	1,318	1,306	102	RBMS2	RNA binding motif (RRM) containing	-
577	12	1,318	1,306	102	METTL1	-	-

577	12	1,318	1,306	102	TMEM194A	-	-
577	12	1,318	1,306	102	TSPAN31	Tetraspanins	membrane
577	12	1,318	1,306	102	PMEL	-	membrane; extracellular region
577	12	1,318	1,306	102	SLC26A10	Solute carriers	membrane
577	12	1,318	1,306	102	Feb-05	RING-type (C3HC4) zinc fingers	-
577	12	1,318	1,306	102	ERBB3	-	membrane; extracellular region
577	12	1,318	1,306	102	AVIL	-	-
577	12	1,318	1,306	102	R3HDM2	-	-
577	12	1,318	1,306	102	MYL6	Myosins / Light chain	membrane
577	12	1,318	1,306	102	PIP4K2C	-	membrane
577	12	1,318	1,306	102	MMP19	-	extracellular region
577	12	1,318	1,306	102	WIBG	-	-
577	12	1,318	1,306	102	OS9	-	-
577	12	1,318	1,306	102	RP11123K3.4	-	-
577	12	1,318	1,306	102	NAB2	-	-
577	12	1,318	1,306	102	MBD6	-	-
577	12	1,318	1,306	102	SPRYD4	-	biological_process; molecular_function
577	12	1,318	1,306	102	LRIG3	Immunoglobulin superfamily / I-set domain containing	membrane
577	12	1,318	1,306	102	PTGES3	-	-
577	12	1,318	1,306	102	DCTN2	-	membrane
577	12	1,318	1,306	102	CYP27B1	Cytochrome P450s	membrane
577	12	1,318	1,306	102	ARHGEF25	Rho guanine nucleotide exchange factors	membrane
577	12	1,318	1,306	102	CTDSP2	Serine/threonine phosphatases / CTD aspartate-based phosphatases	-
577	12	1,318	1,306	102	NABP2	-	-
577	12	1,318	1,306	102	BLOC1S1	Biogenesis of lysosomal organelles complex-1 subunits	membrane
577	12	1,318	1,306	102	RP11362K2.2	-	-
577	12	1,318	1,306	102	PRIM1	-	membrane
577	12	1,318	1,306	102	RP11571M6.15	-	-
577	12	1,318	1,306	102	CDK2	Cyclin-dependent kinases	-
577	12	1,318	1,306	102	IKZF4	Zinc fingers, C2H2-type	-
577	12	1,318	1,306	102	GPR182	GPCR / Class A : Orphans	membrane; signal transducer activity
577	12	1,318	1,306	102	METTL7B	-	metabolic process
577	12	1,318	1,306	102	PAN2	Ubiquitin-specific peptidases	-
577	12	1,318	1,306	102	RP11644F5.10	-	-
577	12	1,318	1,306	102	ANKRD52	Serine/threonine phosphatases / Protein phosphatase 6, regulatory subunits	-
577	12	1,318	1,306	102	XRCC6BP1	-	-
577	12	1,318	1,306	102	APOF	Apolipoproteins	extracellular region
577	12	1,318	1,306	102	CDK4	Cyclin-dependent kinases	membrane
577	12	1,318	1,306	102	ARHGAP9	Pleckstrin homology (PH) domain containing	-
577	12	1,318	1,306	102	DGKA	EF-hand domain containing	membrane
577	12	1,318	1,306	102	NXPH4	-	cellular_component; molecular_function; extracellular region
577	12	1,318	1,306	102	AGAP2	Pleckstrin homology (PH) domain containing	membrane
577	12	1,318	1,306	102	SHMT2	-	membrane; catalytic activity
577	12	1,318	1,306	102	DDIT3	-	-
577	12	1,318	1,306	102	TSFM	-	-
577	12	1,318	1,306	102	LRP1	Low density lipoprotein receptors	membrane
577	12	1,318	1,306	102	SMARCC2	-	-
577	12	1,318	1,306	102	SLC16A7	Solute carriers	membrane
577	12	1,318	1,306	102	SUOX	-	-
577	12	1,318	1,306	102	GLS2	Ankyrin repeat domain containing	-
577	12	1,318	1,306	102	STAC3	-	-
577	12	1,318	1,306	102	TAC3	Endogenous ligands	extracellular region
577	12	1,318	1,306	102	CS	-	membrane
577	12	1,318	1,306	102	STAT6	SH2 domain containing	signal transducer activity
577	12	1,318	1,306	102	ITGA7	Integrins	membrane
577	12	1,318	1,306	102	MYO1A	Myosins / Myosin superfamily : Class I	-
577	12	1,318	1,306	102	MARS	Aminoacyl tRNA synthetases / Class I	membrane
577	12	1,318	1,306	102	RAB5B	RAB, member RAS oncogene	membrane
577	12	1,318	1,306	102	RPL41	L ribosomal proteins	-
577	12	1,318	1,306	102	NDUFA4L2	-	membrane
577	12	1,318	1,306	102	BAZ2A	Zinc fingers, PHD-type	-
577	12	1,318	1,306	102	RNF41	RING-type (C3HC4) zinc fingers	protein tag
577	12	1,318	1,306	102	ZC3H10	Zinc fingers, CCCH-type domain containing	-
577	12	1,318	1,306	102	TIMELESS	-	rhythmic process
577	12	1,318	1,306	102	GDF11	-	cellular_component; growth; extracellular region
577	12	1,318	1,306	102	DTX3	RING-type (C3HC4) zinc fingers	-
577	12	1,318	1,306	102	ORMDL2	-	membrane
577	12	1,318	1,306	102	ATP5B	Mitochondrial respiratory chain complex / Complex V	membrane; transporter activity
577	12	1,318	1,306	102	MIP	Ion channels / Aquaporins	membrane; transporter activity; cell junction; response to stimulus
577	12	1,318	1,306	102	RPS26	S ribosomal proteins	membrane

577	12	1,318	1,306	102	NACA	-	-
577	12	1,318	1,306	102	SLC39A5	Solute carriers	membrane; molecular_function
577	12	1,318	1,306	102	ESYT1	Synaptotagmins	membrane
577	12	1,318	1,306	102	MYL6B	Myosins / Light chain	-
577	12	1,318	1,306	102	GLI1	Zinc fingers, C2H2-type	-
577	12	1,318	1,306	102	KIF5A	Kinesins	membrane
577	12	1,318	1,306	102	IL23A	Interleukins and interleukin receptors	extracellular region; immune system process
577	12	1,318	1,306	102	B4GALNT1	Glycosyltransferase family 2 domain containing	membrane
554	11	1,275	1,418	82	SLC37A4	Solute carriers	membrane; transporter activity
554	11	1,275	1,418	82	RPS25	S ribosomal proteins	-
554	11	1,275	1,418	82	MCAM	Immunoglobulin superfamily / V-set domain containing	membrane
554	11	1,275	1,418	82	ARCN1	-	membrane
554	11	1,275	1,418	82	DDX6	DEAD-boxes	membrane
554	11	1,275	1,418	82	PDZD3	-	membrane
554	11	1,275	1,418	82	BUD13	-	-
554	11	1,275	1,418	82	NLRX1	Nucleotide-binding domain and leucine rich repeat containing	membrane; immune system process
554	11	1,275	1,418	82	OAF	-	-
554	11	1,275	1,418	82	DSCAML1	Immunoglobulin superfamily / I-set domain containing	membrane; synapse; cell junction
554	11	1,275	1,418	82	TRAPPC4	Trafficking protein particle complex	synapse
554	11	1,275	1,418	82	CD3G	CD molecules	membrane
554	11	1,275	1,418	82	TAGLN	-	-
554	11	1,275	1,418	82	THY1	Immunoglobulin superfamily / Immunoglobulin-like domain containing	membrane
554	11	1,275	1,418	82	PCSK7	-	membrane
554	11	1,275	1,418	82	VPS11	RING-type (C3HC4) zinc fingers	membrane
554	11	1,275	1,418	82	KMT2A	Zinc fingers, PHD-type	rhythmic process
554	11	1,275	1,418	82	CBL	RING-type (C3HC4) zinc fingers	membrane; signal transducer activity
554	11	1,275	1,418	82	PVRL1	Immunoglobulin superfamily / V-set domain containing	-
554	11	1,275	1,418	82	MFRP	-	membrane; molecular_function
554	11	1,275	1,418	82	SIDT2	-	membrane
554	11	1,275	1,418	82	TMPRSS13	Serine peptidases / Transmembrane	membrane
554	11	1,275	1,418	82	FXYD2	-	membrane; transporter activity
554	11	1,275	1,418	82	APOA5	Apolipoproteins	extracellular region
554	11	1,275	1,418	82	TRIM29	Tripartite motif containing / Tripartite motif containing	-
554	11	1,275	1,418	82	TECTA	-	membrane; extracellular region
554	11	1,275	1,418	82	RP11770J1.4	-	-
554	11	1,275	1,418	82	HYOU1	Heat shock proteins / HSP70	membrane; extracellular region
554	11	1,275	1,418	82	TTC36	Tetratricopeptide (TTC) repeat domain containing	-
554	11	1,275	1,418	82	TMPRSS4	Serine peptidases / Transmembrane	membrane
554	11	1,275	1,418	82	SCN4B	Immunoglobulin superfamily / V-set domain containing	membrane
554	11	1,275	1,418	82	HMBS	-	-
554	11	1,275	1,418	82	RNF214	RING-type (C3HC4) zinc fingers	-
554	11	1,275	1,418	82	USP2	Ubiquitin-specific peptidases	rhythmic process
554	11	1,275	1,418	82	GRIK4	Ligand-gated ion channels / Glutamate receptors, ionotropic	membrane; synapse; cell junction
554	11	1,275	1,418	82	BCL9L	-	-
554	11	1,275	1,418	82	TBCEL	-	-
554	11	1,275	1,418	82	FOXR1	Forkhead boxes	-
554	11	1,275	1,418	82	MFRP	-	membrane; molecular_function
554	11	1,275	1,418	82	APOC3	Apolipoproteins	extracellular region
554	11	1,275	1,418	82	AP000679.2	-	-
554	11	1,275	1,418	82	TMEM25	Immunoglobulin superfamily / C2-set domain containing	membrane; extracellular region
554	11	1,275	1,418	82	DPAGT1	-	membrane
554	11	1,275	1,418	82	CXCR5	GPCR / Class A : Chemokine receptors : C-X-C motif	membrane; signal transducer activity
554	11	1,275	1,418	82	SIK3	-	-
554	11	1,275	1,418	82	CCDC153	-	-
554	11	1,275	1,418	82	CD3E	CD molecules	membrane; immune system process; signal transducer activity
554	11	1,275	1,418	82	IFT46	Intraflagellar transport homologs	biological_process; molecular_function
554	11	1,275	1,418	82	CD3D	CD molecules	membrane
554	11	1,275	1,418	82	SC5D	Fatty acid hydroxylase domain containing	membrane
554	11	1,275	1,418	82	POU2F3	Homeoboxes / POU class	-
554	11	1,275	1,418	82	TMEM136	-	membrane
554	11	1,275	1,418	82	PAFAH1B2	-	-
554	11	1,275	1,418	82	H2AFX	Histones / Replication-independent	-
554	11	1,275	1,418	82	SCN2B	Immunoglobulin superfamily / V-set domain containing	membrane
554	11	1,275	1,418	82	ABCG4	ATP binding cassette transporters / subfamily G	membrane
554	11	1,275	1,418	82	CCDC84	-	-
554	11	1,275	1,418	82	AMICA1	Immunoglobulin superfamily / V-set domain containing	-
554	11	1,275	1,418	82	ATP5L	Mitochondrial respiratory chain complex / Complex V	membrane
554	11	1,275	1,418	82	CEP164	-	-
554	11	1,275	1,418	82	TREH	-	membrane; catalytic activity; metabolic process
554	11	1,275	1,418	82	C2CD2L	-	biological_process; cellular_component; membrane; molecular_function

554	11	1,275	1,418	82	ZPR1	-	-
554	11	1,275	1,418	82	APOA4	Apolipoproteins	antioxidant activity; extracellular region
554	11	1,275	1,418	82	APOA1	Apolipoproteins	chemorepellent activity; extracellular region
554	11	1,275	1,418	82	IL10RA	Interleukins and interleukin receptors	membrane; signal transducer activity
554	11	1,275	1,418	82	PHLDB1	Pleckstrin homology (PH) domain containing	-
554	11	1,275	1,418	82	MPZL3	Immunoglobulin superfamily / V-set domain containing	membrane
554	11	1,275	1,418	82	RNF26	RING-type (C3HC4) zinc fingers	biological_process; cellular_component; membrane
554	11	1,275	1,418	82	HINFP	Zinc fingers, C2H2-type	-
554	11	1,275	1,418	82	UPK2	-	membrane
554	11	1,275	1,418	82	ARHGEF12	Rho guanine nucleotide exchange factors	membrane
554	11	1,275	1,418	82	C1QTNF5	-	membrane; extracellular region
554	11	1,275	1,418	82	MPZL2	Immunoglobulin superfamily / V-set domain containing	membrane
554	11	1,275	1,418	82	UBE4A	U-box domain containing	-
554	11	1,275	1,418	82	RP11770J1.5	-	-
286	5	1,259	0,055	25	ARHGEF37	Rho guanine nucleotide exchange factors	-
286	5	1,259	0,055	25	CSF1R	Immunoglobulin superfamily / Immunoglobulin-like domain containing	membrane; immune system process
286	5	1,259	0,055	25	SLC6A7	Solute carriers	membrane
286	5	1,259	0,055	25	SH3TC2	Tetratricopeptide (TTC) repeat domain containing	-
286	5	1,259	0,055	25	SLC26A2	Solute carriers	membrane
286	5	1,259	0,055	25	PPARGC1B	RNA binding motif (RRM) containing	-
286	5	1,259	0,055	25	ABLIM3	-	-
286	5	1,259	0,055	25	ARSI	Arylsulfatase family	catalytic activity; metabolic process; extracellular region
286	5	1,259	0,055	25	PDGFRB	Immunoglobulin superfamily / I-set domain containing	membrane
286	5	1,259	0,055	25	PDE6A	Phosphodiesterases	membrane; response to stimulus
286	5	1,259	0,055	25	AFAP1L1	Pleckstrin homology (PH) domain containing	cell junction
286	5	1,259	0,055	25	TIGD6	-	-
286	5	1,259	0,055	25	HTR4	5-HT (serotonin) receptors	membrane; signal transducer activity
286	5	1,259	0,055	25	CDX1	Homeoboxes / ANTP class : HOXL subclass	-
286	5	1,259	0,055	25	HMGXB3	High mobility group / Non-canonical	biological_process; cellular_component
286	5	1,259	0,055	25	CSNK1A1	-	membrane
286	5	1,259	0,055	25	SPINK7	Serine peptidase inhibitors, Kazal type	extracellular region
286	5	1,259	0,055	25	ADRB2	GPCR / Class A : Adrenoceptors : beta	membrane; signal transducer activity
286	5	1,259	0,055	25	IL17B	Interleukins and interleukin receptors	extracellular region
286	5	1,259	0,055	25	SPINK9	Serine peptidase inhibitors, Kazal type	extracellular region
286	5	1,259	0,055	25	CAMK2A	-	membrane; synapse; cell junction
286	5	1,259	0,055	25	FBXO38	F-boxes / "other"	-
286	5	1,259	0,055	25	GRPEL2	-	-
286	5	1,259	0,055	25	PCYOX1L	-	membrane; extracellular region
697	17	1,244	0,867	43	MPDU1	-	membrane
697	17	1,244	0,039	43	TNK1	-	membrane; signal transducer activity
697	17	1,244	0,039	43	NLGN2	-	membrane; synapse; cell junction
697	17	1,244	0,039	43	C17ORF74	-	-
697	17	1,244	0,039	43	RP111099M24.[illegible]	-	-
697	17	1,244	0,039	43	SOX15	SRY (sex determining region Y)-boxes	-
697	17	1,244	0,039	43	EFNB3	Ephrins	membrane
697	17	1,244	0,039	43	TP53	-	membrane; rhythmic process
697	17	1,244	0,039	43	CNTROB	-	-
697	17	1,244	0,039	43	CHD3	Zinc fingers, PHD-type	-
697	17	1,244	0,039	43	SENP3	-	-
697	17	1,244	0,039	43	TRAPPC1	Trafficking protein particle complex	-
697	17	1,244	0,039	43	SAT2	-	-
697	17	1,244	0,039	43	ACAP1	Pleckstrin homology (PH) domain containing	membrane
697	17	1,244	0,039	43	CYB5D1	-	-
697	17	1,244	0,039	43	POLR2A	RNA polymerase subunits	-
697	17	1,244	0,039	43	TMEM88	-	membrane
697	17	1,244	0,039	43	ATP1B2	ATPases / P-type	membrane
697	17	1,244	0,039	43	KCTD11	-	-
697	17	1,244	0,039	43	TMEM102	-	membrane
697	17	1,244	0,039	43	FXR2	-	membrane
697	17	1,244	0,039	43	SHBG	-	extracellular region
697	17	1,244	0,039	43	WRAP53	WD repeat domain containing	-
697	17	1,244	0,039	43	ZBTB4	Zinc fingers, C2H2-type	-
697	17	1,244	0,039	43	CD68	CD molecules	membrane
697	17	1,244	0,039	43	FGF11	-	extracellular region
697	17	1,244	0,039	43	SLC35G6	Solute carriers	membrane
697	17	1,244	0,039	43	EIF4A1	DEAD-boxes	membrane
697	17	1,244	0,039	43	TMEM256PLSC	-	-
697	17	1,244	0,039	43	NAA38	N(alpha)-acetyltransferase subunits	-
697	17	1,244	0,039	43	SPEM1	-	membrane
697	17	1,244	0,039	43	KCNAB3	Potassium channels	-

697	17	1,244	0,039	43	DNAH2	Axonemal dyneins	-
697	17	1,244	0,039	43	TMEM95	-	membrane
697	17	1,244	0,039	43	GUCY2D	-	membrane; response to stimulus
697	17	1,244	0,039	43	CHRNB1	Ligand-gated ion channels / Acetylcholine receptors, nicotinic	membrane; synapse; cell junction
697	17	1,244	0,039	43	KDM6B	Chromatin-modifying enzymes / K-demethylases	-
188	3	1,201	0,039	16	PIK3CA	-	-
188	3	1,201	0,039	16	CCDC39	-	-
188	3	1,201	0,039	16	SOX2	SRY (sex determining region Y)-boxes	-
188	3	1,201	0,039	16	FXR1	-	membrane
188	3	1,201	0,039	16	USP13	Ubiquitin-specific peptidases	-
188	3	1,201	0,039	16	ACTL6A	INO80 complex subunits	-
188	3	1,201	0,039	16	ZMAT3	Zinc fingers, matrin-type	-
188	3	1,201	0,039	16	MRPL47	Mitochondrial ribosomal proteins / large subunits	biological_process; cellular_component; molecular_function
188	3	1,201	0,039	16	MFN1	-	membrane
188	3	1,201	0,039	16	GNB4	WD repeat domain containing	signal transducer activity
188	3	1,201	0,039	16	NDUFB5	Mitochondrial respiratory chain complex / Complex I	membrane
188	3	1,201	0,039	16	DNAJC19	Heat shock proteins / DNAJ (HSP40)	membrane
188	3	1,201	0,039	16	TTC14	Tetratricopeptide (TTC) repeat domain containing	-
188	3	1,201	0,039	16	ZNF639	Zinc fingers, C2H2-type	-
188	3	1,201	0,039	16	PEX5L	Tetratricopeptide (TTC) repeat domain containing	membrane
169	3	1,199	0,474	35	ABHD10	Abhydrolase domain containing	-
169	3	1,199	0,474	35	C3ORF17	-	-
169	3	1,199	0,474	35	TMPRSS7	Serine peptidases / Transmembrane	membrane; extracellular region
169	3	1,199	0,474	35	GRAMD1C	-	membrane
169	3	1,199	0,474	35	GTPBP8	-	-
169	3	1,199	0,474	35	TIGIT	Immunoglobulin superfamily / V-set domain containing	membrane
169	3	1,199	0,474	35	SLC9C1	Solute carriers	membrane
169	3	1,199	0,474	35	BOC	Immunoglobulin superfamily / I-set domain containing	membrane
169	3	1,199	0,474	35	ZNF80	Zinc fingers, C2H2-type	-
169	3	1,199	0,474	35	CD200	Immunoglobulin superfamily / Immunoglobulin-like domain containing	membrane
169	3	1,199	0,474	35	ZBTB20	Zinc fingers, C2H2-type	-
169	3	1,199	0,474	35	CD96	Immunoglobulin superfamily / Immunoglobulin-like domain containing	membrane
169	3	1,199	0,474	35	NAA50	N(alpha)-acetyltransferase subunits	-
169	3	1,199	0,474	35	SIDT1	-	membrane
169	3	1,199	0,474	35	KIAA1407	-	-
169	3	1,199	0,474	35	PVRL3	Immunoglobulin superfamily / V-set domain containing	-
169	3	1,199	0,474	35	PLCXD2	-	signal transducer activity
169	3	1,199	0,474	35	KIAA2018	-	-
169	3	1,199	0,474	35	ZDHHC23	Zinc fingers, DHHC-type	membrane
169	3	1,199	0,474	35	C3ORF52	-	-
169	3	1,199	0,474	35	QTRTD1	-	-
169	3	1,199	0,474	35	ATP6V1A	ATPases / V-type	-
169	3	1,199	0,474	35	SPICE1	-	-
169	3	1,199	0,474	35	TAGLN3	-	-
169	3	1,199	0,474	35	PHLDB2	Pleckstrin homology (PH) domain containing	membrane
169	3	1,199	0,474	35	SLC35A5	Solute carriers	membrane
169	3	1,199	0,474	35	CFAP44	WD repeat domain containing	-
169	3	1,199	0,474	35	ATG3	-	-
169	3	1,199	0,474	35	ZBED2	Zinc fingers, BED-type	-
169	3	1,199	0,474	35	BTLA	Immunoglobulin superfamily / Immunoglobulin-like domain containing	membrane; immune system process
169	3	1,199	0,474	35	CCDC80	-	membrane; extracellular region
169	3	1,199	0,474	35	GCSAM	-	membrane
169	3	1,199	0,474	35	DRD3	GPCR / Class A : Dopamine receptors	membrane; signal transducer activity
377	7	1,192	0,094	15	MDH2	-	membrane; catalytic activity
377	7	1,192	0,094	15	CCL24	Endogenous ligands	extracellular region
377	7	1,192	0,094	15	CCL26	Endogenous ligands	extracellular region
377	7	1,192	0,094	15	POMZP3	-	biological_process; cellular_component; molecular_function
377	7	1,192	0,094	15	SSC4D	-	membrane; extracellular region
377	7	1,192	0,094	15	SRRM3	-	-
377	7	1,192	0,094	15	YWHAG	-	membrane
377	7	1,192	0,094	15	ZP3	Zona pellucida glycoproteins	membrane; extracellular region; signal transducer activity
377	7	1,192	0,094	15	DTX2	RING-type (C3HC4) zinc fingers	extracellular region
377	7	1,192	0,094	15	POR	-	membrane; electron carrier activity
377	7	1,192	0,094	15	RHBDD2	-	membrane
377	7	1,192	0,094	15	UPK3B	-	membrane
377	7	1,192	0,094	15	HSPB1	Heat shock proteins / HSPB	-
377	7	1,192	0,094	15	HIP1	-	membrane
377	7	1,192	0,094	15	STYXL1	Protein tyrosine phosphatases / Class I Cys-based PTPs : MAP kinase phosphatases	-
178	3	1,188	0,09	22	PCOLCE2	-	extracellular region
178	3	1,188	0,09	22	C3ORF58	-	-

178	3	1,188	0,09	22	PXYLP1	-	membrane
178	3	1,188	0,09	22	ATP1B3	CD molecules	membrane
178	3	1,188	0,09	22	RASA2	Pleckstrin homology (PH) domain containing	-
178	3	1,188	0,09	22	TRPC1	Voltage-gated ion channels / Transient receptor potential cation channels	membrane
178	3	1,188	0,09	22	U2SURP	RNA binding motif (RRM) containing	-
178	3	1,188	0,09	22	SLC25A36	Solute carriers	membrane
178	3	1,188	0,09	22	TRIM42	Tripartite motif containing / Tripartite motif containing	-
178	3	1,188	0,09	22	PLS1	EF-hand domain containing	-
178	3	1,188	0,09	22	CHST2	Sulfotransferases, membrane-bound	membrane
178	3	1,188	0,09	22	ATR	-	-
178	3	1,188	0,09	22	PAQR9	-	membrane
178	3	1,188	0,09	22	ZBTB38	Zinc fingers, C2H2-type	-
178	3	1,188	0,09	22	XRN1	-	membrane; synapse
178	3	1,188	0,09	22	SLC9A9	Solute carriers	membrane
178	3	1,188	0,09	22	TFDP2	-	-
178	3	1,188	0,09	22	SPSB4	-	-
178	3	1,188	0,09	22	RNF7	RING-type (C3HC4) zinc fingers	-
178	3	1,188	0,09	22	GK5	Glycerol kinases	-
178	3	1,188	0,09	22	GRK7	-	membrane; response to stimulus
178	3	1,188	0,09	22	CLSTN2	Cadherins / Cadherin-related	membrane
44	1	1,176	0,511	37	SCYL3	-	-
44	1	1,176	0,511	37	MAEL	-	-
44	1	1,176	0,511	37	BLZF1	-	-
44	1	1,176	0,511	37	ILDR2	-	membrane
44	1	1,176	0,511	37	GPA33	Immunoglobulin superfamily / V-set domain containing	membrane
44	1	1,176	0,511	37	F5	-	membrane; extracellular region
44	1	1,176	0,511	37	POU2F1	Homeoboxes / POU class	-
44	1	1,176	0,511	37	DPT	-	extracellular region
44	1	1,176	0,511	37	DUSP27	-	-
44	1	1,176	0,511	37	CD247	CD molecules	membrane
44	1	1,176	0,511	37	NME7	-	-
44	1	1,176	0,511	37	TIPRL	-	-
44	1	1,176	0,511	37	METTL18	-	-
44	1	1,176	0,511	37	MPC2	-	membrane
44	1	1,176	0,511	37	RCSD1	-	-
44	1	1,176	0,511	37	MPZL1	Immunoglobulin superfamily / V-set domain containing	membrane; structural molecule activity
44	1	1,176	0,511	37	TADA1	-	-
44	1	1,176	0,511	37	SFT2D2	-	biological_process; membrane; molecular_function
44	1	1,176	0,511	37	SELL	CD molecules	membrane
44	1	1,176	0,511	37	CREG1	-	extracellular region
44	1	1,176	0,511	37	ATP1B1	ATPases / P-type	membrane
44	1	1,176	0,511	37	POGK	-	-
44	1	1,176	0,511	37	CCDC181	-	-
44	1	1,176	0,511	37	TBX19	T-boxes	-
44	1	1,176	0,511	37	SELE	CD molecules	membrane
44	1	1,176	0,511	37	ADCY10	Adenylate cyclases	membrane
44	1	1,176	0,511	37	C1ORF112	-	-
44	1	1,176	0,511	37	METTL11B	-	-
44	1	1,176	0,511	37	GORAB	-	-
44	1	1,176	0,511	37	PRRX1	Homeoboxes / PRD class	-
44	1	1,176	0,511	37	SELP	CD molecules	membrane
44	1	1,176	0,511	37	GPR161	GPCR / Class A : Orphans	membrane; signal transducer activity
44	1	1,176	0,511	37	SLC19A2	Solute carriers	membrane
44	1	1,176	0,511	37	DCAF6	WD repeat domain containing	-
44	1	1,176	0,511	37	KIFAP3	-	-
743	19	1,162	1,236	87	ICAM5	Immunoglobulin superfamily / Immunoglobulin-like domain containing	membrane
743	19	1,162	1,236	87	ATG4D	-	-
743	19	1,162	1,236	87	LPPR2	-	-
743	19	1,162	1,236	87	RAVER1	RNA binding motif (RRM) containing	-
743	19	1,162	1,236	87	ANGPTL6	Fibrinogen C domain containing	extracellular region
743	19	1,162	1,236	87	UBL5	-	biological_process; molecular_function; protein tag
743	19	1,162	1,236	87	CTC398G3.6	-	-
743	19	1,162	1,236	87	KRI1	-	-
743	19	1,162	1,236	87	AP1M2	-	membrane
743	19	1,162	1,236	87	CCDC159	-	-
743	19	1,162	1,236	87	TMED1	-	membrane
743	19	1,162	1,236	87	ACP5	-	-
743	19	1,162	1,236	87	TYK2	-	membrane
743	19	1,162	1,236	87	TMEM205	-	membrane
743	19	1,162	1,236	87	OLFM2	-	membrane; synapse; cell junction; extracellular region

743	19	1,162	1,236	87	C19ORF52	-	-
743	19	1,162	1,236	87	ZNF653	Zinc fingers, C2H2-type	-
743	19	1,162	1,236	87	ELOF1	-	-
743	19	1,162	1,236	87	PDE4A	Phosphodiesterases	membrane
743	19	1,162	1,236	87	SMARCA4	-	membrane
743	19	1,162	1,236	87	C19ORF38	-	-
743	19	1,162	1,236	87	SPC24	-	-
743	19	1,162	1,236	87	CDKN2D	Ankyrin repeat domain containing	-
743	19	1,162	1,236	87	CTD2369P2.12	-	-
743	19	1,162	1,236	87	DOCK6	-	-
743	19	1,162	1,236	87	ZNF561AS1	-	-
743	19	1,162	1,236	87	RGL3	-	-
743	19	1,162	1,236	87	C19ORF66	-	-
743	19	1,162	1,236	87	DNM2	Pleckstrin homology (PH) domain containing	membrane; synapse; cell junction
743	19	1,162	1,236	87	YIPF2	Yip1 domain family	membrane
743	19	1,162	1,236	87	CCDC151	-	-
743	19	1,162	1,236	87	KANK2	KN motif and ankyrin repeat domain containing	-
743	19	1,162	1,236	87	MRPL4	Mitochondrial ribosomal proteins / large subunits	-
743	19	1,162	1,236	87	RDH8	Short chain dehydrogenase/reductase superfamily / Classical SDR fold cluster 2	-
743	19	1,162	1,236	87	ICAM4	Blood group antigens	membrane; extracellular region
743	19	1,162	1,236	87	SWSAP1	-	-
743	19	1,162	1,236	87	PIN1	-	-
743	19	1,162	1,236	87	RAB3D	RAB, member RAS oncogene	membrane
743	19	1,162	1,236	87	ILF3	-	membrane
743	19	1,162	1,236	87	SLC44A2	Solute carriers	membrane; signal transducer activity
743	19	1,162	1,236	87	COL5A3	Collagens	extracellular region
743	19	1,162	1,236	87	EPOR	Fibronectin type III domain containing	membrane; extracellular region
743	19	1,162	1,236	87	CDC37	-	-
743	19	1,162	1,236	87	DNMT1	-	-
743	19	1,162	1,236	87	QTRT1	-	membrane
743	19	1,162	1,236	87	ZNF561	Zinc fingers, C2H2-type	-
743	19	1,162	1,236	87	CNN1	-	-
743	19	1,162	1,236	87	CARM1	Protein arginine methyltransferases	-
743	19	1,162	1,236	87	KEAP1	Kelch-like	-
743	19	1,162	1,236	87	C19ORF80	-	-
743	19	1,162	1,236	87	ZGLP1	GATA zinc finger domain containing	-
743	19	1,162	1,236	87	ELAVL3	RNA binding motif (RRM) containing	-
743	19	1,162	1,236	87	TSPAN16	Tetraspanins	membrane
743	19	1,162	1,236	87	ECSIT	Mitochondrial respiratory chain complex assembly factors	immune system process
743	19	1,162	1,236	87	PPAN	-	-
743	19	1,162	1,236	87	EIF3G	RNA binding motif (RRM) containing	-
743	19	1,162	1,236	87	FBXL12	F-boxes / Leucine-rich repeats	-
743	19	1,162	1,236	87	PRKCSH	EF-hand domain containing	-
79	2	1,14	0,002	26	FAM49A	-	-
79	2	1,14	0,002	26	SDC1	Proteoglycans / Cell Surface : Syndecans	membrane; extracellular region
79	2	1,14	0,002	26	OSR1	Zinc fingers, C2H2-type	-
79	2	1,14	0,002	26	MATN3	-	extracellular region
79	2	1,14	0,002	26	PUM2	-	-
79	2	1,14	0,002	26	DDX1	DEAD-boxes	membrane
79	2	1,14	0,002	26	C2ORF43	-	-
79	2	1,14	0,002	26	GEN1	-	catalytic activity
79	2	1,14	0,002	26	GDF7	-	growth; extracellular region
79	2	1,14	0,002	26	MSGN1	-	-
79	2	1,14	0,002	26	NBAS	-	membrane
79	2	1,14	0,002	26	APOB	Apolipoproteins	extracellular region
79	2	1,14	0,002	26	TTC32	Tetratricopeptide (TTC) repeat domain containing	-
79	2	1,14	0,002	26	WDR35	WD repeat domain containing	-
79	2	1,14	0,002	26	MYCN	Basic helix-loop-helix proteins	-
79	2	1,14	0,002	26	RDH14	Short chain dehydrogenase/reductase superfamily / Classical SDR fold cluster 2	membrane
79	2	1,14	0,002	26	RAD51AP2	-	-
79	2	1,14	0,002	26	AC008271.1	-	-
79	2	1,14	0,002	26	HS1BP3	-	-
79	2	1,14	0,002	26	RHOB	-	membrane
79	2	1,14	0,002	26	LAPTM4A	-	membrane
79	2	1,14	0,002	26	SMC6	Structural maintenance of chromosomes proteins	-
297	6	1,138	0,862	48	C6ORF201	-	-
297	6	1,138	0,862	48	CAGE1	-	-
297	6	1,138	0,862	48	FARS2	Aminoacyl tRNA synthetases / Class II	-
297	6	1,138	0,862	48	HUS1B	-	-
297	6	1,138	0,862	48	NRN1	-	membrane; synapse; cell junction

297	6	1,138	0,862	48	LY86	-	extracellular region; immune system process
297	6	1,138	0,862	48	BMP6	Endogenous ligands	growth; extracellular region
297	6	1,138	0,862	48	DSP	-	membrane; cell junction; structural molecule activity
297	6	1,138	0,862	48	TXNDC5	Protein disulfide isomerases	cell
297	6	1,138	0,862	48	F13A1	Transglutaminases	extracellular region
297	6	1,138	0,862	48	RREB1	Zinc fingers, C2H2-type	-
297	6	1,138	0,862	48	RPP40	-	-
297	6	1,138	0,862	48	GMDS	Short chain dehydrogenase/reductase superfamily / Extended SDR fold	-
297	6	1,138	0,862	48	NQO2	-	electron carrier activity
297	6	1,138	0,862	48	RIOK1	-	-
297	6	1,138	0,862	48	PSMG4	-	-
297	6	1,138	0,862	48	FOXF2	Forkhead boxes	-
297	6	1,138	0,862	48	PXDC1	-	-
297	6	1,138	0,862	48	WRNIP1	ATPases / AAA-type	membrane
297	6	1,138	0,862	48	SLC22A23	Solute carriers	membrane; transporter activity
297	6	1,138	0,862	48	PRPF4B	-	-
297	6	1,138	0,862	48	EXOC2	-	membrane
297	6	1,138	0,862	48	SNRNP48	-	-
297	6	1,138	0,862	48	PPP1R3G	Serine/threonine phosphatases / Protein phosphatase 1, regulatory subunits	-
297	6	1,138	0,862	48	FOXC1	Forkhead boxes	-
297	6	1,138	0,862	48	FAM50B	-	-
297	6	1,138	0,862	48	LYRM4	LYR motif containing	-
297	6	1,138	0,862	48	SLC35B3	Solute carriers	membrane
297	6	1,138	0,862	48	ECI2	-	membrane; catalytic activity; metabolic process
297	6	1,138	0,862	48	BPHL	-	-
297	6	1,138	0,862	48	CDYL	-	membrane; catalytic activity; metabolic process
297	6	1,138	0,862	48	SSR1	-	membrane
297	6	1,138	0,862	48	FOXQ1	Forkhead boxes	-
297	6	1,138	0,862	48	RIPK1	-	membrane
297	6	1,138	0,862	48	MYLK4	-	-
297	6	1,138	0,862	48	C6ORF195	-	-
297	6	1,138	0,862	48	FAM217A	-	-
744	19	1,126	1,047	59	RNASEH2A	-	-
744	19	1,126	1,047	59	CTD2192J16.2	-	-
744	19	1,126	1,047	59	LYL1	Basic helix-loop-helix proteins	-
744	19	1,126	1,047	59	MRI1	-	-
744	19	1,126	1,047	59	CALR	-	membrane; extracellular region
744	19	1,126	1,047	59	STX10	-	membrane
744	19	1,126	1,047	59	NACC1	BTB/POZ domain containing	-
744	19	1,126	1,047	59	DCAF15	DDB1 and CUL4 associated factors	-
744	19	1,126	1,047	59	ZSWIM4	Zinc fingers, SWIM-type	-
744	19	1,126	1,047	59	FARSA	Aminoacyl tRNA synthetases / Class II	membrane
744	19	1,126	1,047	59	PKN1	-	membrane
744	19	1,126	1,047	59	KLF1	Zinc fingers, C2H2-type	-
744	19	1,126	1,047	59	RAD23A	-	-
744	19	1,126	1,047	59	CCDC130	-	cellular_component
744	19	1,126	1,047	59	PRKACA	-	membrane
744	19	1,126	1,047	59	IL27RA	Interleukins and interleukin receptors	membrane
744	19	1,126	1,047	59	GIPC1	-	membrane
744	19	1,126	1,047	59	WDR83OS	-	membrane
744	19	1,126	1,047	59	C19ORF67	-	-
744	19	1,126	1,047	59	C19ORF53	-	-
744	19	1,126	1,047	59	PALM3	-	membrane
744	19	1,126	1,047	59	DNASE2	-	-
744	19	1,126	1,047	59	RLN3	Endogenous ligands	extracellular region
744	19	1,126	1,047	59	RFX1	-	-
744	19	1,126	1,047	59	NANOS3	-	-
744	19	1,126	1,047	59	MIR1199	-	-
744	19	1,126	1,047	59	GADD45GIP1	-	-
744	19	1,126	1,047	59	RTBDN	-	membrane; extracellular region
744	19	1,126	1,047	59	GCDH	-	electron carrier activity; metabolic process
744	19	1,126	1,047	59	JUNB	basic leucine zipper proteins	cellular process
744	19	1,126	1,047	59	NFIX	-	-
744	19	1,126	1,047	59	IER2	-	-
744	19	1,126	1,047	59	SAMD1	Sterile alpha motif (SAM) domain containing	-
744	19	1,126	1,047	59	PTGER1	GPCR / Class A : Prostanoid receptors	membrane; signal transducer activity
744	19	1,126	1,047	59	CC2D1A	-	membrane; signal transducer activity
744	19	1,126	1,047	59	DHPS	-	-
744	19	1,126	1,047	59	MAST1	-	membrane
744	19	1,126	1,047	59	TRMT1	-	-

744	19	1,126	1,047	59	WDR83	WD repeat domain containing	-
744	19	1,126	1,047	59	DAND5	-	morphogen activity; extracellular region
744	19	1,126	1,047	59	TNPO2	Importins	-
744	19	1,126	1,047	59	MAN2B1	-	catalytic activity; metabolic process
744	19	1,126	1,047	59	LPHN1	GPCR / Class B : Orphans	-
744	19	1,126	1,047	59	BEST2	Ion channels / Chloride channels : Calcium activated : Bestrophins	biological_process; membrane; molecular_function
744	19	1,126	1,047	59	CACNA1A	Voltage-gated ion channels / Calcium channels	membrane
744	19	1,126	1,047	59	C19ORF43	-	-
744	19	1,126	1,047	59	DDX39A	DEAD-boxes	membrane
744	19	1,126	1,047	59	ASF1B	-	-
744	19	1,126	1,047	59	CTD2192J16.24	-	-
744	19	1,126	1,047	59	PRDX2	-	cell; antioxidant activity
744	19	1,126	1,047	59	SYCE2	-	-
744	19	1,126	1,047	59	HOOK2	-	-
744	19	1,126	1,047	59	PODNL1	-	extracellular region
744	19	1,126	1,047	59	C19ORF57	-	-
744	19	1,126	1,047	59	FBXW9	WD repeat domain containing	-
744	19	1,126	1,047	59	ASNA1	-	transporter activity
821	X	1,114	0,328	27	LONRF3	RING-type (C3HC4) zinc fingers	-
821	X	1,114	0,328	27	UPF3B	-	-
821	X	1,114	0,328	27	LAMP2	CD molecules	membrane
821	X	1,114	0,328	27	UBE2A	Ubiquitin-conjugating enzymes E2	-
821	X	1,114	0,328	27	NKRF	G patch domain containing	-
821	X	1,114	0,328	27	CXORF56	-	-
821	X	1,114	0,328	27	TMEM255A	-	membrane
821	X	1,114	0,328	27	ATP1B4	ATPases / P-type	membrane
821	X	1,114	0,328	27	NKAP	-	-
821	X	1,114	0,328	27	RNF113A	RING-type (C3HC4) zinc fingers	-
821	X	1,114	0,328	27	ZCCHC12	Zinc fingers, CCHC domain containing	-
821	X	1,114	0,328	27	CUL4B	-	-
821	X	1,114	0,328	27	ZBTB33	Zinc fingers, C2H2-type	-
821	X	1,114	0,328	27	IL13RA1	Interleukins and interleukin receptors	membrane
821	X	1,114	0,328	27	RHOXF1	Homeoboxes / PRD class	-
821	X	1,114	0,328	27	RPL39	L ribosomal proteins	-
821	X	1,114	0,328	27	SLC25A43	Solute carriers	membrane
821	X	1,114	0,328	27	Aug-02	Septins	-
821	X	1,114	0,328	27	SLC25A5	Solute carriers	membrane; transporter activity
821	X	1,114	0,328	27	SOWAHD	Ankyrin repeat domain containing	-
821	X	1,114	0,328	27	NDUFA1	Mitochondrial respiratory chain complex / Complex I	membrane
821	X	1,114	0,328	27	AKAP14	A-kinase anchor proteins	-
821	X	1,114	0,328	27	PGRMC1	-	membrane
821	X	1,114	0,328	27	DOCK11	Pleckstrin homology (PH) domain containing	-
821	X	1,114	0,328	27	KIAA1210	-	-
294	5	1,105	0,132	40	DDX41	DEAD-boxes	membrane
294	5	1,105	0,132	40	NOP16	-	-
294	5	1,105	0,132	40	GRK6	-	membrane
294	5	1,105	0,132	40	UNC5A	Immunoglobulin superfamily / I-set domain containing	membrane
294	5	1,105	0,132	40	RAB24	RAB, member RAS oncogene	membrane
294	5	1,105	0,132	40	HRH2	GPCR / Class A : Histamine receptors	membrane; signal transducer activity
294	5	1,105	0,132	40	FAF2	UBX domain containing	-
294	5	1,105	0,132	40	FAM193B	-	-
294	5	1,105	0,132	40	NSD1	Zinc fingers, PHD-type	-
294	5	1,105	0,132	40	TSPAN17	Tetraspanins	membrane
294	5	1,105	0,132	40	ARL10	ADP-ribosylation factors-like	-
294	5	1,105	0,132	40	F12	-	extracellular region
294	5	1,105	0,132	40	RGS14	Regulators of G-protein signaling	membrane; synapse; cell junction
294	5	1,105	0,132	40	UIMC1	-	-
294	5	1,105	0,132	40	DOK3	-	membrane
294	5	1,105	0,132	40	HK3	-	cell; catalytic activity; metabolic process
294	5	1,105	0,132	40	SNCB	-	synapse
294	5	1,105	0,132	40	SLC34A1	Solute carriers	membrane
294	5	1,105	0,132	40	PDLIM7	-	-
294	5	1,105	0,132	40	SFXN1	Sideroflexins	membrane
294	5	1,105	0,132	40	GPRIN1	-	membrane
294	5	1,105	0,132	40	MXD3	MAX dimerization proteins	-
294	5	1,105	0,132	40	RNF44	RING-type (C3HC4) zinc fingers	-
294	5	1,105	0,132	40	PRELID1	-	-
294	5	1,105	0,132	40	HIGD2A	-	membrane
294	5	1,105	0,132	40	THOC3	WD repeat domain containing	membrane
294	5	1,105	0,132	40	CLTB	-	membrane; structural molecule activity

294	5	1,105	0,132	40	PFN3	-	-
294	5	1,105	0,132	40	DBN1	-	membrane; cell junction
294	5	1,105	0,132	40	SIMC1	-	-
294	5	1,105	0,132	40	CDHR2	Cadherins / Cadherin-related	membrane; cell junction
294	5	1,105	0,132	40	KIAA1191	-	biological_process; cellular_component; molecular_function
294	5	1,105	0,132	40	LMAN2	-	membrane
294	5	1,105	0,132	40	ZNF346	-	-
294	5	1,105	0,132	40	FGFR4	Immunoglobulin superfamily / I-set domain containing	membrane; extracellular region
294	5	1,105	0,132	40	EIF4E1B	-	-
294	5	1,105	0,132	40	PRR7	-	membrane; synapse; cell junction
294	5	1,105	0,132	40	CPLX2	-	synapse
687	16	1,102	0,485	38	CALB2	EF-hand domain containing	synapse
687	16	1,102	0,485	38	DDX19B	DEAD-boxes	membrane
687	16	1,102	0,485	38	COG4	Components of oligomeric golgi complex	membrane
687	16	1,102	0,485	38	CHST4	Sulfotransferases, membrane-bound	membrane
687	16	1,102	0,485	38	VAC14	-	membrane
687	16	1,102	0,485	38	HYDIN	Serine/threonine phosphatases / Protein phosphatase 1, regulatory subunits	-
687	16	1,102	0,485	38	TAT	-	cellular_component; catalytic activity
687	16	1,102	0,485	38	CMTR2	-	membrane
687	16	1,102	0,485	38	ZNF821	Zinc fingers, C2H2-type	-
687	16	1,102	0,485	38	MTSS1L	-	-
687	16	1,102	0,485	38	HP	-	antioxidant activity; extracellular region; immune system process
687	16	1,102	0,485	38	EXOSC6	-	-
687	16	1,102	0,485	38	PHLPP2	Serine/threonine phosphatases / Protein phosphatases, Mg2+/Mn2+ dependent	membrane; catalytic activity
687	16	1,102	0,485	38	FUK	-	metabolic process
687	16	1,102	0,485	38	ST3GAL2	Sialyltransferases	membrane; extracellular region
687	16	1,102	0,485	38	MARVELD3	-	membrane; cell junction
687	16	1,102	0,485	38	ZFHX3	Homeoboxes / ZF class	-
687	16	1,102	0,485	38	HPR	-	extracellular region
687	16	1,102	0,485	38	DHX38	DEAH-boxes	membrane
687	16	1,102	0,485	38	IL34	Interleukins and interleukin receptors	extracellular region; immune system process
687	16	1,102	0,485	38	PDPR	-	-
687	16	1,102	0,485	38	ATXN1L	-	-
687	16	1,102	0,485	38	PMFBP1	-	-
687	16	1,102	0,485	38	AP1G1	-	membrane; transporter activity
687	16	1,102	0,485	38	ZNF23	Zinc fingers, C2H2-type	-
687	16	1,102	0,485	38	AARS	Aminoacyl tRNA synthetases / Class II	membrane
687	16	1,102	0,485	38	TXNL4B	-	-
687	16	1,102	0,485	38	DHODH	-	membrane; catalytic activity
687	16	1,102	0,485	38	SF3B3	-	-
158	3	1,091	0,037	28	ARHGEF3	Rho guanine nucleotide exchange factors	-
158	3	1,091	0,037	28	CACNA2D3	Calcium channel subunits	membrane
158	3	1,091	0,037	28	PRKCD	-	membrane
158	3	1,091	0,037	28	SELK	-	-
158	3	1,091	0,037	28	IL17RD	Interleukins and interleukin receptors	membrane
158	3	1,091	0,037	28	ARF4	ADP-ribosylation factors	membrane
158	3	1,091	0,037	28	RP11894J14.5	-	-
158	3	1,091	0,037	28	ERC2	-	synapse; cell junction
158	3	1,091	0,037	28	SLMAP	-	membrane
158	3	1,091	0,037	28	TKT	-	catalytic activity; metabolic process
158	3	1,091	0,037	28	WNT5A	Wingless-type MMTV integration sites	membrane; extracellular region
158	3	1,091	0,037	28	DENND6A	DENN/MADD domain containing	-
158	3	1,091	0,037	28	SPATA12	-	-
158	3	1,091	0,037	28	APPL1	Pleckstrin homology (PH) domain containing	membrane
158	3	1,091	0,037	28	CHDH	-	membrane
158	3	1,091	0,037	28	FAM208A	-	-
158	3	1,091	0,037	28	ACTR8	INO80 complex subunits	-
158	3	1,091	0,037	28	CACNA1D	Voltage-gated ion channels / Calcium channels	membrane
158	3	1,091	0,037	28	HESX1	Homeoboxes / PRD class	-
158	3	1,091	0,037	28	SFMBT1	Sterile alpha motif (SAM) domain containing	-
158	3	1,091	0,037	28	LRTM1	-	membrane
158	3	1,091	0,037	28	PDE12	-	-
158	3	1,091	0,037	28	IL17RB	Interleukins and interleukin receptors	membrane; extracellular region
158	3	1,091	0,037	28	ASB14	Ankyrin repeat domain containing	-
158	3	1,091	0,037	28	CCDC66	-	-
158	3	1,091	0,037	28	RFT1	-	membrane
158	3	1,091	0,037	28	DNAH12	Axonemal dyneins	-
415	8	1,053	1,121	77	PPP3CC	Serine/threonine phosphatases / Protein phosphatase, catalytic subunits	-
415	8	1,053	1,121	77	ADRA1A	GPCR / Class A : Adrenoceptors : alpha	membrane; signal transducer activity
415	8	1,053	1,121	77	ADAM28	ADAM metallopeptidase domain containing	membrane; extracellular region

415	8	1,053	1,121	77	HMBOX1	Homeoboxes / HNF class	-
415	8	1,053	1,121	77	BIN3	-	-
415	8	1,053	1,121	77	TNFRSF10C	Tumor necrosis factor receptor superfamily	membrane
415	8	1,053	1,121	77	NUGGC	-	-
415	8	1,053	1,121	77	BNIP3L	-	membrane
415	8	1,053	1,121	77	R3HCC1	-	-
415	8	1,053	1,121	77	EBF2	-	-
415	8	1,053	1,121	77	SCARA5	-	membrane
415	8	1,053	1,121	77	ADAMDEC1	-	cellular_component; extracellular region
415	8	1,053	1,121	77	POLR3D	RNA polymerase subunits	immune system process
415	8	1,053	1,121	77	PBK	-	cellular_component
415	8	1,053	1,121	77	PEBP4	-	membrane
415	8	1,053	1,121	77	LOXL2	-	membrane; electron carrier activity; extracellular region
415	8	1,053	1,121	77	PPP2R2A	WD repeat domain containing	developmental process
415	8	1,053	1,121	77	EXTL3	Exostosin glycosyltransferase family	membrane
415	8	1,053	1,121	77	NEFM	Intermediate filaments type IV	structural molecule activity
415	8	1,053	1,121	77	RHOBTB2	BTB/POZ domain containing	-
415	8	1,053	1,121	77	EPHX2	-	membrane; catalytic activity; metabolic process
415	8	1,053	1,121	77	CHMP7	Charged multivesicular body proteins	-
415	8	1,053	1,121	77	CCDC25	-	-
415	8	1,053	1,121	77	ELP3	Chromatin-modifying enzymes / K-acetyltransferases	catalytic activity
415	8	1,053	1,121	77	NKX26	Homeoboxes / ANTP class : NKL subclass	-
415	8	1,053	1,121	77	FGF17	-	extracellular region
415	8	1,053	1,121	77	REEP4	Receptor accessory proteins	membrane
415	8	1,053	1,121	77	RP11521M14.2	-	-
415	8	1,053	1,121	77	EGR3	Zinc fingers, C2H2-type	-
415	8	1,053	1,121	77	PNOC	Endogenous ligands	extracellular region
415	8	1,053	1,121	77	GNRH1	Endogenous ligands	extracellular region
415	8	1,053	1,121	77	SORBS3	-	cell junction
415	8	1,053	1,121	77	PDLIM2	-	-
415	8	1,053	1,121	77	C8ORF58	-	-
415	8	1,053	1,121	77	DPYSL2	-	membrane
415	8	1,053	1,121	77	CDCA2	-	-
415	8	1,053	1,121	77	CHRNA2	Ligand-gated ion channels / Acetylcholine receptors, nicotinic	membrane; synapse; cell junction
415	8	1,053	1,121	77	KIF13B	Kinesins	-
415	8	1,053	1,121	77	DOCK5	-	membrane
415	8	1,053	1,121	77	ENTPD4	-	membrane
415	8	1,053	1,121	77	BMP1	Bone morphogenetic proteins	extracellular region
415	8	1,053	1,121	77	SCARA3	-	membrane; structural molecule activity
415	8	1,053	1,121	77	ESCO2	-	-
415	8	1,053	1,121	77	FBXO16	F-boxes / "other"	-
415	8	1,053	1,121	77	STC1	-	extracellular region
415	8	1,053	1,121	77	INTS9	-	-
415	8	1,053	1,121	77	PHYHIP	-	-
415	8	1,053	1,121	77	ZNF395	Zinc fingers, C2H2-type	-
415	8	1,053	1,121	77	AC037459.4	-	-
415	8	1,053	1,121	77	LGI3	-	synapse; catalytic activity; cell junction; extracellular region
415	8	1,053	1,121	77	TRIM35	Tripartite motif containing / Tripartite motif containing	molecular_function
415	8	1,053	1,121	77	SLC39A14	Solute carriers	membrane
415	8	1,053	1,121	77	SFTPC	BRICHOS domain containing	membrane; extracellular region
415	8	1,053	1,121	77	NPM2	-	-
415	8	1,053	1,121	77	FAM160B2	-	-
415	8	1,053	1,121	77	CCAR2	-	rhythmic process
415	8	1,053	1,121	77	CLU	-	membrane; extracellular region; immune system process
415	8	1,053	1,121	77	KCTD9	BTB/POZ domain containing	-
415	8	1,053	1,121	77	DMTN	-	membrane
415	8	1,053	1,121	77	PIWIL2	Argonaute/PIWI family	-
415	8	1,053	1,121	77	GFRA2	-	membrane
415	8	1,053	1,121	77	HR	-	-
415	8	1,053	1,121	77	XPO7	Exportins	-
415	8	1,053	1,121	77	NKX31	Homeoboxes / ANTP class : NKL subclass	-
415	8	1,053	1,121	77	SLC25A37	Solute carriers	membrane
415	8	1,053	1,121	77	PTK2B	-	membrane; cell junction; immune system process; signal transducer activity
415	8	1,053	1,121	77	FZD3	GPCR / Class F : Frizzled receptors	membrane; signal transducer activity
415	8	1,053	1,121	77	ADAM7	ADAM metallopeptidase domain containing	membrane
415	8	1,053	1,121	77	DOK2	-	-
415	8	1,053	1,121	77	STMN4	-	-
415	8	1,053	1,121	77	PNMA2	Paraneoplastic Ma antigens	-
640	14	1,043	0,71	48	KLC1	Tetratricopeptide (TTC) repeat domain containing	membrane
640	14	1,043	0,71	48	APOPT1	-	-

640	14	1,043	0,71	48	C14ORF2	-	-
640	14	1,043	0,71	48	NUDT14	Nudix motif containing	-
640	14	1,043	0,71	48	KIF26A	Kinesins	-
640	14	1,043	0,71	48	CINP	-	-
640	14	1,043	0,71	48	PPP1R13B	Serine/threonine phosphatases / Protein phosphatase 1, regulatory subunits	-
640	14	1,043	0,71	48	XRCC3	-	-
640	14	1,043	0,71	48	RD3L	-	-
640	14	1,043	0,71	48	TDRD9	Tudor domain containing	-
640	14	1,043	0,71	48	AKT1	Pleckstrin homology (PH) domain containing	membrane
640	14	1,043	0,71	48	ADSSL1	-	membrane; immune system process
640	14	1,043	0,71	48	ASPG	Ankyrin repeat domain containing	-
640	14	1,043	0,71	48	CKB	-	catalytic activity
640	14	1,043	0,71	48	INF2	-	-
640	14	1,043	0,71	48	RCOR1	-	-
640	14	1,043	0,71	48	EXOC3L4	-	-
640	14	1,043	0,71	48	GPR132	GPCR / Class A : Orphans	membrane; signal transducer activity
640	14	1,043	0,71	48	MARK3	-	membrane
640	14	1,043	0,71	48	SIVA1	-	-
640	14	1,043	0,71	48	TECPR2	-	-
640	14	1,043	0,71	48	HSP90AA1	Heat shock proteins / HSPC	membrane; extracellular region
640	14	1,043	0,71	48	WDR20	WD repeat domain containing	-
640	14	1,043	0,71	48	ZBTB42	Zinc fingers, C2H2-type	-
640	14	1,043	0,71	48	C14ORF180	-	-
640	14	1,043	0,71	48	PPP2R5C	Serine/threonine phosphatases / Protein phosphatase 2, regulatory subunits	-
640	14	1,043	0,71	48	JAG2	-	membrane
640	14	1,043	0,71	48	PLD4	-	membrane; catalytic activity
640	14	1,043	0,71	48	AMN	-	membrane
640	14	1,043	0,71	48	MOK	-	-
640	14	1,043	0,71	48	TRMT61A	-	-
640	14	1,043	0,71	48	DYNC1H1	Cytoplasmic dyneins	membrane
640	14	1,043	0,71	48	C14ORF79	-	-
640	14	1,043	0,71	48	CEP170B	-	-
640	14	1,043	0,71	48	RP1173M18.2	-	-
640	14	1,043	0,71	48	ZNF839	-	-
640	14	1,043	0,71	48	TMEM179	-	membrane
640	14	1,043	0,71	48	ANKRD9	Ankyrin repeat domain containing	-
640	14	1,043	0,71	48	AHNAK2	-	-
640	14	1,043	0,71	48	CDCA4	-	-
640	14	1,043	0,71	48	ZFYVE21	Zinc fingers, FYVE domain containing	cell junction
640	14	1,043	0,71	48	BAG5	-	membrane
640	14	1,043	0,71	48	CDC42BPB	-	membrane; cell junction
640	14	1,043	0,71	48	EIF5	-	-
640	14	1,043	0,71	48	TNFAIP2	-	-
640	14	1,043	0,71	48	TRAF3	-	immune system process; signal transducer activity
691	17	1,026	0,731	69	DPH1	-	-
691	17	1,026	0,731	69	RPA1	-	-
691	17	1,026	0,731	69	TSR1	-	-
691	17	1,026	0,731	69	SGSM2	Small G protein signaling modulators	-
691	17	1,026	0,731	69	ATP2A3	ATPases / P-type	membrane
691	17	1,026	0,731	69	SERPINF2	Serine (or cysteine) peptidase inhibitors	extracellular region
691	17	1,026	0,731	69	PAFAH1B1	WD repeat domain containing	membrane
691	17	1,026	0,731	69	SRR	-	catalytic activity; metabolic process
691	17	1,026	0,731	69	SMYD4	Zinc fingers, MYND-type	-
691	17	1,026	0,731	69	PITPNA	-	-
691	17	1,026	0,731	69	C17ORF97	-	-
691	17	1,026	0,731	69	TIMM22	-	membrane
691	17	1,026	0,731	69	SHPK	-	-
691	17	1,026	0,731	69	RP11676J12.7	-	-
691	17	1,026	0,731	69	TRPV1	Voltage-gated ion channels / Transient receptor potential cation channels	membrane; synapse; cell junction
691	17	1,026	0,731	69	GLOD4	-	-
691	17	1,026	0,731	69	MYO1C	Myosins / Myosin superfamily : Class I	membrane
691	17	1,026	0,731	69	SCARF1	-	membrane
691	17	1,026	0,731	69	WDR81	WD repeat domain containing	-
691	17	1,026	0,731	69	SMG6	-	-
691	17	1,026	0,731	69	BHLHA9	Basic helix-loop-helix proteins	-
691	17	1,026	0,731	69	METTL16	-	-
691	17	1,026	0,731	69	NXN	-	cell
691	17	1,026	0,731	69	MNT	MAX dimerization proteins	-
691	17	1,026	0,731	69	TAX1BP3	-	membrane
691	17	1,026	0,731	69	CTNS	-	membrane

691	17	1,026	0,731	69	GEMIN4	-	membrane
691	17	1,026	0,731	69	SLC43A2	Solute carriers	membrane
691	17	1,026	0,731	69	EMC6	-	membrane
691	17	1,026	0,731	69	HIC1	Zinc fingers, C2H2-type	-
691	17	1,026	0,731	69	GSG2	-	-
691	17	1,026	0,731	69	INPP5K	-	membrane
691	17	1,026	0,731	69	VPS53	-	membrane
691	17	1,026	0,731	69	RAP1GAP2	-	-
691	17	1,026	0,731	69	FAM101B	-	-
691	17	1,026	0,731	69	ITGAE	Integrins	membrane
691	17	1,026	0,731	69	RNMTL1	-	-
691	17	1,026	0,731	69	SERPINF1	Serine (or cysteine) peptidase inhibitors	extracellular region
691	17	1,026	0,731	69	RTN4RL1	-	membrane
691	17	1,026	0,731	69	TUSC5	-	membrane
691	17	1,026	0,731	69	CAMKK1	-	-
691	17	1,026	0,731	69	TRPV1	Voltage-gated ion channels / Transient receptor potential cation channels	membrane; synapse; cell junction
691	17	1,026	0,731	69	ABR	Pleckstrin homology (PH) domain containing	membrane
691	17	1,026	0,731	69	C17ORF85	-	-
691	17	1,026	0,731	69	CRK	SH2 domain containing	membrane
691	17	1,026	0,731	69	PRPF8	-	membrane
691	17	1,026	0,731	69	FAM57A	-	membrane
691	17	1,026	0,731	69	TLCD2	-	membrane
691	17	1,026	0,731	69	YWHAE	-	membrane
691	17	1,026	0,731	69	CLUH	-	-
691	17	1,026	0,731	69	ASPA	-	metabolic process
691	17	1,026	0,731	69	RILP	-	membrane
691	17	1,026	0,731	69	TRPV3	Voltage-gated ion channels / Transient receptor potential cation channels	membrane
